# Supplementary material for: Production of constrained L-cyclo-tetrapeptides by epimerization-resistant direct aminolysis
Source: Nat Commun. 2024 Jun 25;15:5372. doi: 10.1038/s41467-024-49329-3 (PMC11199569; doi:10.1038/s41467-024-49329-3)
Supplement: Supplementary file 1 — Supplementary info [file 41467_2024_49329_MOESM1_ESM.pdf]

Supplementary Information for

Production of Constrained L-Cyclo-tetrapeptides by Epimerization Resistance Direct Aminolysis

Huan Chen, [1] Yuchen Zhang, [2] Yuming Wen, [1] Xinhao Fan, [1] Nicholas Sciolino, [1] Yanyun Lin, [1] Leonard Breindel, [1] Yuanwei Dai, [1] Alexander Shekhtman, [1*] Xiaosong Xue, [2*] and Qiang Zhang [1*]

1. Department of Chemistry, State University of New York, University at Albany, Albany New York 12222, United States
2. Key Laboratory of Organofluorine Chemistry, Shanghai Institute of Organic Chemistry, Chinese Academy of Sciences, 345 Lingling Road, Shanghai, 200032, P. R. China.

*Correspondence to: qzhang5@albany.edu, ashekhtman@albany.edu, xuexs@sioc.ac.cn

Table of Contents

1. Supplementary Methods **3**

1.1 General Information **3**

1.2 Procedures for β-Thiolactone Synthesis **3**

1.3 General Procedures for Peptide Synthesis **6**

1.4 Preparation and Characterization of Peptide Segments **9**

2. Supplementary Discussion **83**

2.1 Thiolactone Mediated Cyclization Compared to Extant Protocols. **83**

2.2 The NMR Structure Determination of **1** and NMR Conformers of Synthesized Cyclopeptides. **90**

2.3 Computational Studies. **111**

2.4. The method of Bioactivity Experiment **127**

3.Supplementary Notes **128**

3.1 Selected NMR Spectrums **128**

4. Supplementary References **165**

1. Supplementary Methods

1.1 General Information

^{1}H NMR spectra were recorded at 500 or 600 MHz at ambient temperature with Chloroform-d ($CDCl_{3}$-*d*), Dimethyl sulfoxide-d6 (DMSO-*d*6) or Methanol-d4 (MeOD-*d4*) (Cambridge Isotope Laboratories, Inc.) as the solvent unless otherwise stated. ^{13}C NMR spectra were recorded at 126 or 150 MHz at ambient temperature with Chloroform-d ($CDCl_{3}$-*d*) and DMSO-*d*6 as the solvent unless otherwise stated. Chemical shifts are reported in parts per million relatives to $CDCl_3$-*d* (^{1}H, δ 7.26; ^{13}C, δ 77.36) DMSO-*d*6 (^{1}H, δ 2.50; ^{13}C, δ 39.51), MeOD-*d4* (^{1}H, δ 3.34; ^{13}C, δ 49.86). All ^{13}C NMR spectra were recorded with complete proton decoupling. Analytical High-Performance Liquid Chromatography (HPLC) and Liquid chromatography-low resolution mass spectrometry (LC-LRMS) were performed using a Waters® 2896 Photodiode array detector and Waters® SQ detector 2 with ZSpray™ source architecture single quadrupole detection system equipped with Waters® e2695 separation module. Analytical thin layer chromatography was performed using 0.25 mm silica gel 60-F plates. Flash chromatography was performed using 200-400 mesh silica gel. (Scientific Absorbents, Inc.) Yields refer to chromatographically and spectroscopically pure materials unless otherwise stated. All other reagents were purchased from Sigma-Aldrich, Alfa Aesar, Chemimpex and Oakwood Chemicals. All reactions were carried out in oven-dried glassware under an argon atmosphere unless otherwise noted. All commercially available materials (Aldrich®, Novabiochem®) were used without further purification. 2,2'-Azobis[2-(2-imidazolin-2-yl) propane] dihydrochloride (VA-044) was purchased from Wako Pure Chemical Industries. HATU was purchased from Genescript® (Piscataway, New Jersey). Bond-Breaker® solution was purchased from ThermoScientific®. Reactions were carried out in Fisherbrand™ premium Microcentrifuge Tube and under an atmosphere of pre-purified dry argon (g). HPLC: All separations involved a mobile phase of 0.05% TFA (v/ v) in water (solvent A)/acetonitrile (solvent B). Analytical LC-MS analyses were performed using a C18 5 μm (150 x 2.0 mm), and Waters Microsorb 300-5, C4 250 x 2.0 mm columns at a flow rate of 0.6 mL/min. LC-MS analyses were performed using a Waters® SQ detector 2 mass detector. Preparative separations were performed using a Dionex Ultimate UHPLC system equipped with a UV detector and Proto reverse phase HPLC column Microsorb 200 C18 5 μm (250 x 10 mm) at a flow rate of 4.75 mL/min or 5.00 mL/min.

1.2 Procedures for β-Thiolactone Synthesis

1.2.1 Synthesis of (*S*)-3-aminothietan-2-one TFA salt (7a)

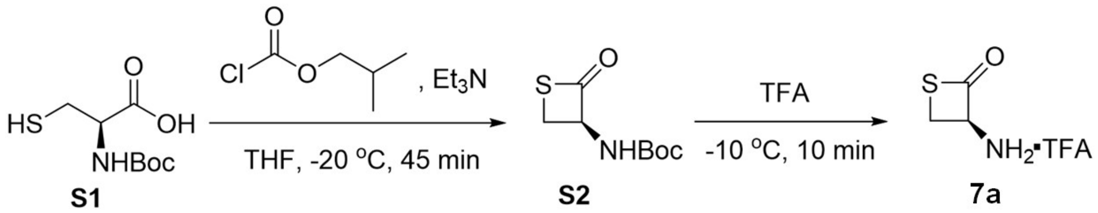

To a solution of Boc-Cys-OH (**S1**) (500 mg, 2.3 mmol) in tetrahydrofuran (10 mL) at 0 °C was added

trimethylamine (345 µL, 2.53 mmol, 1.1 equiv.), then isobutyl chloroformate (339 µL, 2.53 mmol, 1.1 equiv.) was added dropwise. The resulting mixture was stirred at –10 °C for 45 min. The reaction mixture was quenched with 1 M HCl solution and extracted three times with ethyl acetate. The combined organic layers were subsequently washed with brine, dried over anhydrous Na_2SO_4, filtered, and concentrated in vacuo. The residue was purified by silica gel chromatography (hexane/ EtOAc = 3: 1) to afford 210.0 mg (1.05 mmol, 46%) of (S)-O-(tert-Butyl)-N-(thietan-2-on-3-yl)-carbamate (**S2**) as a white solid. The NMR spectra of **S2** are identical to the data as reported in the literature[1].
tert-butyl (*S*)-(2-oxothietan-3-yl) carbamate (**S2**),

^{1}H-NMR ($CDCl_3$, 500 MHz), δ 5.44 (s, 2H), 3.34 – 3.42 (m, 1H), 3.34 (s, 1H), 1.48 (s, 9H).

^{13}C-NMR ($CDCl_3$, 126 MHz), δ 193.52, 154.15, 81.13, 72.23, 28.21 (3C), 27.85.

ESI-HRMS: calcd. for $C_8H_{14}N_1O_3S$, $[M+H]^+$: 204.0689 (*m/z*); found $[M+H]^+$: 204.0684.

S2 (210 mg) was dissolved in cold trifluoroacetic acid (TFA) under -10 °C. After stirring for 10 min under the same temperature, TFA was blown away by air flow. The residual TFA was removed by lyophilizer to yield 200.0 mg (*S*)-3-aminothietan-2-one TFA salt (**7a**) (crude yield = 97%). The crude residual was used in the next step directly.

1.2.2 Synthesis of (*S*)-3-amino-4,4-dimethylthietan-2-one TFA salt (7b)

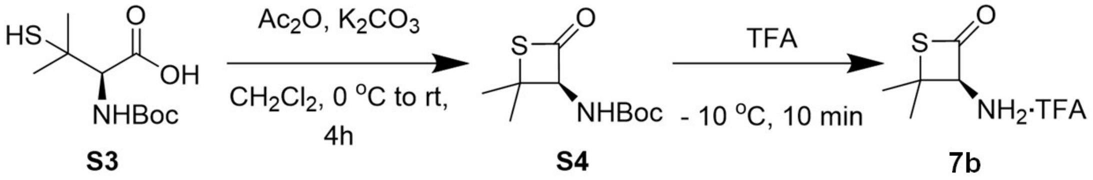

To a solution of L-Boc-penicillamine (**S3**) (680 mg, 2.7 mmol) and K_2CO_3 (221 mg, 1.6 mmol, 2.0 equiv.) in dichloromethane (DCM) (10 mL) at –20 °C, was added acetic anhydride (0.15 mL, 1.6 mmol, 2.0 equiv.) dropwise. The resulting mixture was stirred at –20 °C for 4h. The reaction mixture was quenched with 1 M HCl and extracted three times with dichloromethane. The combined organic layers were washed with brine, dried with anhydrous Na_2SO_4, filtered, and concentrated in vacuo. The residue was purified by silica gel chromatography (hexane/ EtOAc = 3: 2) to yield 411.0 mg (1.78 mmol, 66 %) of tert-butyl (*S*)-(2,2-dimethyl-4-oxothietan-3-yl) carbamate (**S4**) as white amorphous solid.
tert-butyl (*S*)-(2,2-dimethyl-4-oxothietan-3-yl) carbamate (**S4**),

^{1}H-NMR ($CDCl_3$, 500 MHz), δ 5.52 – 5.51 (m, 1H), 5.41 – 5.40 (d, *J* = 8.5 Hz, 1H), 1.82 (s, 3H), 1.64 (s, 3H), 1.44 (s, 9H);

^{13}C-NMR ($CDCl_3$, 126 MHz), δ 191.76, 154.33, 80.80, 77.67, 51.41, 30.04, 28.19 (3C), 26.21.

ESI-HRMS: calcd. for $C_{10}H_{18}N_1O_3S$, $[M+H]^+$: 232.1002 (*m/z*); found $[M+H]^+$: 232.1013.

S4 (200.0 mg) was dissolved in cold TFA under -10 °C. After stirring for 10 min under the same

temperature, TFA was blown away by air flow. The residual TFA was removed by lyophilizer to yield 187.0 mg (S)-3-amino-4,4-dimethylthietan-2-one TFA salt (**7b**) (crude yield = 95%). The crude residual was used in the next step directly.

1.2.3 Synthesis of (3*S*,4*R*)-3-amino-4-isopropylthietan-2-one TFA salt (7c) and (3*S*,4*R*)-3-amino-4-phenylthietan-2-one TFA salt (7d)

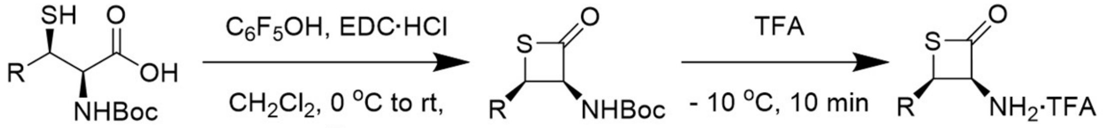

S5 R = *i*Pr
S7 R = Ph

S6 R = *i*Pr
S8 R = Ph

7c R = *i*Pr
7d R = Ph

To a solution of (2*R*,3*R*)-2-((tert-butoxycarbonyl)amino)-3-mercapto-4-methylpentanoic acid (**S5**)[2] (2.0 mmol) or (2*R*,3*R*)-2-((tert-butoxycarbonyl)amino)-3-mercapto-3- phenylpropanoic acid (**S7**)[3] (2.0 mmol) and Pentafluorophenol (1.1 equiv.) in dichloromethane (10 mL) at 0 °C, was added N-(3-Dimethylaminopropyl)-N-ethylcarbodiimide hydrochloride (EDC•HCl) (3.5 equiv.). The resulting mixture was stirred at 0 °C for 30 min, and the reaction was allowed to rise to room temperature for another 1h. The reaction mixture was quenched with saturated NH_4Cl solvent and extracted twice with dichloromethane. The combined organic layers were washed with brine, dried with anhydrous Na_2SO_4, filtered, and concentrated in vacuo. The residue was purified by silica gel chromatography to yield the desired β-Thiolactone (**S6** or **S8**).

tert-butyl ((2*S*,3*S*)-2-isopropyl-4-oxothietan-3-yl) carbamate (**S6, yield = 78 %**),
^{1}H-NMR ($CDCl_3$, 500 MHz), δ 5.85 – 5.82 (dd, *J* = 7.3, 9.4 Hz, 1H), 5.29 – 5.27 (d, *J* = 9.4 Hz), 3.69 – 3.66 (dd, *J* = 7.4, 8.7 Hz, 1H), 1.99 – 1.93 (m, 1H), 1.46 (s, 9H), 1.09 – 1.08 (d, *J* = 6.5 Hz, 3H), 1.07 – 1.05 (d, *J* = 6.5 Hz, 3H);
^{13}C-NMR ($CDCl_3$, 126 MHz), δ 193.94, 154.01, 81.03, 71.74, 51.37, 30.38, 28.20 (3C), 21.66, 21.24.
ESI-HRMS: calcd. for $C_{14}H_{18}NO_3S$, $[M+H]^+$: 246.1158 (*m/z*); found $[M+H]^+$: 246.1154.

tert-butyl ((3*S*,4*S*)-2-oxo-4-phenylthietan-3-yl) carbamate (**S8, yield = 53 %**),
^{1}H-NMR ($CDCl_3$, 400 MHz), δ 7.53 – 7.51 (d, *J* = 7.0 Hz, 2H), 7.42 – 7.39 (m, 2H), 7.35 – 7.33 (m, 1H), 5.26 (s, 1H), 5.09 (s, 1H), 5.04 (s, 1H), 1.46 (s, 9H);
^{13}C-NMR ($CDCl_3$, 126 MHz), δ 191.66, 154.30, 137.69, 129.01(2C), 128.56, 127.43(2C), 81.40, 79.01, 47.97, 28.20.
ESI-HRMS: calcd. for $C_{11}H_{20}NO_3S$, $[M+H]^+$: 280.1002 (*m/z*); found $[M+H]^+$: 280.1010.

β-Thiolactone (**S6** or **S8**) was dissolved in cold TFA under -10 °C. After stringing for 10 min under the same temperature, TFA was blown away by air flow. The residual TFA was removed by lyophilizer to afford the β-Thiolactone TFA salt (**7c** (crude yield = 96%) or **7d** (crude yield = 91%)). The crude residual was used in the next step directly.

1.3 General Procedures for Peptide Synthesis

1.3.1 Automated solid-phase peptide synthesis

Automated Solid-Phase peptide synthesis (SPPS) was performed on a Pioneer peptide synthesis system (GEN600611).

The Fmoc-Xxx-NovaSyn® TGT resin was employed in SPPS. Peptides were synthesized under standard automated Fmoc protocols using DMF as solvent, deblocking for 5 min in piperidine/ DBU/ DMF (2: 2: 96, V/ V/ V), coupling with HATU as coupling reagent for 25 min ('standard cycle'), or 55 min ('extended cycle') for amino acids such as prolines, valines, threonines, isoleucines and arginines.

The following $^{\alpha}N$-Fmoc or $^{\alpha}N$-Boc-protected amino acids from Novabiochem or Chem-impex were employed in SPPS: Fmoc-Ala-OH, Fmoc-Arg(Pbf)-OH, Fmoc-Asn(Trt)-OH, Fmoc-Asp(O^{t}Bu)-OH, Fmoc-Glu(O^{t}Bu)-OH, Fmoc-Gln(Trt)-OH, Fmoc-Gly-OH, Fmoc-His(Trt)-OH, Fmoc-Ile-OH, Fmoc-Leu-OH, Fmoc-Lys(NHBoc)-OH, Fmoc-Met-OH, Fmoc-Phe-OH, Fmoc-Pro-OH, Fmoc-Ser(^{t}Bu)-OH, Fmoc-Thr(^{t}Bu)-OH, Fmoc-Trp(Boc)-OH, Fmoc-Tyr-OH, Fmoc-Val-OH, Boc-Glu(O^{t}Bu)-OH, Boc-Cys(S^{t}Bu)-OH, Boc-Cys(Trt)-OH, Boc-Ser(O^{t}Bu)-OH.

1.3.2. Preparation of regular polypeptides

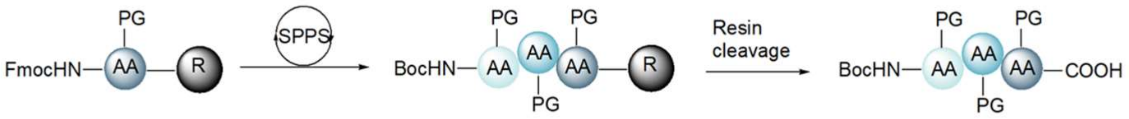

12

AA = amino acid, R = TGT resin, PG = protecting group

Supplementary Figure 1. Preparation of linear peptides via SPPS with TGT resin

Upon completion of the automated synthesis on a 0.04 mmol scale, the peptide resin was washed into a peptide synthesis vessel by using dichloromethane. The resin was cleaved by CH_2Cl_2/ TFE/ AcOH (8: 1: 1, V/ V/ V) solution for 20 min (× 3). After washing and filtration, the combined cleavage solution was concentrated under reduced pressure. The remaining residue was dissolved in a mixture of acetonitrile and water (V/ V = 1/ 1), which was lyophilized to remove the solvent. Without further purification, the lyophilized solid crude tripeptide **12** was directly used in the next coupling step.

1.3.3 Preparation of β-thiolactone bearing linear tetrapeptides

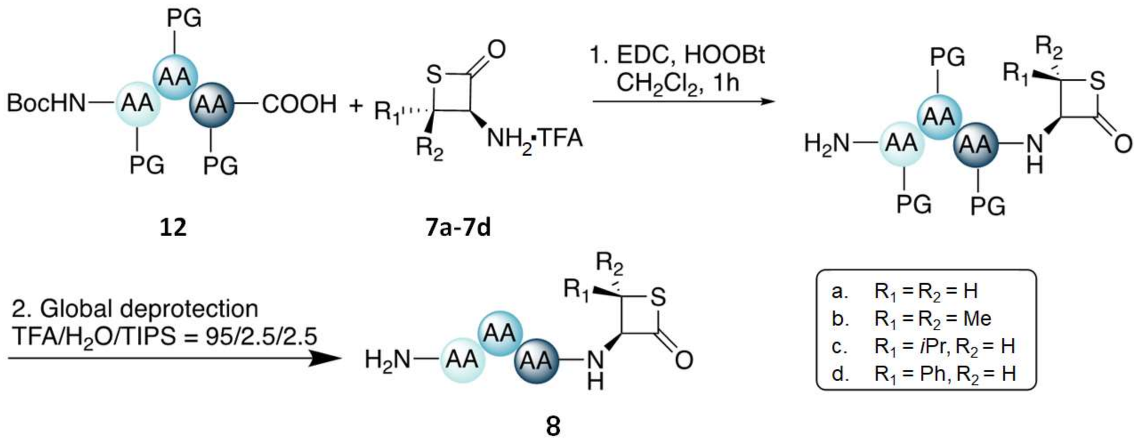

Supplementary Figure 2. Preparation of β-thiolactone bearing linear tetrapeptides

Sidechain fully protected tripeptide was prepared according to general procedure **A** and **B**. To the mixture of **12** (1.0 equiv.), the β-thiolactone TFA salt **7a-7d** (see section II) (1.2 equiv.) and 3-hydroxy-1,2,3-benzotriazin-4-one (HOOBt) (1.2 equiv.) were added anhydrous CH_2Cl_2. The resulting solution was stirred at 0 °C for 2 min, and N-(3-Dimethylaminopropyl)-N-ethylcarbodiimide (EDC) (1.2 equiv.) was added. The resulting mixture was stirred at 0 °C for another 1 h. The reaction was quenched with saturated NH_4Cl and extracted with CH_2Cl_2 (5 mL × 3), the organic layer was dried over anhydrous Na_2SO_4. The Na_2SO_4 was filtered and the solvent was removed via vacuum to afford a crude oily residue which was subjected to appropriate cocktail deprotection conditions at room temperature. After acid deprotection, the resulting solution was gently blown off by an argon stream to afford oily residue again. Finally, the oily residue was washed with cold diethyl ether to yield a white solid, which was dissolved in a mixture of acetonitrile and water and ready for HPLC purification after filtration. The desired β-thiolactone bearing tetrapeptide **8** was purified through preparation HPLC and generated as a white powder after lyophilization.

1.3.4 Cyclization of *C*-terminal β-thiolactone free tetrapeptides

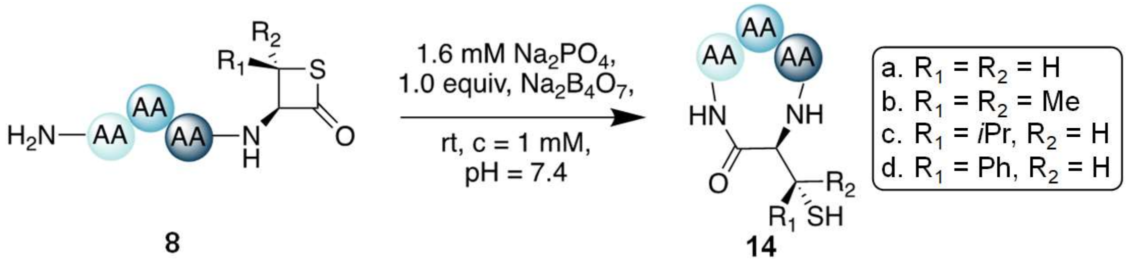

Supplementary Figure 3. Cyclization of C-terminal β-thiolactone

2 mL of 1.6 mM Na_2HPO_4 with $Na_2B_4O_7$ (1.0 equiv., 0.002 mmol) stock solution was prepared (pH = 7.4). To this stock solution, β-thiolactone bearing linear tetrapeptides (0.002 mmol) **8** was added (final reaction concentration 1.0 mM), and the mixture was stirred at room temperature for 4 to 8 hours under argon atmosphere in the plastic reaction tube. The reaction was analyzed by LC-MS until the starting material was totally consumed. The solvent was removed by lyophilization and the resulting white residue was dissolved in a mixture of acetonitrile and water prior to HPLC purification.

Preparation of reaction buffer, 1.6 mM Na_2HPO_4 stock solution was prepared by dissolving 2.27 mg of

Na_2HPO_4 in 10 mL degassed water, and to 2 mL of 1.6 mM Na_2PO_4 stock solution was added $Na_2B_4O_7$ (0.4 mg, 0.002 mmol, 1.0 equiv.). The resulting solution was used as the reaction solvent after $Na_2B_4O_7$ was completely dissolved (the final buffer pH = 7.4).

1.3.5 One-pot Ligation and Desulfurization

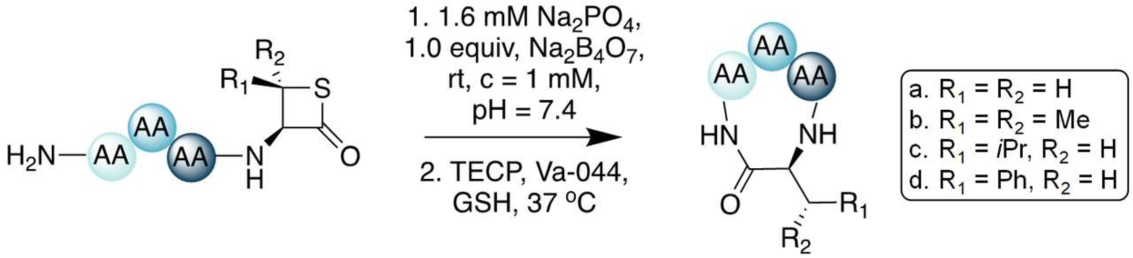

8

15

Supplementary Figure 4. One-pot ligation and desulfurization

Upon completion of the cyclization as indicated by LC-MS analysis, to the reaction vessel was added 350 μL of 0.5 M Tris(2-carboxyethyl)phosphine Hydrochloride (TCEP), L-Glutathione reduced (GSH) solution and 150 μL of radical initiator 2,2'-azobis[2-(2-imidazolin-2-yl) propane] dihydrochloride (VA-044) (0.1 M in degassed water). The reaction mixture was stirred at 37 °C until the completion with LC-MS monitoring, it was then quenched by adding 50 μL H_2O/ MeCN/ AcOH (90: 5: 5, *v/ v/ v*) and purified by HPLC.

Preparation of 0.5 M TCEP-GSH solution, 57.3 mg of TCEP and 6 mg GSH were added to 350 μl of cold, degassed, molecular biology grade water. Adjust the solution to pH 7.0 with 10 N NaOH and calibrate the total solution volume to 400 μL with water. This stock solution was stored at -20 ^oC and used within one week.

Preparation of 0.1 M VA-044, 32 mg of VA-044 was dissolved in 1 mL of cold, degassed water. The resulting solution was used immediately.

1.3.6 Cyclization of sidechain protected tetrapeptides by HATU coupling.

According to general procedure B, the starting material of HATU cyclization was generated from SPPS, the N-terminus Fmoc protecting group was removed on resin. After the peptide was cleaved by CH_2Cl_2/TFE/AcOH (8: 1: 1, V/V/V), the resulting crude residue was dissolved in acetonitrile/H_2O (1: 1, V/V) and lyophilized to generate light yellow fluffy solid. Then the crude residue was dissolved in DMF (c = 0.5 mM). Then the solution was subsequently added HATU (4.0 equiv.) and DIPEA (4.0 equiv.). After the reaction mixture stirring at room temperature for 6 to 8h, the solvent evaporated out. Then the residue was dissolved in 3 mL TFA/TIPSH/H_2O (95/2.5/2.5, V/V/V) solution and stirred at room temperature for 10 to 20 min. After the global deprotection, the resulting solution was gently blown off by an argon stream to afford oily residue. Finally, the oily residue was washed with cold diethyl ether and was dissolved in a mixture of acetonitrile and water for HPLC purification after filtration.

1.4 Preparation and Characterization of Peptide Segments

1.4.1 preparation of β-thiolactone bearing linear tetrapeptides

Peptidyl Pen-β-thiolactone S9a

–

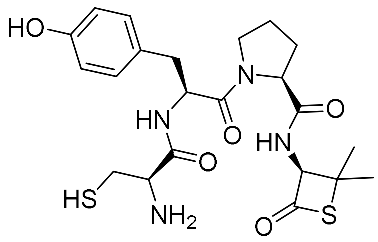

S9

According to the general procedure A and B, the side chain fully protected tripeptide Boc-Cys(Trt)-Tyr(O*t*Bu)-Pro-OH was generated from SPPS on a 0.04 mmol scale. The resulting tripeptide Boc-Cys(Trt)-Tyr(O*t*Bu)-Pro-OH was coupled with β-thiolactone **14b** to afford the desired tetrapeptide following the general procedure C. Purification of the crude product using preparative HPLC (10 to 60% solvent B over 20 min, Higgins Analytical Proto 200 5 μm 250 × 10 nm C18 column) afforded peptide **8** as a white solid after lyophilization (14.6 mg, 74%).

Analytical HPLC: t_R = 12.63 min (20 to 60% solvent B over 20 min, Higgins Analytical Proto 200 5 μm 150 × 2.0 nm C18 column).
ESI-LRMS: calcd. for $C_{22}H_{31}N_4O_5S_2$, $[M+H]^+$: 495.1730 (*m/z*); found $[M+H]^+$: 495.1729.

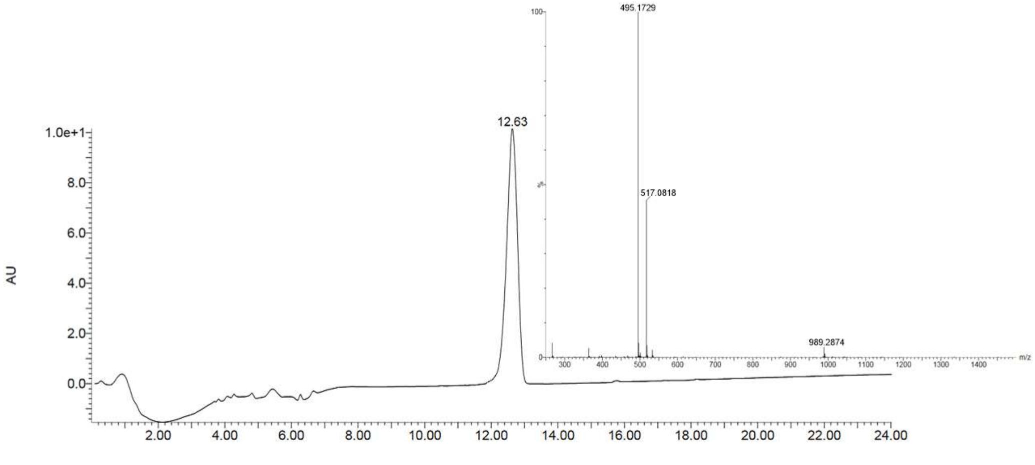

Peptidyl Phe-β-thiolactone 8a

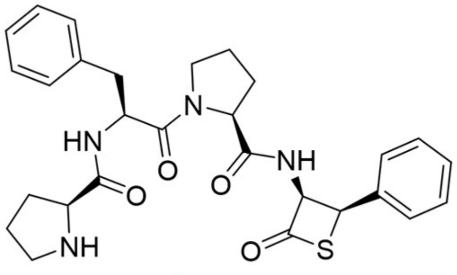

8a

Chemical Formula: $C_{28}H_{32}N_4O_4S$
Exact Mass: 520.2144

According to the general procedure A and B, the side chain fully protected tripeptide Boc-Pro-Phe-Pro-OH was generated from SPPS on a 0.04 mmol scale. The resulting Boc-Pro-Phe-Pro-OH coupled with β-thiolactone **7d** to afford the desired tetrapeptide following the general procedure C. Purification of the crude product using preparative HPLC (20 to 50% solvent B over 20 min, Higgins Analytical Proto 200 5 μm 250 × 10 nm C18 column) afforded peptide **8a** as a white solid after lyophilization (18.5 mg, 89%).

Analytical HPLC: t_R = 19.30 min (20 to 60% solvent B over 20 min, Higgins Analytical Proto 200 5 μm 150 × 2.0 nm C18 column).
^{1}H NMR (600 MHz, DMSO-*d6*) δ 8.88 – 8.87 (d, *J* = 8.0 Hz, 1H), 8.84 – 8.82 (d, *J* = 7.8 Hz, 1H), 7.59 – 7.57 (m, 2H), 7.40 – 7.38 (m, 2H), 7.35 – 7.32 (m, 1H), 7.26 – 7.23 (m, 4H), 7.20 – 7.17 (m, 1H), 5.42 – 5.40 (dd, *J* = 7.8, 5.2 Hz, 1H), 5.01 – 5.00 (d, *J* = 5.2 Hz, 1H), 4.69 – 4.66 (ddd, *J* = 9.9, 8.2, 4.1 Hz, 1H), 4.29 – 4.26 (dd, *J* = 8.6, 4.6 Hz, 1H), 4.08 (s, 1H), 3.65 – 3.63 (m, 1H), 3.60 – 3.58 (m, 1H), 3.18 – 3.12 (m, 2H), 3.07 – 3.04 (dd, *J* = 14.2, 4.0 Hz, 1H), 2.75 – 2.71 (dd, *J* = 14.2, 9.9 Hz, 1H), 2.30 – 2.25 (m, 1H), 2.13 – 2.08 (m, 1H), 2.00 – 1.96 (m, 1H), 1.92 – 1.89 (m, 1H), 1.80 – 1.76 (m, 4H).
^{13}C NMR (151 MHz, DMSO-*d6*) δ 191.17, 172.54, 169.73, 168.44, 138.45, 137.71, 129.69, 129.61, 129.17, 129.15, 129.11, 128.72, 128.68, 128.16, 128.12, 126.95, 77.80, 60.08, 59.11, 53.20, 47.29, 46.43, 46.12, 36.67, 29.91, 29.69, 25.07, 23.87.
ESI-LRMS: calcd. for $C_{28}H_{33}N_4O_4S$, $[M+H]^+$: 521.2217 (*m/z*); found $[M+H]^+$: 521.2295.

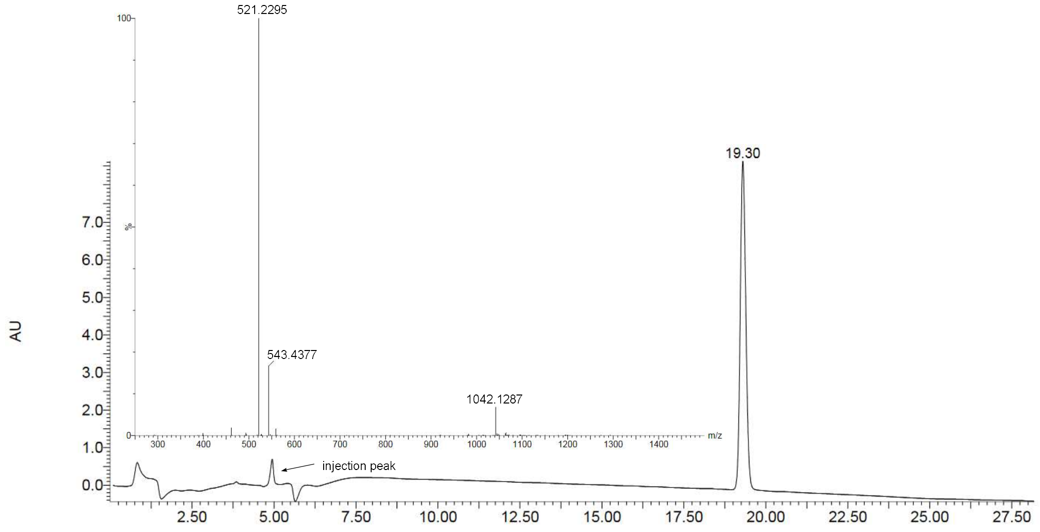

Peptidyl Pen-β-thiolactone 5

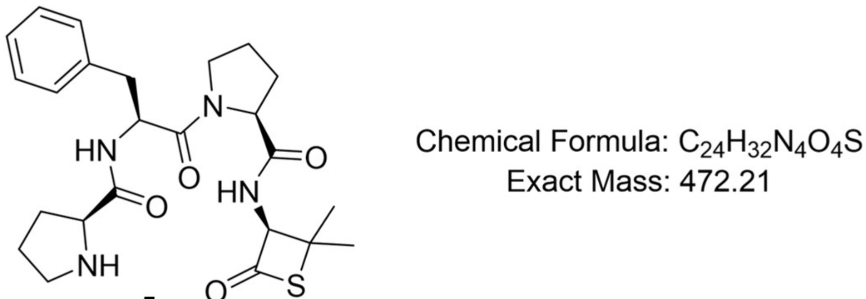

5

According to the general procedure A and B, the side chain fully protected tripeptide Boc-Pro-Phe-Pro-OH was generated from SPPS on a 0.04 mmol scale. The resulting tripeptide Boc-Pro-Phe-Pro-OH was coupled with β-thiolactone **7b** to afford the desired tetrapeptide following the general procedure C. Purification of the crude product using preparative HPLC (20 to 60% solvent B over 20 min, Higgins Analytical Proto 200 5 µm 250 × 10 nm C18 column) afforded peptide **5** as a white solid after lyophilization (12.6 mg, 67%).

Analytical HPLC: t_R = 15.43 min (20 to 60% solvent B over 20 min, Higgins Analytical Proto 200 5 µm 150 × 2.0 nm C18 column).
^{1}H NMR (600 MHz, DMSO-*d6*) δ 9.08 (d, J = 8.5 Hz, 1H), 8.85 (d, J = 8.0 Hz, 1H), 7.28 – 7.22 (m, 4H), 7.19 – 7.18 (m, 1H), 5.61 (d, J = 8.5 Hz, 1H), 4.74 – 4.70 (td, J = 8.3, 4.8 Hz, 1H), 4.30 – 4.28 (dd, J = 8.4, 4.9 Hz, 1H), 4.15 – 4.10 (m, 1H), 3.65 – 3.62 (m, 1H), 3.60 – 3.56 (m, 1H), 3.54 – 3.52 (dd, J = 11.1, 7.3 Hz, 1H), 3.40 – 3.36 (dd, J = 15.9, 8.3 Hz, 2H), 3.32 – 3.30 (d, J = 8.1 Hz, 1H), 3.08 – 3.05 (dd, J = 14.1, 4.6 Hz, 1H), 2.97 – 2.94 (dd, J = 11.2, 9.3 Hz, 1H), 2.79 – 2.70 (m, 2H), 2.13 – 2.08 (m, 1H), 1.98 – 1.93 (m, 1H), 1.90 – 1.85 (m, 1H), 1.77 – 1.74 (m, 1H), 1.73 (s, 3H), 1.68 – 1.62 (m, 2H), 1.60 (s, 3H).
^{13}C NMR (151 MHz, DMSO-*d6*) δ 191.95, 171.73, 169.12, 167.50, 137.41, 129.89 (2C), 128.64 (2C), 126.98, 77.02, 59.86, 59.12, 53.69, 53.11, 51.93, 47.36, 40.44, 36.73, 34.70, 30.45, 29.78, 26.31, 25.17.
ESI-LRMS: calcd. for $C_{24}H_{33}N_4O_4S$, $[M+H]^+$: 473.2217 (*m/z*); found $[M+H]^+$: 473.2353.

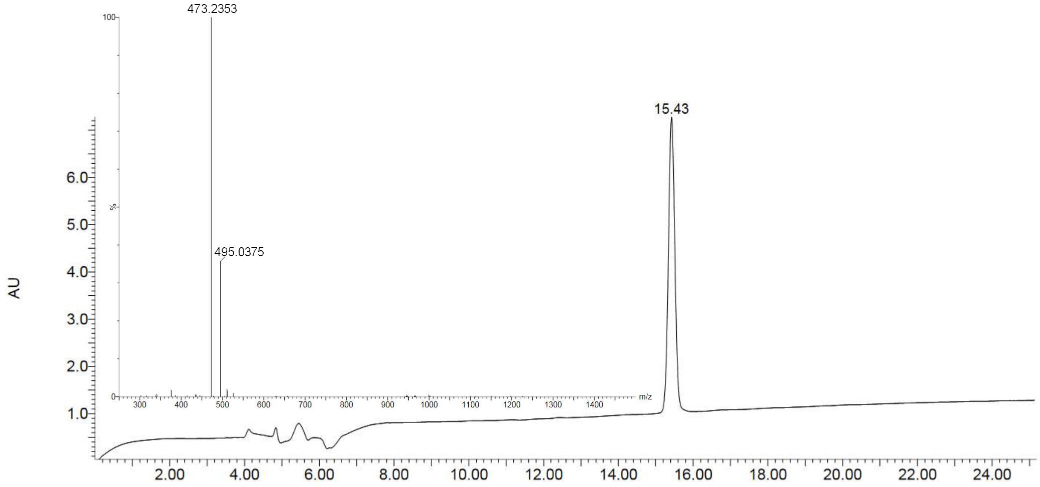

Peptidyl Pen-β-thiolactone 8c

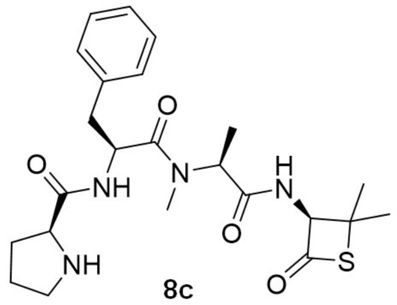

8c

Chemical Formula: $C_{23}H_{32}N_4O_4S$
Exact Mass: 460.21

According to the general procedure A and B, the side chain fully protected tripeptide Boc-Pro-Phe-Ala(NMe)-OH was generated from SPPS on a 0.04 mmol scale. The resulting tripeptide Boc-Pro-Phe-Ala(NMe)-OH was coupled with β-thiolactone **7b** to afford the desired tetrapeptide following the general procedure C. Purification of the crude product using preparative HPLC (20 to 60% solvent B over 20 min, Higgins Analytical Proto 200 5 μm 250 × 10 nm C18 column) afforded peptide **8c** as a white solid after lyophilization (12.3 mg, 67%).

Analytical HPLC: t_R = 11.10 min (30 to 70% solvent B over 20 min, Higgins Analytical Proto 200 5 μm 150 × 2.0 nm C18 column).

^{1}H NMR (600 MHz, DMSO-*d6*) δ 8.92 – 8.90 (d, *J* = 8.7 Hz, 1H), 8.86 – 8.85 (d, *J* = 8.1 Hz, 1H), 7.28 – 7.24 (m, 5H), 5.62 – 5.61 (d, *J* = 8.6 Hz, 1H), 4.91 – 4.88 (td, *J* = 8.7, 4.7 Hz, 1H), 4.81 – 4.78 (q, *J* = 7.2 Hz, 1H), 4.12 – 4.10 (t, *J* = 7.1 Hz, 1H), 3.19 – 3.17 (m, 1H), 3.05 – 3.01 (m, 1H), 2.96 (s, 3H), 2.82 – 2.78 (m, 1H), 2.30 – 2.24 (m, 1H), 1.88 – 1.81 (m, 2H), 1.80 – 1.74 (m, 2H), 1.71 (s, 3H), 1.55 (s, 3H), 1.25 – 1.23 (d, *J* = 7.2 Hz, 3H).

^{13}C NMR (151 MHz, DMSO-*d6*) δ 192.01, 171.03, 171.01, 168.19, 137.53, 129.72 (2C), 128.70 (2C), 127.02, 76.70, 59.15, 53.53, 51.81, 51.69, 46.15, 37.06, 32.19, 30.51, 30.01, 26.09, 25.89, 14.69.

ESI-LRMS: calcd. for $C_{23}H_{33}N_4O_4S$, $[M+H]^+$: 461.2217 (*m/z*); found $[M+H]^+$: 461.2174.

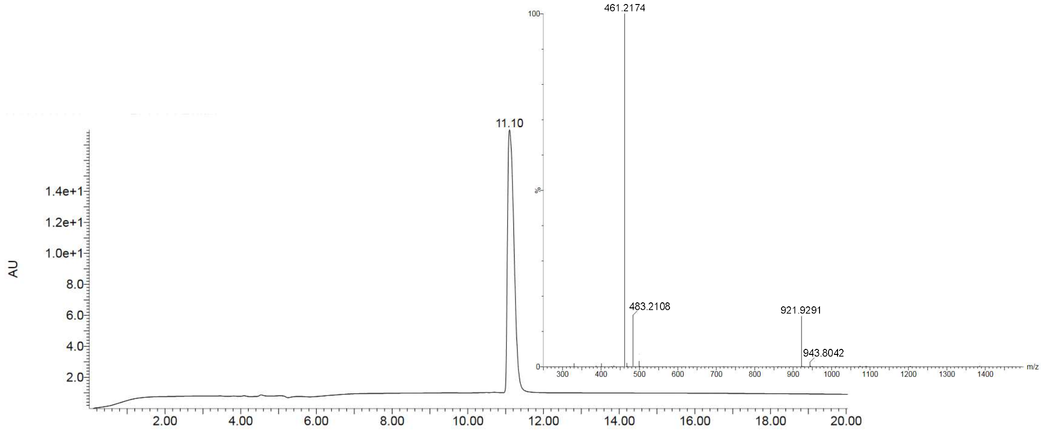

Peptidyl Pen-β-thiolactone 8d

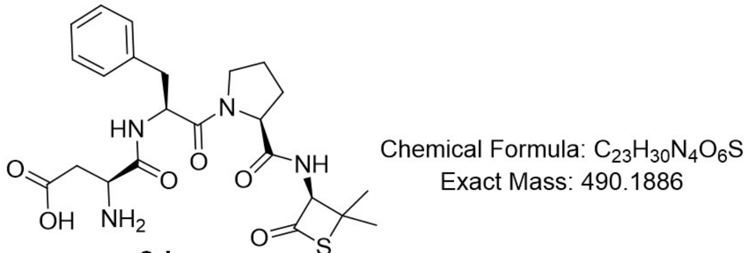

8d

According to the general procedure A and B, the side chain fully protected tripeptide Boc-Asp(O^{t}Bu)-Phe-Pro-OH was generated from SPPS on a 0.04 mmol scale. The resulting tripeptide Boc-Asp(O^{t}Bu)-Phe-Pro-OH was coupled with β-thiolactone **7b** to afford the desired tetrapeptide following the general procedure C. Purification of the crude product using preparative HPLC (20 to 60% solvent B over 20 min, Higgins Analytical Proto 200 5 µm 250 × 10 nm C18 column) afforded peptide **8d** as a white solid after lyophilization (11.2 mg, 57%).

Analytical HPLC: t_R = 10.54 min (30 to 60% solvent B over 20 min, Higgins Analytical Proto 200 5 µm 150 × 2.0 nm C18 column).

^{1}H NMR (600 MHz, DMSO-*d6*) δ 9.04 (d, *J* = 8.5 Hz, 1H), 8.70 (d, *J* = 7.7 Hz, 1H), 7.25 (d, *J* = 2.4 Hz, 5H), 7.19 (d, *J* = 5.9 Hz, 2H), 5.57 (d, *J* = 8.5 Hz, 1H), 4.67 (d, *J* = 4.9 Hz, 1H), 4.27 (dd, *J* = 8.0, 5.0 Hz, 1H), 3.94 – 3.89 (m, 1H), 3.06 (dd, *J* = 14.0, 4.5 Hz, 1H), 2.77 – 2.66 (m, 3H), 2.54 (dd, *J* = 17.2, 8.8 Hz, 2H), 2.10 (dd, *J* = 12.3, 7.7 Hz, 1H), 1.94 (dd, *J* = 12.9, 6.4 Hz, 1H), 1.91 – 1.83 (m, 1H), 1.78 – 1.73 (m, 1H), 1.72 (s, 3H), 1.59 (s, 3H).

^{13}C NMR (151 MHz, DMSO-*d6*) δ 191.97, 171.89, 171.67, 169.34, 168.60, 137.46, 129.81 (2C), 128.67 (2C), 126.98, 76.95, 59.90, 52.98, 51.92, 49.50, 47.31, 36.69, 36.20, 30.42, 29.74, 26.23, 25.10.

ESI-LRMS: calcd. for $C_{23}H_{31}N_4O_6S$, $[M+H]^+$: 491.1959 (*m/z*); found $[M+H]^+$: 491.1852.

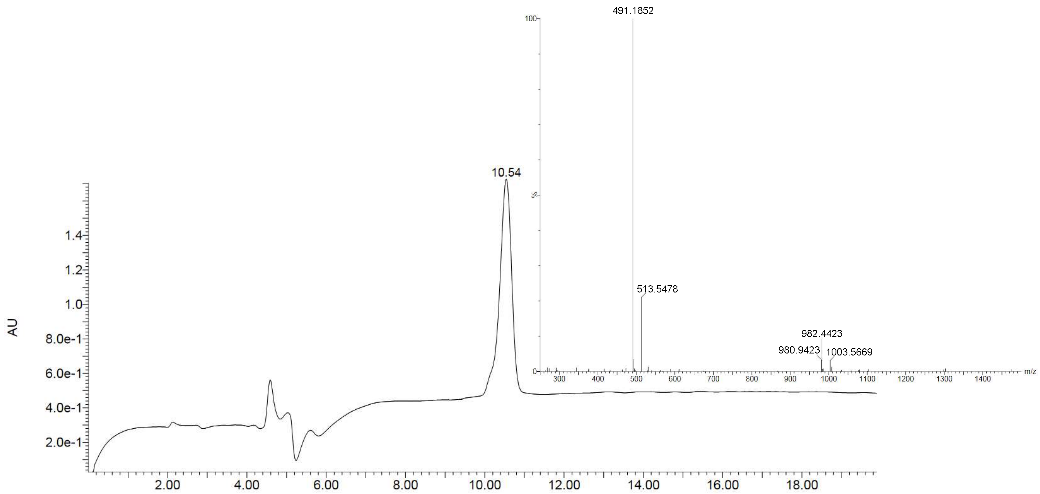

Peptidyl Pen-β-thiolactone 8e

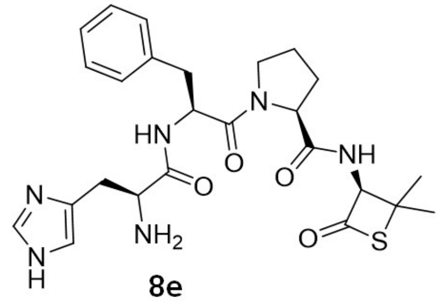

8e

Chemical Formula: $C_{25}H_{32}N_6O_4S$
Exact Mass: 512.22

According to the general procedure A and B, the side chain fully protected tripeptide Boc-His-Phe-Pro-OH was generated from SPPS method on a 0.04 mmol scale. The resulting tripeptide Boc-His-Phe-Pro-OH was coupled with β-thiolactone **7b** to afford the desired tetrapeptide following the general procedure C. Purification of the crude product using preparative HPLC (20 to 30% solvent B over 20 min, Higgins Analytical Proto 200 5 µm 250 × 10 nm C18 column) afforded peptide **8e** as a white solid after lyophilization (16.6 mg, 81%).

Analytical HPLC: t_R = 12.58 min (20 to 60% solvent B over 20 min, Higgins Analytical Proto 200 C18 5 µm 150 × 2.0 nm column);
^{1}H NMR (600 MHz, DMSO-*d6*) δ 9.06 (d, *J* = 8.5 Hz, 1H), 8.82 (d, *J* = 7.1 Hz, 1H), 7.36 (s, 1H), 7.33 – 7.23 (m, 4H), 7.24 – 7.15 (m, 1H), 5.62 (dd, *J* = 8.6, 2.3 Hz, 1H), 4.69 (dd, *J* = 12.9, 7.5 Hz, 1H), 4.31 (dd, *J* = 8.3, 5.0 Hz, 1H), 4.16 (dd, *J* = 11.1, 4.9 Hz, 1H), 3.63 (dd, *J* = 11.7, 4.7 Hz, 1H), 3.48 (dt, *J* = 9.3, 7.0 Hz, 2H), 3.16 (d, *J* = 6.1 Hz, 2H), 3.08 (dd, *J* = 14.2, 5.2 Hz, 1H), 2.84 (s, 1H), 2.81 (dd, *J* = 16.5, 8.7 Hz, 1H), 2.16 – 2.07 (m, 1H), 1.99 – 1.92 (m, 1H), 1.90 – 1.83 (m, 2H), 1.80 – 1.75 (m, 1H), 1.74 (s, 3H), 1.68 (dd, *J* = 10.6, 8.1 Hz, 1H), 1.60 (d, *J* = 5.8 Hz, 3H).
^{13}C NMR (151 MHz, DMSO-*d6*) δ 191.94, 171.64, 169.73, 167.77, 137.19, 134.93, 129.89 (2C), 128.68 (2C), 127.29, 127.02, 118.53, 77.03, 60.04, 53.25, 51.95, 51.56, 47.41, 36.49, 30.47, 29.71, 27.04, 26.25, 25.16.
ESI-LRMS: calcd. for $C_{25}H_{33}N_6O_4S$, $[M+H]^+$: 513.2279 (*m/z*); found $[M+H]^+$: 513.2602.

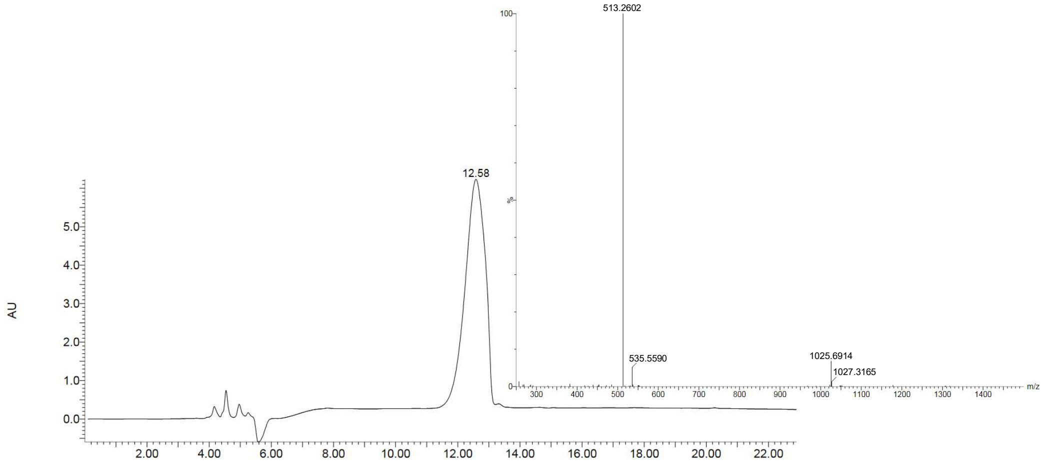

Peptidyl Pen-β-thiolactone 8f

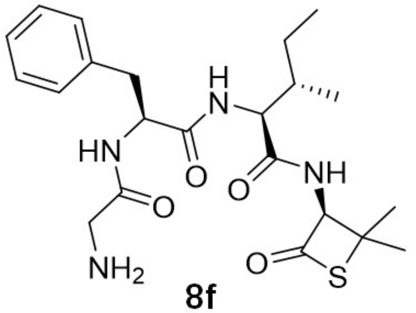

8f

Chemical Formula: $C_{22}H_{32}N_4O_4S$
Exact Mass: 448.2144

According to the general procedure A and B, the side chain fully protected tripeptide Boc-Gly-Phe-Ile-OH was generated from SPPS on a 0.04 mmol scale. The resulting tripeptide Boc-Gly-Phe-Ile-OH was coupled with β-thiolactone **7b** to afford the desired tetrapeptide following the general procedure C. Purification of the crude product using preparative HPLC (20 to 60% solvent B over 20 min, Higgins Analytical Proto 200 5 µm 250 × 10 nm C18 column) afforded peptide **8f** as a white solid after lyophilization (15.9 mg, 89%).

Analytical HPLC: t_R = 17.13 min (20 to 60% solvent B over 20 min, Higgins Analytical Proto 200 5 µm 150 × 2.0 nm C18 column).

^{1} H NMR (600 MHz, DMSO-*d*6) δ 9.08 – 9.06 (d, *J* = 8.6 Hz, 1H), 8.55 – 8.54 (d, *J* = 8.2 Hz, 1H), 8.37 – 8.35 (d, *J* = 8.1 Hz, 1H), 7.92 (s, 2H), 7.24 – 7.21 (m, 4H), 7.18 – 7.15 (m, 1H), 5.65 – 5.64 (d, *J* = 8.7 Hz, 1H), 4.74 – 4.70 (td, *J* = 4.2, 8.8 Hz, 1H), 4.12 – 4.09 (t, *J* = 8.1 Hz, 1H), 3.54 – 3.51 (d, *J* = 16.2 Hz, 1H), 3.40 – 3.38 (d, *J* = 16.2 Hz, 1H), 3.00 – 2.97 (dd, *J* = 4.1, 13.9 Hz, 1H), 2.72 – 2.68 (dd, *J* = 9.3, 13.9 Hz, 1H), 1.71 (s, 3H), 1.68 – 1.67 (m, 1H), 1.57 (s, 3H), 1.53 – 1.44 (m, 1H), 1.12 – 1.07 (m, 1H), 0.85 – 0.81 (m, 6H).

^{13}C NMR (151 MHz, DMSO-*d*6) δ 191.71, 171.25, 171.05, 166.00, 137.74, 129.76 (2C), 128.46 (2C), 126.81, 76.75, 57.67, 53.98, 51.59, 38.25, 36.58, 30.39 (2C), 26.30, 25.02, 15.74, 11.39.

ESI-LRMS: calcd. for $C_{22}H_{33}N_4O_4S$, $[M+H]^+$: 449.2217 (*m/z*); found $[M+H]^+$: 449.2156.

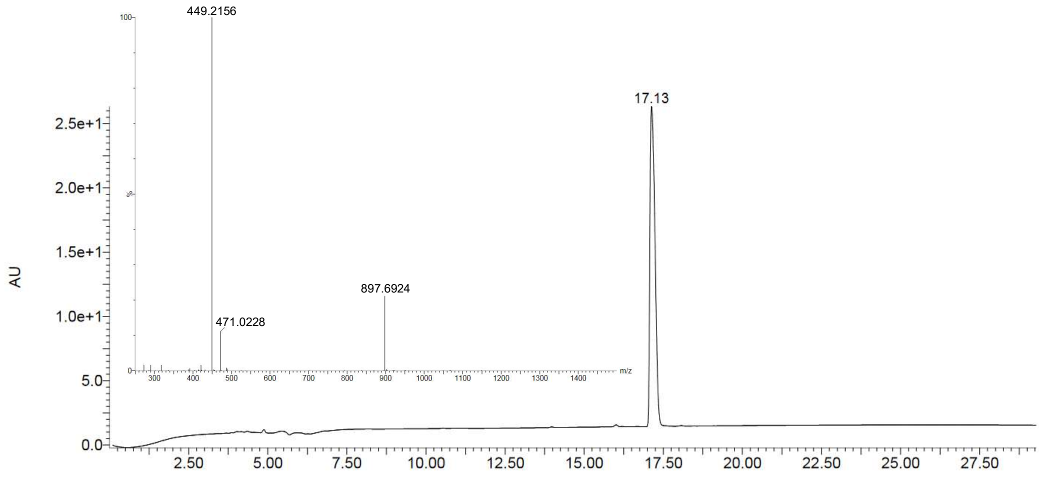

Peptidyl Pen-β-thiolactone 8g

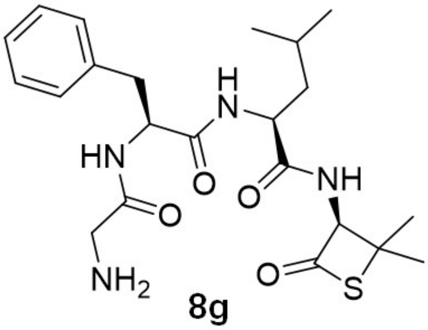

8g

Chemical Formula: $C_{22}H_{32}N_4O_4S$
Exact Mass: 448.2144

According to the general procedure A and B, the side chain fully protected tripeptide Boc-Gly-Phe-Leu-OH was generated from SPPS on a 0.04 mmol scale. The resulting tripeptide Boc-Gly-Phe-Leu-OH was coupled with β-thiolactone **7b** to afford the desired tetrapeptide following the general procedure C. Purification of the crude product using preparative HPLC (20 to 60% solvent B over 20 min, Higgins Analytical Proto 200 5 µm 250 × 10 nm C18 column) afforded peptide **8g** as a white solid after lyophilization (14.9 mg, 83%).

Analytical HPLC: t_R = 11.50 min (30 to 70% solvent B over 20 min, Higgins Analytical Proto 200 5 µm 150 × 2.0 nm C18 column).
^{1}H NMR (DMSO-*d*6, 600 MHz) δ 9.10 – 9.09 (d, *J* = 8.5Hz, 1H), 8.56 – 8.55 (d, *J* = 8.2 Hz, 1H), 8.44 – 8.43 (d, *J* = 7.3 Hz, 1H), 7.96 (s, 3H), 7.23 – 7.22 (m, 3H), 7.18 – 7.16 (m, 1H), 5.61 – 5.59 (d, *J* = 8.5 Hz, 1H), 4.66 – 4.63 (td, *J* = 3.9, 8.6 Hz, 1H), 4.28 – 4.24 (m, 1H), 3.54 – 3.51 (d, *J* = 16.2 Hz, 1H), 3.40 – 3.38 (d, *J* = 16.2 Hz, 1H), 3.04 – 3.01 (dd, *J* = 3.8, 13.9 Hz, 1 H), 2.73 – 2.69 (dd, *J* = 9.4, 13.8 Hz, 1H), 1.71 (s, 3H), 1.63 – 1.59 (m, 1H), 1.57 (s, 3H), 1.54 – 1.49 (m, 1H), 1.43 – 1.39 (m, 1H), 0.90 – 0.89 (d, *J* = 6.5Hz, 3H), 0.85 – 0.84 (d, *J* = 6.5 Hz, 3H).
^{13}C NMR (DMSO-*d*6, 151 MHz) δ 191.77, 172.33, 171.00, 166.03, 137.80, 129.78 (2C), 128.46 (2C), 126.79, 76.94, 54.06, 51.74, 51.68, 41.02, 38.21, 30.48, 26.14, 24.68, 23.39 (2C), 21.97.
ESI-LRMS: calcd. for $C_{22}H_{33}N_4O_4S$, $[M+H]^+$: 449.2217 (*m/z*); found $[M+H]^+$: 449.2080.

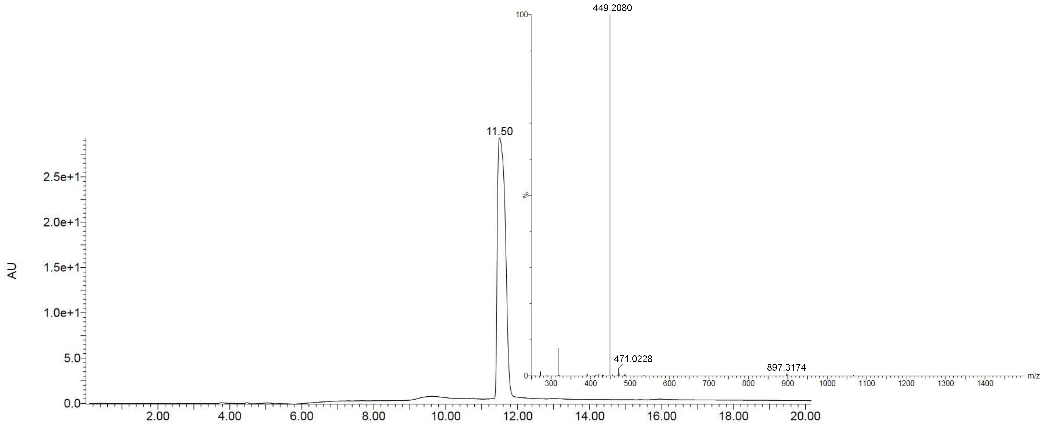

Peptidyl Pen-β-thiolactone 8h

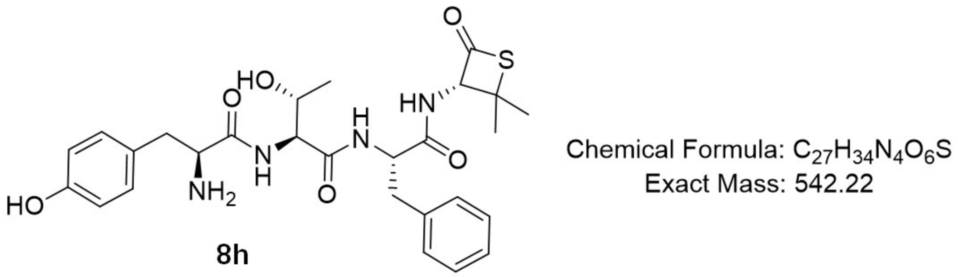

8h

According to the general procedure A and B, the side chain fully protected tripeptide Boc-Tyr(O*t*Bu)-Thr(O*t*Bu)-Phe-OH was generated from SPPS on a 0.04 mmol scale. The resulting tripeptide Boc-Tyr(O*t*Bu)-Thr(O*t*Bu)-Phe-OH was coupled with β-thiolactone **7b** to afford the desired tetrapeptide following the general procedure C. Purification of the crude product using preparative HPLC (20 to 60% solvent B over 20 min, Higgins Analytical Proto 200 5 μm 250 × 10 nm C18 column) afforded peptide **8h** as a white solid after lyophilization (14.3 mg, 66%).

Analytical HPLC: t_R = 10.83 min (30 to 70% solvent B over 20 min, Higgins Analytical Proto 200 5 μm 150 × 2.0 nm C18 column).
^{1}H NMR (500 MHz, DMSO-d_6) δ 9.17 (d, *J* = 8.7 Hz, 1H), 8.61 (d, *J* = 8.4 Hz, 1H), 8.14 (d, *J* = 7.8 Hz, 1H), 8.04 – 7.95 (m, 3H), 7.30 (d, *J* = 4.4 Hz, 1H), 7.26 (d, *J* = 4.3 Hz, 4H), 7.19 (p, *J* = 4.4 Hz, 1H), 7.04 (d, *J* = 8.0 Hz, 2H), 6.68 (d, *J* = 8.0 Hz, 2H), 5.64 (d, *J* = 8.6 Hz, 1H), 4.60 (td, *J* = 7.9, 5.0 Hz, 1H), 4.34 (dd, *J* = 8.4, 4.7 Hz, 1H), 4.08 (s, 1H), 3.97 (p, *J* = 6.2 Hz, 1H), 3.06 (dd, *J* = 14.0, 5.1 Hz, 1H), 2.96 (dd, *J* = 14.5, 4.6 Hz, 1H), 2.90 (dd, *J* = 13.9, 8.3 Hz, 1H), 2.74 (dd, *J* = 14.4, 8.5 Hz, 1H), 1.70 (s, 3H), 1.56 (s, 3H), 1.05 (d, *J* = 6.2 Hz, 3H).
^{13}C NMR (126 MHz, DMSO) δ 191.61, 170.92, 169.60, 168.77, 157.03, 137.65, 131.18, 131.00, 129.61, 128.73, 128.63, 126.90, 125.22, 115.79, 76.61, 67.26, 58.44, 54.22, 53.94, 51.67, 37.70, 36.77, 30.29, 26.27, 19.63.
ESI-LRMS: calcd. for $C_{27}H_{35}N_4O_6S$, $[M+H]^+$: 543.2277 (*m/z*); found $[M+H]^+$: 543.2338.

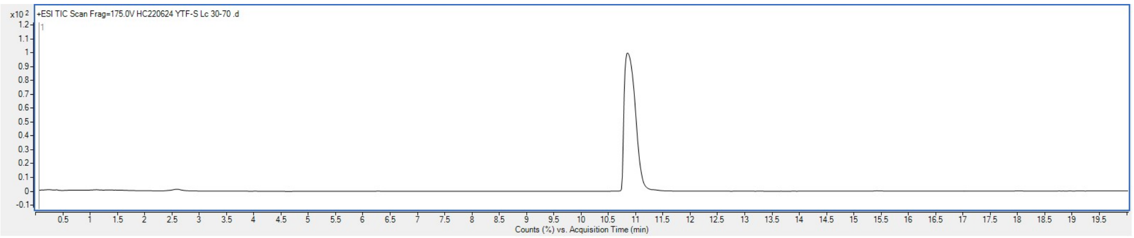

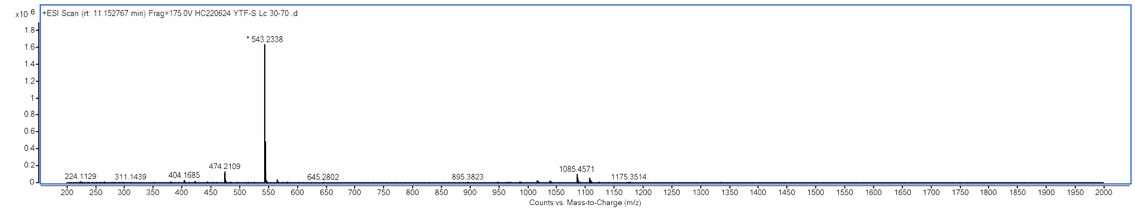

Peptidyl Pen-β-thiolactone S14a

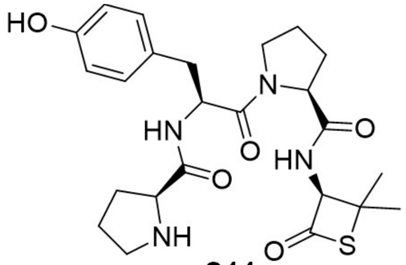

S14a

Chemical Formula: $C_{24}H_{32}N_4O_5S$
Exact Mass: 488.21

According to the general procedure A and B, the side chain fully protected tripeptide Boc-Pro-Tyr(O*t*Bu)-Pro-OH was generated from SPPS on a 0.04 mmol scale. The resulting tripeptide Boc-Pro-Tyr(O*t*Bu)-Pro-OH was coupled with β-thiolactone **7b** to afford the desired tetrapeptide following the general procedure C. Purification of the crude product using preparative HPLC (20 to 60% solvent B over 20 min, Higgins Analytical Proto 200 5 µm 250 × 10 nm C18 column) afforded peptide **S14a** as a white solid after lyophilization (15.4 mg, 79%).

Analytical HPLC: t_R = 12.43 min (10 to 60% solvent B over 20 min, Higgins Analytical Proto 200 5 µm 150 × 2.0 nm C18 column).
^{1}H NMR (600 MHz, DMSO-*d6*) δ 9.07 (d, *J* = 8.5 Hz, 1H), 8.78 (d, *J* = 8.0 Hz, 1H), 7.04 (d, *J* = 8.5 Hz, 2H), 6.62 (d, *J* = 8.5 Hz, 2H), 5.61 (d, *J* = 8.5 Hz, 1H), 4.68 – 4.61 (m, 1H), 4.28 (dd, *J* = 8.4, 4.9 Hz, 1H), 4.14 (t, *J* = 8.7 Hz, 1H), 3.61 (dd, *J* = 11.7, 4.7 Hz, 1H), 3.58 – 3.50 (m, 3H), 3.39 (dd, *J* = 15.3, 7.7 Hz, 2H), 3.31 (d, *J* = 8.1 Hz, 1H), 3.01 – 2.90 (m, 2H), 2.80 – 2.72 (m, 1H), 2.65 (dd, *J* = 14.2, 8.5 Hz, 1H), 2.10 (dq, *J* = 12.5, 7.9 Hz, 1H), 1.95 (dt, *J* = 21.2, 7.1 Hz, 1H), 1.92 – 1.81 (m, 1H), 1.76 (dd, *J* = 12.0, 6.0 Hz, 1H), 1.73 (s, 3H), 1.67 – 1.62 (m, 1H), 1.60 (s, 3H).
^{13}C NMR (151 MHz, DMSO-*d6*) δ 191.96, 171.75, 169.30, 167.41, 156.43, 130.80 (2C), 127.37, 115.43 (2C), 77.01, 59.81, 59.13, 53.69, 53.44, 51.94, 47.33, 40.42, 36.01, 34.70, 30.45, 29.76, 26.31, 25.16.
ESI-LRMS: calcd. for $C_{24}H_{33}N_4O_5S$, $[M+H]^+$: 489.2166 (*m/z*); found $[M+H]^+$: 489.2116.

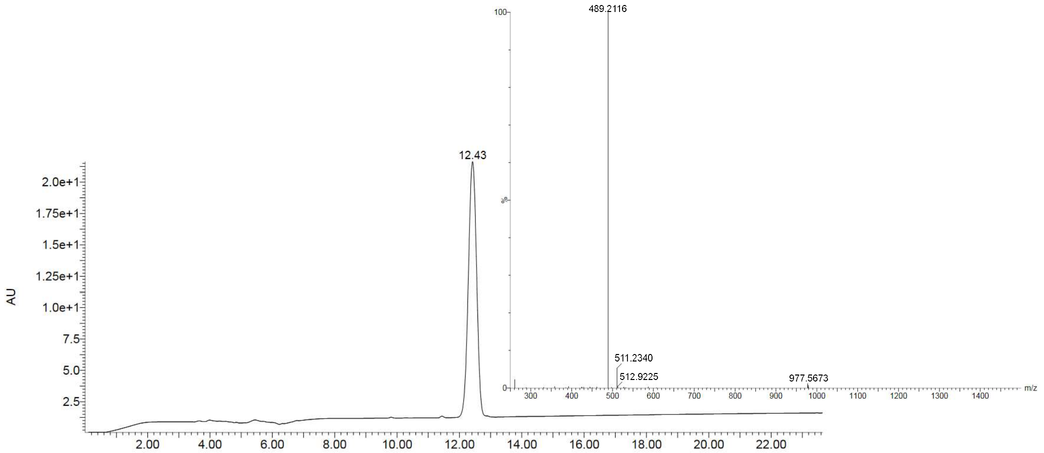

Peptidyl Leu-β-thiolactone S2a

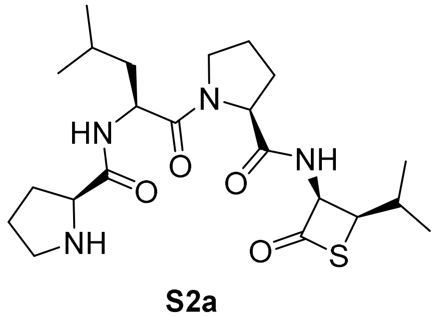

Chemical Formula: $C_{22}H_{36}N_4O_4S$
Exact Mass: 452.2457

S2a

According to the general procedure A and B, the side chain fully protected tripeptide Boc-Pro-Leu-Pro-OH was generated from SPPS on a 0.04 mmol scale. The resulting Boc-Pro-Leu-Pro-OH was coupled with β-thiolactone **7c** to afford the desired tetrapeptide following the general procedure C. Purification of the crude product using preparative HPLC (30 to 70% solvent B over 20 min, Higgins Analytical Proto 200 5 µm 250 × 10 nm C18 column) afforded peptide **S2a** as a white solid after lyophilization (15.6 mg, 86%).

Analytical HPLC: t_R = 11.42 min (30 to 60% solvent B over 20 min, Higgins Analytical Proto 200 5 µm 150 × 2.0 nm C18 column).
^{1}H NMR (600 MHz, DMSO-*d*6) δ 9.36 (s, 1H), 8.94 (d, *J* = 9.2 Hz, 1H), 8.73 (d, *J* = 7.9 Hz, 1H), 5.91 (dd, *J* = 9.2, 7.3 Hz, 1H), 4.55 (dd, *J* = 14.7, 7.6 Hz, 1H), 4.30 (dd, *J* = 8.5, 4.4 Hz, 1H), 4.17 (s, 1H), 3.70 – 3.65 (m, 1H), 3.63 – 3.57 (m, 1H), 3.56 – 3.50 (m, 1H), 3.27 – 3.11 (m, 2H), 2.32 – 2.21 (m, 1H), 2.09 (ddd, *J* = 15.8, 12.5, 8.2 Hz, 1H), 2.04 – 1.98 (m, 1H), 1.98 – 1.91 (m, 1H), 1.90 – 1.80 (m, 3H), 1.79 – 1.68 (m, 2H), 1.64 (dt, *J* = 13.6, 6.7 Hz, 1H), 1.47 – 1.41 (m, 2H), 0.98 (d, *J* = 6.6 Hz, 3H), 0.91 (d, *J* = 6.4 Hz, 3H), 0.90 (d, *J* = 4.5 Hz, 3H), 0.89 (d, *J* = 4.4 Hz, 3H).
^{13}C NMR (151 MHz, DMSO-*d*6) δ 193.96, 171.54, 170.17, 168.46, 70.97, 59.59, 59.13, 50.93, 49.63, 47.13, 46.17, 29.88, 29.81, 29.74, 24.97, 24.46, 23.89, 23.65, 22.53, 21.66, 21.51 (2C).
ESI-LRMS: calcd. for $C_{22}H_{37}N_4O_4S$, $[M+H]^+$: 453.2530 (*m/z*); found $[M+H]^+$: 453.2584.

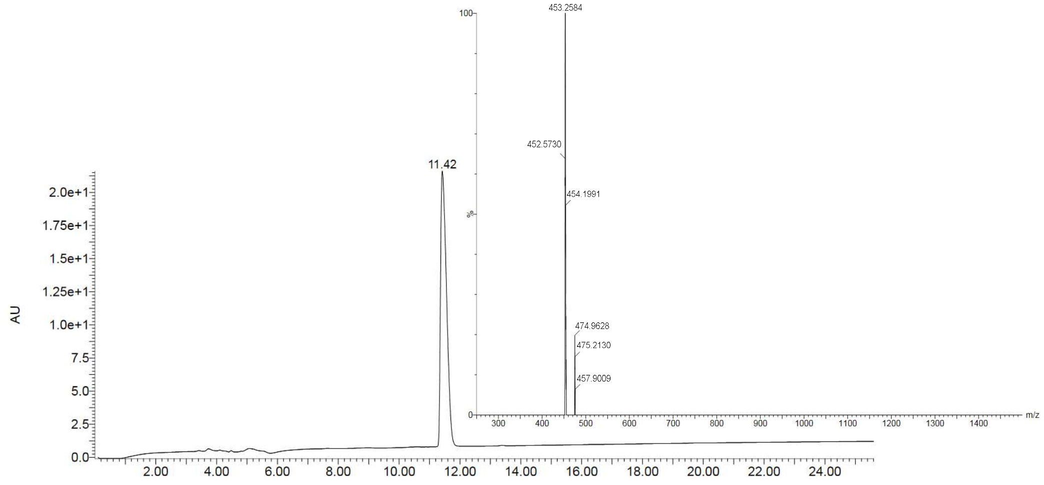

Peptidyl Pen-β-thiolactone S4a

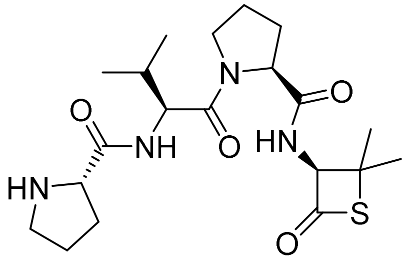

Chemical Formula: $C_{20}H_{32}N_4O_4S$
Exact Mass: 424.2144

S4a

According to the general procedure A and B, the side chain fully protected tripeptide Boc-Pro-Val-Pro-OH was generated from SPPS on a 0.04 mmol scale. The resulting Boc-Pro-Val-Pro-OH was coupled with β-thiolactone **7b** to afford the desired tetrapeptide following the general procedure C. Purification of the crude product using preparative HPLC (20 to 60% solvent B over 20 min, Higgins Analytical Proto 200 5 μm 250 × 10 nm C18 column) afforded peptide **S4a** as a white solid after lyophilization (11.7 mg, 69%).

Analytical HPLC: t_R = 9.72 min (20 to 40% solvent B over 20 min, Higgins Analytical Proto 200 5 μm 150 × 2.0 nm C18 column).
^{1}H NMR (600 MHz, DMSO-*d6*) δ 9.09 – 9.07 (d, *J* = 8.6 Hz, 1H), 8.65 – 8.64 (d, *J* = 8.3 Hz, 1H), 5.60 – 5.59 (d, *J* = 8.6 Hz , 1H), 4.40 – 4.38 (t, *J* = 7.7 Hz, 1H), 4.27 – 4.25 (m, 1H), 4.23 – 4.20 (t, *J* = 7.6 Hz, 1H), 3.68 – 3.65 (m, 1H), 3.60 – 3.56 (m, 1H), 3.21 (m, 1H), 3.17 (m, 1H), 2.29 – 2.25 (td, *J* = 13.9, 7.6 Hz, 1H), 2.11 – 2.07 (dt, *J* = 14.8, 7.4 Hz, 1H), 2.03 – 1.95 (m, 2H), 1.84 – 1.81 (m, 3H), 1.74 – 1.70 (m, 2H), 1.67 (s, 3H), 1.61 (s, 3H), 0.92 – 0.91 (d, *J* = 9.4 Hz, 3H), 0.87 – 0.86 (d, *J* = 6.9 Hz, 3H).

^{13}C NMR (151 MHz, DMSO-*d6*) δ 192.01, 171.90, 169.45, 168.46, 76.88, 59.69, 59.19, 56.42, 52.03, 47.67, 30.53, 30.35, 29.99, 29.71, 26.41, 25.22, 23.92, 19.35, 18.37.
ESI-LRMS: calcd. for $C_{20}H_{33}N_4O_4S$, $[M+H]^+$: 425.2217 (*m/z*); found $[M+H]^+$: 425.2653.

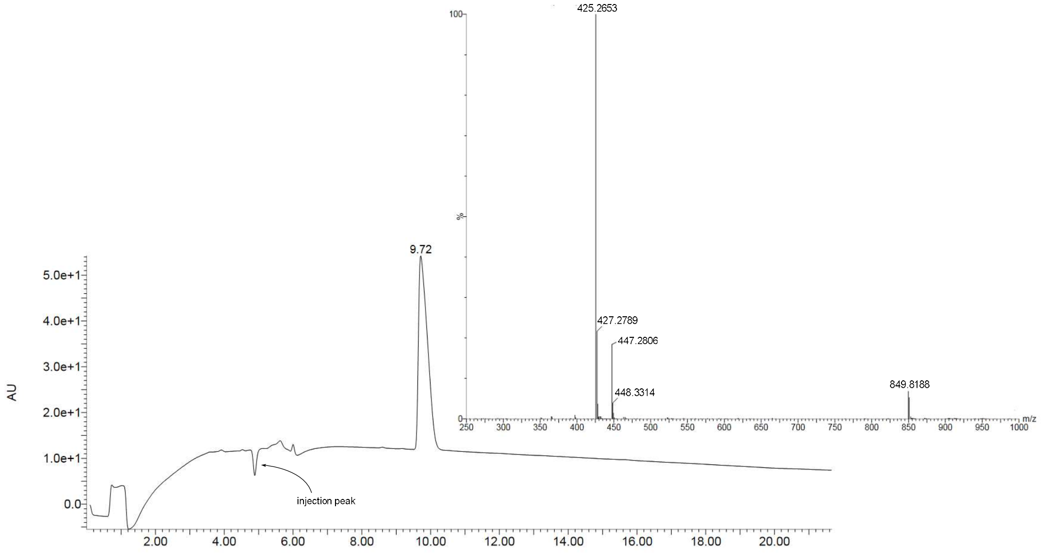

Peptidyl Pen-β-thiolactone S14c

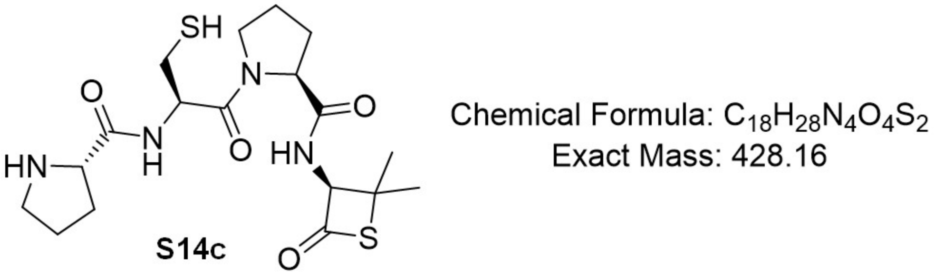

S14c

According to the general procedure A and B, the side chain fully protected tripeptide Boc-Pro-Cys(Trt)-Pro-OH was generated from SPPS on a 0.04 mmol scale. The resulting tripeptide Boc-Pro-Cys(Trt)-Pro-OH coupled with β-thiolactone **7b** to afford the desired tetrapeptide following the general procedure C. Purification of the crude peptide using preparative HPLC (18 to 40% solvent B over 20 min, Higgins Analytical Proto 200 5 µm 250 × 10 nm C18 column) afforded peptide **S14c** as a white solid after lyophilization (12.5 mg, 73%).

Analytical HPLC: t_R = 7.30 min (30 to 50% solvent B over 20 min, Higgins Analytical Proto 200 C18 5 µm 150 × 2.0 nm column).

^{1} H NMR (DMSO-*d6*, 600 MHz) δ 9.09 – 9.08 (d, *J* = 8.6 Hz, 1H), 8.82 – 8.80 (d, *J* = 7.4 Hz, 1H), 7.77 – 7.75 (t, *J* = 6.0 Hz, 1H), 5.56 – 5.54 (d, *J* = 8.6 Hz, 1H), 4.51 – 4.48 (m, 1H), 4.46 – 4.43 (t, J = 7.68 Hz, 1H), 4.27 – 4.25 (dd, *J* = 5.1, 8.2 Hz, 1H), 3.63 – 3.55 (m, 3H), 3.26 –3.25 (d, *J* = 5.9 Hz, 1H), 3.08 – 3.05 (m, 3H), 2.24 – 2.21 (t, *J* = 7.3 Hz, 2H), 2.13 – 2.07 (m, 1H), 1.95 – 1.91 (m, 1H), 1.87 – 1.83 (m, 1H), 1.75 – 1.69 (m, 2H), 1.66 (s, 3H), 1.58 (m, 3H), 1.53 (m, 1H).

^{13} C NMR (DMSO-*d6*, 151 MHz) δ 191.84, 171.89, 168.52, 168.21, 76.87, 60.05, 59.27, 53.98, 51.89, 47.59, 46.18, 30.42, 29.85, 29.79, 26.23, 25.83, 25.06, 23.91.

ESI-LRMS: calcd. for $C_{18}H_{29}N_4O_4S_2$, $[M+H]^+$: 429.1625 (*m/z*); found $[M+H]^+$: 429.1609.

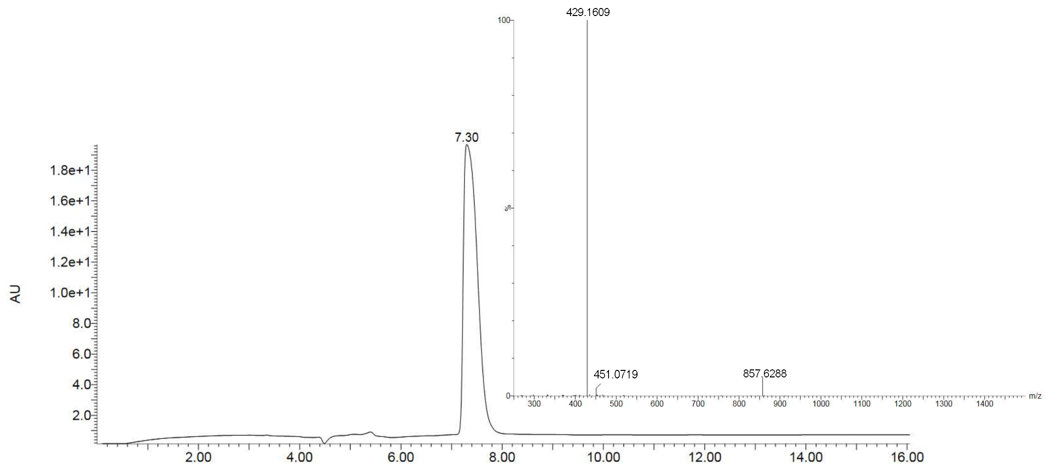

Peptidyl Pen-β-thiolactone S14d

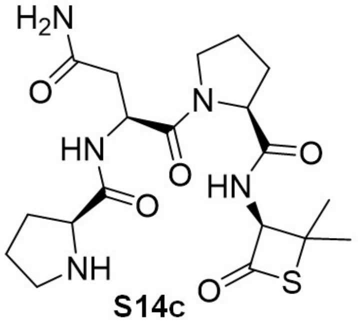

Chemical Formula: $C_{19}H_{29}N_5O_5S$
Exact Mass: 439.19

According to the general procedure A and B, the side chain fully protected tripeptide Boc-Pro-Asn(Trt)-Pro-OH was generated from SPPS on a 0.04 mmol scale. Boc-Pro-Asn(Trt)-Pro-OH was coupled with β-thiolactone **7b** to afford the desired tetrapeptide following the general procedure C. Purification of the crude product using preparative HPLC (10 to 40% solvent B over 20 min, Higgins Analytical Proto 200 5 µm 250 × 10 nm C18 column) afforded peptide **S14d** as a white solid after lyophilization (15.2 mg, 86%).

Analytical HPLC: t_R = 9.24 min (20 to 40% solvent B over 20 min, Higgins Analytical Proto 200 5 µm 150 × 2.0 nm C18 column).

^{1}H NMR (600 MHz, DMSO-*d*6) δ 8.88 – 8.84 (dd, *J* = 15.7, 7.9 Hz, 2H), 7.53 (s, 1H), 6.99 (s, 1H), 5.54 – 5.53 (d, *J* = 8.5 Hz, 1H), 4.84 – 4.81 (m, 1H), 4.28 – 4.26 (dd, *J* = 8.5, 3.9 Hz, 1H), 4.13 – 4.11 (t, *J* = 7.5 Hz, 1H), 3.65 – 3.64 (m, 2H), 3.20 – 3.15 (m, 2H), 2.58 – 2.55 (m, 1H), 2.43 – 2.39 (dd, *J* = 15.7, 7.9 Hz, 1H), 2.28 – 2.24 (m, 1H), 2.10 – 2.05 (m, 1H), 187 – 1.74 (m, 6H), 1.68 (s, 3H), 1.58 (s, 3H).

^{13}C NMR (151 MHz, DMSO-*d*6) δ 191.47, 171.58, 171.38, 169.90, 168.28, 76.78, 59.99, 59.17, 51.69, 48.51, 47.26, 46.19, 37.02, 30.50, 29.83, 26.08, 24.82, 23.90, 22.78.

ESI-LRMS: calcd. for $C_{19}H_{30}N_5O_5S$, $[M+H]^+$: 440.1962 (*m/z*); found $[M+H]^+$: 440.1768.

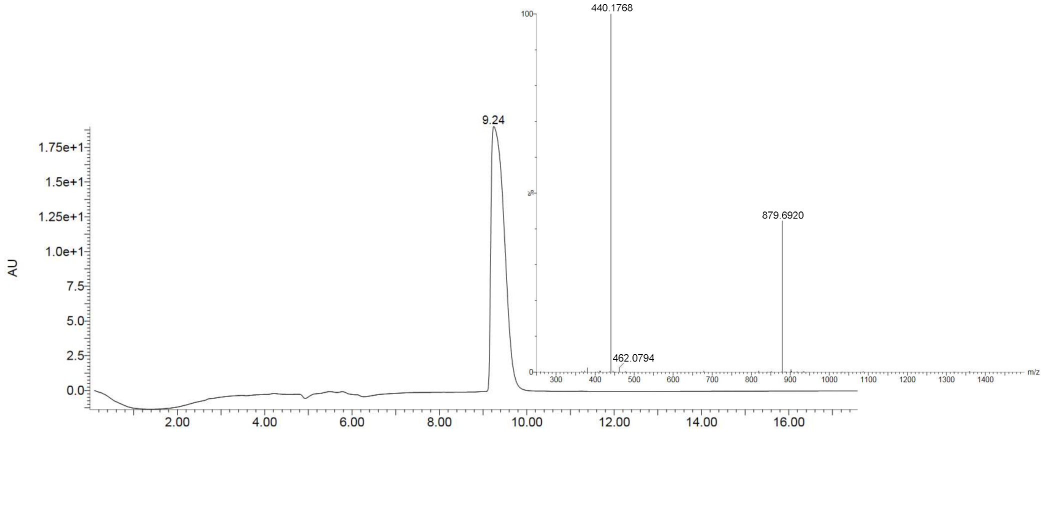

Peptidyl Pen-β-thiolactone S14f

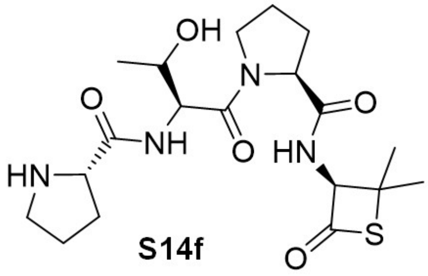

S14f

Chemical Formula: $C_{19}H_{30}N_4O_5S$
Exact Mass: 426.19

According to the general procedure A and B, the side chain fully protected tripeptide Boc-Pro-Thr(^{t}Bu)-Pro-OH was generated from SPPS on a 0.04 mmol scale. The resulting tripeptide Boc-Pro-Thr(^{t}Bu)-Pro-OH was coupled with β-thiolactone **7b** to afford the desired tetrapeptide following the general procedure C. Purification of the crude product using preparative HPLC (22 to 60% solvent B over 20 min, Higgins Analytical Proto 200 5 µm 250 × 10 nm C18 column) afforded peptide **S14f** as a white solid after lyophilization (9.5 mg, 56%).

Analytical HPLC: t_R = 11.04 min (20 to 30% solvent B over 20 min, Higgins Analytical Proto 200 5 µm 150 × 2.0 nm C18 column).
^{1}H NMR (600 MHz, DMSO-*d6*) δ 9.01 (d, *J* = 8.6 Hz, 1H), 8.71 (d, *J* = 7.0 Hz, 1H), 5.58 (d, *J* = 8.6 Hz, 1H), 4.98 (s, 1H), 4.44 (t, *J* = 7.1 Hz, 1H), 4.28 (dd, *J* = 7.6, 5.9 Hz, 1H), 4.21 (t, *J* = 7.6 Hz, 1H), 3.83 (s, 1H), 3.68 (dt, *J* = 16.6, 8.0 Hz, 2H), 3.24 – 3.11 (m, 2H), 2.27 (tt, *J* = 16.5, 8.3 Hz, 1H), 2.09 (dt, *J* = 15.0, 7.5 Hz, 1H), 1.93 (td, *J* = 12.9, 6.7 Hz, 1H), 1.88 – 1.78 (m, 3H), 1.76 – 1.69 (m, 2H), 1.67 (s, 3H), 1.58 (s, 3H), 1.11 (d, *J* = 6.2 Hz, 3H).
^{13}C NMR (151 MHz, DMSO-*d6*) δ 191.89, 171.86, 168.53 (2C), 76.70, 67.46, 59.72, 59.25, 57.51, 51.91, 47.91, 46.23, 30.31, 29.96, 29.77, 26.33, 25.10, 23.97, 19.57.
ESI-LRMS: calcd. for $C_{19}H_{31}N_4O_5S$, $[M+H]^+$: 427.2010 (*m/z*); found $[M+H]^+$: 427.2094.

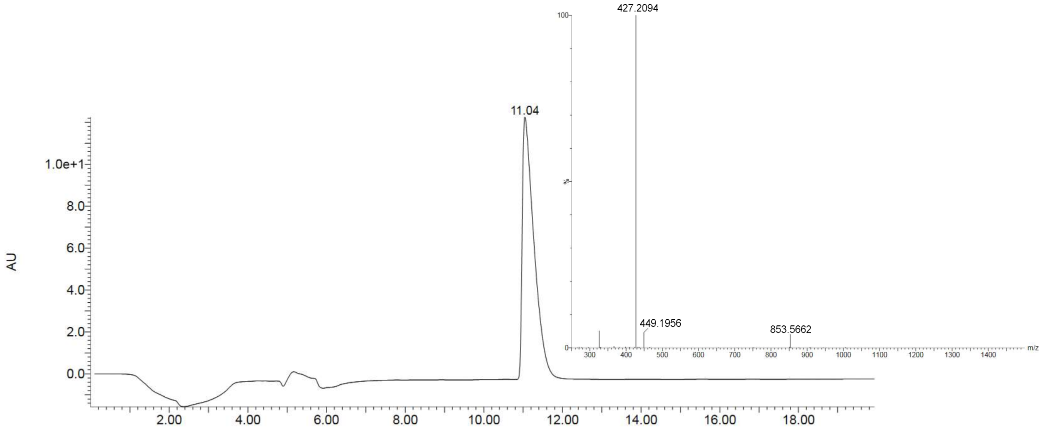

Peptidyl Pen-β-thiolactone S14g

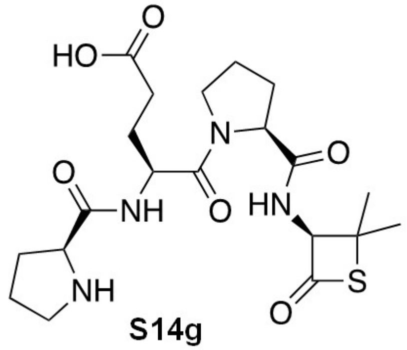

S14g

Chemical Formula: $C_{20}H_{30}N_4O_6S$
Exact Mass: 454.1886

According to the general procedure A and B, the side chain fully protected tripeptide Boc-Pro-Glu(O^{t}Bu)-Pro-OH was generated from SPPS on a 0.04 mmol scale. The resulting tripeptide Boc-Pro-Glu(O^{t}Bu)-Pro-OH was coupled with β-thiolactone **7b** to afford the desired tetrapeptide following the general procedure C. Purification of the crude product using preparative HPLC (15 to 30% solvent B over 20 min, Higgins Analytical Proto 200 5 µm 250 × 10 nm C18 column) afforded peptide **S14g** as a white solid after lyophilization (12.5 mg, 69%).

Analytical HPLC: t_R = 8.40 min (10 to 30% solvent B over 20 min, Higgins Analytical Proto 200 5 µm 150 × 2.0 nm C18 column).
^{1}H NMR (600 MHz, DMSO-*d6*) δ 12.14 (s, 1H), 8.07 – 8.05 (d, *J* = 9.3 Hz, 1H), 7.85 – 7.84 (d, *J* = 9.4 Hz, 1H), 4.63 – 4.62 (m, 1H), 4.46 – 4.43 (dd, *J* = 9.1, 4.4 Hz, 1H), 4.17 (m, 2H), 3.48 – 3.45 (m, 2H), 3.18 – 3.16 (m, 2H), 2.11 – 2.10 (m, 3H), 2.01 – 2.00 (m, 2H), 1.96 – 1.92 (m, 2H), 1.88 – 1.87 (m, 1H), 1.83 – 1.81 (m, 1H), 1.63 – 1.61 (m, 2H), 1.51 (m, 2H), 0.82 – 0.78 (m, 6H).

^{13}C NMR (151 MHz, DMSO-*d6*) δ 174.75, 171.54 (2C), 171.27, 170.87, 61.65, 55.02, 50.07, 46.98, 46.83, 31.92, 31.72, 30.25, 29.46, 29.14, 27.11, 22.07, 21.91, 20.95, 17.91.

ESI-LRMS: calcd. for $C_{20}H_{31}N_4O_6S$, $[M+H]^+$: 455.1959 (*m/z*); found $[M+H]^+$: 455.1900.

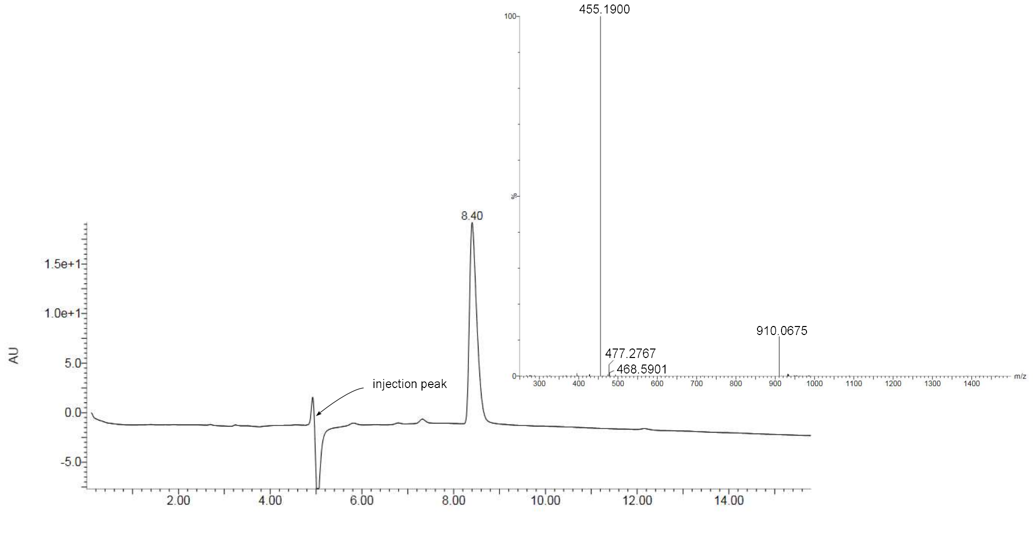

Peptidyl Pen-β-thiolactone S14h

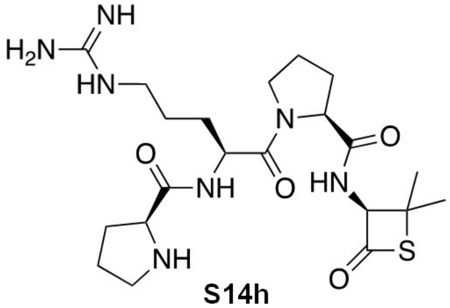

S14h

Chemical Formula: $C_{21}H_{35}N_7O_4S$
Exact Mass: 481.2471

According to the general procedure A and B, the side chain fully protected tripeptide Boc-Pro-Arg(Pbf)-Pro-OH was generated from SPPS on a 0.04 mmol scale. The resulting tripeptide Boc-Pro-Arg(Pbf)-Pro-OH coupled with β-thiolactone **7b** to afford the desired tetrapeptide following the general procedure C (the global deprotection step take 1.5h for this example). Purification of the crude product using preparative HPLC (15 to 20% solvent B over 20 min, Higgins Analytical Proto 200 5 μm 250 × 10 nm C18 column) afforded peptide **S14h** as a white solid after lyophilization (9.0 mg, 47%).

Analytical HPLC: t_R = 8.72 min (5 to 10% solvent B over 20 min, Higgins Analytical Proto 200 5 μm 150 × 2.0 nm C18 column).

^{1}H NMR (600 MHz, DMSO-*d6*) δ 9.33 (s, 1H), 9.10 – 9.08 (d, *J* = 8.7 Hz, 1H), 8.77 – 8.75 (d, *J* = 7.7 Hz, 1H), 8.49 (s, 1H), 7.66 – 7.64 (t, *J* = 5.5 Hz, 1H), 5.59 – 5.57 (d, *J* = 8.6 Hz, 1H), 4.543 – 4.50 (m, 1H), 4.28 – 4.26 (dd, *J* = 8.4, 5.2 Hz, 1H), 4.18 (s, 1H), 3.65 – 3.61 (m, 1H), 3.58 – 3.55 (m, 1H), 3.18 (m, 2H), 3.10 – 3.06 (m, 2H), 2.30 – 2.25 (m, 1H), 2.14 – 2.09 (m, 1H), 1.98 – 1.93 (m, 1H), 1.90 – 1.71 (m, 7H), 1.68 (s, 3H), 1.60 (s, 3H), 1.53 (s, 2H).

^{13}C NMR (151 MHz, DMSO-*d6*) δ 191.99, 171.79, 169.46, 168.43, 157.21, 77.34, 76.90, 59.69, 59.19, 51.95, 51.03, 47.34, 46.22, 30.38, 29.95, 29.77, 26.33, 25.53, 25.13, 24.94, 23.92.

ESI-LRMS: calcd. for $C_{21}H_{36}N_7O_4S$, $[M+H]^+$: 482.2544 (*m/z*); found $[M+H]^+$: 482.2524.

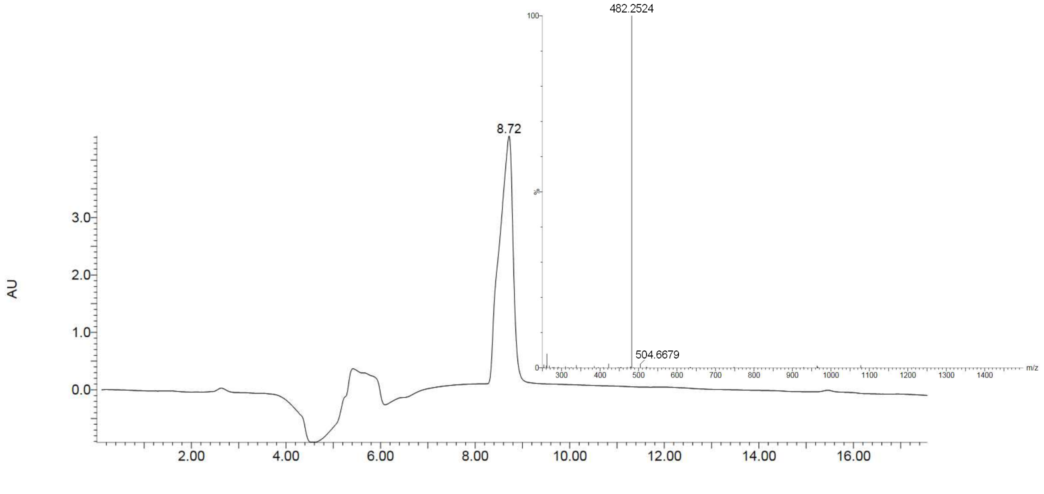

Peptidyl Pen-β-thiolactone S14i

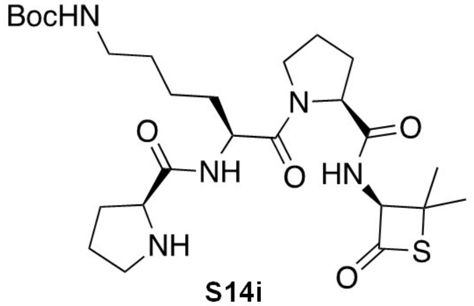

S14i

Chemical Formula: $C_{26}H_{43}N_5O_6S$
Exact Mass: 553.2934

According to the general procedure A and B, the side chain fully protected tripeptide H-Pro-Lys(NHBoc)-Pro-OH was generated from SPPS on a 0.04 mmol scale. The resulting tripeptide H-Pro-Lys(NHBoc)-Pro-OH was coupled with β-thiolactone **7b** to afford the desired tetrapeptide following the general procedure C (without the global deprotection step). Purification of the crude product using preparative HPLC (10 to 30% solvent B over 20 min, Higgins Analytical Proto 200 5 µm 250 × 10 nm C18 column) afforded peptide **S14i** as a white solid after lyophilization (5.1 mg, 23%).

Analytical HPLC: t_R = 12.77 min (30 to 60% solvent B over 20 min, Higgins Analytical Proto 200 5 µm 150 × 2.0 nm C18 column).
^{1}H NMR (500 MHz, DMSO-*d6*) δ 9.06 (d, *J* = 8.6 Hz, 1H), 8.74 (d, *J* = 7.6 Hz, 1H), 6.77 (t, *J* = 5.4 Hz, 1H), 5.60 (d, *J* = 8.6 Hz, 1H), 4.51 (dd, *J* = 12.9, 7.9 Hz, 1H), 4.29 (dd, *J* = 8.3, 5.1 Hz, 1H), 4.21 (d, *J* = 7.1 Hz, 1H), 3.65 (dd, *J* = 11.4, 4.7 Hz, 1H), 3.62 – 3.55 (m, 1H), 3.21 (d, *J* = 11.2 Hz, 3H), 2.89 (dd, *J* = 12.5, 6.4 Hz, 2H), 2.35 – 2.23 (m, 2H), 2.12 (dt, *J* = 12.5, 7.8 Hz, 1H), 2.04 – 1.95 (m, 1H), 1.93 – 1.82 (m, 4H), 1.78 (dt, *J* = 12.6, 6.0 Hz, 3H), 1.71 (s, 3H), 1.62 (s, 3H), 1.58 – 1.49 (m, 1H), 1.37 (s, 9H), 1.35 – 1.26 (m, 2H).
^{13}C NMR (151 MHz, DMSO-*d6*) δ 191.96, 171.86, 169.81, 168.39, 156.01, 77.86, 76.95, 59.72, 59.19, 51.98, 51.36, 47.34, 46.21, 31.05, 30.41, 29.94, 29.73, 29.69, 28.73 (4C), 26.36, 25.19, 23.94, 22.60.
ESI-LRMS: calcd. for $C_{26}H_{44}N_5O_6S$, $[M+H]^+$: 554.3007 (*m/z*); found $[M+H]^+$: 554.3155.

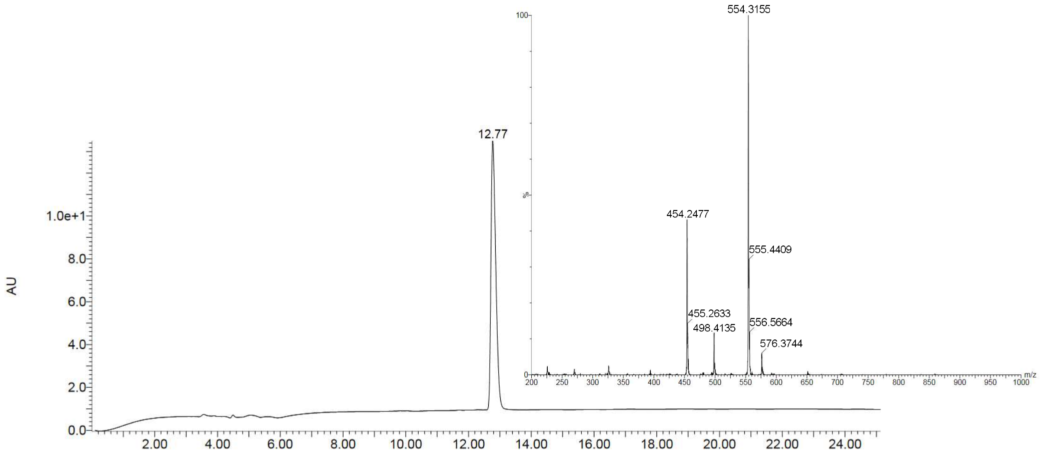

Peptidyl Pen-β-thiolactone S14j

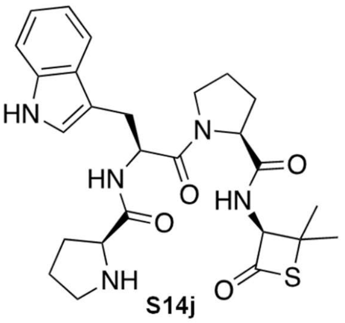

Chemical Formula: $C_{26}H_{33}N_5O_4S$
Exact Mass: 511.2253

According to the general procedure A and B, the side chain fully protected tripeptide Boc-Pro-Trp(Boc)-Pro-OH was generated from SPPS on a 0.04 mmol scale. The resulting tripeptide Boc-Pro-Trp(Boc)-Pro-OH was coupled with β-thiolactone **7b** to afford the desired tetrapeptide following the general procedure C (1h needed for the global deprotection). Purification of the crude product using preparative HPLC (20 to 60% solvent B over 20 min, Higgins Analytical Proto 200 5 µm 250 × 10 nm C18 column) yielded **S14j** as a white solid after lyophilization (14.9 mg, 73%).

Analytical HPLC: t_R = 13.00 min (20 to 60% solvent B over 20 min, Higgins Analytical Proto 200 C18 5 µm 150 × 2.0 nm column).
^{1}H NMR (600 MHz, DMSO-*d6*) δ 10.90 (s, 1H), 9.06 – 9.05 (d, *J* = 8.5 Hz, 1H), 8.88 – 8.86 (d, *J* = 7.68 Hz, 1H), 7.58 – 7.57 (d, *J* = 7.7 Hz, 1H), 7.33 – 8.32 (m, 1H), 7.22 (s, 1H), 7.06 – 7.04 (m, 1H), 7.00 – 6.96 (m, 1H), 5.62 – 5.60 (d, *J* = 8.5 Hz, 1H), 4.77 – 4.74 (m, 1H), 4.31 – 4.29 (m, 1H), 4.10 – 4.09 (m, 1H), 3.61 – 3.58 (m, 1H), 3.47 – 3.45 (m, 1H), 3.18 – 3.13 (m, 3H), 2.94 – 2.90 (m, 1H), 2.28 – 2.26 (m, 1H), 2.13 – 2.10 (m, 1H), 1.95 – 1.92 (m, 1H), 1.86 – 1.82 (m, 3H), 1.81 – 1.77 (m, 2H), 1.74 (s, 3H), 1.63 (s, 3H).
^{13}C NMR (151 MHz, DMSO-*d6*) δ 191.95, 171.85, 169.83, 168.43, 136.55, 127.59, 124.73, 121.37, 118.95, 118.31, 111.93, 109.65, 77.04, 59.95, 59.16, 52.44, 51.99, 47.39, 46.21, 30.55, 30.01, 29.75, 27.30, 26.30, 25.19, 23.92.
ESI-LRMS: calcd. for $C_{26}H_{34}N_5O_4S$, $[M+H]^+$: 512.2326 (*m/z*); found $[M+H]^+$: 512.2549.

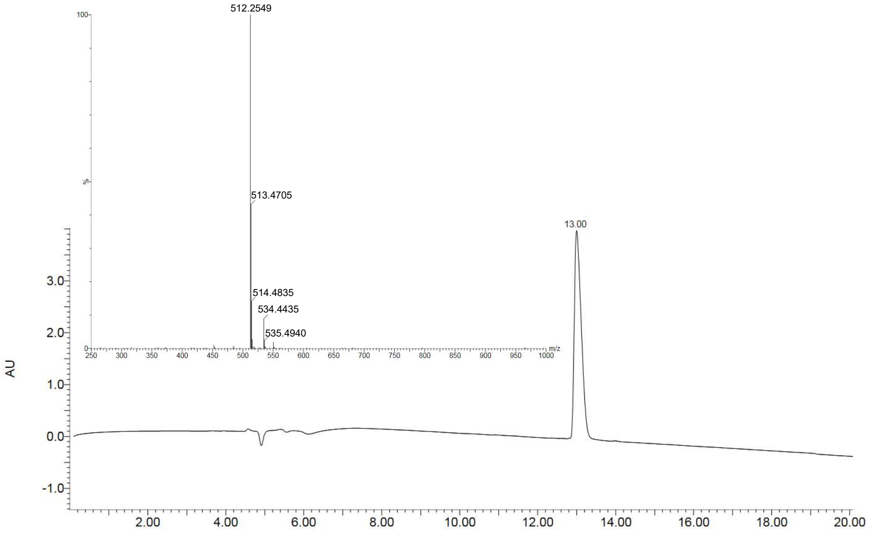

Peptidyl Pen-β-thiolactone S14k

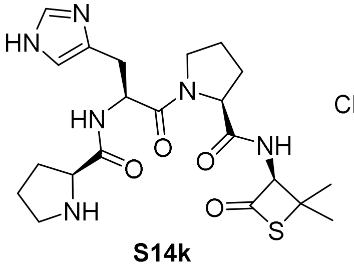

Chemical Formula: $C_{21}H_{30}N_6O_4S$
Exact Mass: 462.2049

S14k

According to the general procedure A and B, the side chain fully protected tripeptide Boc-Pro-His-Pro-OH was generated from SPPS on a 0.04 mmol scale. The resulting tripeptide Boc-Pro-His-Pro-OH was coupled with β-thiolactone **7b** to afford the desired tetrapeptide following the general procedure C. Purification of the crude product using preparative HPLC (20 to 40% solvent B over 20 min, Higgins Analytical Proto 200 5 µm 250 × 10 nm C18 column) afforded peptide **S14k** as a white solid after lyophilization (12.4 mg, 67%).

Analytical HPLC: t_R = 12.58 min (15 to 30% solvent B over 20 min, Higgins Analytical Proto 200 5 µm 150 × 2.0 nm C18 column).
^{1}H NMR (500 MHz, DMSO-d_6) δ 9.42 (s, 1H), 9.17 (d, *J* = 8.5 Hz, 1H), 8.98 (s, 1H), 8.90 (d, *J* = 7.9 Hz, 1H), 8.51 (s, 1H), 5.60 (d, *J* = 8.4 Hz, 1H), 4.90 (td, *J* = 7.6, 5.7 Hz, 1H), 4.32 (dd, *J* = 8.5, 5.1 Hz, 1H), 4.19 (t, *J* = 7.9 Hz, 1H), 3.64 (td, *J* = 6.7, 2.2 Hz, 2H), 3.28 – 3.19 (m, 2H), 3.12 (dd, *J* = 15.3, 5.6 Hz, 1H), 2.97 (dd, *J* = 15.3, 7.5 Hz, 1H), 2.34 – 2.24 (m, 1H), 2.17 (dq, *J* = 12.4, 7.5 Hz, 1H), 1.96 (dd, *J* = 13.1, 6.6 Hz, 1H), 1.93 – 1.83 (m, 4H), 1.78 (dt, *J* = 12.6, 7.0 Hz, 2H), 1.73 (s, 3H), 1.61 (s, 3H).
^{13}C NMR (126 MHz, DMSO-d_6) δ 191.84, 171.96, 168.56, 168.08, 134.29, 128.90, 117.96, 77.02, 59.98, 59.26, 51.88, 50.57, 47.53, 46.22, 30.57, 29.96, 29.93, 26.63, 26.26, 25.11, 23.94.
ESI-HRMS: calcd. for $C_{21}H_{31}N_6O_4S$, $[M+H]^+$: 463.2122 (*m/z*); found $[M+H]^+$: 463.2121.

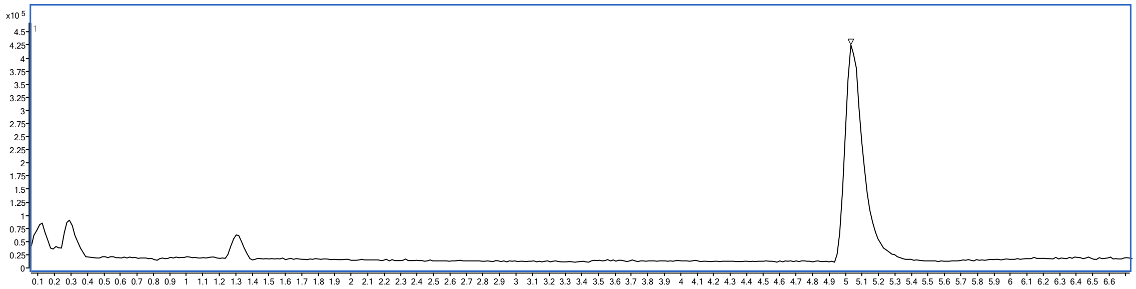

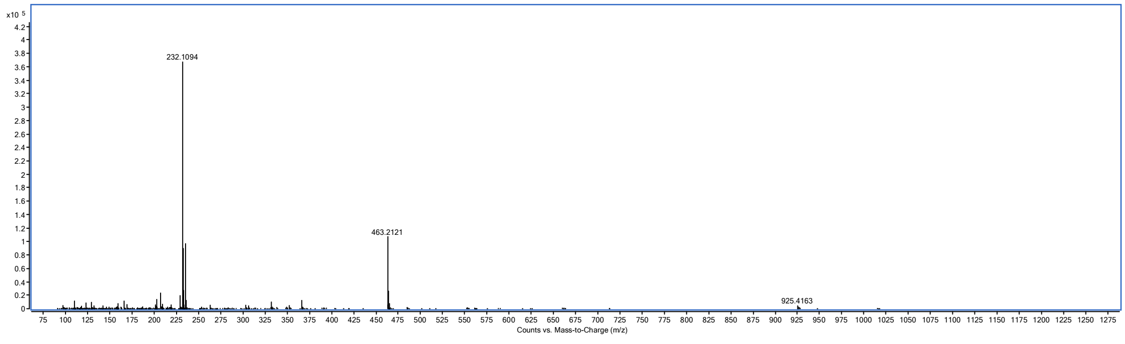

Peptidyl Cys-β-thiolactone S14l

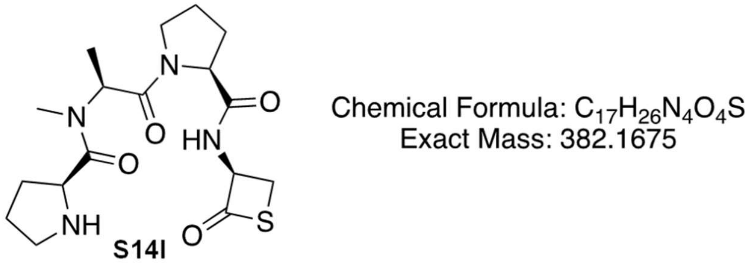

S14l

According to the general procedure A and B, the side chain fully protected tripeptide Boc-Pro-Ala(NMe)-Pro-OH was generated from SPPS on a 0.04 mmol scale. The resulting tripeptide Boc-Pro-Ala(NMe)-Pro-OH was coupled with β-thiolactone **7a** to afford the desired tetrapeptide following the general procedure C. Purification of the crude product using preparative HPLC (10 to 30% solvent B over 20 min, Higgins Analytical Proto 200 5 μm 250 × 10 nm C18 column) afforded peptide **S14l** as a white solid after lyophilization (4.9 mg, 32%).

Analytical HPLC: t_R = 11.65 min (20 to 60% solvent B over 20 min, Higgins Analytical Proto 200 5 μm 150 × 2.0 nm C18 column).
^{1}H NMR (500 MHz, DMSO-*d6*) δ 8.73 (d, *J* = 8.0 Hz, 1H), 5.58 (td, *J* = 7.9, 5.0 Hz, 1H), 5.16 (q, *J* = 6.9 Hz, 1H), 4.61 (s, 1H), 4.22 (dd, *J* = 8.8, 3.4 Hz, 1H), 3.61 – 3.52 (m, 1H), 3.41 (t, *J* = 7.8 Hz, 1H), 3.29 (dt, *J* = 9.5, 7.5 Hz, 2H), 3.23 (dd, *J* = 7.8, 5.0 Hz, 2H), 3.19– 3.16 (m, 1H), 2.90 (s, 3H), 2.48 – 2.39 (m, 1H), 2.14 – 2.04 (m, 1H), 2.00 – 1.90 (m, 2H), 1.90 – 1.82 (m, 2H), 1.79 – 1.67 (m, 2H), 1.22 (d, *J* = 7.0 Hz, 3H).
^{13}C NMR (126 MHz, DMSO-*d6*) δ 193.94, 172.18, 169.06, 168.73, 71.41, 60.10, 58.56, 51.76, 46.69, 46.06, 30.59, 29.84, 28.58, 26.64, 24.77, 24.04, 13.97.
ESI-LRMS: calcd. for $C_{17}H_{27}N_4O_4S$, $[M+H]^+$: 383.1748 (*m/z*); found $[M+H]^+$: 383.1611.

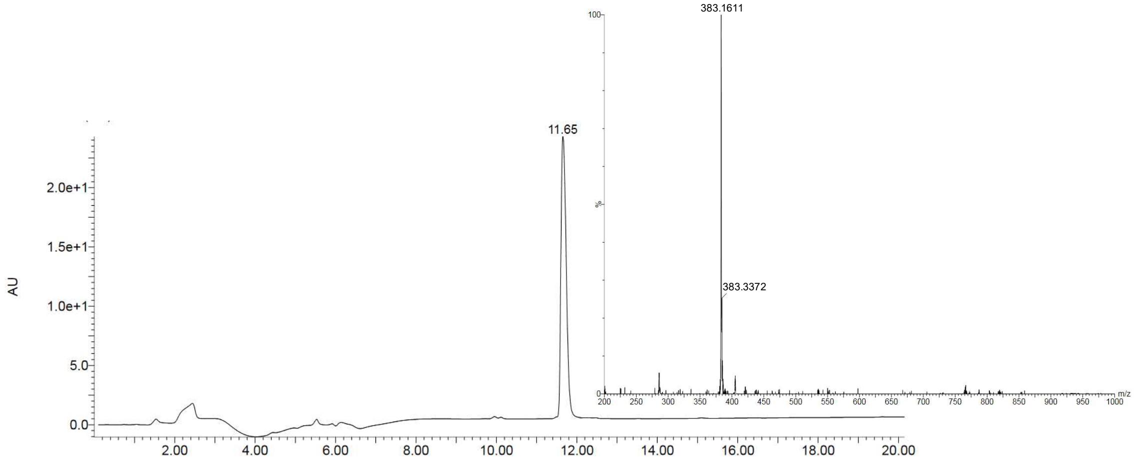

Peptidyl Pen-β-thiolactone S14m

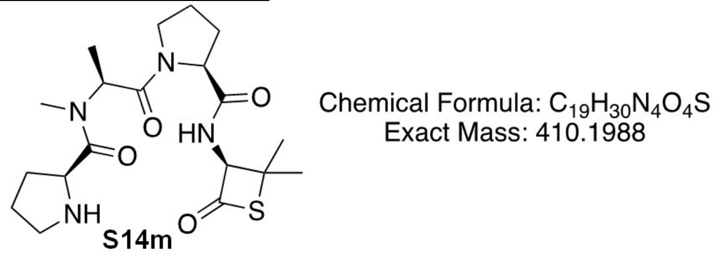

According to the general procedure A and B, the side chain fully protected tripeptide Boc-Pro-Ala(NMe)-Pro-OH was generated from SPPS on a 0.04 mmol scale. The resulting tripeptide Boc-Pro-Ala(NMe)-Pro-OH was coupled with β-thiolactone **7b** to afford the desired tetrapeptide following the general procedure C. Purification of the crude product using preparative HPLC (10 to 30% solvent B over 20 min, Higgins Analytical Proto 200 5 µm 250 × 10 nm C18 column) afforded peptide **S14m** as a white solid after lyophilization (12.6 mg, 77%).

Analytical HPLC: t_R = 10.73 min (20 to 60% solvent B over 20 min, Higgins Analytical Proto 200 5 µm 150 × 2.0 nm C18 column).

^{1}H NMR (600 MHz, DMSO-*d6*) δ 8.88 – 8.87 (d, *J* = 8.22 Hz, 1H), 5.54 – 5.53 (d, *J* = 8.4 Hz, 1H), 5.15 – 5.14 (m, 1H), 4.56 (s, 1H), 4.24 – 4.22 (dd, *J* = 8.7, 3.1 Hz, 1H), 3.55 (d, *J* = 9.3 Hz, 2H), 3.28 – 3.24 (m, 2H), 2.87 (s, 3H), 2.45 – 2.38 (m, 1H), 2.12 – 2.07 (m, 1H), 1.95 – 1.81 (m, 5H), 1.77 – 1.73 (m, 1H), 1.69 (s, 3H), 1.57 (s, 3H), 1.20 – 1.19 (d, *J* = 6.84 Hz, 3H).

^{13}C NMR (151 MHz, DMSO-*d6*) δ 191.86, 171.82, 168.88, 168.66, 76.88, 60.08, 58.61, 51.80, 51.68, 46.86, 46.10, 30.56, 30.51, 29.86, 28.56, 26.09, 24.87, 24.00, 13.98.

ESI-LRMS: calcd. for $C_{19}H_{31}N_4O_4S$, $[M+H]^+$: 411.2061 (*m/z*); found $[M+H]^+$: 411.2544.

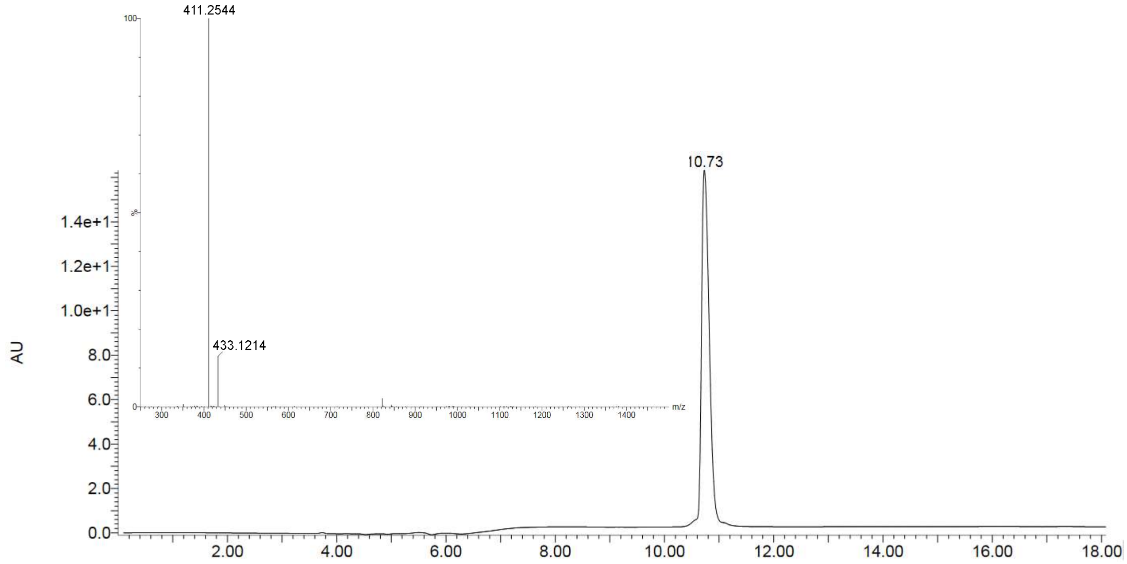

Peptidyl Cys-β-thiolactone S14o

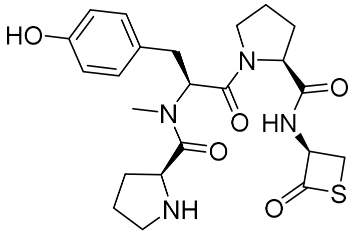

Chemical Formula: $C_{23}H_{30}N_4O_5S$
Exact Mass: 474.1937

S14o

According to the general procedure A and B, the side chain fully protected tripeptide Boc-Pro-Tyr (NMe)-Pro-OH was generated from SPPS on a 0.04 mmol scale. Then follow the general procedure C, Boc-Pro-Tyr (NMe)-Pro-OH coupled with β-thiolactone **7a** to afford the desired tetrapeptide. Purification of the crude peptide using preparative HPLC (20 to 70% solvent B over 20 min, Higgins Analytical Proto 200 5 µm 250 × 10 nm C18 column) afforded peptide **S14o** as a white solid after lyophilization (4.3 mg, 23%).

Analytical HPLC: t_R = 13.79 min (10 to 30% solvent B over 20 min, Higgins Analytical Proto 200 C18 5 µm 150 × 2.0 nm column).

^{1}H NMR (500 MHz, DMSO) δ 8.72 (d, *J* = 8.0 Hz, 1H), 7.03 (d, *J* = 8.5 Hz, 2H), 6.67 (d, *J* = 8.5 Hz, 2H), 5.59 (td, *J* = 7.9, 5.0 Hz, 1H), 5.28 (dd, *J* = 9.3, 5.6 Hz, 1H), 4.55 – 4.47 (m, 1H), 4.24 (dd, *J* = 8.8, 3.6 Hz, 1H), 3.55 – 3.52 (m, 1H), 3.42 (t, *J* = 7.8 Hz, 2H), 3.33 (dd, *J* = 17.1, 7.5 Hz, 1H), 3.23 (dd, *J* = 7.8, 5.0 Hz, 2H), 3.16 – 3.08 (m, 2H), 3.05 (dd, *J* = 14.8, 5.4 Hz, 1H), 2.93 (s, 3H), 2.84 (dd, *J* = 14.7, 9.4 Hz, 1H), 2.41 (dt, *J* = 13.7, 8.0 Hz, 1H), 2.12 – 2.04 (m, 1H), 1.96 – 1.80 (m, 4H), 1.76 (ddd, *J* = 16.2, 7.2, 4.2 Hz, 1H), 1.67 (dd, *J* = 12.8, 7.6 Hz, 1H).

^{13}C NMR (151 MHz, DMSO-*d6*) δ 193.88, 172.10, 169.01, 168.48, 156.33, 130.26(2C), 127.68, 115.65(2C), 71.46, 60.18, 58.40, 57.19, 47.00, 46.11, 33.00, 31.39, 29.74, 28.70, 26.63, 24.85, 24.04.

ESI-MS: calcd. for $C_{23}H_{31}N_4O_5S$, $[M+H]^+$: 475.2010 (*m/z*); found $[M+H]^+$: 475.2190.

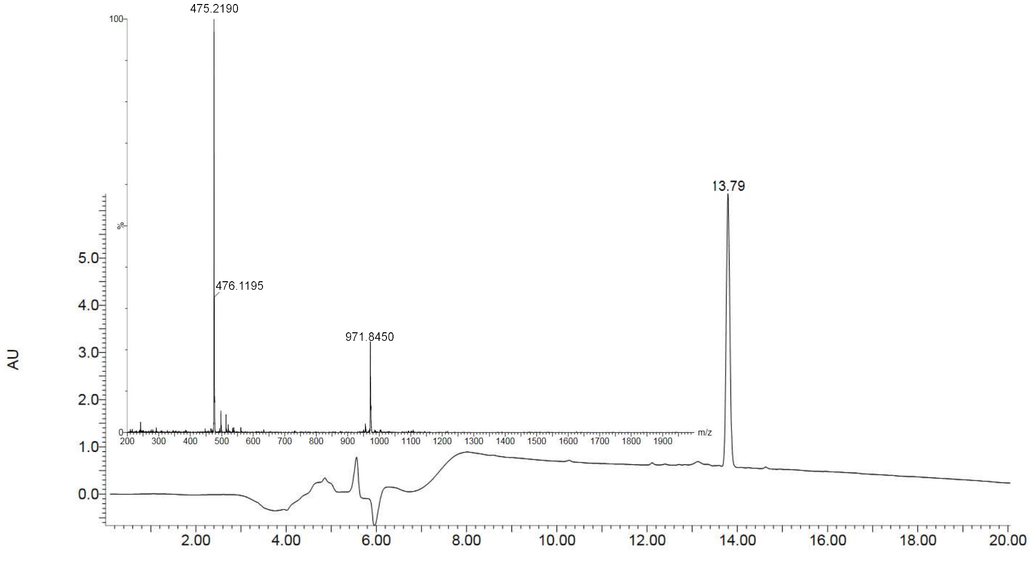

Peptidyl Pen-β-thiolactone S14p

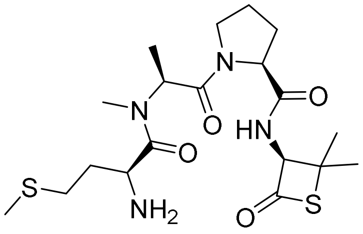

Chemical Formula: $C_{19}H_{32}N_4O_4S_2$
Exact Mass: 444.1865

S14p

According to the general procedure A and B, the side chain fully protected tripeptide Boc-Met-Ala(NMe)-Pro-OH was generated from SPPS on a 0.04 mmol scale. The resulting tripeptide Boc-Met-Ala(NMe)-Pro-OH was coupled with β-thiolactone **7b** to afford the desired tetrapeptide following the general procedure C. Purification of the crude product using preparative HPLC (20 to 30% solvent B over 20 min, Higgins Analytical Proto 200 5 μm 250 × 10 nm C18 column) afforded peptide **S14p** as a white solid after lyophilization (12.6 mg, 71%).

Analytical HPLC: t_R = 13.22 min (20 to 50% solvent B over 20 min, Higgins Analytical Proto 200 5 μm 150 × 2.0 nm C18 column).

^{1}H NMR (500 MHz, DMSO-*d6*) δ 8.90 (d, *J* = 8.5 Hz, 1H), 8.20 (s, 2H), 5.57 (d, *J* = 8.5 Hz, 1H), 5.18 (d, *J* = 7.0 Hz, 1H), 4.44 (s, 1H), 4.25 (dd, *J* = 8.8, 3.9 Hz, 1H), 3.63 – 3.56 (m, 1H), 3.34 (dd, *J* = 17.0, 7.3 Hz, 1H), 2.94 (s, 3H), 2.67 – 2.57 (m, 1H), 2.17 – 2.10 (m, 1H), 2.07 (s, 3H), 2.00 – 1.89 (m, 3H), 1.88 – 1.81 (m, 2H), 1.81 – 1.76 (m, 1H), 1.71 (s, 3H), 1.60 (s, 3H), 1.22 (d, *J* = 7.0 Hz, 3H).

^{13}C NMR (126 MHz, DMSO-*d6*) δ 191.84, 171.77, 169.08, 169.01, 76.93, 60.09, 51.83, 51.46, 49.84, 46.91, 30.64, 30.55, 30.16, 29.89, 28.60, 26.11, 24.94, 15.02, 14.03.

ESI-LRMS: calcd. for $C_{19}H_{33}N_4O_4S_2$, $[M+H]^+$: 445.1938 (*m/z*); found $[M+H]^+$: 445.1569.

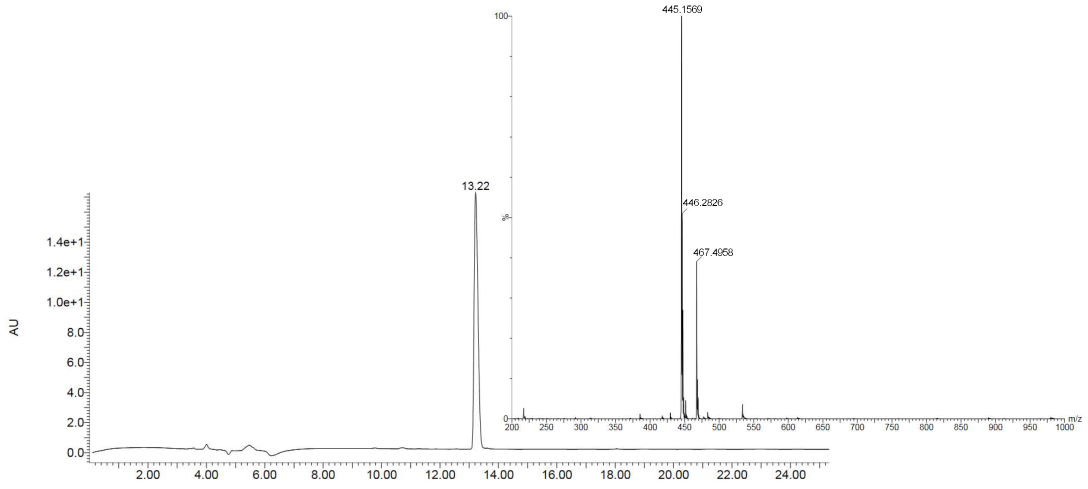

Peptidyl Pen-β-thiolactone S14q

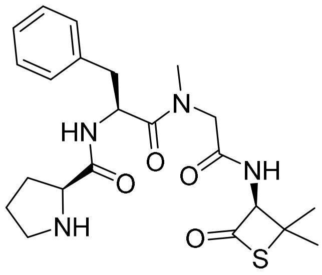

Chemical Formula: $C_{22}H_{30}N_4O_4S$
Exact Mass: 446.1988

S14q

According to the general procedure A and B, the side chain fully protected tripeptide Boc-Pro-Phe-Gly(NMe)-OH was generated from SPPS on a 0.04 mmol scale. The resulting tripeptide Boc-Pro-Phe-Gly(NMe)-OH was coupled with β-thiolactone **7b** to afford the desired tetrapeptide following the general procedure C. Purification of the crude product using preparative HPLC (20 to 60% solvent B over 20 min, Higgins Analytical Proto 200 5 μm 250 × 10 nm C18 column) afforded peptide **S14q** as a white solid after lyophilization (16.6 mg, 81%).

Analytical HPLC: t_R = 9.38 min (20 to 60% solvent B over 20 min, Higgins Analytical Proto 200 5 μm 150 × 2.0 nm C18 column).

^{1}H NMR (600 MHz, DMSO-*d6*) δ 9.04 (d, *J* = 8.6 Hz, 1H), 8.86 (d, *J* = 8.3 Hz, 1H), 7.25 (t, *J* = 5.9 Hz, 3H), 7.22 – 7.17 (m, 2H), 5.62 (d, *J* = 8.7 Hz, 1H), 4.98 (dd, *J* = 13.5, 8.3 Hz, 1H), 4.16 – 4.04 (m, 1H), 3.99 (d, *J* = 18.2 Hz, 2H), 3.18 (s, 1H), 3.14 (d, *J* = 5.6 Hz, 2H), 3.02 (s, 3H), 2.82 (d, *J* = 7.7 Hz, 1H), 2.78 (dd, *J* = 9.6, 4.1 Hz, 1H), 2.27 (m, 1H), 1.84 – 1.77 (m, 3H), 1.71 (s, 3H), 1.55 (s, 3H).

^{13}C NMR (151 MHz, DMSO-*d6*) δ 192.00, 171.12, 168.25, 168.12, 137.49, 129.72 (2C), 128.66 (2C), 127.02, 76.63, 59.16, 51.71, 51.09, 50.69, 46.18, 37.28, 36.94, 30.33, 30.00, 26.20, 23.84.

ESI-LRMS: calcd. for $C_{22}H_{31}N_4O_4S$, $[M+H]^+$: 447.2061 (*m/z*); found $[M+H]^+$: 447.2532.

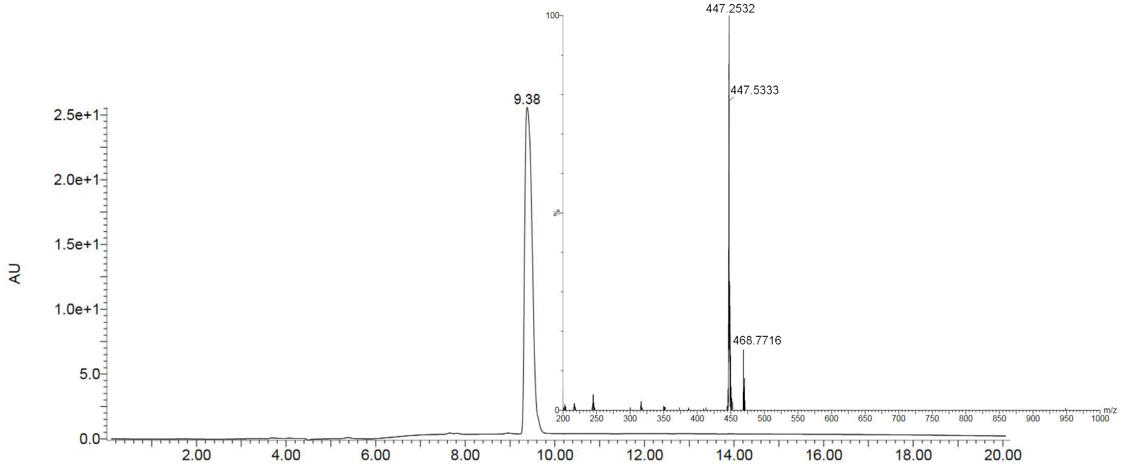

Peptidyl Pen-β-thiolactone S14t

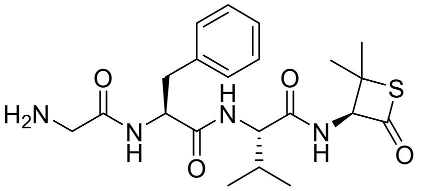

Chemical Formula: $C_{21}H_{30}N_4O_4S$
Exact Mass: 434.1988

S14t

According to the general procedure A and B, the side chain fully protected tripeptide Boc-Gly-Phe-Val-OH was generated from SPPS method on a 0.04 mmol scale. The resulting tripeptide Boc-Gly-Phe-Val-OH was coupled with β-thiolactone **7b** to afford the desired tetrapeptide following the general procedure C. Purification of the crude product using preparative HPLC (20 to 60% solvent B over 20 min, Higgins Analytical Proto 200 5 µm 250 × 10 nm C18 column) afforded peptide **S14t** as a white solid after lyophilization (15.1 mg, 87%).

Analytical HPLC: t_R = 15.82 min (20 to 60% solvent B over 20 min, Higgins Analytical Proto 200 C18 5 µm 150 × 2.0 nm column);

^{1}H NMR (DMSO-*d6*, 600 MHz) δ 9.06 – 9.05 (d, *J* = 8.7 Hz, 1H), 8.55 – 8.54 (d, *J* = 8.2 Hz, 1H), 8.34 – 8.33 (d, *J* = 8.2 Hz, 1H), 7.92 (s, 2H), 7.23 – 7.21 (m, 4H), 7.18 – 7.17 (m, 1H), 5.65 – 5.63 (d, *J* = 8.7 Hz, 1H), 4.76 – 4.72 (m, 1H), 4.11 – 4.08 (t, *J* = 7.8 Hz, 1H), 3.54 – 3.38 (m, 2H), 3.00 – 2.97 (dd, *J* = 4.2 Hz, 1H), 2.74 – 2.70 (dd, *J* = 9.2, 13. 9 Hz, 1H), 1.95 – 1.92 (m, 1H), 1.72 (s, 3H), 1.57 (s, 3H), 0.88-0.86 (m, 1H).

^{13}C NMR (151 MHz, DMSO-*d6*) δ 191.73, 171.15, 171.12, 166.00, 137.74, 129.77, 128.45, 126.81, 76.75, 58.61, 53.99, 51.61, 40.44, 38.25, 30.61, 30.40, 26.28, 19.60, 18.89.

ESI-LRMS: calcd. for $C_{21}H_{31}N_4O_4S$, $[M+H]^+$: 435.2061 (*m/z*); found $[M+H]^+$: 435.2265.

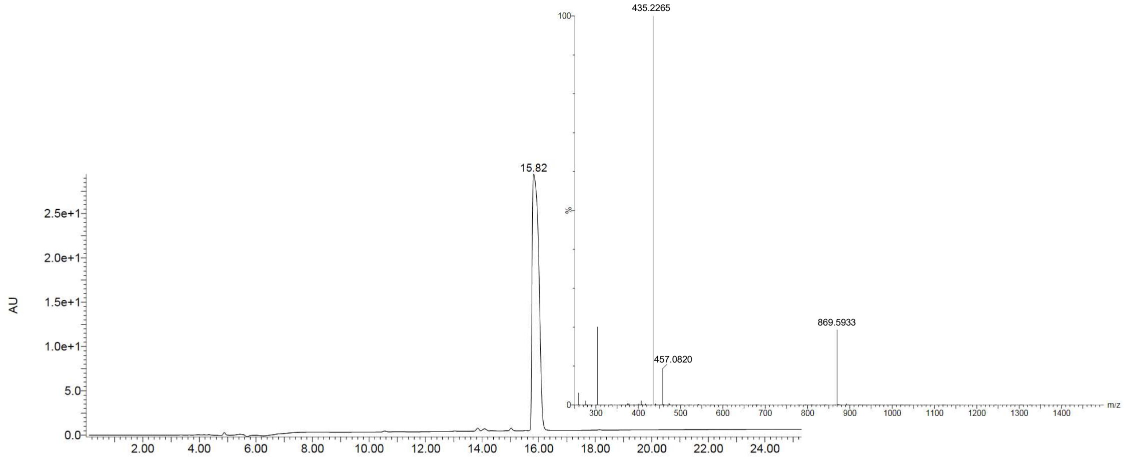

Peptidyl Pen-β-thiolactone S14u

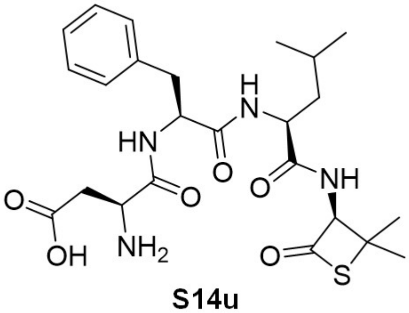

S14u

Chemical Formula: $C_{24}H_{34}N_4O_6S$
Exact Mass: 506.2199

According to the general procedure A and B, the side chain fully protected tripeptide Boc-Asp(O^{t}Bu)-Phe-Val-OH was generated from SPPS on a 0.04 mmol scale. The resulting tripeptide Boc-Asp(O^{t}Bu)-Phe-Val-OH was coupled with β-thiolactone **7b** to afford the desired tetrapeptide following the general procedure C. Purification of the crude product using preparative HPLC (20 to 60% solvent B over 20 min, Higgins Analytical Proto 200 5 µm 250 × 10 nm C18 column) afforded peptide **S14u** as a white solid after lyophilization (15.0 mg, 76%).

Analytical HPLC: t_R = 17.52 min (20 to 60% solvent B over 20 min, Higgins Analytical Proto 200 5 µm 150 × 2.0 nm C18 column).
^{1}H NMR (500 MHz, DMSO-*d6*) δ 9.10 (d, *J* = 8.6 Hz, 1H), 8.60 (d, *J* = 8.2 Hz, 1H), 8.37 (d, *J* = 7.7 Hz, 1H), 7.32 – 7.25 (m, 4H), 7.25 – 7.15 (m, 1H), 5.62 (d, *J* = 8.6 Hz, 1H), 4.60 (td, *J* = 9.2, 4.2 Hz, 1H), 4.29 (ddd, *J* = 9.9, 7.7, 5.4 Hz, 1H), 3.98 (dd, *J* = 9.3, 3.0 Hz, 1H), 3.08 (dd, *J* = 14.0, 4.1 Hz, 1H), 2.82 (dd, *J* = 17.8, 3.0 Hz, 1H), 2.75 (dd, *J* = 14.0, 9.6 Hz, 1H), 2.61 (dd, *J* = 17.9, 9.4 Hz, 1H), 1.74 (s, 3H), 1.67 – 1.60 (m, 1H), 1.59 (s, 3H), 1.56 – 1.49 (m, 1H), 1.48 – 1.41 (m, 1H), 0.92 (dd, *J* = 6.5, 3.5 Hz, 3H), 0.89 – 0.85 (m, 3H).
^{13}C NMR (126 MHz, DMSO-*d6*) δ 191.80, 172.33, 171.55, 170.96, 168.47, 137.89, 129.73 (2C), 128.58 (2C), 126.89, 76.94, 54.53, 51.71, 51.69, 49.28, 41.08, 37.65, 36.17, 30.50, 28.14, 26.18, 24.70, 23.43, 22.00.
ESI-LRMS: calcd. for $C_{24}H_{35}N_4O_6S$, $[M+H]^+$: 507.2272 (*m/z*); found $[M+H]^+$: 507.2684.

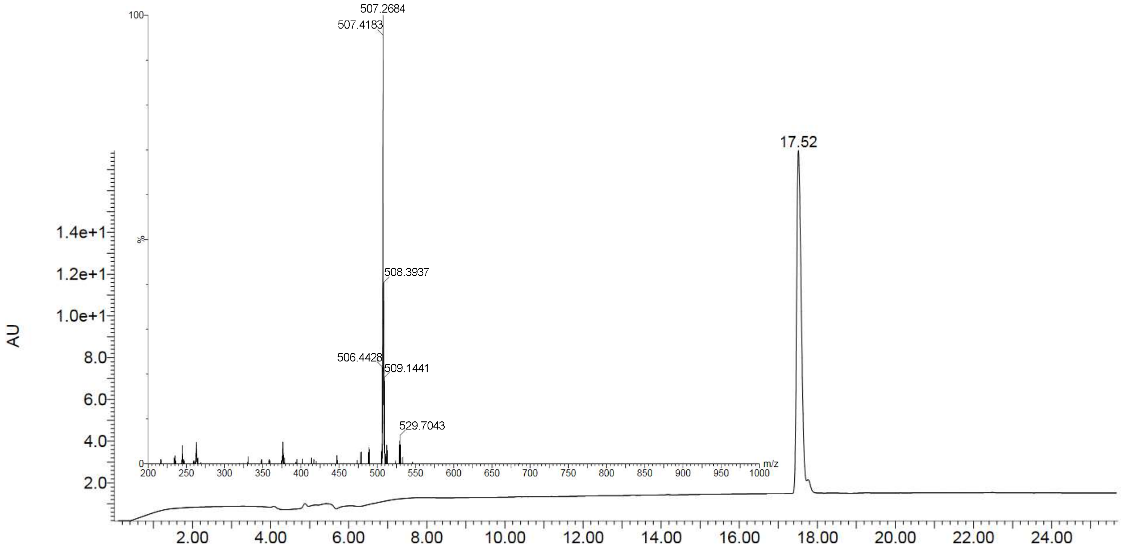

IV.2 preparation of L-cyclo-tetrapeptides from Peptidyl β-thiolactone linear peptides

cyclo-tetrapeptide S10

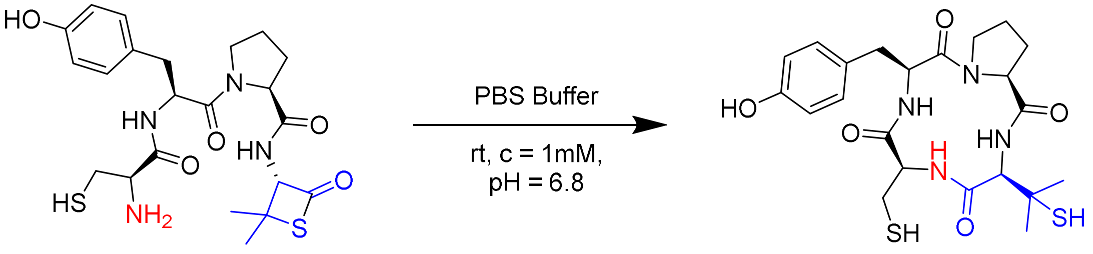

S9 **S10**

Tetrapeptide **S9** (5.0 mg, 0.010 mmol) was dissolved in 10.0 mL PBS buffer (pH = 6.8). After 12h at room temperature, the reaction was done (checked by LC-MS). Purification of the crude peptide using preparative HPLC (20 to 60% solvent B over 20 min, Higgins Analytical Proto 200 5 µm 250 × 10 nm C18 column) afforded peptide **S10** as a white solid after lyophilization (1.0 mg, 20%).

Analytical HPLC: t_R = 12.29 min (30 to 60% solvent B over 20 min, Higgins Analytical Proto 200 5 µm 150 × 2.0 nm C18 column).
ESI-LRMS: calcd. for $C_{22}H_{31}N_4O_5S_2$ Exact Mass: $[M+H]^+$: 495.1730 (*m/z*); found $[M+H]^+$: 495.1625.

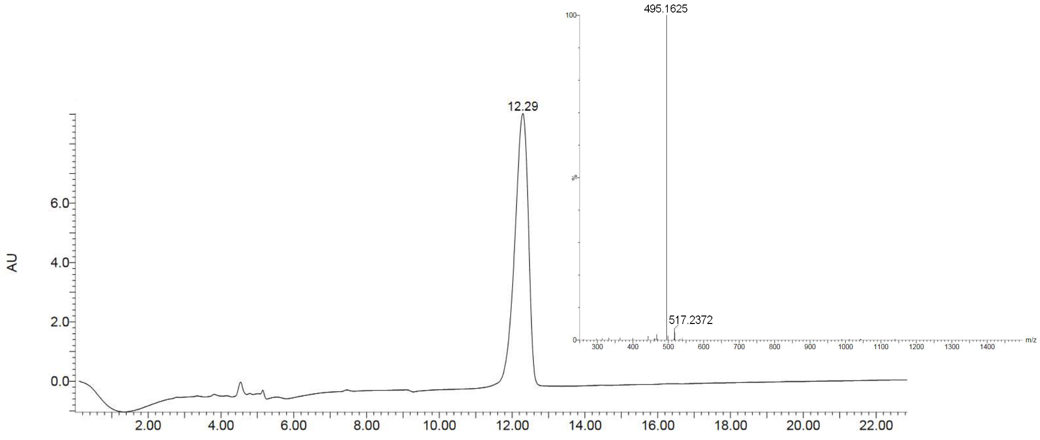

cyclo-tetrapeptide S12

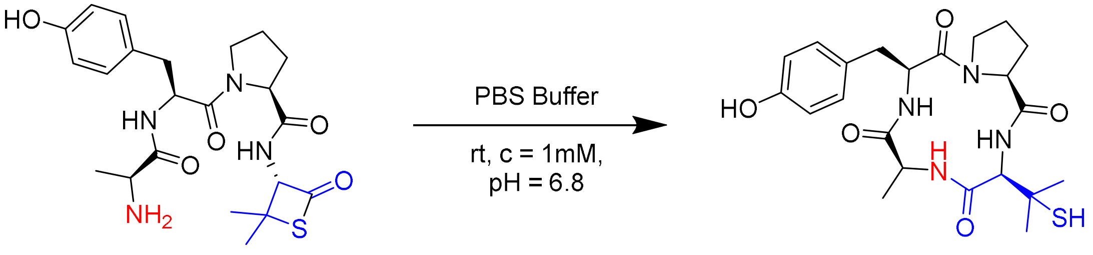

S11 **S12**

Tetrapeptide **S11** (5.0 mg, 0.011 mmol) was dissolved in 11.0 mL PBS buffer (pH = 6.8). After 12h at room temperature, the reaction was complete (checked by LC-MS). Purification of the crude peptide using preparative HPLC (20 to 60% solvent B over 20 min, Higgins Analytical Proto 200 5 µm 250 × 10 nm C18 column) afforded peptide **S12** as a white solid after lyophilization (2.0 mg, 40%).

Analytical HPLC: t_R = 13.81 min (20 to 60% solvent B over 20 min, Higgins Analytical Proto 200 5 µm 150 × 2.0 nm C18 column).
ESI-LRMS: calcd. for $C_{22}H_{31}N_4O_5S$ Exact Mass: $[M+H]^+$: 463.2010 (*m/z*); found $[M+H]^+$: 463.2052.

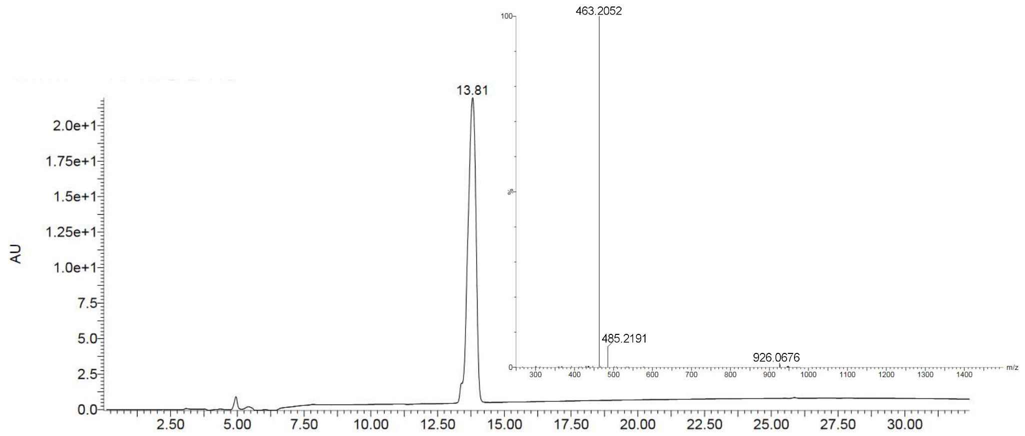

cyclo-tetrapeptides 9a

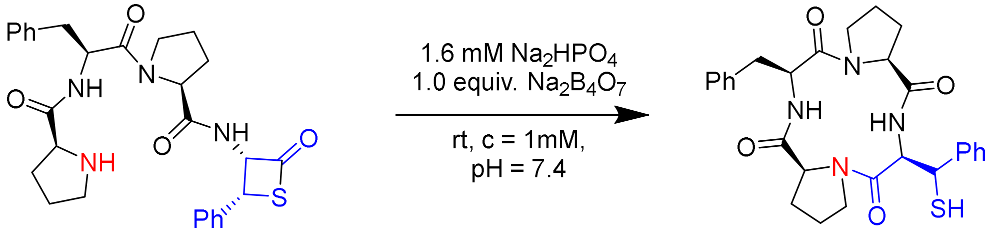

According to the general procedure D, **8a** (5.00 mg, 0.0095 mmol) was dissolved in 9.5 mL cyclization buffer with $Na_2B_4O_7$ (pH = 7.4). After 4h at room temperature, the cyclization reaction was complete (checked by LC-MS). Purification of the crude peptide using preparative HPLC (30 to 90% solvent B over 20 min, Higgins Analytical Proto 200 5 µm 250 × 10 nm C18 column) afforded peptide **9a** as a white solid after lyophilization (2.90 mg, 58%).

Analytical HPLC: t_R = 18.28 min (30 to 60% solvent B over 20 min, Higgins Analytical Proto 200 5 µm 150 × 2.0 nm C18 column).
ESI-LRMS: calcd. for $C_{28}H_{33}N_4O_4S$ Exact Mass: $[M+H]^+$: 521.2223 (*m/z*); found $[M+H]^+$: 521.2644.

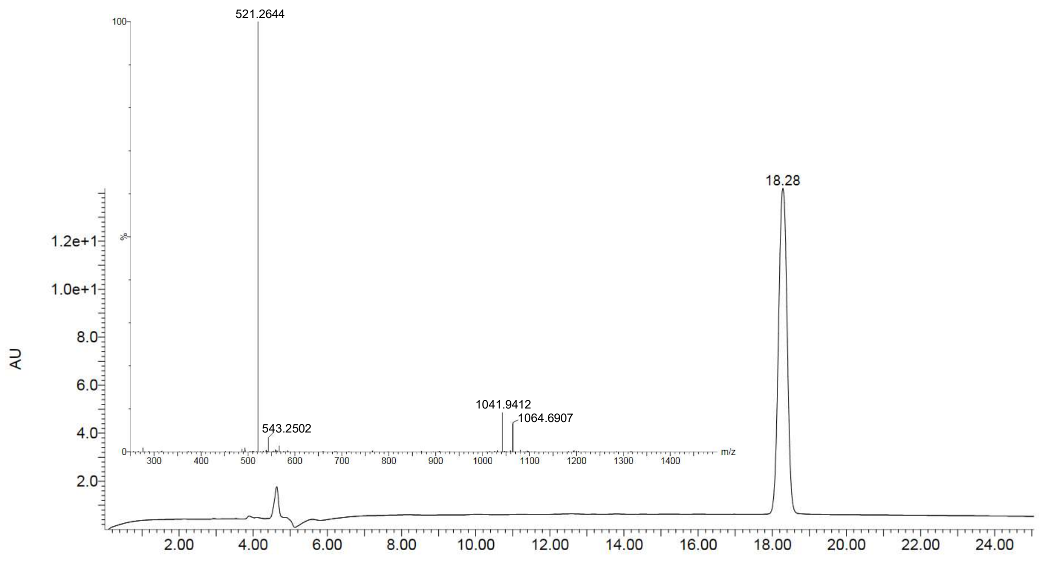

cyclo-tetrapeptides 3

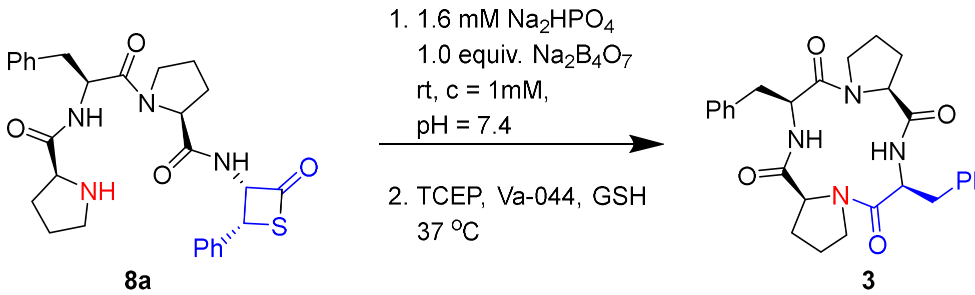

According to the general procedure D and E, **8a** (5.00 mg, 0.0095 mmol) was dissolved in 9.5 mL cyclization buffer with $Na_2B_4O_7$ (pH = 7.4). After the cyclization (4h) and desulfurization (1h) one pot reaction was complete (checked by LC-MS for each step), purification of the crude cyclo-tetrapeptide using preparative HPLC (30 to 90% solvent B over 20 min, Higgins Analytical Proto 200 5 µm 250 × 10 nm C18 column) afforded peptide **3** as a white solid after lyophilization (2.45mg, 49% for two steps). Analytical HPLC: t_R = 7.69 min (50 to 60% solvent B over 20 min, Higgins Analytical Proto 200 5 µm 150 × 2.0 nm C18 column).

Cyclopeptide **3** exists as two conformers in DMSO-*d6* , see ^{1}H spectra in section VII below for details) ^{1}H NMR (600 MHz, DMSO-*d6*) (two conformers are given together) δ 8.07 (d, *J* = 6.9 Hz, 1H), 7.89 (d, *J* = 9.9 Hz, 1H), 7.80 (d, *J* = 10.0 Hz, 1H), 7.22 (t, *J* = 7.5 Hz, 1H), 7.20 – 7.16 (m, 3H), 7.16 – 7.11 (m, 5H), 7.06 (d, *J* = 7.2 Hz, 1H), 4.96 – 4.86 (m, 2.5H), 4.07 (d, *J* = 8.0 Hz, 1H), 4.04 (d, *J* = 9.2 Hz, 1H), 3.90 (d, *J* = 7.7 Hz, 1H), 3.89 – 3.86 (m, 1H), 3.52 (s, 1H), 3.42 (dt, *J* = 18.8, 9.4 Hz, 2H), 3.13 – 3.03 (m, 4H), 2.77 (dd, *J* = 13.9, 10.1 Hz, 1H), 2.64 (dd, *J* = 14.0, 9.9 Hz, 2H), 1.95 – 1.83 (m, 2H), 1.81 – 1.69 (m, 4H), 1.60 (dd, *J* = 11.4, 5.8 Hz, 3H), 1.53 (d, *J* = 9.9 Hz, 1H), 1.48 (d, *J* = 12.6 Hz, 2H).

^{1}H NMR (500 MHz, MeOH-*d4*) (single asymmetric conformer) δ 7.30 (t, *J* = 7.5 Hz, 2H), 7.23 (dt, *J* = 12.0, 7.4 Hz, 5H), 7.16 (dd, *J* = 13.8, 7.0 Hz, 3H), 5.18 (dd, *J* = 10.5, 5.0 Hz, 1H), 4.12 (d, *J* = 8.9 Hz, 1H), 4.05 (d, *J* = 7.1 Hz, 1H), 3.99 (dd, *J* = 11.1, 4.6 Hz, 1H), 3.67 (dd, *J* = 21.4, 14.1 Hz, 2H), 3.59 – 3.50 (m, 1H), 3.48 – 3.39 (m, 2H), 3.19 – 3.15 (m, 1H), 2.86 (dd, *J* = 14.2, 10.5 Hz, 1H), 2.08 – 1.97 (m, 2H), 1.97

– 1.87 (m, 2H), 1.73 (d, J = 5.9 Hz, 1H), 1.69 (d, J = 7.1 Hz, 1H), 1.58 – 1.52 (m, 1H), 1.18 (dd, J = 12.3, 6.6 Hz, 1H), 0.91 (d, J = 6.8 Hz, 1H).

Symmetric minor conformer ^{13}C NMR (151 MHz, DMSO-*d6*) δ 171.29 (2C), 170.54(2C), 138.46 (2C), 130.00 (4C), 128.12 (4C), 126.35 (2C), 61.45 (2C), 51.75 (2C), 47.02 (2C), 38.01 (2C), 31.29 (2C), 21.23 (2C);

Asymmetric major conformer ^{13}C NMR (151 MHz, DMSO-*d6*) δ 172.09, 171.64, 170.99, 168.77, 139.83, 139.02, 130.17 (2C), 129.81 (2C), 128.38 (2C), 127.88 (2C), 126.35, 126.11, 62.46, 62.25, 58.73, 58.30, 55.38, 48.17, 47.80, 37.52, 35.56, 31.76, 31.30, 21.75.

ESI-LRMS: calcd. for $C_{28}H_{33}N_4O_4$ Exact Mass: $[M+H]^+$: 489.2496 (*m/z*); found $[M+H]^+$: 489.2342.

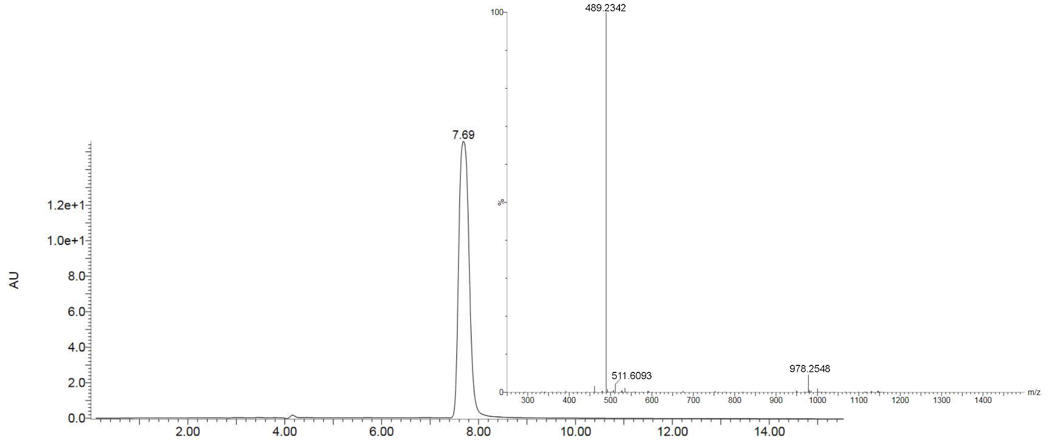

cyclo-tetrapeptides 6

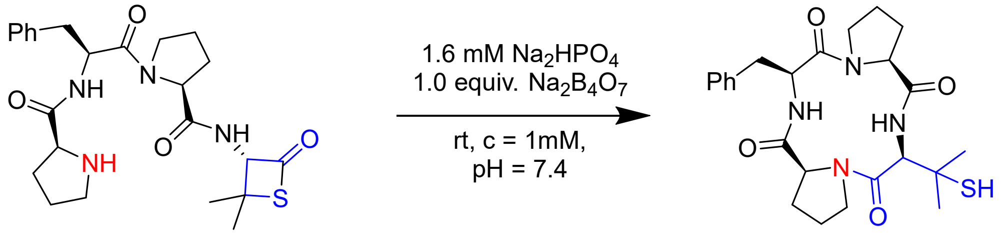

5 **6**

According to the general procedure D, **5** (5.00 mg, 0.011 mmol) was dissolved in 10.5 mL cyclization buffer with $Na_2B_4O_7$ (pH = 7.4). After 4h at room temperature, the cyclization reaction was complete (checked by LC-MS). Purification of the crude peptide using preparative HPLC (20 to 60% solvent B over 20 min, Higgins Analytical Proto 200 5 µm 250 × 10 nm C18 column) afforded peptide **6** as a white solid after lyophilization (3.15 mg, 63%).

Analytical HPLC: t_R = 16.79 min (20 to 80% solvent B over 20 min, Higgins Analytical Proto 200 5 µm 150 × 2.0 nm C18 column).
^{1}H NMR (500 MHz, DMSO-d_6) δ 8.12 (d, J = 9.6 Hz, 1H), 8.05 (d, J = 9.5 Hz, 1H), 7.16 (q, J = 8.6, 7.9 Hz, 5H), 4.96 (d, J = 8.0 Hz, 1H), 4.57 (dd, J = 9.5, 2.4 Hz, 1H), 4.26 (d, J = 6.2 Hz, 1H), 4.07 (d, J = 8.0 Hz, 1H), 3.52 (d, J = 9.5 Hz, 1H), 3.40 (d, J = 10.0 Hz, 1H), 3.23 (t, J = 10.3 Hz, 1H), 3.11 (d, J = 12.7 Hz, 2H), 3.01 (s, 1H), 2.69 – 2.59 (m, 1H), 2.00 (d, J = 8.1 Hz, 2H), 1.93 – 1.87 (m, 1H), 1.80 – 1.75 (m, 1H), 1.57

(dt, J = 11.6, 5.5 Hz, 3H), 1.34 (s, 3H), 1.23 (s, 3H), 1.12 (q, J = 11.6, 10.7 Hz, 2H).
^{13}C NMR (126 MHz, DMSO-d_6) δ 171.83, 171.69, 170.31, 168.90, 138.52, 130.03, 128.17, 126.39, 61.54, 61.44, 57.98, 51.60, 47.23, 46.76, 46.72, 38.02, 32.09, 31.40, 30.59, 22.18, 20.95.
ESI-LRMS: calcd. For Exact Mass: $C_{24}H_{33}N_4O_4S$ [M+H]$^+$: 473.2217 (*m/z*); found [M+H]$^+$: 473.2491.

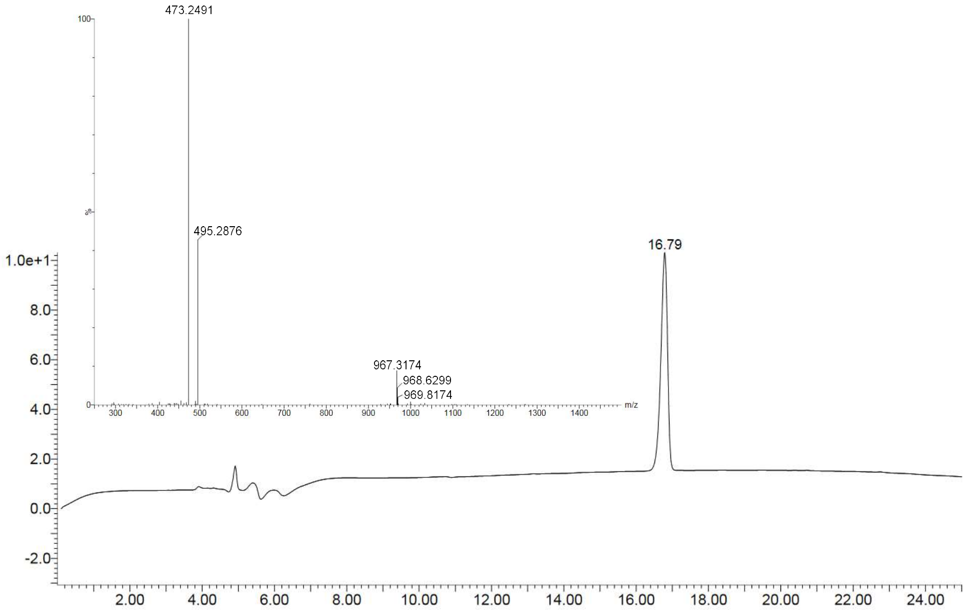

cyclo-tetrapeptides 11b

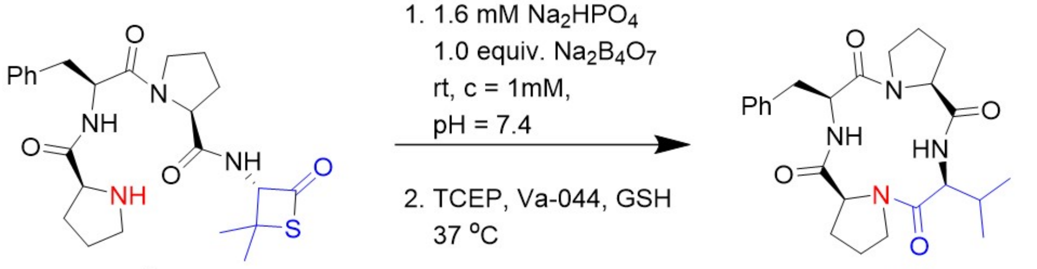

5 **11b**

According to the general procedure D and E, **5** (5.00 mg, 0.011 mol) was dissolved in 10.5 mL cyclization buffer with $Na_2B_4O_7$ (pH = 7.4). After the cyclization (4h) and desulfurization (1h) one pot reaction was complete (checked by LC-MS for each step), purification of the crude cyclo-tetrapeptide using preparative HPLC (20 to 60% solvent B over 20 min, Higgins Analytical Proto 200 5 µm 250 × 10 nm C18 column) afforded peptide **11b** as a white solid after lyophilization (2.75 mg, 55% for two steps).

Analytical HPLC: t_R = 13.38 min (20 to 80% solvent B over 20 min, Higgins Analytical Proto 200 5 µm 150 × 2.0 nm C18 column).
^{1}H NMR (600 MHz, DMSO-*d*6) δ 8.01 – 8.00 (d, J = 9.8 Hz, 1H), 7.82 – 7.80 (d, J = 9.7Hz, 1H), 7.19 – 7.12 (m, 5H), 4.98 – 4.94 (td, J = 5.1, 7.6 Hz, 1H), 4.39 – 4.37 (dd, J = 4.7, 9.6 Hz, 1H), 4.24 – 4.23 (d, J = 7.6 Hz, 1H), 4.02 – 4.00 (d, J = 8.1 Hz, 1H), 3.53 – 3.48 (m, 1H), 3.39 – 3.34 (m, 2H), 3.21 – 3.17 (m, 1H), 3.12 – 3.11 (m, 1H), 3.10 – 3.05 (m, 1H), 2.66 – 2.62 (dd, J = 10.0, 14.1 Hz, 1H), 2.10 – 2.07 (m, 1H), 2.05 – 2.02 (m, 1H), 1.96 – 1.92 (m, 1H), 1.91 – 1.85 (m, 1H), 1.79 – 1.72 (m, 1H), 1.60 – 1.52 (m, 2H), 1.14 – 1.11 (m, 1H), 0.80 – 0.79 (d, J = 6.7 Hz, 3H), 0.77 – 0.76 (d, J = 6.7 Hz, 3H).

^{13}C NMR (151 MHz, DMSO-*d6*) δ 171.55, 171.45, 170.74, 170.68, 138.49, 129.99 (2C), 128.13 (2C), 126.36, 61.64, 61.38, 55.13, 51.46, 47.10, 46.73, 37.96, 31.84, 31.30, 29.12, 22.04, 21.19, 20.97, 17.93.
ESI-LRMS: calcd. for Exact Mass: $C_{24}H_{33}N_4O_4$ $[M+H]^+$: 441.2496 (*m/z*); found $[M+H]^+$: 441.2266.

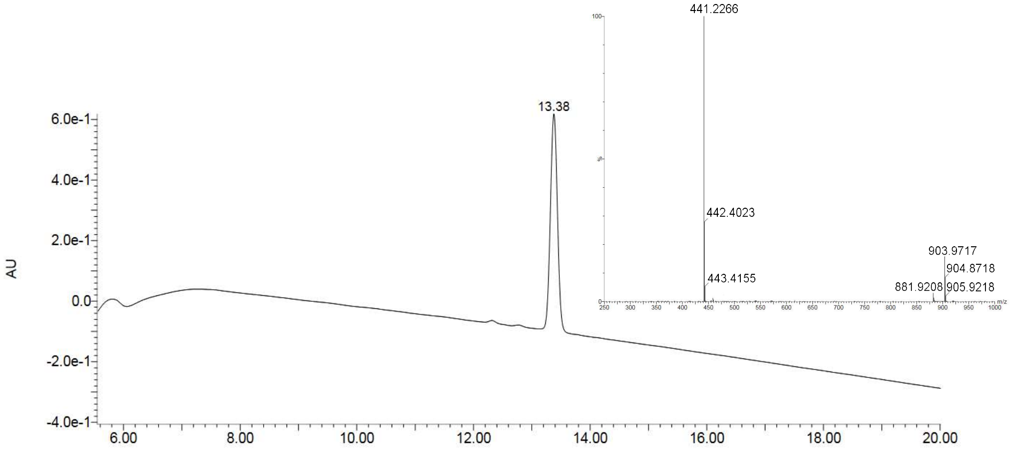

cyclo-tetrapeptides 9c

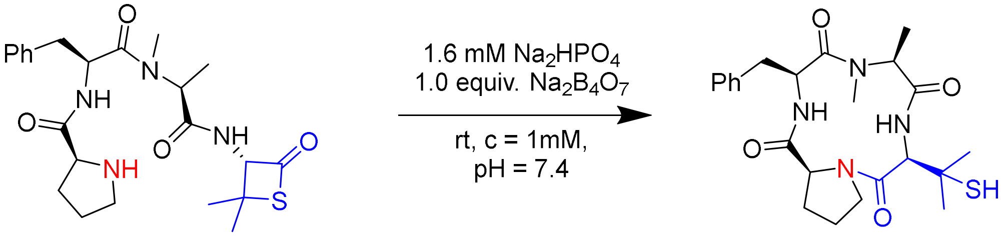

8c **9c**

According to the general procedure D, **8c** (5.00 mg, 0.011 mmol) was dissolved in 10.8 mL cyclization buffer with $Na_2B_4O_7$ (pH = 7.4). After 6h at room temperature, the cyclization reaction was complete (checked by LC-MS). Purification of the crude peptide using preparative HPLC (20 to 60% solvent B over 20 min, Higgins Analytical Proto 200 5 µm 250 × 10 nm C18 column) afforded peptide **9c** as a white solid after lyophilization (3.65 mg, 73%).
Analytical HPLC: t_R = 16.61 min (30 to 70% solvent B over 20 min, Higgins Analytical Proto 200 5 µm 150 × 2.0 nm C18 column).
ESI-LRMS: calcd. for Exact Mass: $C_{23}H_{33}N_4O_4S$ $[M+H]^+$: 461.2217 (*m/z*); found $[M+H]^+$: 461.2165.

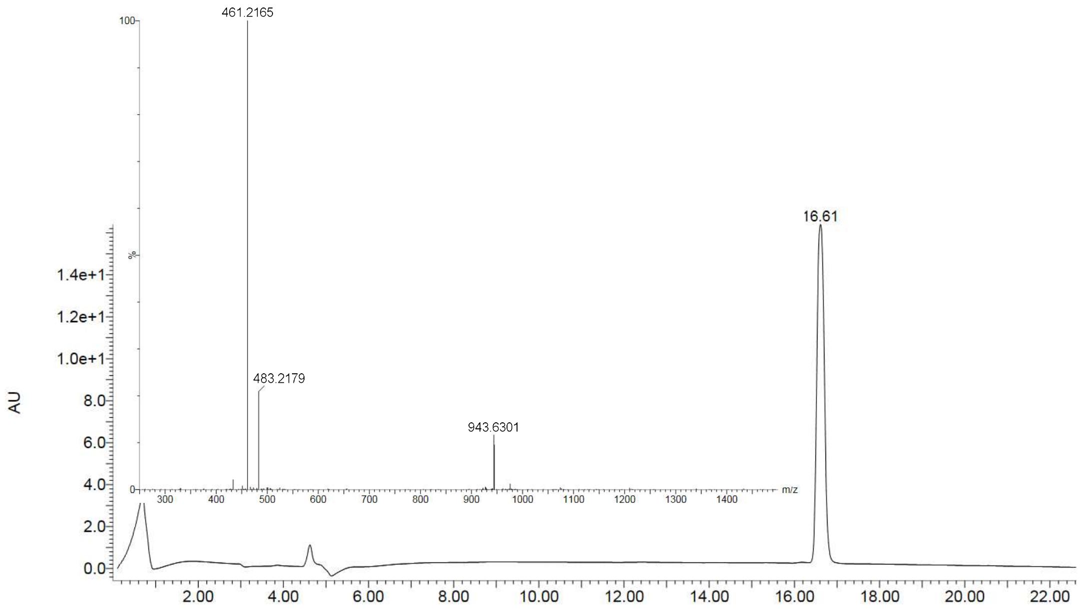

cyclo-tetrapeptides 15n

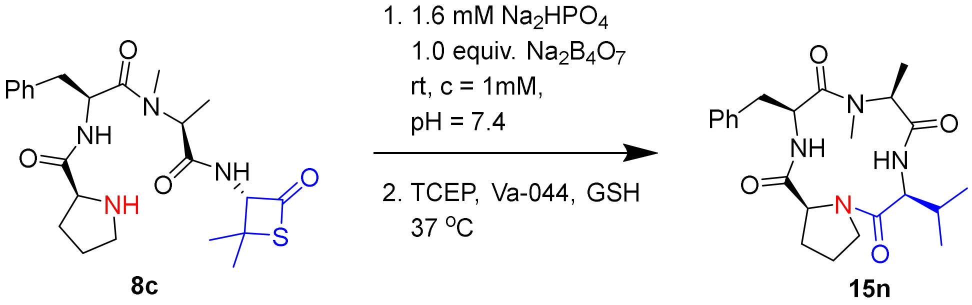

According to the general procedure D and E, **8c** (5.00 mg, 0.011 mmol) was dissolved in 10.8 mL cyclization buffer with $Na_2B_4O_7$ (pH = 7.4). After the cyclization (4h) and desulfurization (1h) one pot reaction was complete (checked by LC-MS for each step), purification of the crude cyclo-tetrapeptide using preparative HPLC (20 to 60% solvent B over 20 min, Higgins Analytical Proto 200 5 µm 250 × 10 nm C18 column) afforded peptide **15n** as a white solid after lyophilization (2.8 mg, 56% for two steps).

Analytical HPLC: t_R = 13.08 min (30 to 70% solvent B over 20 min, Higgins Analytical Proto 200 5 µm 150 × 2.0 nm C18 column).

^{1}H NMR (600 MHz, DMSO-*d6*) δ 8.04 – 8.03 (d, *J* = 9.9 Hz, 1H), 7.77 – 7.76 (d, *J* = 9.3 Hz, 1H), 7.19 – 7.17 (m, 4H), 7.13 – 7.12 (d, *J* = 6.4 Hz, 1H), 5.10 – 5.06 (td, *J* = 9.9, 4.6 Hz, 1H), 4.35 – 4.33 (dd, *J* = 9.0, 4.3 Hz, 1H), 4.21 – 4.20 (dd, *J* = 13.6, 6.6 Hz, 1H), 3.94 – 3.93 (d, *J* = 8.0 Hz, 1H), 3.42 – 3.37 (dd, *J* = 18.7, 10.9 Hz, 2H), 3.12 – 3.10 (dd, *J* = 14.1, 4.2 Hz, 1H), 3.09 – 3.06 (m, 1H), 2.67 (s, 3H), 2.45 – 2.36 (m, 1H), 2.09 – 2.07 (dd, *J* = 11.4, 6.1 Hz, 1H), 1.79 – 1.70 (m, 1H), 1.62 – 1.55 (m, 1H), 1.52 – 1.49 (dd, *J* = 11.7, 6.3 Hz, 1H), 1.33 – 1.32 (d, *J* = 6.9 Hz, 3H), 0.83 – 0.82 (d, *J* = 6.7 Hz, 3H), 0.81 – 0.80 (d, *J* = 6.8 Hz, 3H).

^{13}C NMR (126 MHz, DMSO-*d6*) δ 172.30, 171.04, 170.88, 170.63, 138.54, 129.97 (2C), 128.12 (2C), 126.39, 61.33, 56.00, 55.47, 50.65, 46.75, 38.21, 31.36, 30.58, 29.32, 21.14, 20.92, 18.03, 15.96.

ESI-LRMS: calcd. for Exact Mass: $C_{23}H_{33}N_4O_4$ $[M+H]^+$: 429.2496 (*m/z*); found $[M+H]^+$: 429.2937.

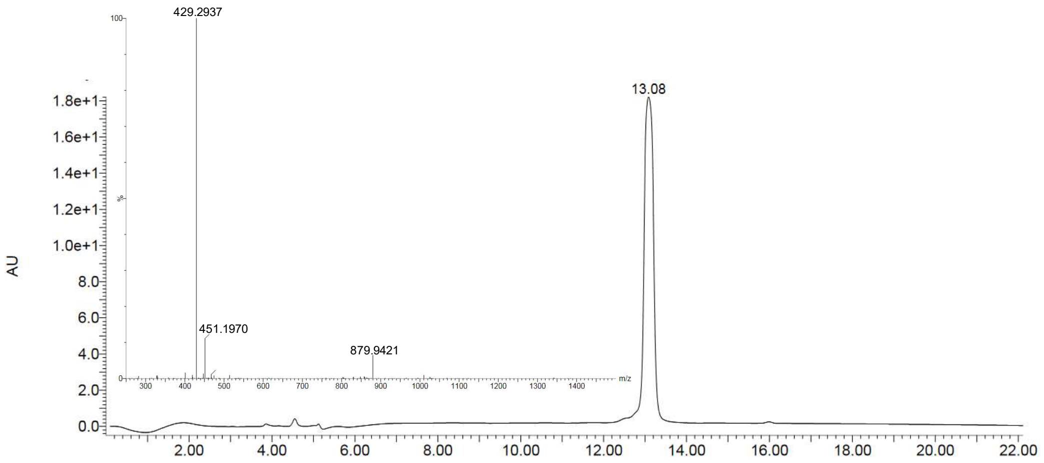

cyclo-tetrapeptides 9d

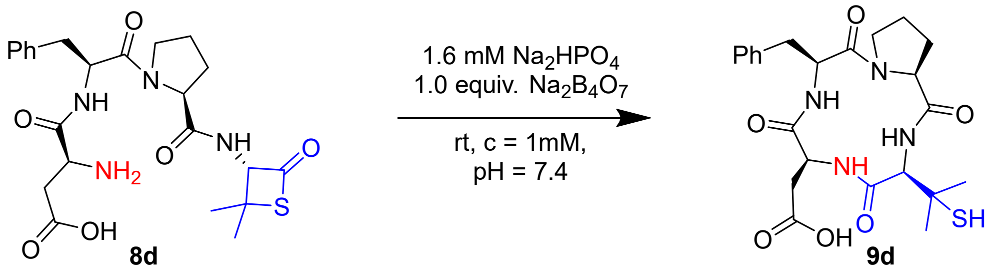

8d **9d**

According to the general procedure E, **8d** (5.00 mg, 0.010 mmol) was dissolved in 10.1 mL cyclization buffer with $Na_2B_4O_7$ (pH = 7.4). After 6h at room temperature, the cyclization reaction was complete (checked by LC-MS). Purification of the crude peptide using preparative HPLC (30 to 60% solvent B over 20 min, Higgins Analytical Proto 200 5 µm 250 × 10 nm C18 column) afforded peptide **9d** as a white solid after lyophilization (2.4 mg, 48%).

Analytical HPLC: t_R = 12.05 min (30 to 60% solvent B over 20 min, Higgins Analytical Proto 200 5 µm 150 × 2.0 nm C18 column).
ESI-LRMS: calcd. for Exact Mass: $C_{23}H_{31}N_4O_6S$ $[M+H]^+$: 491.1959 (*m/z*); found $[M+H]^+$: 491.2227.

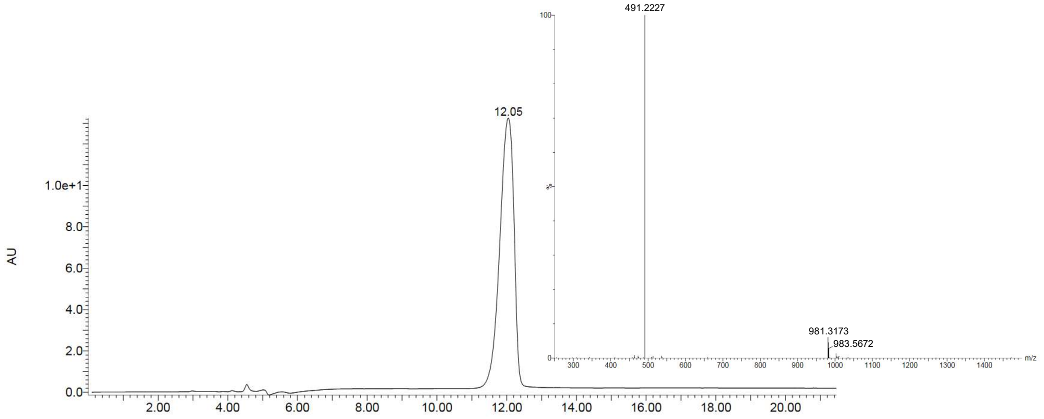

cyclo-tetrapeptides 15r

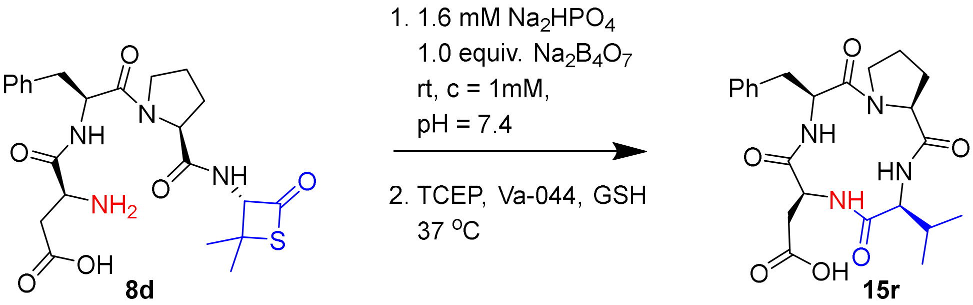

According to the general procedure D and E, **8d** (5.00 mg, 0.010 mmol) was dissolved in 10.1 mL cyclization buffer with $Na_2B_4O_7$ (pH = 7.4). After the cyclization (6h) and desulfurization (1h) one pot reaction was complete (checked by LC-MS for each step), purification of the crude cyclo-tetrapeptide using preparative HPLC (30 to 60% solvent B over 20 min, Higgins Analytical Proto 200 5 µm 250 × 10 nm C18 column) afforded peptide **15r** as a white solid after lyophilization (1.95 mg, 42% for two steps).

Analytical HPLC: t_R = 9.28 min (30 to 60% solvent B over 20 min, Higgins Analytical Proto 200 5 µm 150 × 2.0 nm C18 column).
^{1}H NMR (500 MHz, DMSO-*d6*) δ 12.28 (s, 1H), 8.30 (d, *J* = 9.0 Hz, 1H), 8.05 (s, 1H), 7.52 (d, *J* = 8.9 Hz, 1H), 7.20 (d, *J* = 5.8 Hz, 4H), 7.15 (dd, *J* = 10.2, 6.8 Hz, 1H), 4.76 (s, 1H), 4.35 (s, 1H), 4.17 (s, 1H), 4.03 (d, *J* = 7.9 Hz, 1H), 3.47 (s, 1H), 3.42 (s, 1H), 3.27 – 3.21 (m, 1H), 3.15 (dd, *J* = 13.7, 5.2 Hz, 1H), 2.59 (s, 1H), 2.39 (dd, *J* = 17.0, 10.8 Hz, 1H), 2.15 – 2.03 (m, 1H), 1.99 (s, 2H), 1.90 (dd, *J* = 16.5, 8.8 Hz, 1H), 1.55 (s, 1H), 0.82 (t, *J* = 7.7 Hz, 6H).
^{13}C NMR (151 MHz, DMSO-*d6*) δ 172.73, 172.45, 172.11, 170.98, 169.97, 138.76, 130.15 (2C), 128.17 (2C), 126.27, 61.78, 54.06, 53.35, 52.72, 47.26, 37.90, 36.68, 32.10, 28.71, 22.04, 20.88, 18.21.
ESI-LRMS: calcd. for Exact Mass: $C_{23}H_{31}N_4O_6$ $[M+H]^+$: 459.2238, (*m/z*); found $[M+H]^+$: 459.2025.

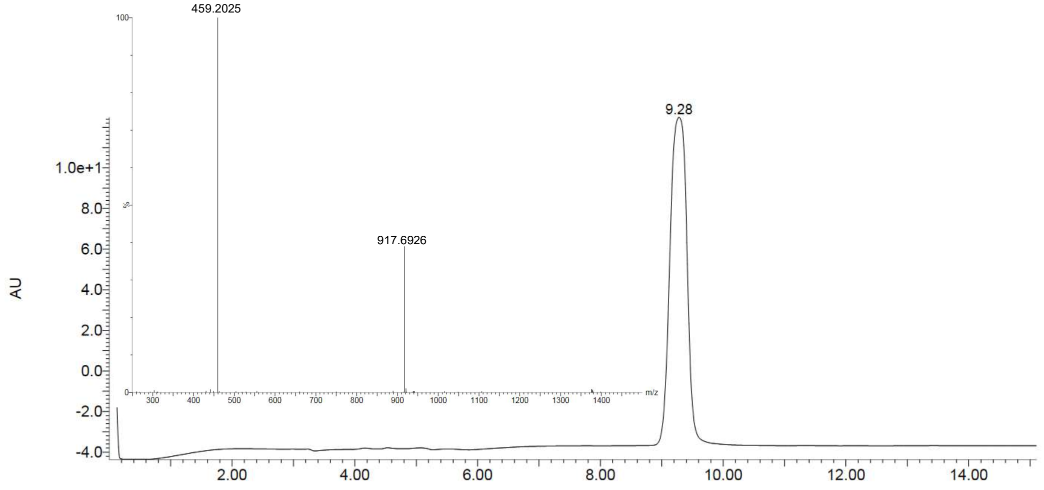

cyclo-tetrapeptides 9e

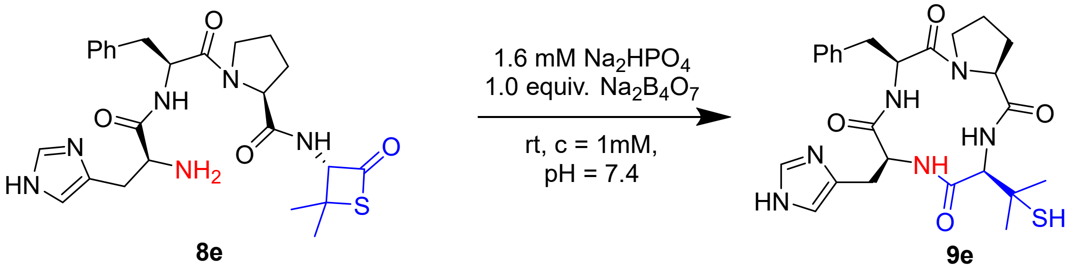

According to the general procedure E, **8e** (5.00 mg, 0.010 mmol) was dissolved in 9.7 mL cyclization buffer with $Na_2B_4O_7$ (pH = 7.4). After 4h at room temperature, the cyclization reaction was done (checked by Lc-MS). Purification of the crude peptide using preparative HPLC (20 to 50% solvent B over 20 min, Higgins Analytical Proto 200 5 µm 250 × 10 nm C18 column) afforded peptide **9e** as a white solid after lyophilization (3.15 mg, 63%).

Analytical HPLC: t_R = 12.43 min (20 to 60% solvent B over 20 min, Higgins Analytical Proto 200 C18 5 µm 150 × 2.0 nm column);

^1H NMR (500 MHz, DMSO-d_6) δ 8.98 (s, 1H), 8.42 (d, *J* = 9.6 Hz, 1H), 8.21 (d, *J* = 9.3 Hz, 1H), 7.73 (d, *J* = 9.1 Hz, 1H), 7.23 (t, *J* = 5.5 Hz, 5H), 7.17 (dt, *J* = 8.6, 4.1 Hz, 1H), 4.82 (s, 1H), 4.56 (d, *J* = 9.3 Hz, 1H), 4.25 (d, *J* = 7.5 Hz, 1H), 4.01 (s, 1H), 3.29 (s, 1H), 3.18 (dd, *J* = 13.9, 4.9 Hz, 1H), 2.99 (d, *J* = 15.5 Hz, 1H), 2.95 (s, 1H), 2.73 (t, *J* = 13.4 Hz, 1H), 2.68 – 2.56 (m, 1H), 2.02 (d, *J* = 21.5 Hz, 2H), 1.95 – 1.86 (m, 1H), 1.59 (d, *J* = 13.5 Hz, 1H), 1.27 (s, 6H).

^{13}C NMR (126 MHz, DMSO) δ 172.44, 171.30, 168.87, 138.53, 134.35, 130.28, 128.21, 126.41, 117.43, 61.58, 56.48, 55.35, 52.13, 47.33, 46.06, 38.14, 32.26, 30.31, 29.00, 27.21, 22.18.

ESI-LRMS: calcd. for Exact Mass: $C_{25}H_{33}N_6O_4S$ $[M+H]^+$: 513.2279 (*m/z*); found $[M+H]^+$: 513.2227.

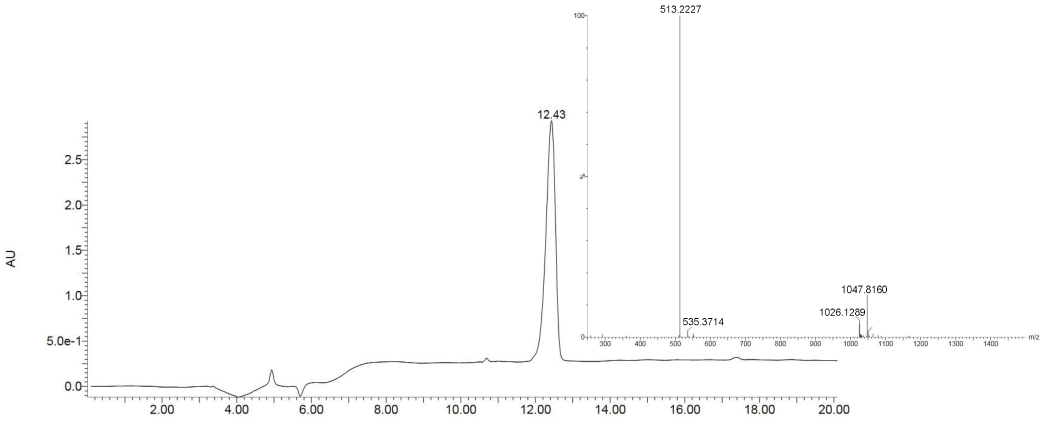

cyclo-tetrapeptides 8f

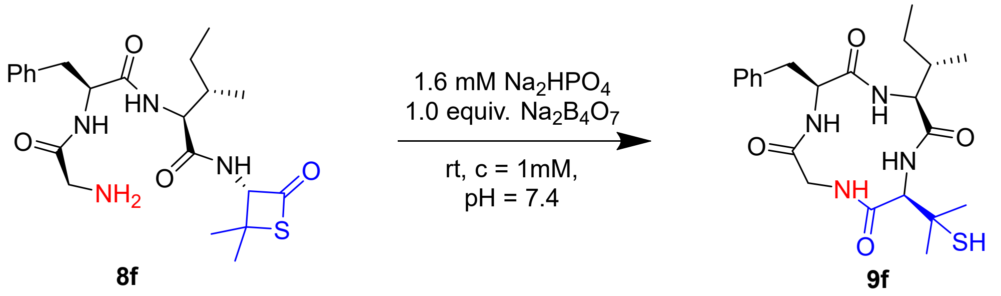

According to the general procedure E, **8f** (5.00 mg, 0.011 mmol) was dissolved in 10.0 mL cyclization buffer with $Na_2B_4O_7$ (pH = 7.4). After 8h at room temperature, the cyclization reaction was complete (checked by LC-MS). Purification of the crude peptide using preparative HPLC (20 to 60% solvent B over 20 min, Higgins Analytical Proto 200 5 µm 250 × 10 nm C18 column) afforded peptide **9f** as a white solid after lyophilization (2.7 mg, 54%).

Analytical HPLC: t_R = 18.53 min (30 to 80% solvent B over 20 min, Higgins Analytical Proto 200 5 µm 150 × 2.0 nm C18 column).
^1H NMR (600 MHz, DMSO-*d6*) δ 8.77 – 8.75 (q, *J* = 4.6 Hz, 1H), 8.07 – 8.06 (d, *J* = 9.1 Hz, 1H), 7.42 – 7.40 (d, *J* = 9.6 Hz, 1H), 7.28 – 7.26 (m, 2H), 7.21 – 7.18 (m, 3H), 6.86 – 6.85 (d, *J* = 9.8 Hz, 1H), 4.49 – 4.45 (q, *J* = 9.1 Hz, 1H), 4.32 – 4.30 (d, *J* = 9.6 Hz, 1H), 4.22 – 4.28 (dd, *J* = 8.0, 13.8 Hz, 1H), 3.68 – 3.65 (t, *J* = 10.5 Hz, 1H), 3.13 – 3.10 (dd, *J* = 4.6, 13.8 Hz, 1H), 3.02 – 2.98 (dd, *J* = 9.1, 14.0 Hz, 1H), 2.93 – 2.89 (dd, *J* = 7.8, 14.0 Hz, 1H), 2.89 (s, 1H), 1.96 (m, 1H), 1.43 – 1.39 (m, 1H), 1.34 (s, 3H), 1.33 (s, 3H), 1.05 – 1.01 (m, 1H), 0.84 – 0.83 (d, *J* = 6.7 Hz, 3H), 0.79 – 0.76 (t, *J* = 7.4 Hz, 3H).
^{13}C NMR (151 MHz, DMSO-*d6*) δ 173.29, 172.19, 171.73, 170.36, 137.28, 129.24 (2C), 128.77 (2C), 127.06, 62.39, 61.67, 56.94, 44.98, 43.81, 36.58, 34.31, 30.77, 28.89, 25.50, 15.94, 10.27.
ESI-LRMS: calcd. for Exact Mass: $C_{22}H_{33}N_4O_4S$ $[M+H]^+$: 449.2217 (*m/z*); found $[M+H]^+$: 449.2156.

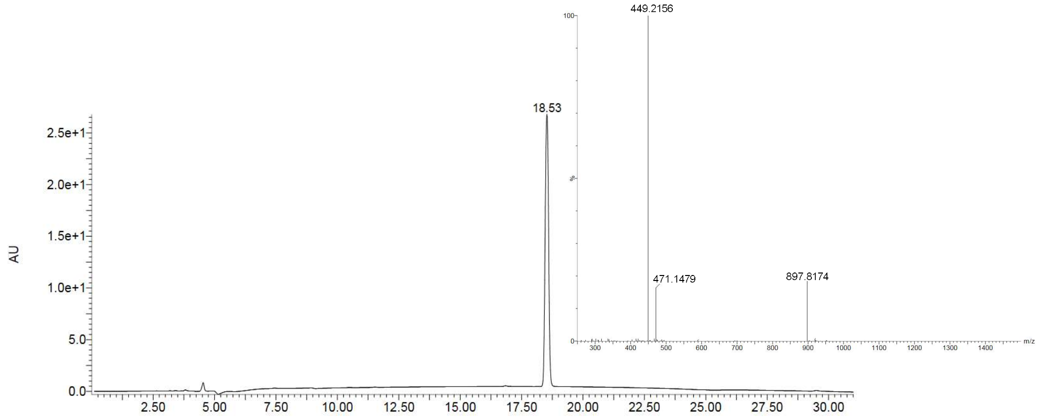

cyclo-tetrapeptides 9g

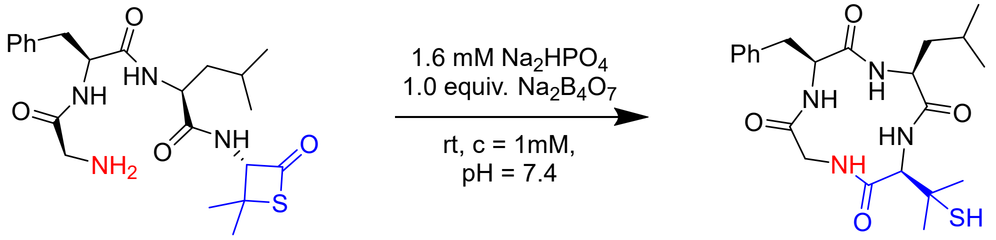

According to the general procedure E, **8g** (5.00 mg, 0.011 mmol) was dissolved in 11.1 mL cyclization buffer with $Na_2B_4O_7$ (pH = 7.4). After 8h at room temperature, the cyclization reaction was complete (checked by LC-MS). Purification of the crude peptide using preparative HPLC (30 to 80% solvent B over 20 min, Higgins Analytical Proto 200 5 µm 250 × 10 nm C18 column) afforded peptide **9g** as a white solid after lyophilization (2.35 mg, 47%).

Analytical HPLC: t_R = 17.92 min (30 to 80% solvent B over 20 min, Higgins Analytical Proto 200 5 µm 150 × 2.0 nm C18 column).

ESI-LRMS: calcd. for Exact Mass: $C_{21}H_{31}N_4O_4S$ $[M+H]^+$: 449.2217 (*m/z*); found $[M+H]^+$: 449.2330.

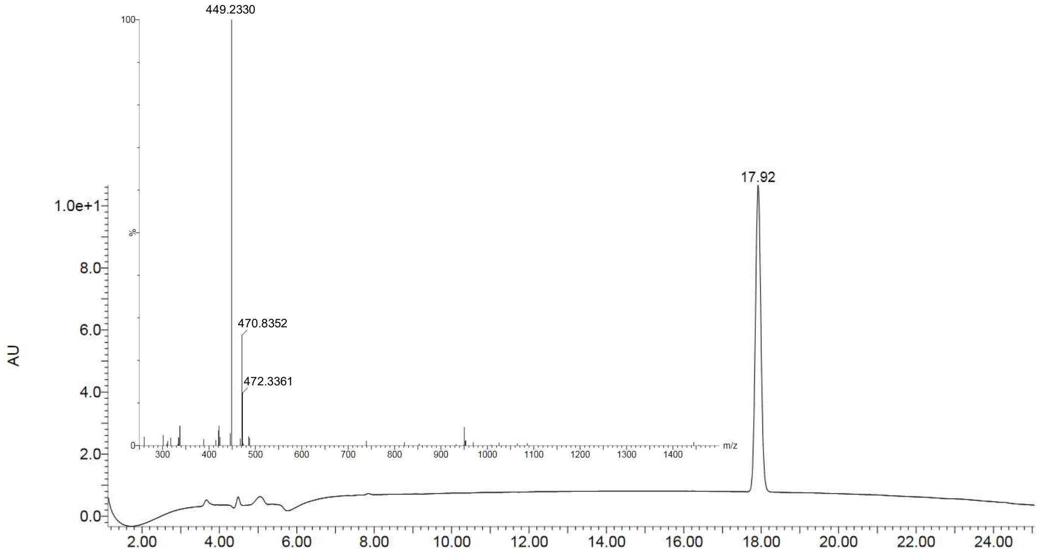

cyclo-tetrapeptides 15s

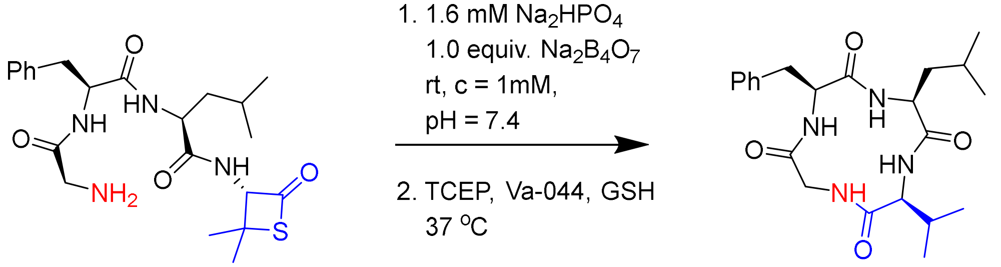

8g **15s**

According to the general procedure D and E, **8g** (5.00 mg, 0.011 mmol) was dissolved in 11.1 mL cyclization buffer with $Na_2B_4O_7$ (pH = 7.4). After the cyclization (8h), one-pot desulfurization could not completely consume the starting material after 4 hours, at this point, the reaction was stopped (checked by Lc-MS for each step), purification of the crude cyclo-tetrapeptide using preparative HPLC (30 to 80% solvent B over 20 min, Higgins Analytical Proto 200 5 µm 250 × 10 nm C18 column) afforded peptide **15s** as a white solid after lyophilization (0.95 mg, 20% over two steps with 0.22 mg, 22% **8g** recovered).

Analytical HPLC: t_R = 15.36 min (30 to 80% solvent B over 20 min, Higgins Analytical Proto 200 5 µm 150 × 2.0 nm C18 column).

^{1}H NMR (600 MHz, DMSO-*d6*) δ 8.56 (s, 1H), 8.21 – 7.85 (m, 1H), 7.65 (s, 1H), 7.29 – 7.23 (m, 2H), 7.20 – 7.18 (m, 4H), 4.42 (d, *J* = 8.1 Hz, 1H), 4.08 (dd, *J* = 15.9, 8.7 Hz, 1H), 3.89 – 3.71 (m, 2H), 3.41 – 3.37 (m, 1H), 2.98 (d, *J* = 7.9 Hz, 1H), 2.95 – 2.83 (m, 1H), 1.95 (s, 1H), 1.61 (s, 1H), 1.57 (dd, *J* = 15.0, 8.6 Hz, 1H), 1.54 – 1.42 (m, 2H), 0.90 (d, *J* = 6.2 Hz, 3H), 0.84 (d, *J* = 6.5 Hz, 3H), 0.82 (d, *J* = 6.6 Hz, 3H), 0.80 (d, *J* = 6.2 Hz, 3H).

^{13}C NMR (151 MHz, DMSO-*d6*) δ 173.44, 172.96, 171.59, 170.20, 137.58, 129.25 (2C), 128.74 (2C), 128.95, 61.51, 56.99, 54.77, 43.93, 36.51, 29.50, 29.12, 25.22, 22.98, 22.09, 19.64, 19.35.

ESI-LRMS: calcd. for Exact Mass: $C_{22}H_{33}N_4O_4$ $[M+H]^+$: 417.2496 (*m/z*); found $[M+H]^+$: 417.2465.

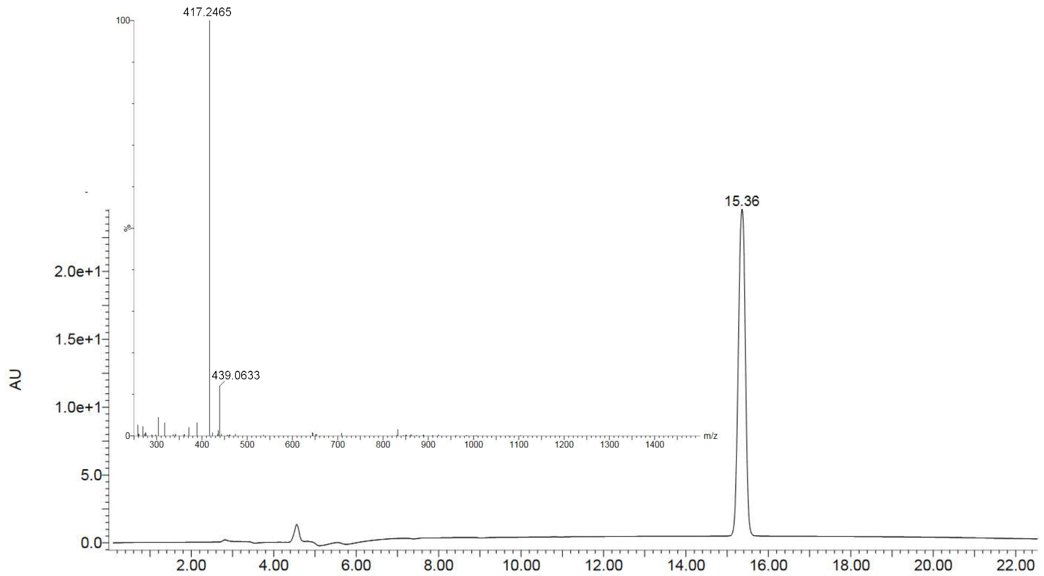

cyclo-tetrapeptide 9h

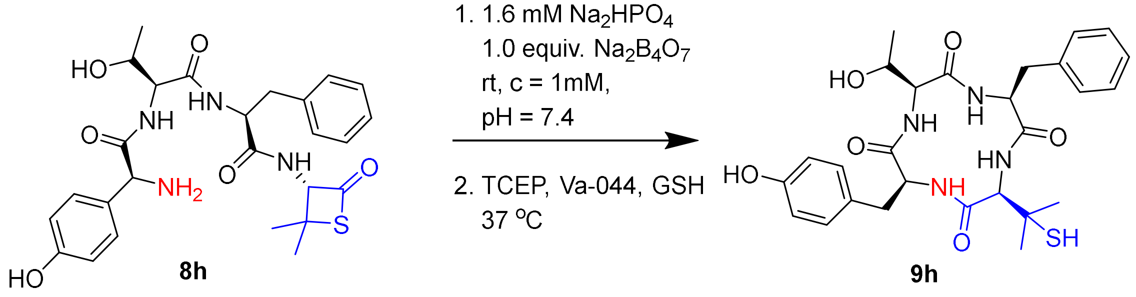

Tetrapeptide **8h** (5.0 mg, 0.092 mmol) was dissolved in 9.2 mL PBS buffer (pH = 7.1). After 12h at room temperature, the reaction was complete (checked by LC-MS). Purification of the crude peptide using preparative HPLC (20 to 60% solvent B over 20 min, Higgins Analytical Proto 200 5 µm 250 × 10 nm C18 column) afforded peptide **9h** as a white solid after lyophilization (2.0 mg, 40%).

Analytical HPLC: t_R = 10.14 min (20 to 60% solvent B over 20 min, Higgins Analytical Proto 200 5 µm 150 × 2.0 nm C18 column).

^{1}H NMR (500 MHz, DMSO-d_6) δ 9.51 (s, 1H), 9.34 (s, 1H), 8.56 (d, *J* = 8.5 Hz, 2H), 8.23 (d, *J* = 7.9 Hz, 1H), 7.96 (s, 3H), 7.33 – 7.25 (m, 5H), 7.19 (t, *J* = 7.2 Hz, 1H), 7.04 (d, *J* = 8.1 Hz, 3H), 6.70 – 6.65 (m, 3H), 6.55 (s, 2H), 5.02 (s, 1H), 4.70 (td, *J* = 8.7, 5.0 Hz, 1H), 4.34 (dd, *J* = 8.5, 4.9 Hz, 1H), 4.06 (s, 1H), 3.97 (d, *J* = 6.5 Hz, 1H), 3.19 (dd, *J* = 14.1, 4.9 Hz, 1H), 2.96 (dd, *J* = 14.4, 4.6 Hz, 1H), 2.92 – 2.84 (m, 1H), 2.72 (dd, *J* = 14.4, 8.6 Hz, 1H), 2.30 (d, *J* = 13.0 Hz, 1H), 1.98 (s, 3H), 1.61 (s, 3H), 1.06 (d, *J* = 6.3 Hz, 4H).

^{13}C NMR (126 MHz, DMSO) δ 169.85, 168.77, 165.09, 162.38, 157.01, 155.28, 137.17, 130.97, 130.02, 129.65, 128.77, 127.10, 125.26, 115.80, 67.25, 58.93, 58.37, 54.00, 49.41, 37.12, 36.78, 22.84, 20.06, 19.59.

ESI-LRMS: calcd. for $C_{27}H_{35}N_4O_6S$ Exact Mass: $[M+H]^+$: 543.2277 (*m/z*); found $[M+H]^+$: 543.2367.

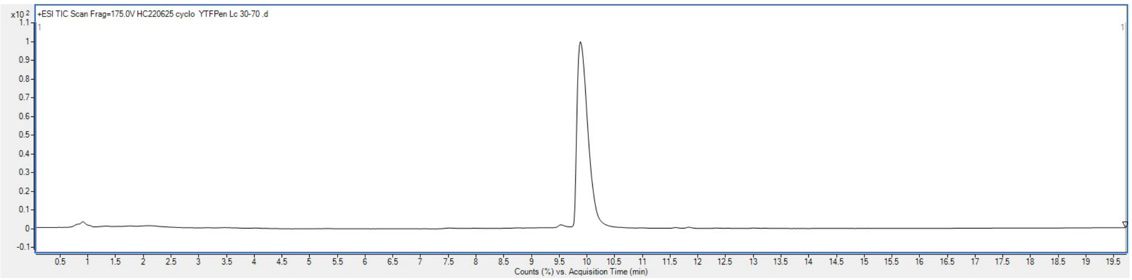

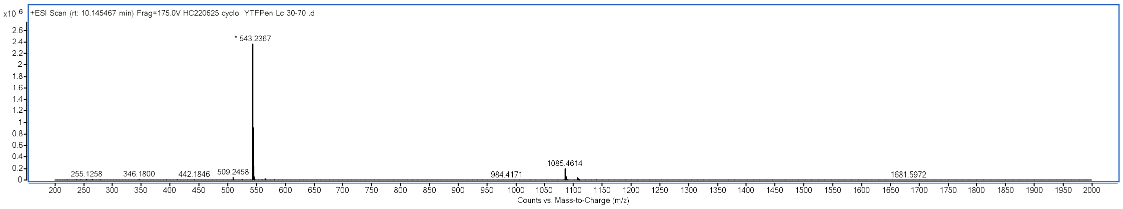

cyclo-tetrapeptides 14a

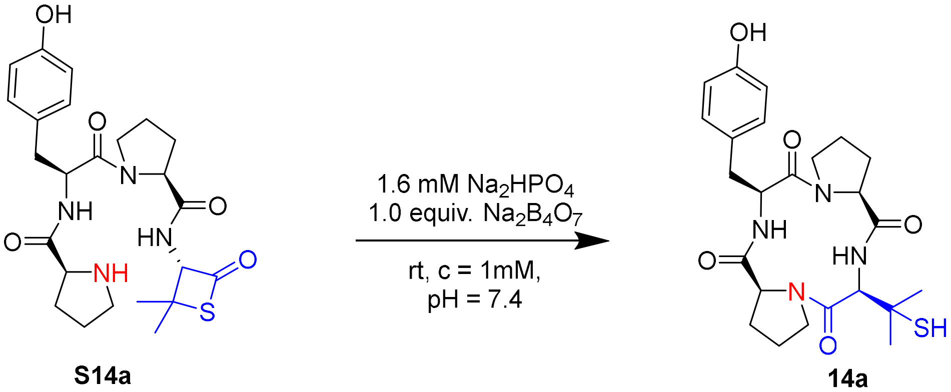

According to the general procedure D, **S14a** (5.0 mg, 0.010 mmol) was dissolved in 10.2 mL cyclization buffer with $Na_2B_4O_7$ (pH = 7.4). After 4h at room temperature, the cyclization reaction was complete (checked by LC-MS). The purification of the crude peptide using preparative HPLC (20 to 60% solvent B over 20 min, Higgins Analytical Proto 200 5 µm 250 × 10 nm C18 column) afforded peptide **14a** as a white solid after lyophilization (3.4 mg, 68%).

Analytical HPLC: t_R = 15.36 min (20 to 60% solvent B over 20 min, Higgins Analytical Proto 200 5 µm 150 × 2.0 nm C18 column).
^{1}H NMR (500 MHz, DMSO-d_6) δ 9.08 (s, 1H), 8.09 (t, J = 10.3 Hz, 2H), 6.96 (d, J = 8.2 Hz, 2H), 6.59 (d, J = 8.3 Hz, 2H), 4.86 (d, J = 5.1 Hz, 1H), 4.60 (d, J = 9.5 Hz, 1H), 4.32 – 4.22 (m, 1H), 4.10 (d, J = 8.0 Hz, 1H), 3.55 (dd, J = 11.6, 7.3 Hz, 2H), 3.44 (td, J = 11.3, 7.8 Hz, 2H), 3.24 (dd, J = 11.7, 8.7 Hz, 1H), 3.13 (t, J = 10.5 Hz, 1H), 3.04 (d, J = 3.4 Hz, 1H), 3.01 (d, J = 5.1 Hz, 1H), 2.04 – 1.99 (m, 2H), 1.94 – 1.85 (m, 2H), 1.86 – 1.78 (m, 1H), 1.66 (dt, J = 12.2, 6.5 Hz, 2H), 1.63 – 1.54 (m, 1H), 1.37 (s, 3H), 1.25 (s, 4H).
^{13}C NMR (126 MHz, DMSO-d_6) δ 171.86, 171.77, 170.25, 168.91, 155.93, 130.85, 128.50, 115.01, 61.52, 61.48, 57.98, 51.85, 47.19, 46.76, 46.72, 37.16, 32.08, 31.41, 30.59, 29.50, 22.17, 20.99.
ESI-LRMS: calcd. for $C_{24}H_{33}N_4O_5S$ Exact Mass: $[M+H]^+$: 489.2166 (m/z); found $[M+H]^+$: 489.2147.

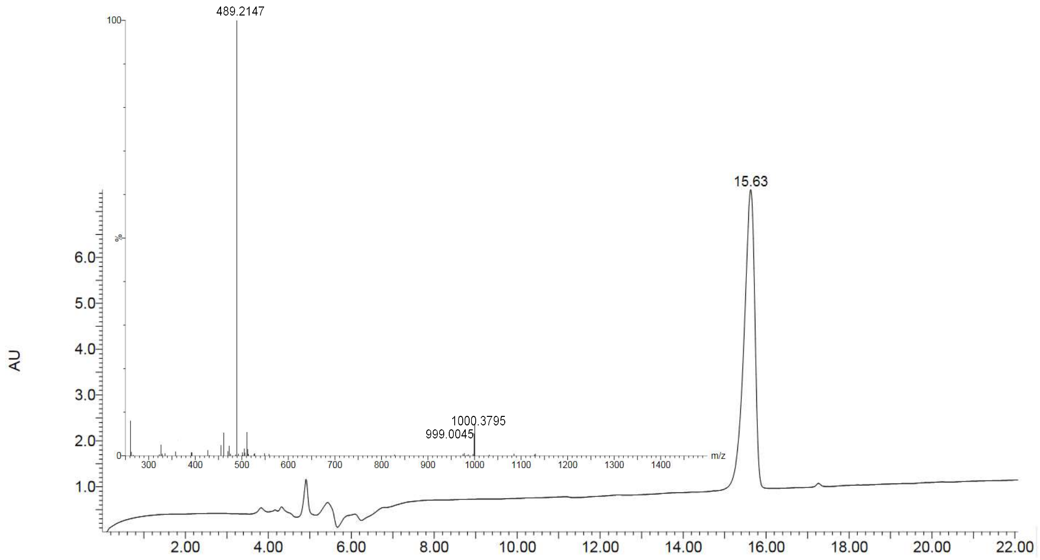

cyclo-tetrapeptides 1

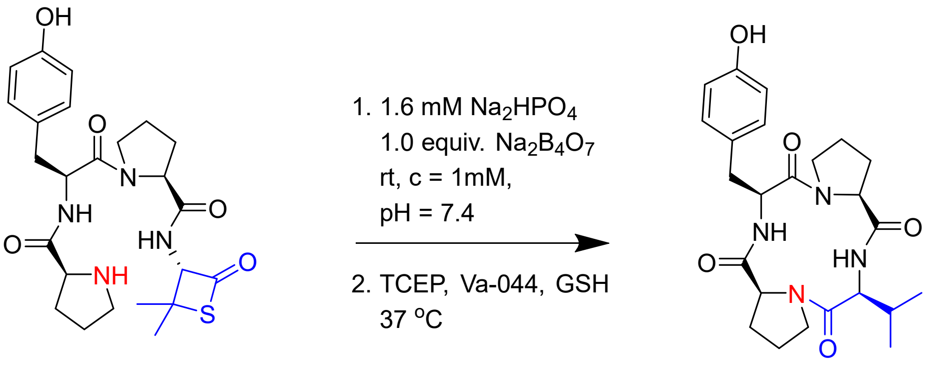

S14a **1**

According to the general procedure D and E, **S14a** (5.00 mg, 0.010 mmol) was dissolved in 10.2 mL cyclization buffer with $Na_2B_4O_7$ (pH = 7.4). After the cyclization (4h) and desulfurization (1h) one pot reaction was done (checked by Lc-MS for each step). Purification of the crude cyclo-tetrapeptide using preparative HPLC (20 to 60% solvent B over 20 min, Higgins Analytical Proto 200 5 µm 250 × 10 nm C18 column) afforded peptide **1** as a white solid after lyophilization (2.65 mg, 57% for two steps).

Analytical HPLC: t_R = 10.85 min (20 to 60% solvent B over 20 min, Higgins Analytical Proto 200 5 µm 150 × 2.0 nm C18 column).

^{1}H NMR (600 MHz, DMSO-*d6*) δ 9.08 (s, 1H), 7.98 – 7.97 (d, *J* = 9.8 Hz, 1H), 7.84 – 7.82 (d, *J* = 9.7 Hz, 1H), 6.93 – 6.92 (d, *J* = 8.4 Hz, 2H), 6.59 – 6.54 (m, 2H), 4.86 – 4.82 (td, *J* = 9.9, 5.0 Hz, 1H), 4.40 – 4.38 (dd, *J* = 9.6, 4.8 Hz, 1H), 4.22 – 4.21 (d, *J* = 7.7 Hz, 1H), 4.03 – 4.01 (d, *J* = 8.2 Hz, 1H), 3.51 – 3.46 (m, 1H), 3.42 – 3.39 (m, 1H), 3.20 – 3.16 (m, 1H), 3.10 – 3.06 (m, 1H), 3.00 – 2.97 (dd, *J* = 14.3, 4.9 Hz, 1H), 2.60 – 2.59 (m, 1H), 2.37 – 2.36 (m, 1H), 2.10 – 2.06 (m, 1H), 2.04 – 2.01 (m, 1H), 1.94 – 1.92 (m, 1H), 1.89 – 1.86 (m, 1H), 1.79 – 1.75 (m, 1H), 1.62 – 1.59 (m, 2H), 1.49 (m, 1H), 0.80 – 0.79 (d, *J* = 6.7 Hz, 2H), 0.77 – 0.76 (d, *J* = 6.7 Hz, 2H).

^{13}C NMR (126 MHz, DMSO-*d6*) δ 171.66, 171.52, 170.78, 170.63, 155.91, 130.85 (2C), 128.50, 115.00

(2C), 61.65, 61.45, 55.16, 51.75, 47.09, 46.76, 37.12, 31.85, 31.34, 29.15, 22.06, 21.26, 21.01, 17.97.
ESI-LRMS: calcd. for $C_{24}H_{33}N_4O_5$ Exact Mass: $[M+H]^+$: 457.2445 (*m/z*); found $[M+H]^+$: 457.2499.

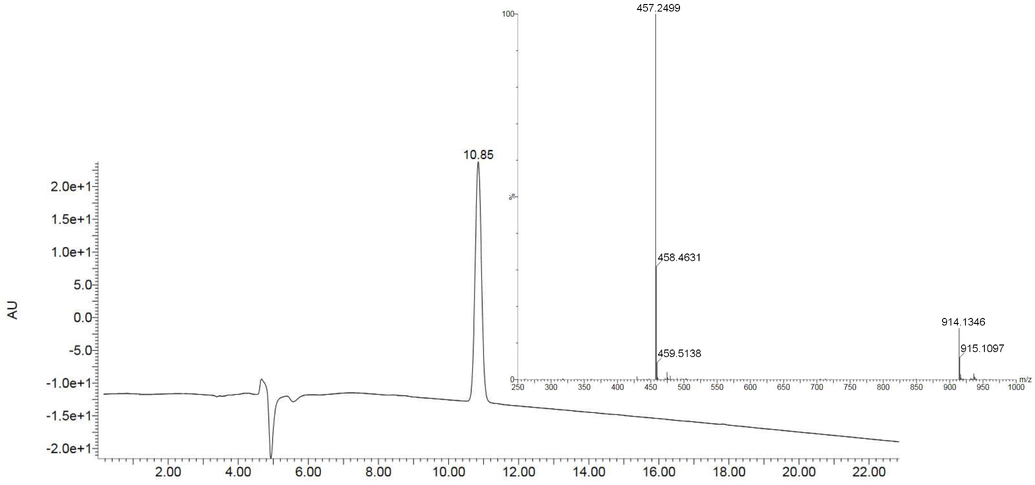

cyclo-tetrapeptides 2a

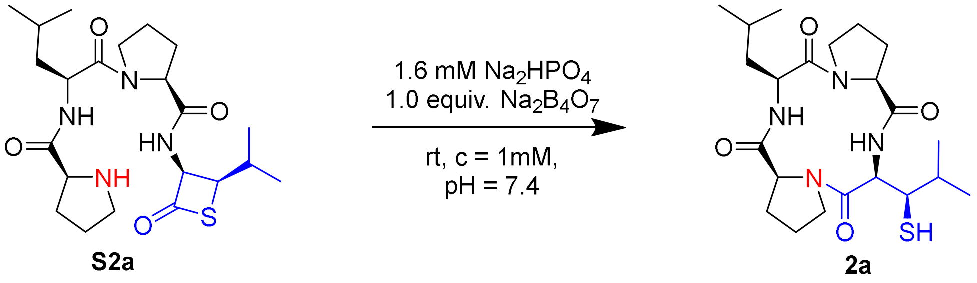

According to the general procedure D, **S2a** (5.00 mg, 0.011 mmol) was dissolved in 11.0 mL cyclization buffer with $Na_2B_4O_7$ (pH = 7.4). After 4h at room temperature, the cyclization reaction was complete (checked by LC-MS). Purification of the crude peptide using preparative HPLC (30 to 70% solvent B over 20 min, Higgins Analytical Proto 200 5 µm 250 × 10 nm C18 column) afforded peptide **2a** as a white solid after lyophilization (2.8 mg, 56%).

Analytical HPLC: t_R = 18.13 min (30 to 60% solvent B over 20 min, Higgins Analytical Proto 200 5 µm 150 × 2.0 nm C18 column).
ESI-LRMS: calcd. For $C_{22}H_{37}N_4O_4S$ Exact Mass: $[M+H]^+$: 453.2530 (*m/z*); found $[M+H]^+$: 453.2584.

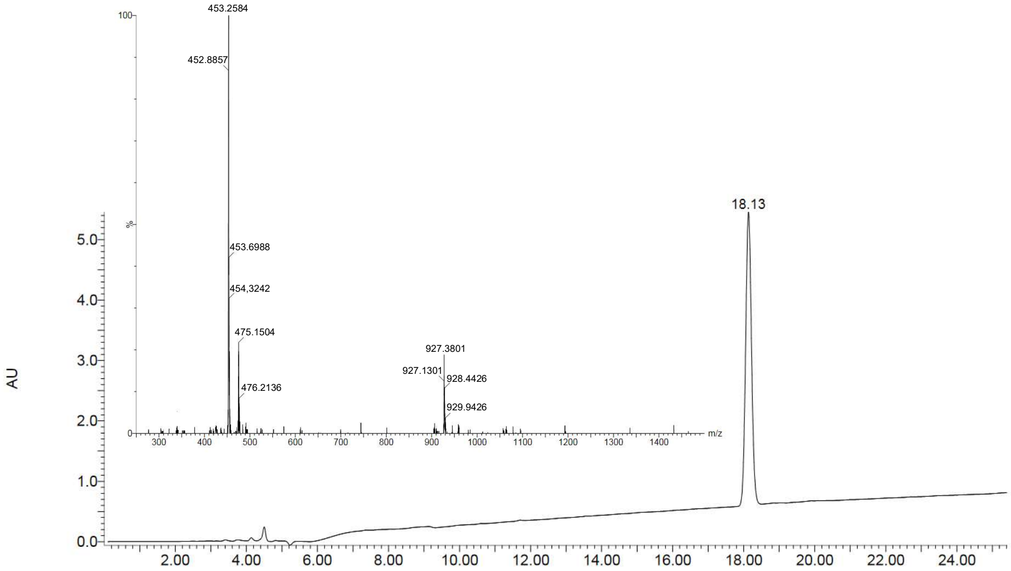

cyclo-tetrapeptides 2

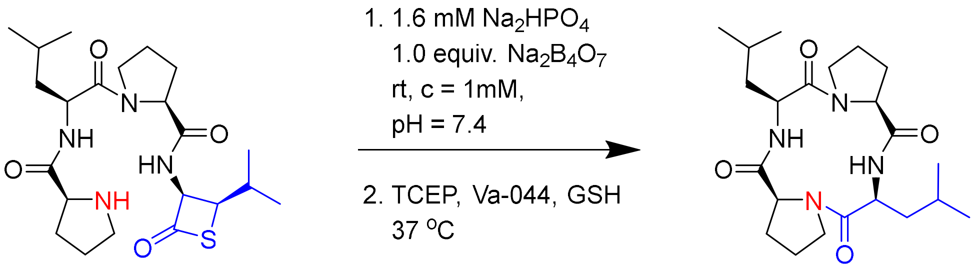

S2a **2**

According to the general procedure D and E, **S2a** (5.00 mg, 0.011 mmol) was dissolved in 11.0 mL cyclization buffer with $Na_2B_4O_7$ (pH = 7.4). After the cyclization (4h) and desulfurization (1h) one pot reaction was complete (checked by LC-MS for each step), purification of the crude cyclo-tetrapeptide using preparative HPLC (30 to 70% solvent B over 20 min, Higgins Analytical Proto 200 5 µm 250 × 10 nm C18 column) afforded peptide **2** as a white solid after lyophilization (2.0 mg, 43% for two steps). Analytical HPLC: t_R = 12.91 min (20 to 60% solvent B over 20 min, Higgins Analytical Proto 200 5 µm 150 × 2.0 nm C18 column).

Cyclopeptide **2** exists as two conformers in DMSO-*d6* (ratio = 5: 3), while only one conformer shows in $CDCl_3$.^{1}H NMR spectrum in $CDCl_3$ clearly demonstrates its symmetric structure where peaks for two Pros and Leus completely overlapped. ^{1}H NMR spectrum is also identical to the literature reported data[4]. (see ^{1}H spectra in section VII below in DMSO-*d6* and $CDCl_3$ for details)

^{1}H NMR (600 MHz, DMSO-*d6*) δ 7.87 (d, *J* = 9.7 Hz, 2H), 4.67 – 4.61 (m, 2H), 4.23 (d, *J* = 7.7 Hz, 2H), 3.21 – 3.13 (m, 2H), 2.02 – 1.99 (dd, *J* = 11.8, 6.5 Hz, 2H), 1.98 – 1.92 (m, 2H), 1.84 – 1.81 (m, 2H), 1.73 – 1.71 (m, 2H), 1.57 – 1.52 (ddd, *J* = 13.6, 9.6, 4.0 Hz, 2H), 1.47 – 1.45 (m, 2H), 1.37 – 1.36 (m, 2H), 1.34 – 1.29 (m, 2H), 0.86 – 0.84 (m, 12H).

^{1}H NMR (500 MHz, $CDCl_3$) δ 7.62 (s, 1H), 4.88 (s, 1H), 4.53 – 4.52 (d, *J* = 7.2 Hz, 1H), 3.61 – 3.55 (td, *J* =

11.5, 7.4 Hz, 1H), 3.38 (s, 1H), 2.40 (s, 1H), 2.15 – 2.10 (ddd, *J* = 20.8, 13.1, 7.8 Hz, 1H), 2.02 – 1.97 (dd, *J* = 12.5, 6.5 Hz, 1H), 1.70 – 1.69 (d, *J* = 9.0 Hz, 2H), 1.52 – 1.50 (m, 2H), 0.98 – 0.97 (d, *J* = 6.4 Hz, 3H), 0.94 – 0.93 (d, *J* = 6.5 Hz, 3H).

^{13}C NMR (126 MHz, DMSO-*d*6) δ 171.65, 171.23, 61.55, 48.97, 47.13, 41.42, 31.69, 24.33, 24.19, 22.30, 21.99.

ESI-LRMS: calcd. for $C_{22}H_{37}N_4O_4$ Exact Mass: $[M+H]^+$: 421.2809 (*m/z*); found $[M+H]^+$: 421.2748.

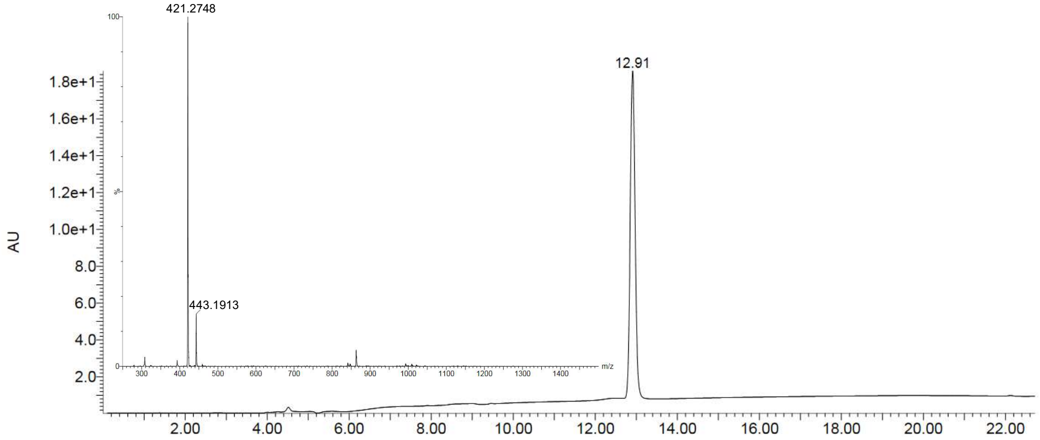

cyclo-tetrapeptides 4a

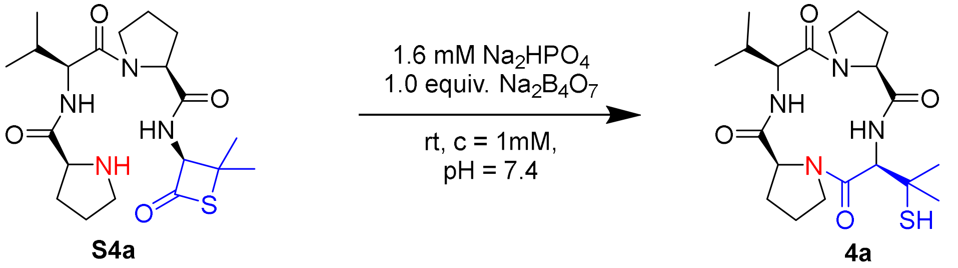

According to the general procedure D, **S4a** (5.00 mg, 0.012 mmol) was dissolved in 11.8 mL cyclization buffer with $Na_2B_4O_7$ (pH = 7.4). After 4h at room temperature, the cyclization reaction was complete (checked by LC-MS). Purification of the crude peptide using preparative HPLC (20 to 60% solvent B over 20 min, Higgins Analytical Proto 200 5 µm 250 × 10 nm C18 column) afforded peptide **4a** as a white solid after lyophilization (2.2 mg, 44%).

Analytical HPLC: t_R = 14.84 min (20 to 80% solvent B over 20 min, Higgins Analytical Proto 200 5 µm 150 × 2.0 nm C18 column).

ESI-LRMS: calcd. For Exact Mass: $C_{20}H_{33}N_4O_4S$ $[M+H]^+$: 425.2217 (*m/z*); found $[M+H]^+$: 425.2153.

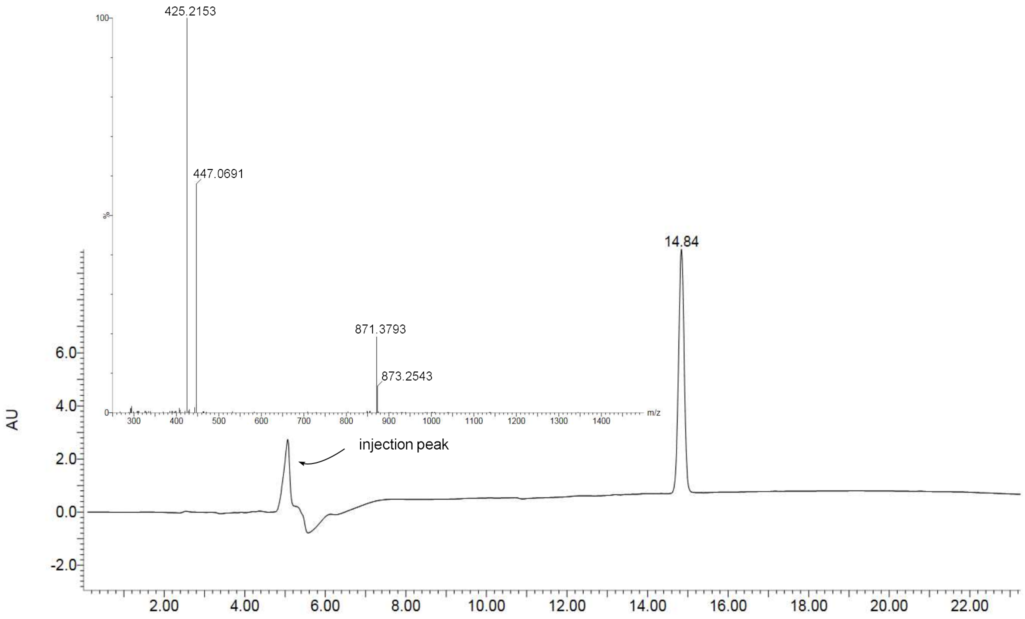

cyclo-tetrapeptides 4

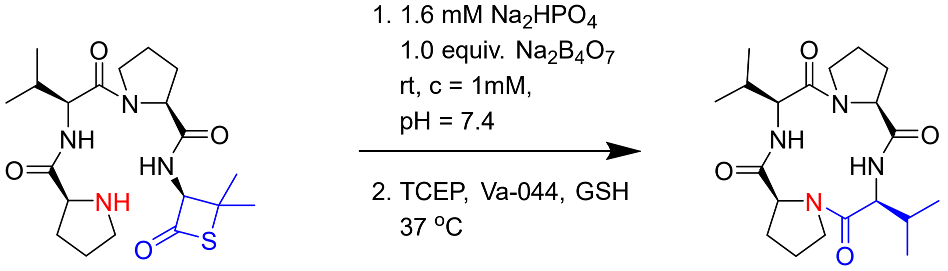

S4a **4**

According to the general procedure D and E, **S4a** (5.00 mg, 0.012 mmol) was dissolved in 11.8 mL cyclization buffer with $Na_2B_4O_7$ (pH = 7.4). After the cyclization (4h) and desulfurization (1.5h) one pot reaction was complete (checked by LC-MS for each step), purification of the crude cyclo-tetrapeptide using preparative HPLC (20 to 60% solvent B over 20 min, Higgins Analytical Proto 200 5 µm 250 × 10 nm C18 column) afforded peptide **4** as a white solid after lyophilization (1.6 mg, 35% for two steps). Analytical HPLC: t_R = 9.96 min (30 to 60% solvent B over 20 min, Higgins Analytical Proto 200 5 µm 150 × 2.0 nm C18 column).

Cyclopeptide **4** exists as one conformer in DMSO-*d6* (ratio = 3: 5), the minor conformer was symmetric, and the major conformer was asymmetric, while in MeOH there was only one conformer which shows asymmetric conformation (probably due to the rotation of phenyl rings). (see ^{1}H spectra in section VII below in DMSO-*d6* and MeOH-*d4* for details)

^{1}H NMR (600 MHz, DMSO-*d6*) δ 7.92 – 9.91 (d, *J*=9.5 Hz, 2H), 4.45 – 4.42 (dd, *J* = 9.5, 5.3 Hz, 2H), 4.20–4.19 (d, *J* = 7.6, 2H), 3.50 – 3.45 (m, 2H), 3.18–3.15 (dd, *J* = 11.6, 8.9 Hz, 2H), 2.60 – 2.59 (m, 1H), 2.37 – 2.36 (m, 1H), 2.11 – 2.06 (m, 2H), 2.02 – 1.92 (m, 4H), 1.89 – 1.84 (m, 2H), 0.82 – 0.80 (m, 12H).

^{13}C NMR (151 MHz, DMSO-*d6*) δ 171.63 (2C), 170.96 (2C), 61.70 (2C), 55.14 (2C), 46.85 (2C), 31.96 (2C), 29.25 (2C), 22.03 (2C), 21.06 (2C), 18.98 (2C).

ESI-LRMS: calcd. for Exact Mass: $C_{20}H_{33}N_4O_4$ $[M+H]^+$: 393.2496 (*m/z*); found $[M+H]^+$: 393.2690.

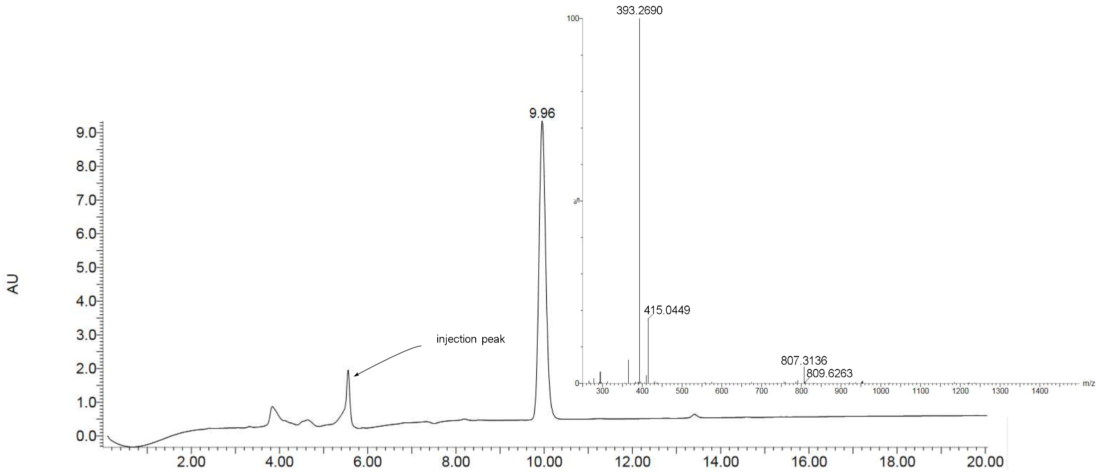

cyclo-tetrapeptides 14c

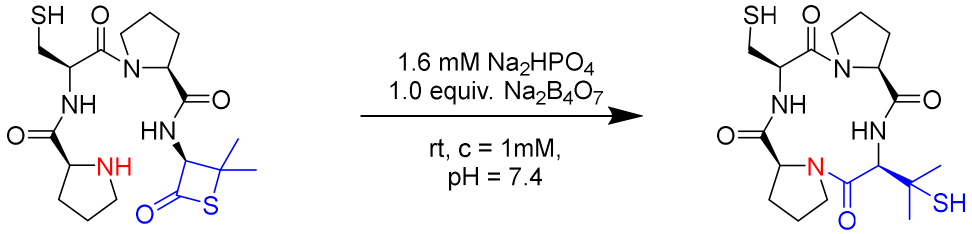

S14c **14c**

According to the general procedure D, **S14c** (5.00 mg, 0.012 mmol) was dissolved in 11.6 mL cyclization buffer with $Na_2B_4O_7$ (pH = 7.4). After 6h at room temperature, the cyclization reaction was complete (checked by LC-MS). Purification of the crude peptide using preparative HPLC (30 to 50% solvent B over 20 min, Higgins Analytical Proto 200 5 µm 250 × 10 nm C18 column) afforded peptide **14c** as a white solid after lyophilization (3.35 mg, 67%).

Analytical HPLC: t_R = 11.89 min (30 to 50% solvent B over 20 min, Higgins Analytical Proto 200 5 µm 150 × 2.0 nm C18 column).

^{1}H NMR (600 MHz, DMSO-*d6*) δ 8.24 – 8.22 (d, *J* = 9.6 Hz, 1H), 8.08 – 8.06 (d, *J* = 9.5 Hz, 1H), 6.51 (s, 1H), 4.75 – 4.73 (dd, *J* = 15.8, 6.6 Hz, 1H), 4.65 – 4.64 (d, *J* = 9.5 Hz, 1H), 4.26 – 4.22 (dd, *J* = 19.6, 7.7 Hz, 2H), 3.50 – 3.42 (dtd, *J* = 18.7, 11.4, 7.4 Hz, 2H), 3.24 – 3.18 (m, 2H), 3.01 (s, 1H), 2.83 – 2.78 (ddd, *J* = 13.5, 7.6, 6.0 Hz, 1H), 2.54 – 2.49 (m, 1H), 2.06 – 2.03 (m, 1H), 2.02 – 1.99 (m, 1H), 1.98 – 1.93 (m, 2H), 1.91 – 1.87 (dt, *J* = 10.7, 6.7 Hz, 1H), 1.84 – 1.79 (m, 1H), 1.62 – 1.56 (dd, *J* = 20.3, 14.4 Hz, 2H), 1.36 (s, 3H), 1.24 (s, 3H).

^{13}C NMR (151 MHz, DMSO-*d6*) δ 171.75, 170.94, 170.58, 168.96, 61.64, 61.58, 57.81,52.49, 47.13, 46.88, 40.50, 32.17, 31.84, 30.58, 29.54, 26.38, 22.17, 21.61.

ESI-LRMS: calcd. for Exact Mass: $C_{18}H_{29}N_4O_4S_2$ $[M+H]^+$: 429.1625 (*m/z*); found $[M+H]^+$: 429.1184.

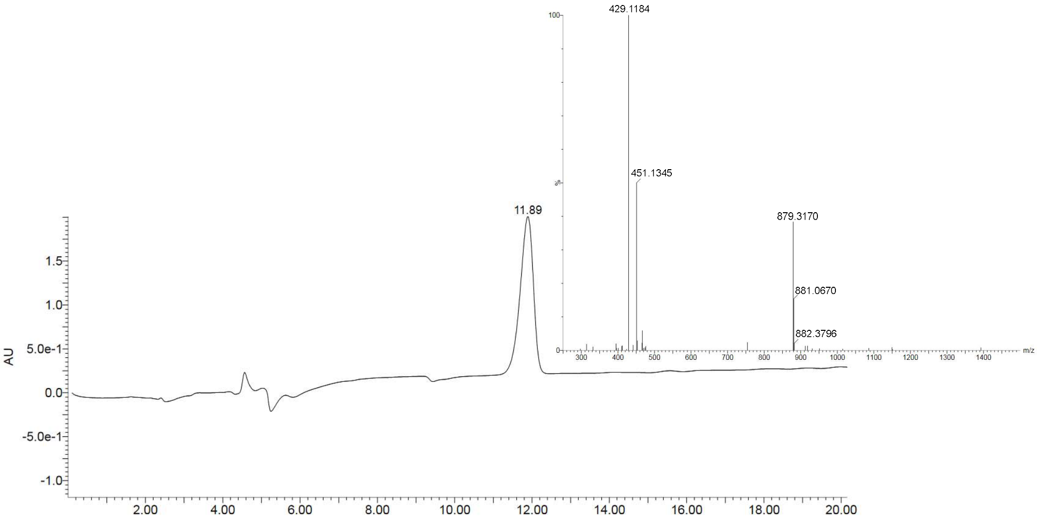

cyclo-tetrapeptides 15c

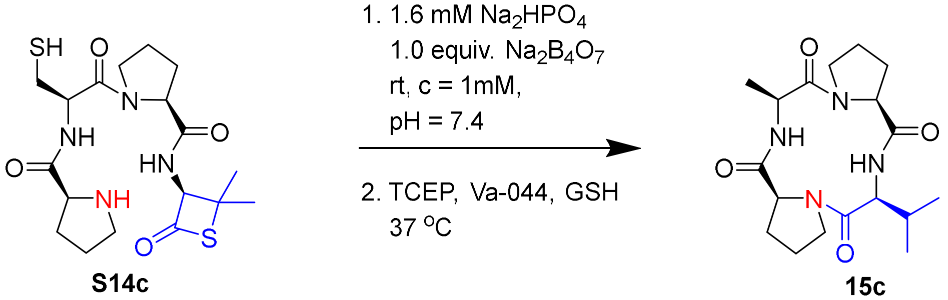

According to the general procedure D and E, **S14c** (5.00 mg, 0.012 mmol) was dissolved in 11.6 mL cyclization buffer with $Na_2B_4O_7$ (pH = 7.4). After the cyclization (4h) and desulfurization (2h) one pot reaction was complete (checked by LC-MS for each step), purification of the crude cyclo-tetrapeptide using preparative HPLC (18 to 40% solvent B over 20 min, Higgins Analytical Proto 200 5 µm 250 × 10 nm C18 column) afforded peptide **15c** as a white solid after lyophilization (0.51 mg, 55% for two steps).

Analytical HPLC: t_R = 6.63 min (20 to 50% solvent B over 20 min, Higgins Analytical Proto 200 5 µm 150 × 2.0 nm C18 column).

^{1}H NMR (500 MHz, DMSO-*d6*) δ 8.16 (d, *J* = 9.2 Hz, 1H), 7.83 (d, *J* = 9.8 Hz, 1H), 4.79 – 4.68 (m, 1H), 4.47 (dd, *J* = 9.3, 4.6 Hz, 1H), 4.23 (d, *J* = 7.3 Hz, 1H), 4.19 (d, *J* = 7.8 Hz, 1H), 3.33 – 3.09 (m, 4H), 2.12 (d, *J* = 5.7 Hz, 1H), 2.02 (s, 2H), 1.99 – 1.86 (m, 2H), 1.80 – 1.79 (m, 2H), 1.60 – 1.42 (m, 2H), 1.09 (d, *J* = 6.6 Hz, 3H), 0.83 (d, *J* = 6.7 Hz, 3H), 0.81 (d, *J* = 6.7 Hz, 3H).

^{13}C NMR (126 MHz, DMSO-*d6*) δ 171.91, 171.70, 170.85, 170.51, 61.72, 61.59, 55.02, 47.08, 46.86, 46.32, 31.98, 31.55, 29.20, 22.16, 21.85, 20.97, 18.32, 17.95.

ESI-LRMS: calcd. for Exact Mass: $C_{18}H_{29}N_4O_4$ $[M+H]^+$: 365.2183 (*m/z*); found $[M+H]^+$: 365.2964.

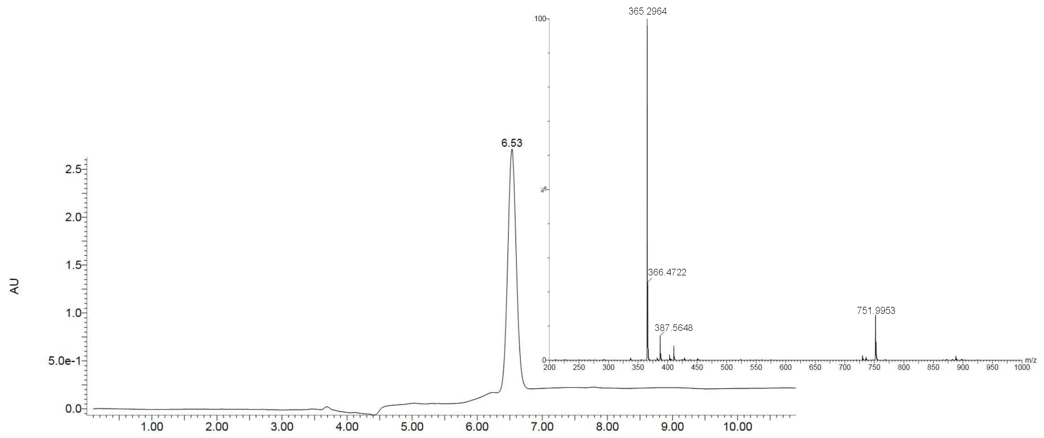

cyclo-tetrapeptides 14d

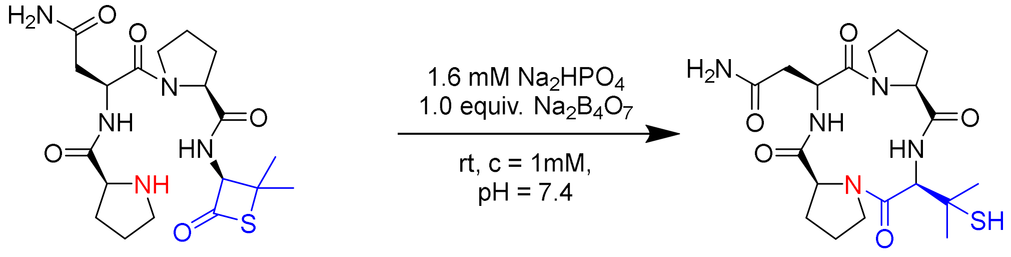

S14d **14d**

According to the general procedure D, **S14d** (5.00 mg, 0.011 mmol) was dissolved in 11.3 mL cyclization buffer with $Na_2B_4O_7$ (pH = 7.4). After 4h at room temperature, the cyclization reaction was complete (checked by LC-MS). Purification of the crude peptide using preparative HPLC (10 to 40% solvent B over 20 min, Higgins Analytical Proto 200 5 µm 250 × 10 nm C18 column) afforded peptide **14d** as a white solid after lyophilization (2.8 mg, 56%).

Analytical HPLC: t_R = 12.18 min (20 to 80% solvent B over 20 min, Higgins Analytical Proto 200 5 µm 150 × 2.0 nm C18 column).

^{1}H NMR (500 MHz, DMSO-d_6) δ 8.20 (d, J = 9.6 Hz, 1H), 8.10 (d, J = 9.4 Hz, 1H), 5.08 – 4.95 (m, 1H), 4.66 (d, J = 9.4 Hz, 1H), 4.24 (d, J = 7.7 Hz, 2H), 3.48 (dtd, J = 22.7, 11.4, 7.4 Hz, 3H), 3.23 (ddd, J = 17.6, 11.7, 8.7 Hz, 2H), 3.04 (s, 1H), 2.62 (dd, J = 14.7, 7.5 Hz, 1H), 2.09 – 2.02 (m, 3H), 2.02 – 1.97 (m, 1H), 1.98 – 1.89 (m, 2H), 1.86 – 1.73 (m, 2H), 1.60 (d, J = 10.8 Hz, 2H), 1.38 (s, 3H), 1.27 (s, 3H).

^{13}C NMR (126 MHz, DMSO-d_6) δ 172.03, 171.77, 170.89, 170.28, 168.95, 61.61, 61.58, 57.93, 48.34, 47.23, 46.88, 46.79, 38.59, 32.21, 31.71, 30.58, 22.18, 21.40.

ESI-LRMS: calcd. for Exact Mass: $C_{19}H_{30}N_5O_5S$ $[M+H]^+$: 440.1962 (*m/z*); found $[M+H]^+$: 440.1266.

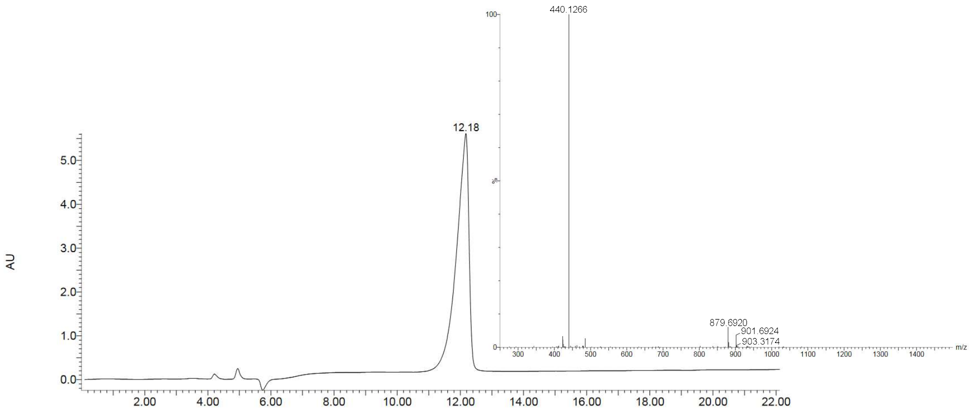

cyclo-tetrapeptides 14f

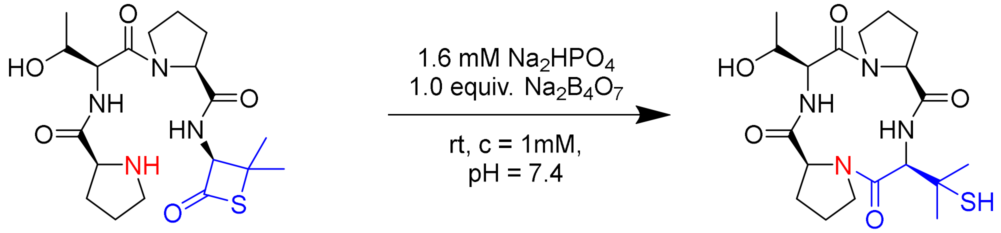

S14f **14f**

According to the general procedure D, **S14f** (5.00 mg, 0.012 mmol) was dissolved in 11.7 mL cyclization buffer with $Na_2B_4O_7$ (pH = 7.4). After 4h at room temperature, the cyclization reaction was complete (checked by LC-MS). Purification of the crude peptide using preparative HPLC (20 to 30% solvent B over 20 min, Higgins Analytical Proto 200 5 µm 250 × 10 nm C18 column) afforded peptide **14f** as a white solid after lyophilization (2.15 mg, 43%).

Analytical HPLC: t_R = 17.16 min (20 to 30% solvent B over 20 min, Higgins Analytical Proto 200 5 µm 150 × 2.0 nm C18 column).
ESI-LRMS: calcd. for Exact Mass: $C_{19}H_{31}N_4O_5S$ $[M+H]^+$: 427.2010 (*m/z*); found $[M+H]^+$: 427.1994.

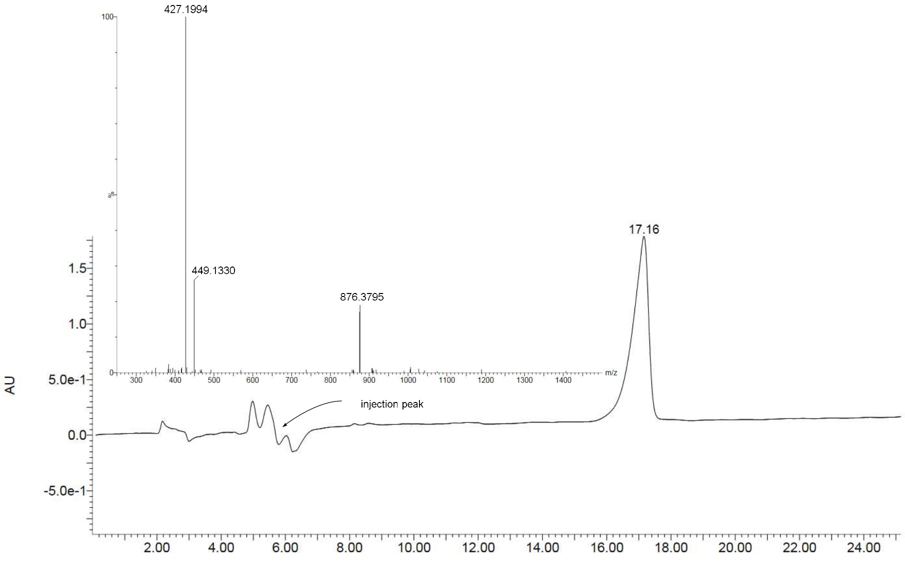

cyclo-tetrapeptides 15f

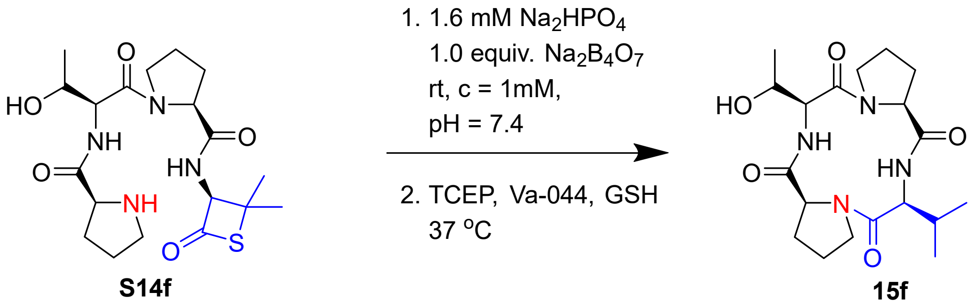

According to the general procedure D and E, **S14f** (5.00 mg, 0.012 mmol) was dissolved in 11.7mL cyclization buffer with $Na_2B_4O_7$ (pH = 7.4). After the cyclization (4h) and desulfurization (1h) one pot reaction was complete (checked by LC-MS for each step), purification of the crude cyclo-tetrapeptide using preparative HPLC (20 to 30% solvent B over 20 min, Higgins Analytical Proto 200 5 µm 250 × 10 nm C18 column) afforded peptide **15f** as a white solid after lyophilization (0.95 mg, 21% for two steps).

Analytical HPLC: t_R = 11.12 min (30 to 60% solvent B over 20 min, Higgins Analytical Proto 200 5 µm 150 × 2.0 nm C18 column).
^{1} H NMR (600 MHz, DMSO-*d*6) δ 8.03 – 8.02 (d, *J* = 9.5 Hz, 1H), 7.91 – 7.89 (d, *J* = 9.7 Hz, 1H), 4.53 – 4.51 (dd, *J* = 2.3, 9.7 Hz, 1H), 4.44 – 4.42 (dd, *J* = 5.2, 9.5 Hz, 1H), 4.20 – 4.19 (d, *J* = 6.8 Hz, 1H), 4.19 – 4.17 (m, 1H), 4.10 – 4.08 (m, 1H), 3.50 – 3.43 (m, 2H), 3.20 – 3.16 (m, 2H), 2.51 (s, 1H), 2.10 – 2.07 (m, 1H), 2.02 – 1.98 (m, 2H), 1.96 – 1.92 (m, 2H), 1.89 – 1.82 (m, 2H), 1.60 – 1.55 (m, 1H), 1.53 – 1.49 (m, 1H), 0.88 –0.87 (d, *J* = 6.3 Hz, 3H), 0.81 – 0.80 (d, *J* = 3.6, 3H), 0.79 – 0.79 (d, *J* = 3.6, 3H).
^{13} C NMR (151 MHz, DMSO-*d*6) δ 171.93, 171.64, 171.37, 171.00, 65.63, 61.85, 61.64, 55.20, 54.81, 46.99, 46.78, 31.92, 31.89, 29.22, 22.01, 21.95, 21.07, 19.67, 18.07.
ESI-LRMS: calcd. for Exact Mass: $C_{19}H_{31}N_4O_5$ $[M+H]^+$: 395.2289 (*m/z*); found $[M+H]^+$: 395.2307.

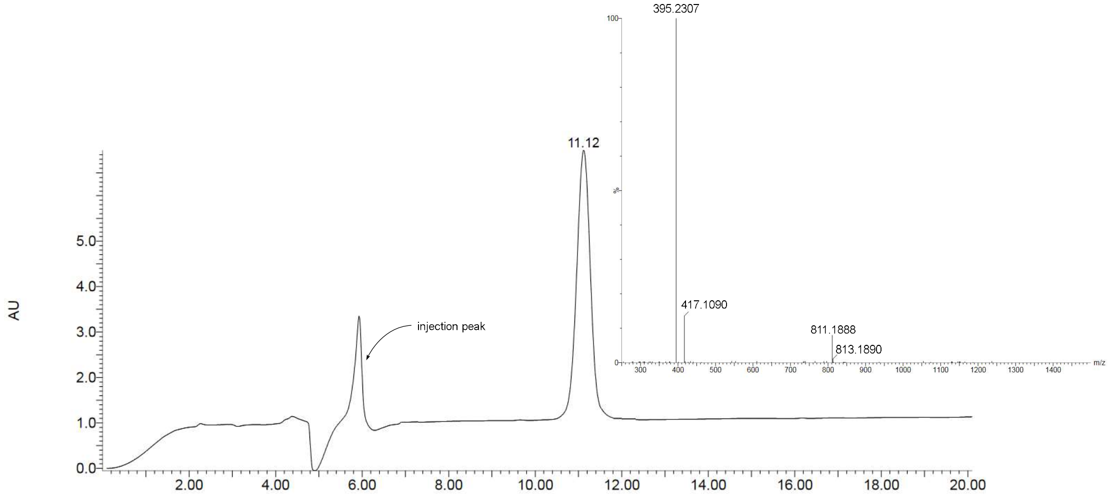

cyclo-tetrapeptides 14g

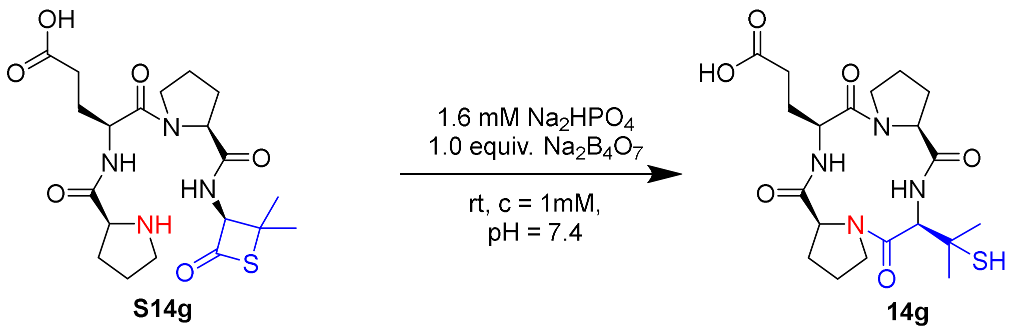

According to the general procedure D, **S14g** (5.00 mg, 0.011 mmol) was dissolved in 11.0 mL cyclization buffer with $Na_2B_4O_7$ (pH = 7.4). After 4h at room temperature, the cyclization reaction was complete (checked by LC-MS). Purification of the crude peptide using preparative HPLC (22% solvent B over 20 min, Higgins Analytical Proto 200 5 µm 250 × 10 nm C18 column) afforded peptide **14g** as a white solid after lyophilization (0.43 mg, 43%).

Analytical HPLC: t_R = 17.78 min (20 to 30% solvent B over 20 min, Higgins Analytical Proto 200 5 µm 150 × 2.0 nm C18 column).

^1H NMR (500 MHz, DMSO-d_6) δ 12.00 (s, 1H), 8.16 (d, *J* = 9.2 Hz, 1H), 8.07 (d, *J* = 9.5 Hz, 1H), 6.50 (s, 1H), 4.64 (d, *J* = 9.7 Hz, 2H), 4.21 (dd, *J* = 23.9, 7.5 Hz, 2H), 3.48 (t, *J* = 10.1 Hz, 5H), 3.21 (dd, *J* = 20.2, 10.1 Hz, 3H), 2.13 (t, *J* = 7.7 Hz, 2H), 2.02 (d, *J* = 6.8 Hz, 2H), 1.96 (dt, *J* = 20.1, 6.7 Hz, 4H), 1.86 (dq, *J* = 18.7, 6.2, 5.8 Hz, 3H), 1.66 – 1.57 (m, 2H), 1.50 (d, *J* = 9.5 Hz, 2H), 1.36 (s, 4H), 1.24 (s, 3H).

^{13}C NMR (126 MHz, DMSO-d_6) δ 174.66, 171.88, 171.39, 171.15, 169.03, 61.70, 61.56, 57.84, 50.17, 47.14, 46.81, 32.16, 31.81, 31.16, 30.57, 30.18, 29.53, 27.12, 22.20, 21.71.

ESI-LRMS: calcd. for Exact Mass: $C_{20}H_{31}N_4O_6S$ $[M+H]^+$: 455.1959 (*m/z*); found $[M+H]^+$: 455.2122.

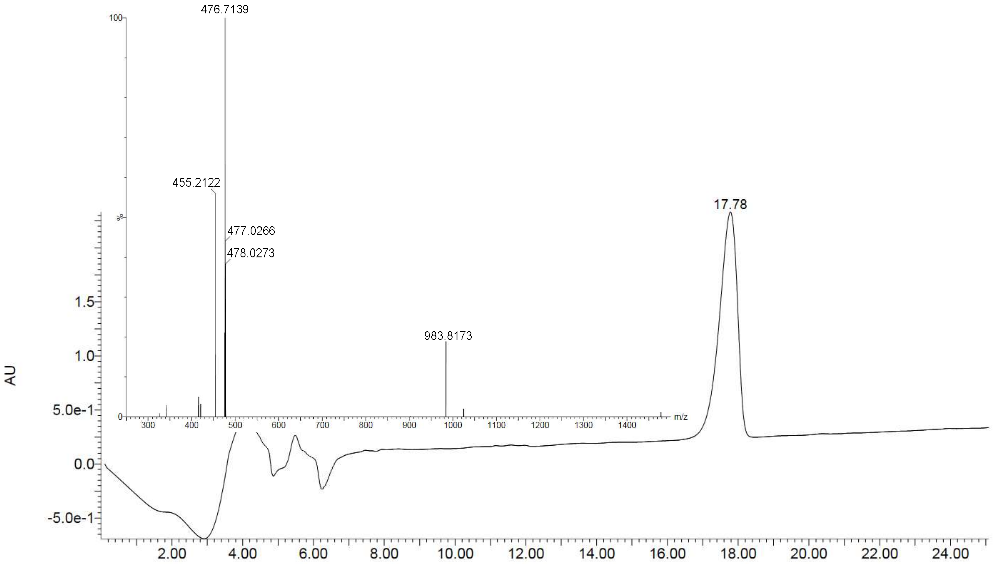

cyclo-tetrapeptides 14h

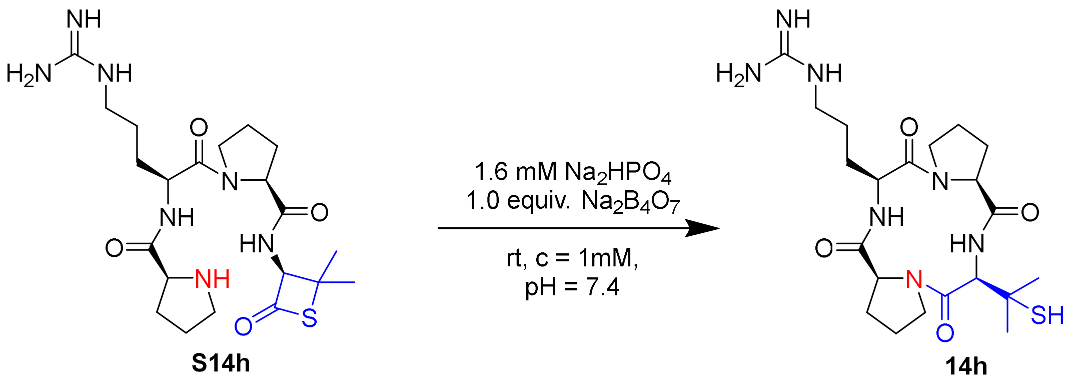

According to the general procedure D, **S14h** (5.00 mg, 0.010 mmol) was dissolved in 10.3 mL cyclization buffer with $Na_2B_4O_7$ (pH = 7.4). After 6h at room temperature, the cyclization reaction was complete (checked by LC-MS). Purification of the crude peptide using preparative HPLC (30% solvent B over 20 min, Higgins Analytical Proto 200 5 µm 250 × 10 nm C18 column) afforded peptide **14h** as a white solid after lyophilization (2.35 mg, 47%).

Analytical HPLC: t_R = 18.67 min (20% solvent B over 20 min, Higgins Analytical Proto 200 5 µm 150 × 2.0 nm C18 column).

^{1}H NMR (500 MHz, DMSO-d_6) δ 8.23 (d, J = 9.3 Hz, 1H), 8.07 (d, J = 9.5 Hz, 1H), 7.56 (t, J = 5.9 Hz, 1H), 7.26 (s, 1H), 6.84 (s, 1H), 6.55 (s, 1H), 4.67 (d, J = 9.5 Hz, 2H), 4.28 (t, J = 5.8 Hz, 2H), 3.50 (td, J = 7.4, 3.7 Hz, 2H), 3.28 – 3.19 (m, 2H), 3.13 (dq, J = 12.8, 6.6 Hz, 2H), 3.03 (s, 1H), 2.11 – 1.96 (m, 3H), 1.96 – 1.84 (m, 2H), 1.82 – 1.71 (m, 1H), 1.62 (q, J = 9.8 Hz, 1H), 1.55 – 1.45 (m, 1H), 1.39 (s, 3H), 1.35 – 1.30 (m, 1H), 1.26 (s, 2H).

^{13}C NMR (126 MHz, DMSO-d_6) δ 171.90, 171.51, 171.30, 169.03, 157.07, 61.72, 61.56, 57.82, 50.20, 47.16, 46.84, 32.19, 31.81, 30.58, 29.03, 25.15, 22.23, 21.76.

ESI-LRMS: calcd. for Exact Mass: $C_{21}H_{36}N_7O_4S$ $[M+H]^+$: 482.2544 (m/z); found $[M+H]^+$: 482.2675.

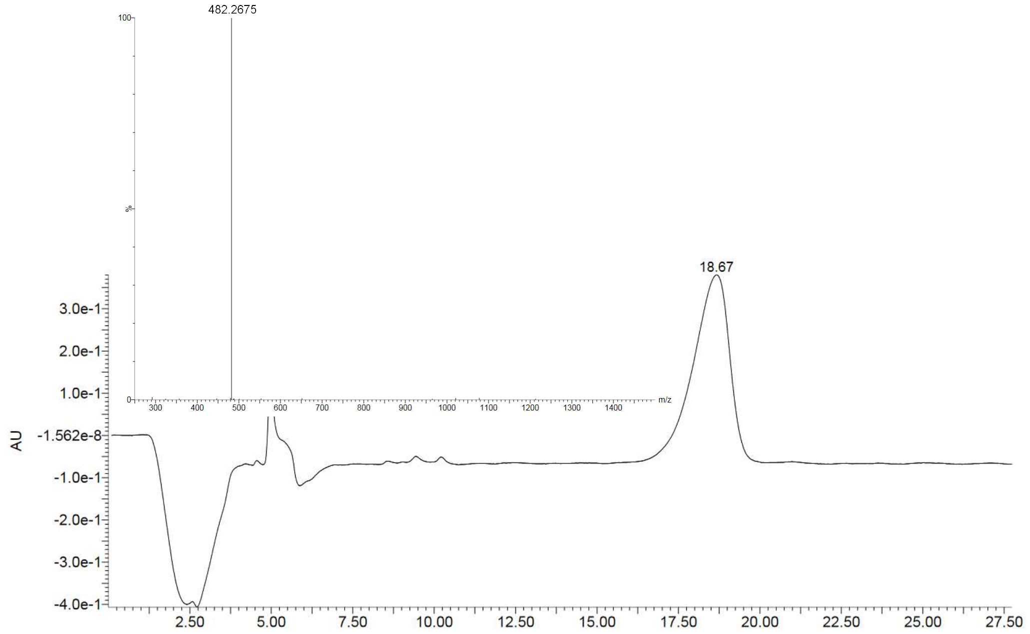

cyclo-tetrapeptides 14i

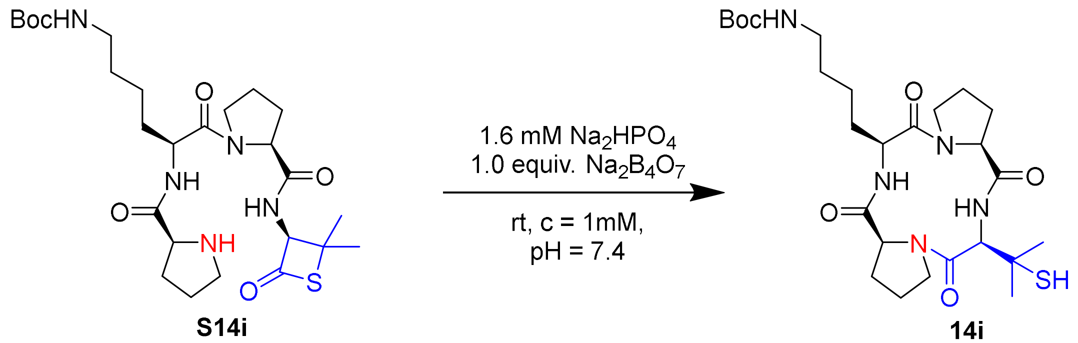

S14i **14i**

According to the general procedure D, **S14i** (5.00 mg, 0.0090 mmol) was dissolved in 9.0 mL cyclization buffer with $Na_2B_4O_7$ (pH = 7.4). After 6h at room temperature, the cyclization reaction was complete (checked by LC-MS). Purification of the crude peptide using preparative HPLC (20 to 60% solvent B over 20 min, Higgins Analytical Proto 200 5 µm 250 × 10 nm C18 column) afforded peptide **14i** as a white solid after lyophilization (3.15 mg, 63%).

Analytical HPLC: t_R = 14.85 min (30 to 80% solvent B over 20 min, Higgins Analytical Proto 200 5 µm 150 × 2.0 nm C18 column).
ESI-LRMS: calcd. for Exact Mass: $C_{26}H_{44}N_5O_6S$ $[M+H]^+$: 554.3007 (*m/z*); found $[M+H]^+$: 554.2903.

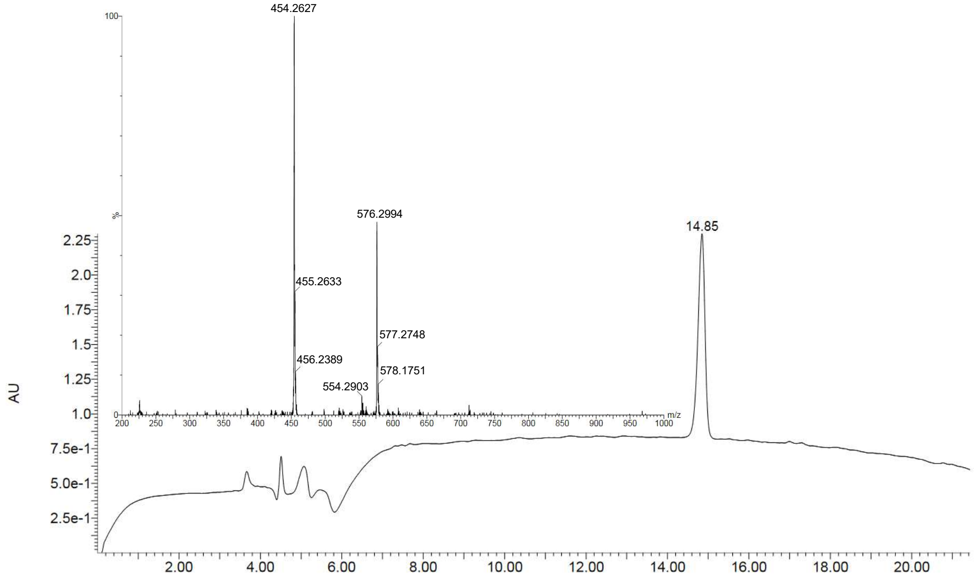

cyclo-tetrapeptides 15i

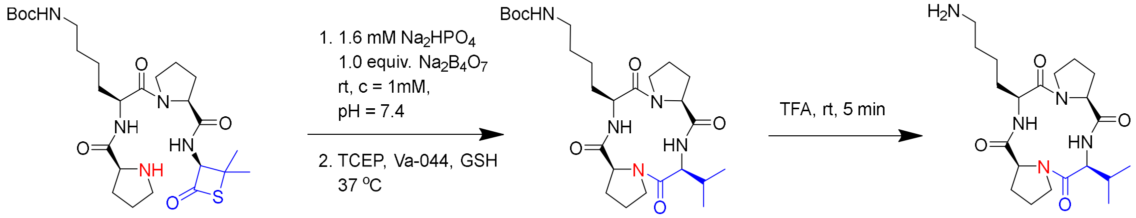

S14i **S12** **14i**

According to the general procedure D and E, **S14i** (5.00 mg, 0.0090 mmol) was dissolved in 9.0 mL cyclization buffer with $Na_2B_4O_7$ (pH = 7.4). After the cyclization (6h) and desulfurization (1h) one pot reaction was complete (checked by LC-MS for each step), purification of the crude cyclo-tetrapeptide using preparative HPLC (30 to 80% solvent B over 20 min, Higgins Analytical Proto 200 5 µm 250 × 10 nm C18 column) afforded peptide **S12** as a white solid after lyophilization.

Analytical HPLC: t_R = 12.45 min (30 to 80% solvent B over 20 min, Higgins Analytical Proto 200 5 µm 150 × 2.0 nm C18 column).
ESI-LRMS: calcd. for Exact Mass: $C_{26}H_{44}N_5O_6$ $[M+H]^+$: 522.3286 (*m/z*); found $[M+H]^+$: 522.3510.

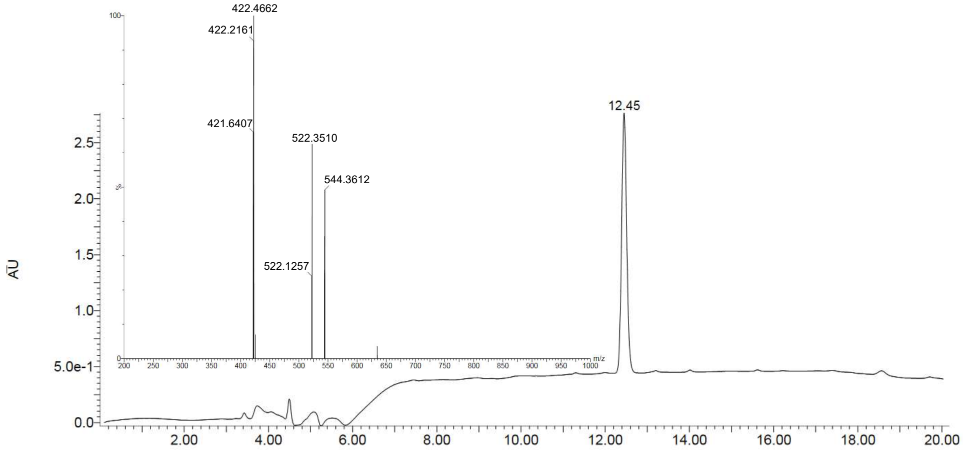

Cyclopeptide **S31** was treated with TFA for 5 min, and the TFA was blown away under argon afforded the desired product **19i** as white solid after lyophilization without further purification (0.9 mg, 24% over three steps).

Analytical HPLC: t_R = 16.63 min (10 to 30% solvent B over 20 min, Higgins Analytical Proto 200 5 µm 150 × 2.0 nm C18 column).
^{1}H NMR (500 MHz, DMSO-*d6*) δ 8.09 (d, *J* = 9.4 Hz, 1H), 7.84 (d, *J* = 9.6 Hz, 1H), 7.67 (s, 2H), 4.62 (d, *J* = 4.8 Hz, 1H), 4.47 (dd, *J* = 9.6, 4.9 Hz, 1H), 4.27 – 4.18 (m, 2H), 3.56 – 3.43 (m, 2H), 3.26 – 3.14 (m, 2H), 2.77 – 2.74 (m, 2H), 2.30 (dd, *J* = 16.3, 8.8 Hz, 1H), 2.13 (dd, *J* = 11.8, 6.7 Hz, 1H), 2.08 – 2.02 (m, 1H), 1.97 – 1.95 (m, 1H), 1.93 – 1.90 (m, 1H), 1.86 – 1.83 (m, 1H), 1.77 – 1.76 (m, 2H), 1.63 – 1.59 (m, 1H), 1.57 – 1.52 (m, 2H), 1.38 – 1.34 (m, 2H), 1.29 – 1.18 (m, 2H), 0.84 (d, *J* = 6.7 Hz, 3H), 0.82 (d, *J* = 6.8 Hz, 3H).
^{13}C NMR (126 MHz, DMSO-*d6*) δ 174.04, 171.55, 171.46, 170.89, 61.68, 55.09, 50.17, 47.03, 46.92, 39.24, 31.95, 31.76, 31.47, 29.19, 27.25, 26.52, 22.41, 22.09, 22.02, 20.99, 17.96.
ESI-LRMS: calcd. for Exact Mass: $C_{21}H_{36}N_5O_4$ $[M+H]^+$: 422.2762 (*m/z*); found $[M+H]^+$: 422.2639.

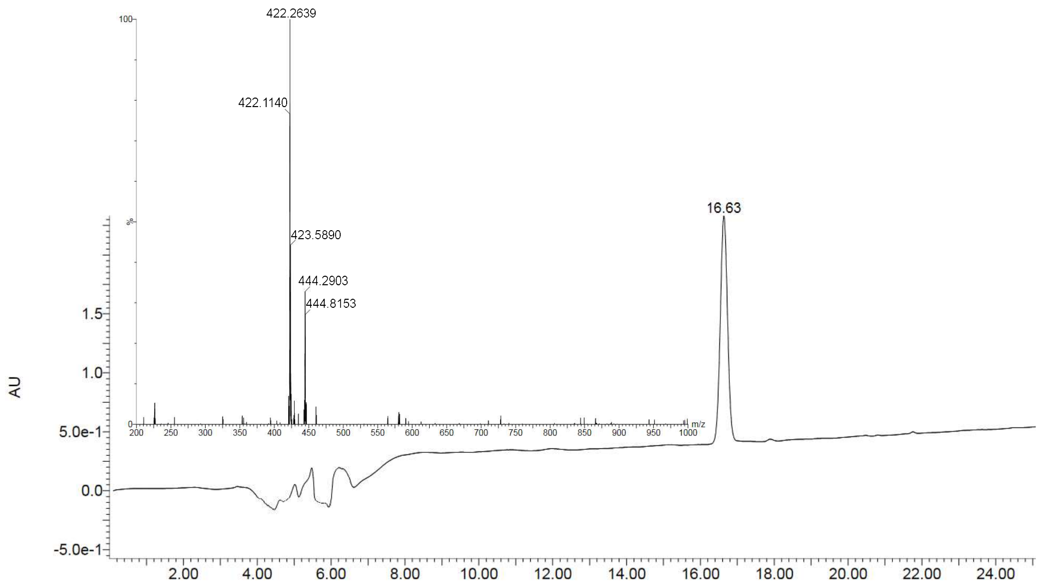

cyclo-tetrapeptides 14j

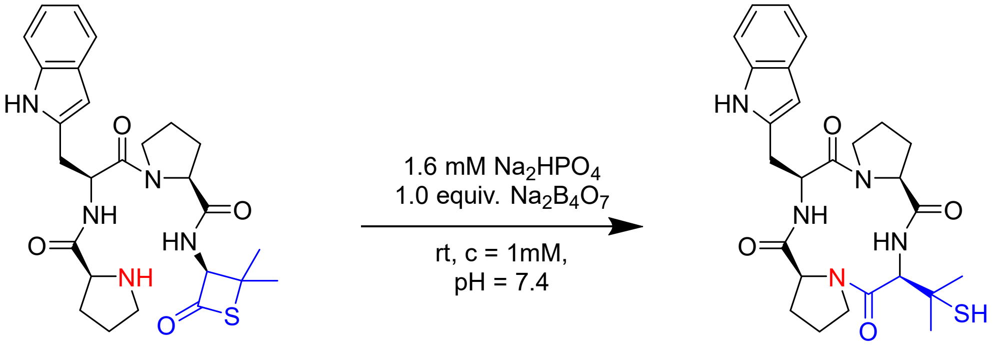

S14j **14j**

According to the general procedure D, **S14j** (5.00 mg, 0.0098 mmol) was dissolved in 9.8 mL cyclization buffer with $Na_2B_4O_7$ (pH = 7.4). After 4h at room temperature, the cyclization reaction was complete (checked by LC-MS). Purification of the crude peptide using preparative HPLC (20 to 60% solvent B over 20 min, Higgins Analytical Proto 200 5 µm 250 × 10 nm C18 column) yielded **14j** as a white solid after lyophilization (0.62 mg, 62%).

Analytical HPLC: t_R = 15.98 min (20 to 80% solvent B over 20 min, Higgins Analytical Proto 200 5 µm 150 × 2.0 nm C18 column).
ESI-LRMS: calcd. for Exact Mass: $C_{26}H_{34}N_5O_4S$ $[M+H]^+$: 512.2326 (*m/z*); found $[M+H]^+$: 512.2575.

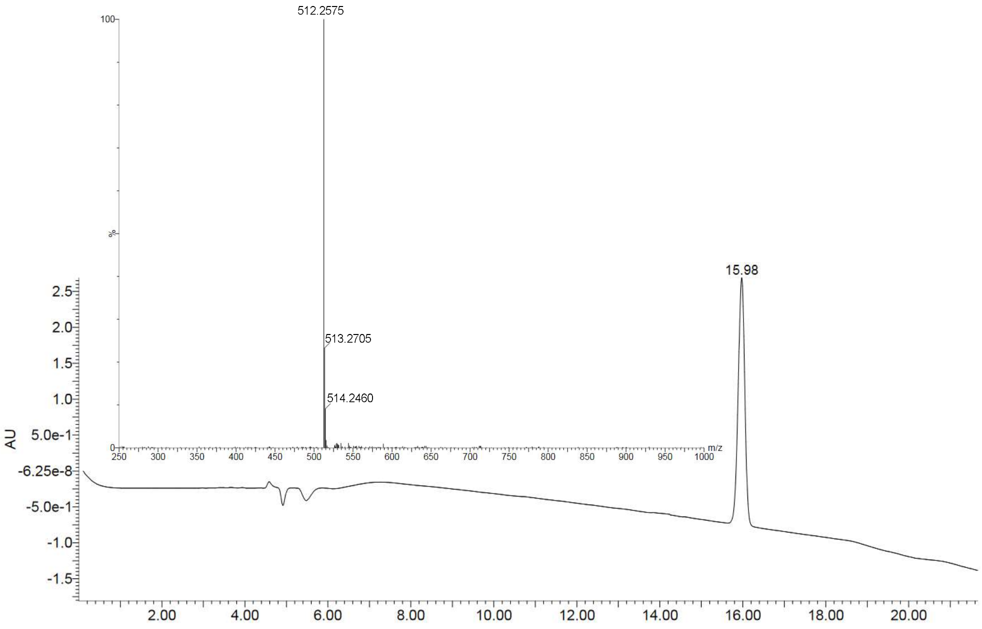

cyclo-tetrapeptides 15j

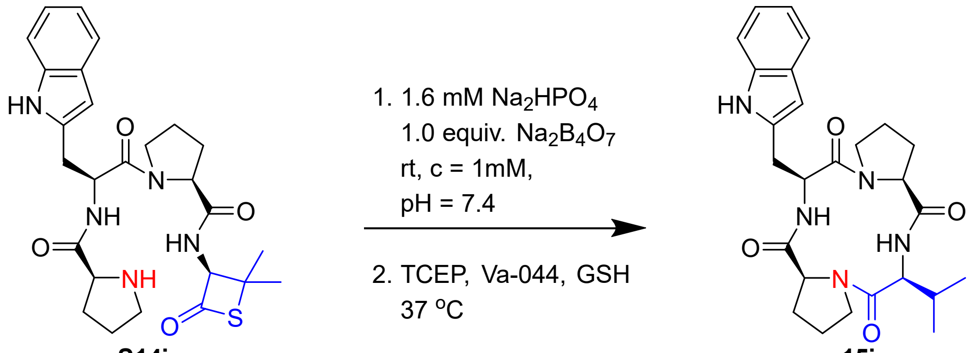

S14j **15j**

According to the general procedure D and E, **S14j** (5.00 mg, 0.0098 mmol) was dissolved in 9.8 mL cyclization buffer with $Na_2B_4O_7$ (pH = 7.4). After the cyclization (4h) and desulfurization (1h) one pot reaction was complete (checked by LC-MS for each step), purification of the crude cyclo-tetrapeptide using preparative HPLC (20 to 60% solvent B over 20 min, Higgins Analytical Proto 200 5 µm 250 × 10 nm C18 column) yielded **15j** as a white solid after lyophilization (2.55 mg, 54% for two steps).

Analytical HPLC: t_R = 14.68 min (20 to 80% solvent B over 20 min, Higgins Analytical Proto 200 5 µm 150 × 2.0 nm C18 column).
^{1}H NMR (600 MHz, DMSO-*d6*) δ 10.70 (s, 1H), 8.06 – 8.04 (d, *J* = 9.8 Hz, 1H), 7.86 – 7.84 (d, *J* = 9.7 Hz, 1H), 7.51 – 7.50 (d, *J* = 7.8 Hz, 1H), 7.28 – 7.26 (d, *J* = 8.0 Hz, 1H), 7.06 – 7.05 (d, *J* = 1.8 Hz, 1H), 7.02 – 7.00 (m, 1H), 6.95 – 6.93 (t, *J* = 7.2 Hz, 1H), 4.98 – 4.94 (td, *J* = 9.1, 5.7 Hz, 1H), 4.41 – 4.38 (dd, *J* = 9.6, 4.9 Hz, 1H), 4.24 – 4.23 (d, *J* = 7.8 Hz, 1H), 4.06 – 4.04 (d, *J* = 8.1 Hz, 1H), 3.54 – 3.49 (td, *J* = 11.6, 7.6 Hz, 1H), 3.23 – 3.17 (m, 2H), 2.98 – 2.95 (m, 1H), 2.83 – 2.79 (dd, *J* = 15.0, 9.0 Hz, 1H), 2.12 – 2.07 (m, 1H), 2.04 – 2.01 (m, 1H), 1.98 – 1.94 (m, 1H), 1.90 – 1.85 (m, 1H), 1.79 – 1.72 (m, 2H), 1.66 – 1.63 (dd, J = 12.0, 6.4 Hz, 1H), 1.55 – 1.49 (m, 1H), 1.47 – 1.42 (m, 1H), 0.81 – 0.80 (d, *J* = 6.7 Hz, 3H), 0.78 – 0.77 (d, *J* = 6.7 Hz, 3H).

^{13}C NMR (151 MHz, DMSO-*d6*) δ 171.58, 171.55, 170.86, 170.75, 136.32, 128.38, 123.79, 121.08, 118.58, 118.51, 111.57, 110.66, 61.60, 61.45, 55.14, 51.00, 47.10, 46.72, 31.85, 31.29, 29.14, 27.46, 22.04, 21.10, 20.99, 17.97.

ESI-LRMS: calcd. for Exact Mass: $C_{26}H_{34}N_5O_4$ [M+H]$^+$: 480.2605 (*m/z*); found [M+H]$^+$: 480.2662.

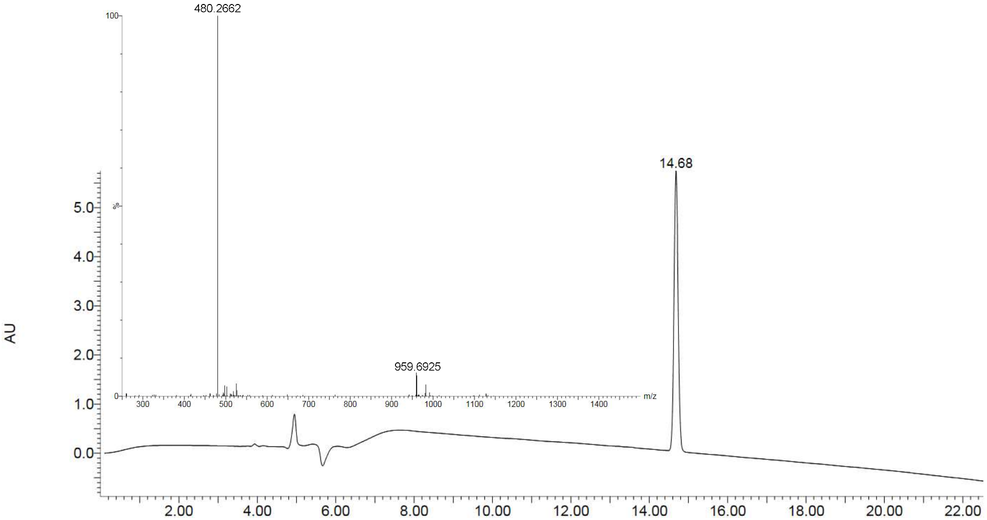

cyclo-tetrapeptides 14k

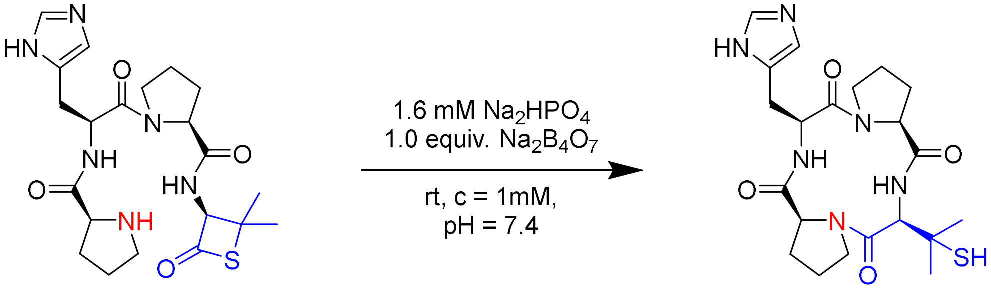

S14k **14k**

According to the general procedure E, **S14k** (5.00 mg, 0.011 mmol) was dissolved in 11.0 mL cyclization buffer with $Na_2B_4O_7$ (pH = 7.4). After 4h at room temperature, the cyclization reaction was complete (checked by LC-MS). Purification of the crude peptide using preparative HPLC (20 to 50% solvent B over 20 min, Higgins Analytical Proto 200 5 µm 250 × 10 nm C18 column) afforded peptide **14k** as a white solid after lyophilization (0.52 mg, 52%).

Analytical HPLC: t_R = 10.12 min (20 to 60% solvent B over 20 min, Higgins Analytical Proto 200 5 µm 150 × 2.0 nm C18 column).

^1H NMR (500 MHz, DMSO-d_6) δ 14.24 (s, 2H), 8.95 (s, 1H), 8.30 (d, *J* = 9.4 Hz, 1H), 8.09 (d, *J* = 9.4 Hz, 1H), 7.38 (s, 1H), 5.10 – 4.95 (m, 1H), 4.64 (d, *J* = 9.5 Hz, 1H), 4.28 – 4.17 (m, 2H), 3.55 – 3.50 (m, 2H), 3.28 (dd, *J* = 11.7, 8.6 Hz, 1H), 3.17 (t, *J* = 10.3 Hz, 1H), 3.09 (dd, *J* = 15.4, 5.1 Hz, 1H), 3.01 (s, 1H), 2.78 (dd, *J* = 15.4, 9.3 Hz, 1H), 2.03 (q, *J* = 4.8, 4.2 Hz, 2H), 1.95 – 1.89 (m, 2H), 1.75 (dd, *J* = 12.0, 5.7 Hz, 2H), 1.63 (td, *J* = 11.6, 5.6 Hz, 1H), 1.37 (s, 3H), 1.31 (d, *J* = 13.1 Hz, 1H), 1.24 (s, 3H).

^{13}C NMR (126 MHz, DMSO-d_6) δ 171.59, 171.14, 170.54, 168.94, 133.94, 130.43, 117.36, 61.63, 61.49, 57.79, 50.13, 47.30, 46.83, 46.72, 32.23, 31.57, 30.56, 29.58, 27.52, 22.21, 21.34.

ESI-LRMS: calcd. for Exact Mass: $C_{21}H_{31}N_6O_4S$ [M+H]$^+$: 463.2122 (*m/z*); found [M+H]$^+$: 463.2122.

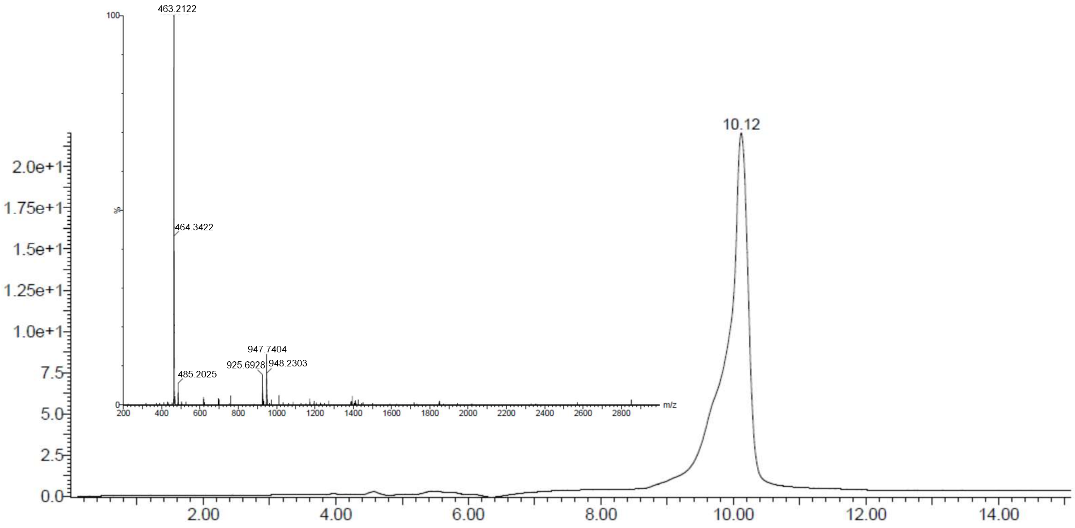

cyclo-tetrapeptides 14l

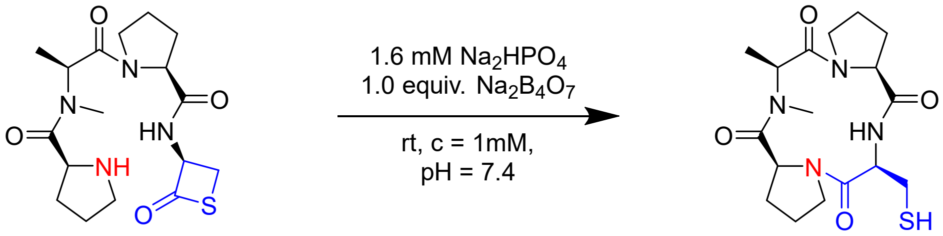

S14l **14l**

According to the general procedure D, **S14l** (5.00 mg, 0.013 mmol) was dissolved in 13.0 mL cyclization buffer with $Na_2B_4O_7$ (pH = 7.4). After 4h at room temperature, the cyclization reaction was complete (checked by LC-MS). Purification of the crude peptide using preparative HPLC (20% to 60% solvent B over 20 min, Higgins Analytical Proto 200 5 µm 250 × 10 nm C18 column) afforded peptide **14l** as a white solid after lyophilization (2.65 mg, 53%).

Analytical HPLC: t_R = 13.47 min (10% to 50% solvent B over 20 min, Higgins Analytical Proto 200 5 µm 150 × 2.0 nm C18 column).

^{1}H NMR (500 MHz, DMSO-*d6*) δ 7.80 (d, *J* = 9.8 Hz, 1H), 4.69 (dt, *J* = 9.7, 6.9 Hz, 1H), 4.36 (d, *J* = 8.2 Hz, 1H), 4.19 (d, *J* = 8.4 Hz, 1H), 3.70 (dd, *J* = 13.2, 6.6 Hz, 1H), 3.65 – 3.56 (m, 1H), 3.48 – 3.42 (m, 1H), 3.27 (dd, *J* = 14.2, 6.6 Hz, 2H), 2.94 (s, 3H), 2.49 – 2.38 (m, 2H), 2.23 – 2.10 (m, 1H), 2.10 – 2.00 (m, 1H), 1.97 – 1.90 (m, 2H), 1.80 (dd, *J* = 11.9, 6.7 Hz, 2H), 1.77 – 1.64 (m, 2H), 1.24 (d, *J* = 6.5 Hz, 3H).

^{13}C NMR (151 MHz, DMSO-*d6*) δ 171.50, 171.40, 170.67, 167.64, 62.02, 59.36, 57.02, 52.73, 48.16, 47.64, 36.22, 32.35, 29.67, 26.29, 22.03, 21.30, 13.29.

ESI-LRMS: calcd. for Exact Mass: $C_{17}H_{27}N_4O_4S$ $[M+H]^+$: 383.1748 (*m/z*); found $[M+H]^+$: 383.1362.

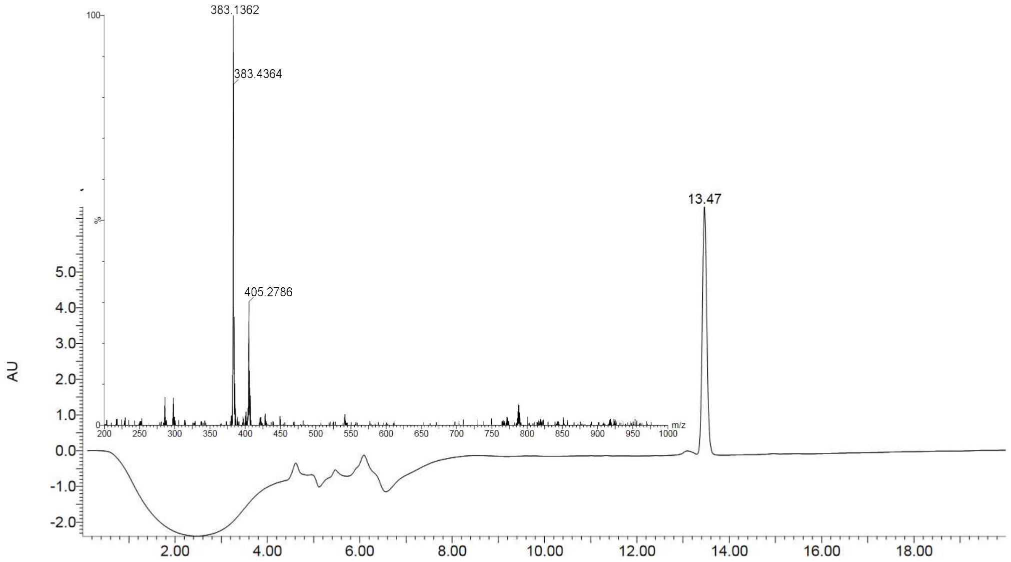

cyclo-tetrapeptides 14m

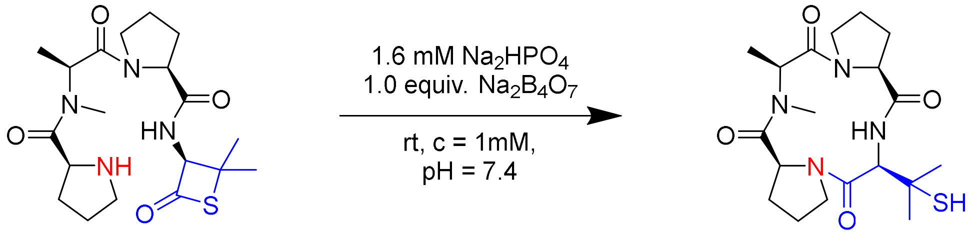

S14m **14m**

According to the general procedure D, **S14m** (5.00 mg, 0.012 mmol) was dissolved in 12.0 mL cyclization buffer with $Na_2B_4O_7$ (pH = 7.4). After 6h at room temperature, the cyclization reaction was complete (checked by LC-MS). Purification of the crude peptide using preparative HPLC (20 to 60% solvent B over 20 min, Higgins Analytical Proto 200 5 µm 250 × 10 nm C18 column) afforded peptide **14m** as a white solid after lyophilization (3.55 mg, 71%).

Analytical HPLC: t_R = 13.56 min (20 to 60% solvent B over 20 min, Higgins Analytical Proto 200 5 µm 150 × 2.0 nm C18 column);
ESI-LRMS: calcd. for Exact Mass: $C_{19}H_{31}N_4O_4S$ $[M+H]^+$: 411.2061 (*m/z*); found $[M+H]^+$: 411.2421.

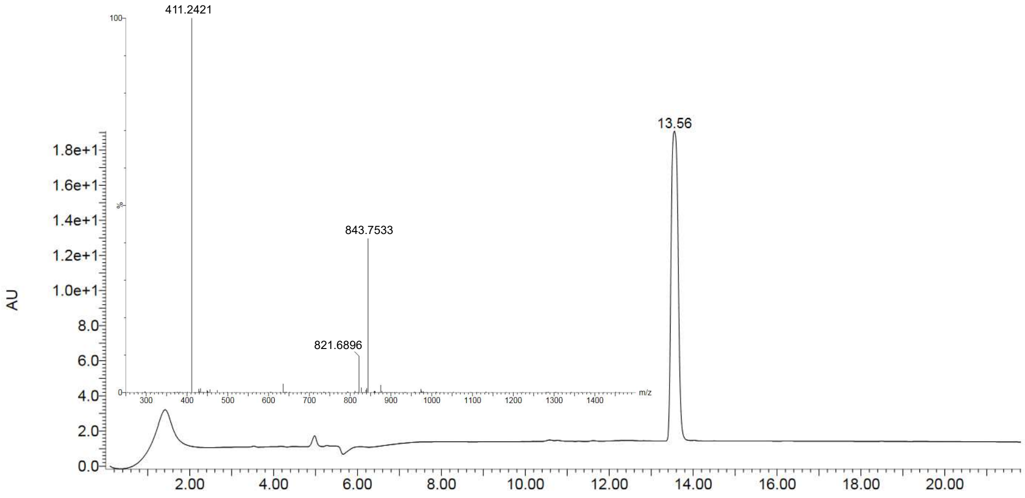

cyclo-tetrapeptides 15m

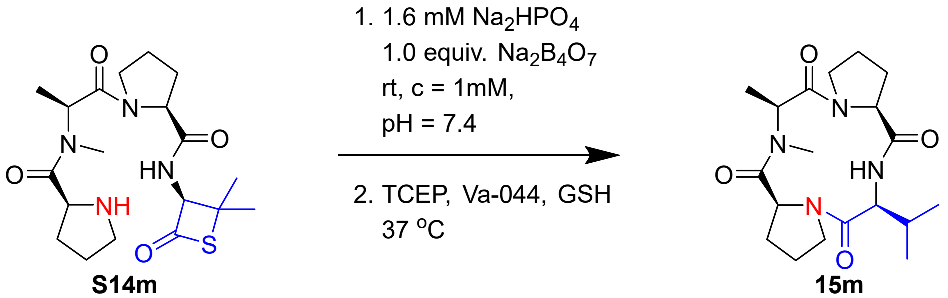

According to the general procedure D and E, **S14m** (5.00 mg, 0.012 mmol) was dissolved in 12.0 mL cyclization buffer with $Na_2B_4O_7$ (pH = 7.4). After the cyclization (6h) and desulfurization (1h) one pot reaction was complete (checked by LC-MS for each step), purification of the crude cyclo-tetrapeptide using preparative HPLC (20 to 60% solvent B over 20 min, Higgins Analytical Proto 200 5 µm 250 × 10 nm C18 column) afforded peptide **15m** as a white solid after lyophilization (1.45 mg, 31% for two steps).

Analytical HPLC: t_R = 6.82 min (30 to 60% solvent B over 20 min, Higgins Analytical Proto 200 5 µm 150 × 2.0 nm C18 column);

^{1} H NMR (600 MHz, DMSO-*d6*) δ 7.47 – 7.45 (d, *J* = 9.7 Hz, 1H), 4.33 – 4.30 (t, *J* = 8.2 Hz, 1H), 4.30 – 4.28 (d, *J* = 7.8 Hz, 1H), 4.14 – 4.13 (d, *J* = 8.8 Hz, 1H), 3.65 – 3.64 (m, 2H), 3,60 – 3.57 (m, 2H), 3.25 – 3.24 (m, 1H), 2.89 (s, 3H), 2.17 – 2.13 (m, 1H), 2.13 – 2.07 (m, 2H), 1.83 – 1.79 (m, 1H), 1.79 – 1.73 (m, 4H), 1.68 – 1.63 (m, 1H), 1.20 – 1.19 (d, *J* = 6.54 Hz, 3H), 0.83 – 0.82 (d, *J* = 6.6 Hz, 3H), 0.73 – 0.72 (d, *J* = 6.8 Hz, 3H).

^{13}C NMR (151 MHz, DMSO-*d6*) δ 171.70, 171.49, 170.92, 167.80, 62.20, 59.29, 56.84, 55.51, 48.22, 47.47, 36.02, 32.35, 29.52, 29.47, 22.06, 21.53, 21.08, 18.30, 13.22.

ESI-LRMS: calcd. for Exact Mass: $C_{19}H_{31}N_4O_4$ $[M+H]^+$: 379.2340 (*m/z*); found $[M+H]^+$: 379.2015.

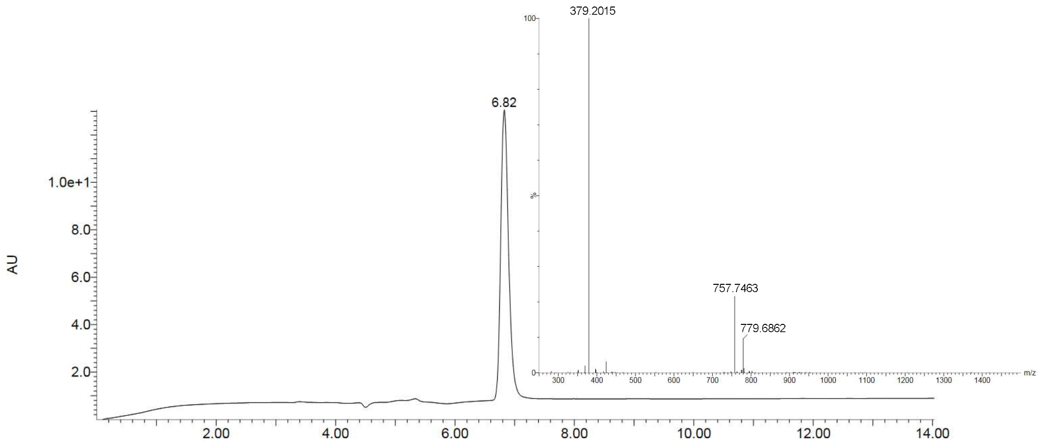

cyclo-tetrapeptides 14o

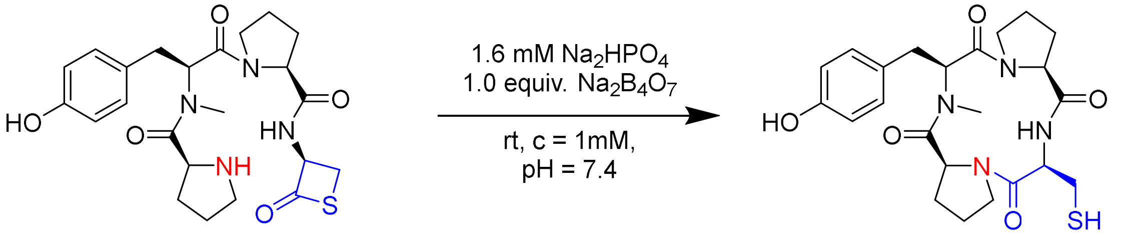

S14o **14o**

According to the general procedure D, **S14o** (5.00 mg, 0.011 mmol) was dissolved in 10.5 mL cyclization buffer with $Na_2B_4O_7$ (pH = 7.4). After 4h at room temperature, the cyclization reaction was complete (checked by LC-MS). Purification of the crude peptide using preparative HPLC (10% to 50% solvent B over 20 min, Higgins Analytical Proto 200 5 µm 250 × 10 nm C18 column) afforded peptide **14o** as a white solid after lyophilization (2.35 mg, 47%).

Analytical HPLC: t_R = 13.66 min (10% to 30% solvent B over 20 min, Higgins Analytical Proto 200 5 µm 150 × 2.0 nm C18 column).

^{1}H NMR (500 MHz, DMSO-*d6*) δ 9.26 (s, 1H), 7.88 (d, *J* = 9.9 Hz, 1H), 6.93 (d, *J* = 8.3 Hz, 2H), 6.69 (d, *J* = 8.4 Hz, 2H), 4.65 (dd, *J* = 16.7, 7.0 Hz, 1H), 4.25 (d, *J* = 8.2 Hz, 1H), 4.19 (d, *J* = 8.6 Hz, 1H), 3.89 (dd, *J* = 10.3, 4.9 Hz, 1H), 3.71 – 3.59 (m, 1H), 3.48 – 3.40 (m, 4H), 3.17 – 3.06 (m, 1H), 3.03 (dd, *J* = 13.8, 4.9 Hz, 1H), 2.99 – 2.87 (m, 1H), 2.29 (s, 3H), 2.05 (dd, *J* = 17.1, 8.8 Hz, 2H), 2.00 – 1.91 (m, 1H), 1.86 (d, *J* = 19.0 Hz, 2H), 1.76 (s, 2H), 1.65 (dd, *J* = 12.3, 6.2 Hz, 1H).

^{13}C NMR (126 MHz, DMSO-*d6*) δ 171.87, 171.35, 170.33, 167.60, 156.36, 130.79 (2C), 129.14, 115.57 (2C), 65.78, 61.76, 57.12, 52.79, 48.35, 47.61, 38.07, 34.10, 32.49, 29.53, 26.23, 21.89, 21.14.

ESI-LRMS: calcd. for Exact Mass: $C_{23}H_{31}N_4O_5S$ $[M+H]^+$: 475.2010 (*m/z*); found $[M+H]^+$:475.2223.

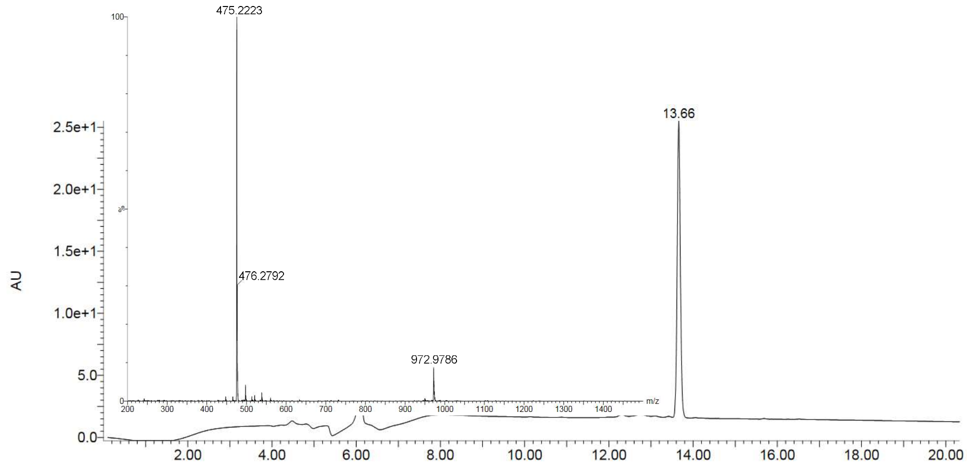

cyclo-tetrapeptides 14p

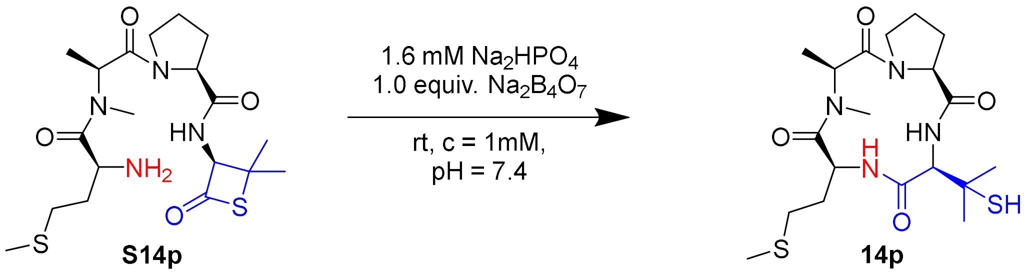

S14p **14p**

According to the general procedure D, **S14p** (5.00 mg, 0.011 mmol) was dissolved in 11.2 mL cyclization buffer with $Na_2B_4O_7$ (pH = 7.4). After 4h at room temperature, the cyclization reaction was complete (checked by LC-MS). Purification of the crude peptide using preparative HPLC (20 to 50% solvent B over 20 min, Higgins Analytical Proto 200 5 µm 250 × 10 nm C18 column) afforded peptide **14p** as a white solid after lyophilization (3.60 mg, 72%).

Analytical HPLC: t_R = 18.52 min (20 to 60% solvent B over 20 min, Higgins Analytical Proto 200 5 µm 150 × 2.0 nm C18 column).
ESI-LRMS: calcd. for Exact Mass: $C_{19}H_{33}N_4O_4S_2$ $[M+H]^+$: 445.1938 (*m/z*); found $[M+H]^+$: 445.1570.

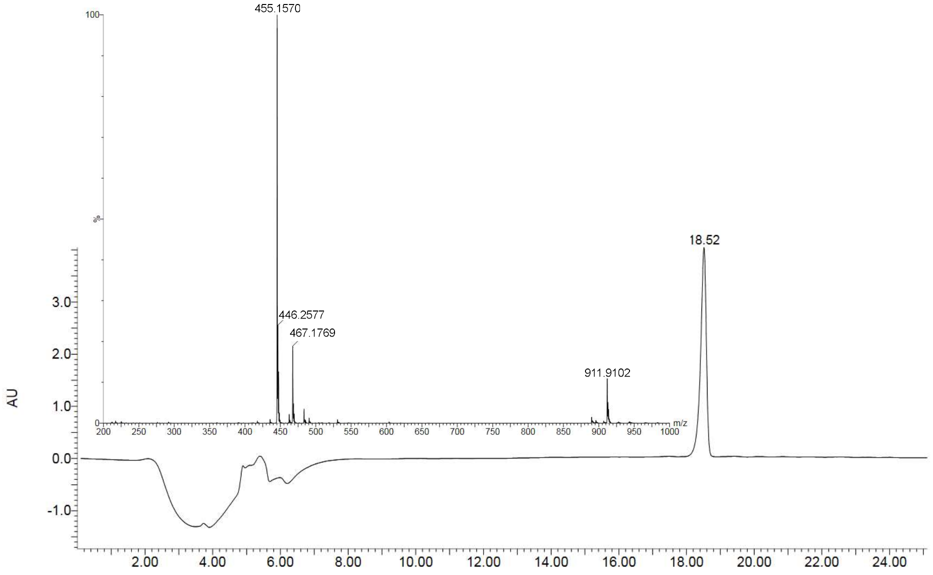

cyclo-tetrapeptides 15p

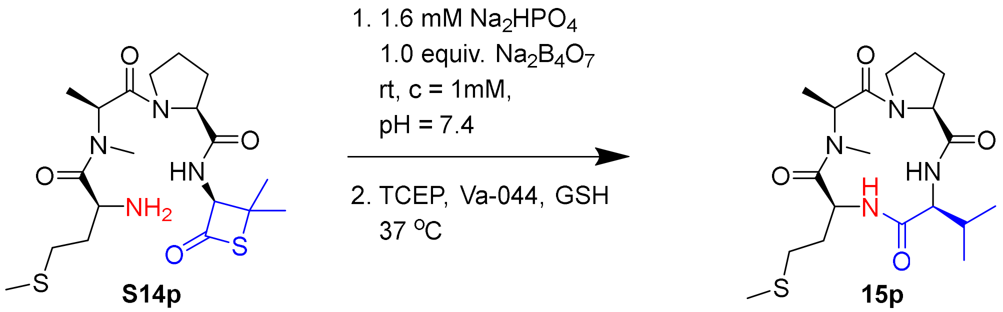

According to the general procedure D and E, **S14p** (5.00 mg, 0.011 mmol) was dissolved in 10.5 mL cyclization buffer with $Na_2B_4O_7$ (pH = 7.4). After the cyclization (4h) and desulfurization (1h) one pot reaction was complete (checked by LC-MS for each step), purification of the crude cyclo-tetrapeptide using preparative HPLC (20 to 50% solvent B over 20 min, Higgins Analytical Proto 200 5 µm 250 × 10 nm C18 column) afforded peptide **15p** as a white solid after lyophilization (0.63 mg, 68% for two steps).

Analytical HPLC: t_R = 15.23 min (20 to 50% solvent B over 20 min, Higgins Analytical Proto 200 C18 5 µm 150 × 2.0 nm column).
^{1}H NMR (500 MHz, DMSO-*d6*) δ 7.49 – 7.47 (d, *J* = 9.5 Hz, 1H), 7.45 – 7.43 (d, *J* = 9.0 Hz, 1H), 4.22 – 4.19 (t, *J* = 9.0 Hz, 1H), 4.17 – 4.16 (d, *J* = 8.5 Hz), 4.09 – 4.06 (td, *J* = 3.-, 3.2 Hz, 1H), 3.69 – 3.63 (m,

1H), 2.94 (s, 3H), 2.58 – 2.54 (m, 2H), 2.16 – 2.13 (m, 1H), 2.07 (s, 3H), 1.93 – 1.91 (m, 1H), 1.84 – 1.82 (m, 2H), 1.79 – 1.68 (m, 3H), 1.23 – 1.22 (d, *J* = 6.5 Hz, 3H), 0.85 – 0.83 (d, *J* = 6.5 Hz, 3H), 0.75 – 0.73 (d, *J* = 6.8 Hz, 3H).

^{13}C NMR (126 MHz, DMSO-*d6*) δ 171.75, 171.69, 171.33, 171.11, 62.10, 59.66, 54.14, 51.27, 48.39, 36.36, 32.36, 30.45, 29.19, 29.03, 22.15, 21.34, 18.36, 15.28, 13.19.

ESI-LRMS: calcd. for Exact Mass: $C_{19}H_{33}N_4O_4S$ $[M+H]^+$: 413.2217 (*m/z*); found $[M+H]^+$: 413.2597.

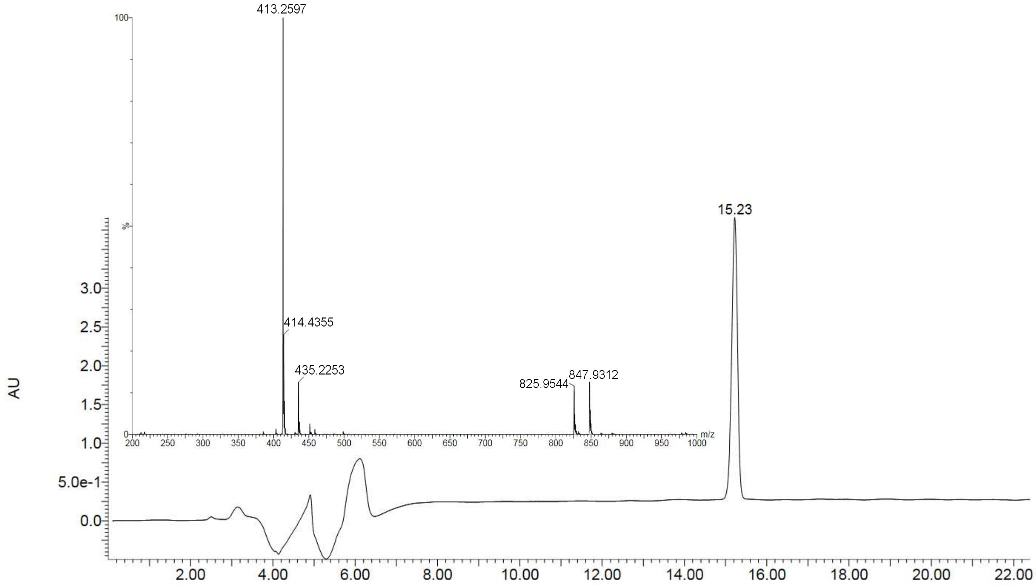

cyclo-tetrapeptides 14q

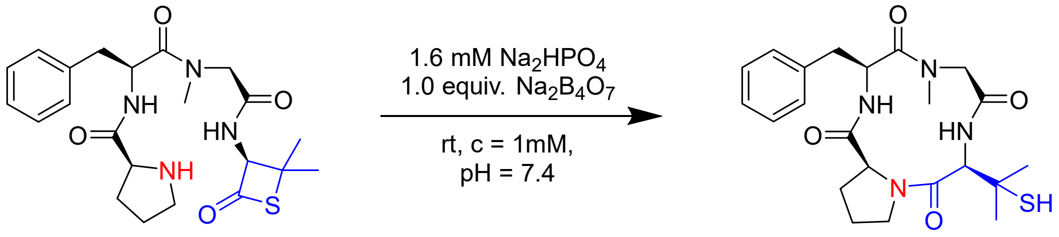

S14q **14q**

According to the general procedure E, **S14q** (5.00 mg, 0.011 mmol) was dissolved in 11.2 mL cyclization buffer with $Na_2B_4O_7$ (pH = 7.4). After 6h at room temperature, the cyclization reaction was complete (checked by LC-MS). Purification of the crude peptide using preparative HPLC (20 to 60% solvent B over 20 min, Higgins Analytical Proto 200 5 µm 250 × 10 nm C18 column) afforded peptide **14q** as a white solid after lyophilization (3.05 mg, 61%).

Analytical HPLC: t_R = 15.98 min (30 to 60% solvent B over 20 min, Higgins Analytical Proto 200 5 µm 150 × 2.0 nm C18 column).

ESI-LRMS: calcd. for Exact Mass: $C_{22}H_{31}N_4O_4S$ $[M+H]^+$: 447.2061 (*m/z*); found $[M+H]^+$: 447.1942.

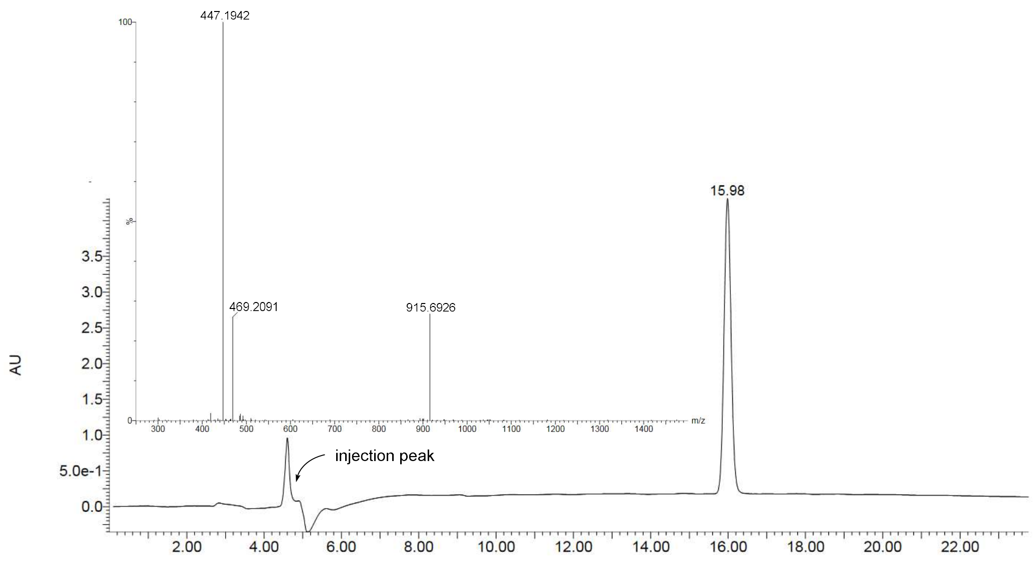

cyclo-tetrapeptides 15q

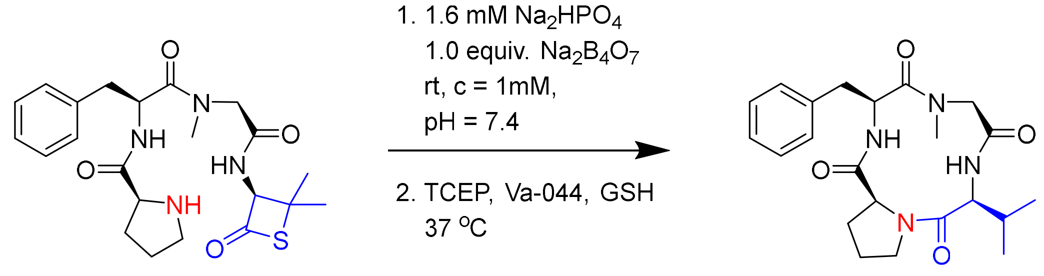

According to the general procedure D and E, **S14q** (5.00 mg, 0.011 mmol) was dissolved in 11.2 mL cyclization buffer with $Na_2B_4O_7$ (pH = 7.4). After the cyclization (4h) and desulfurization (1h) one pot reaction was complete (checked by LC-MS for each step), purification of the crude cyclo-tetrapeptide using preparative HPLC (20 to 60% solvent B over 20 min, Higgins Analytical Proto 200 5 μm 250 × 10 nm C18 column) afforded peptide **15q** as a white solid after lyophilization (2.15 mg, 47% for two steps).

Analytical HPLC: t_R = 16.21 min (20 to 60% solvent B over 20 min, Higgins Analytical Proto 200 5 μm 150 × 2.0 nm C18 column).
^{1}H NMR (600 MHz, DMSO-*d*6) δ 8.00 – 7.99 (d, *J* = 10.1 Hz, 1H), 7.80 – 7.78 (d, *J* = 9.4 Hz, 1H), 7.20 – 7.11 (m, 5H), 5.06 – 5.02 (td, *J* = 5.0, 10.1 Hz, 1H), 4.39 – 4.37 (q, *J* = 4.6 Hz, 1H), 4.04 – 4.01 (d, *J* = 18.3 Hz, 1H), 3.88 – 3.97 (d, *J* = 7.9 Hz, 1H), 3.59 – 3.56 (d, *J* = 18.3 Hz, 1H), 3.42 – 3.37 (td, *J* = 7.5, 11.4 Hz, 1H), 3.13 – 3.10 (dd, *J* = 5.1, 14.2 Hz, 1H), 3.08 – 3.04 (t, *J* = 10.1 Hz, 1H), 2.80 (s, 3H), 2.65 – 2.61 (dd, *J* = 10.1, 13.8 Hz, 1H), 2.11 – 2.08 (m, 1H), 1.79 – 1.72 (m, 1H), 1.60 – 1.55 (m, 1H), 1.13 – 1.11 (m, 1H), 0.84 – 0.83 (d, *J* = 6.7 Hz, 3H), 0.82 – 0.81 (d, 6.7 Hz, 3H).
^{13}C NMR (151 MHz, DMSO-*d*6) δ 172.04, 170.87, 170.74, 168.33, 138.47, 129.96 (2C), 128.16 (2C), 126.40, 61.41, 55.16, 53.18, 50.33, 46.77, 38.18, 35.95, 31.43, 29.19, 21.16, 20.88, 18.06.
ESI-LRMS: calcd. for Exact Mass: $C_{22}H_{31}N_4O_4$ $[M+H]^+$: 415.2340 (*m/z*); found $[M+H]^+$: 415.2325.

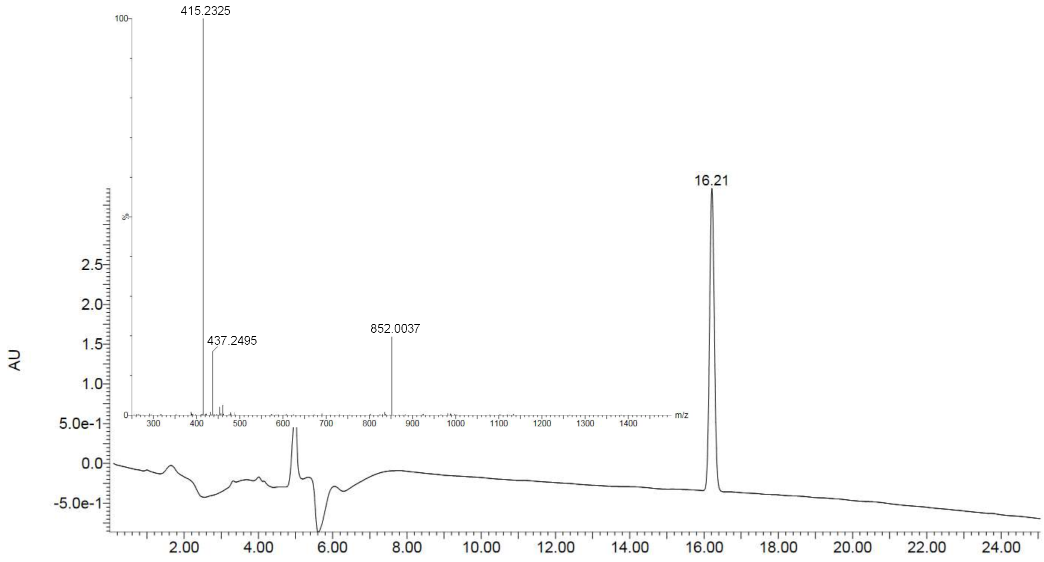

cyclo-tetrapeptides 14t

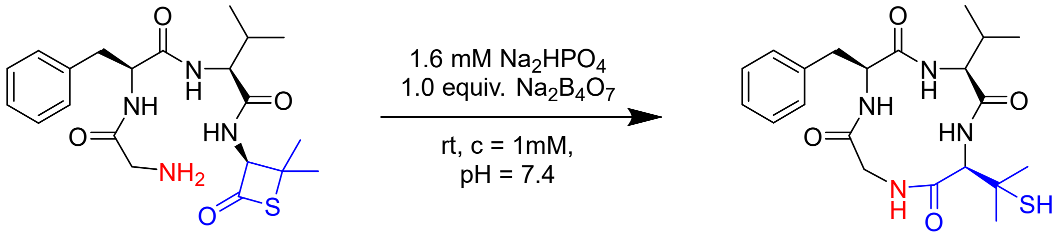

According to the general procedure D, **S14t** (5.00 mg, 0.011 mmol) was dissolved in 11.5 mL cyclization buffer with $Na_2B_4O_7$ (pH = 7.4). After 8h at room temperature, the cyclization reaction was complete (checked by LC-MS). Purification of the crude peptide using preparative HPLC (30 to 80% solvent B over 20 min, Higgins Analytical Proto 200 5 µm 250 × 10 nm C18 column) afforded peptide **14t** as a white solid after lyophilization (2.60 mg, 52%).

Analytical HPLC: t_R = 16.79 min (30 to 80% solvent B over 20 min, Higgins Analytical Proto 200 5 µm 150 × 2.0 nm C18 column).
ESI-LRMS: calcd. for Exact Mass: $C_{21}H_{31}N_4O_4S$ $[M+H]^+$: 435.2061 (*m/z*); found $[M+H]^+$: 435.2055.

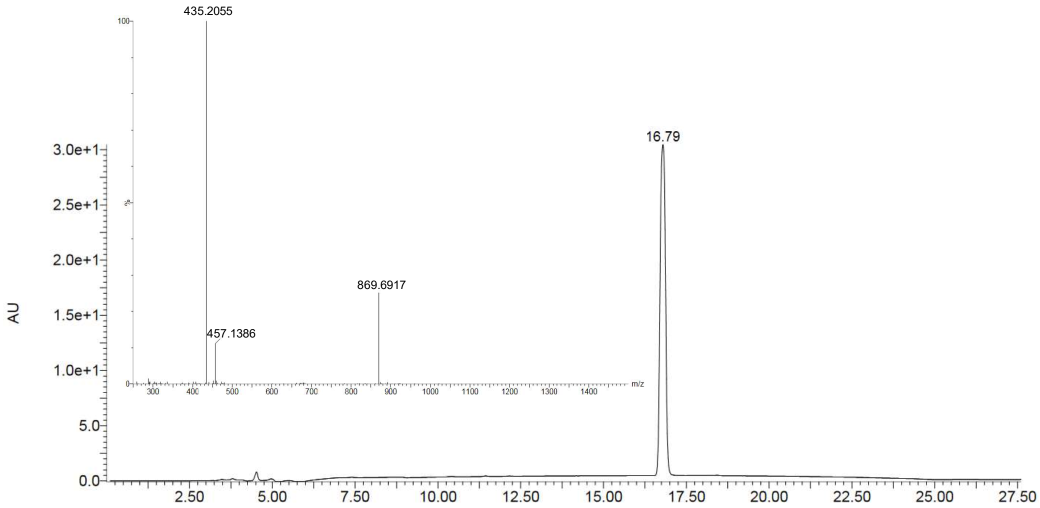

cyclo-tetrapeptides 15t

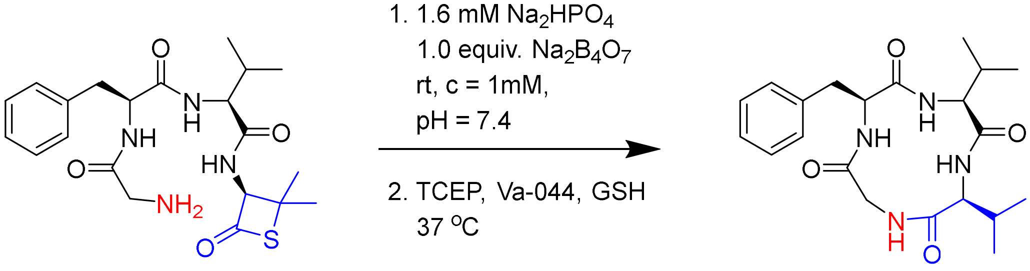

According to the general procedure D and E, **S14t** (5.00 mg, 0.011 mmol) was dissolved in 11.5 mL cyclization buffer with $Na_2B_4O_7$ (pH = 7.4). After the cyclization (8h), one-pot desulfurization could not completely consume the starting material after 4 hours, at this point, the reaction was stopped (checked by LC-MS for each step), purification of the crude cyclo-tetrapeptide using preparative HPLC (30 to 80% solvent B over 20 min, Higgins Analytical Proto 200 5 µm 250 × 10 nm C18 column) afforded peptide **15t** as a white solid after lyophilization (0.85 mg, 18% over two steps with 0.20 mg, 20% **S14t** recovered).

Analytical HPLC: t_R = 14.20 min (30 to 80% solvent B over 20 min, Higgins Analytical Proto 200 5 µm 150 × 2.0 nm C18 column).

^{1}H NMR (600 MHz, DMSO-*d6*) δ 8.49 (s, 1H), 7.77 (s, 1H), 7.43 (s, 1H), 7.32 – 7.23 (m, 2H), 7.20 (d, *J* = 7.5 Hz, 3H), 7.05 (s, 1H), 4.45 (d, *J* = 8.5 Hz, 1H), 4.07 (dd, *J* = 15.9, 8.7 Hz, 1H), 3.96 (s, 1H), 3.80 (t, *J* = 10.1 Hz, 1H), 2.99 (dd, *J* = 13.8, 8.3 Hz, 1H), 2.95 – 2.87 (m, 1H), 1.95 – 1.89 (m, 1H), 1.65 – 1.57 (m, 1H), 1.53 (m, 2H), 0.90 (d, *J* = 6.2 Hz, 3H), 0.85 (d, *J* = 6.5 Hz, 3H), 0.82 (d, *J* = 6.5 Hz, 3H), 0.80 (d, *J* = 6.2 Hz, 3H).

^{13}C NMR (151 MHz, DMSO-*d6*) δ 173.58, 173.03, 171.51, 170.34, 138.65, 129.25 (2C), 128.81 (2C), 127.03, 61.13, 56.94, 54.84, 44.07, 36.65, 28.94, 25.22, 22.97, 22.11, 19.62, 19.18.

ESI-LRMS: calcd. for Exact Mass: $C_{21}H_{31}N_4O_4$ $[M+H]^+$: 403.2340 (*m/z*); found $[M+H]^+$: 403.2976.

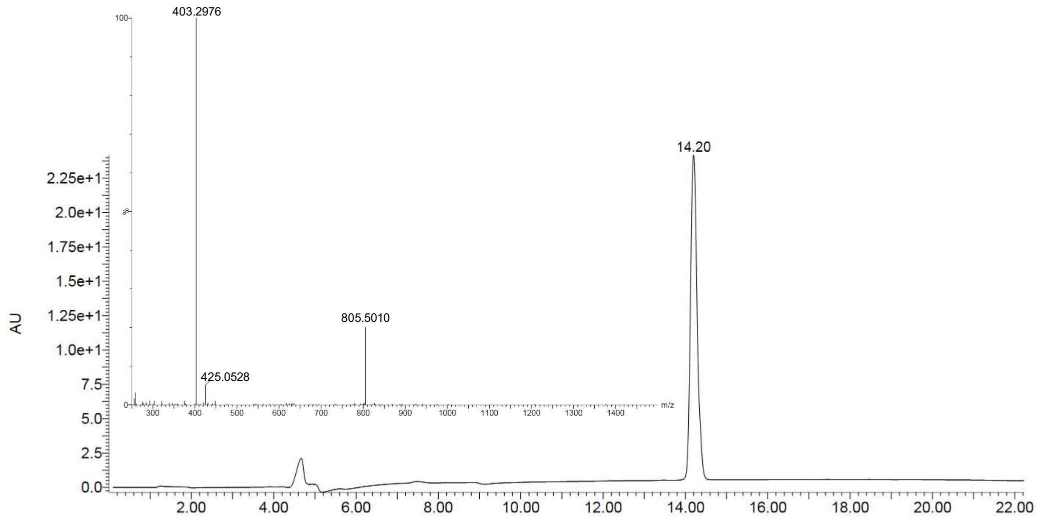

cyclo-tetrapeptides 14u

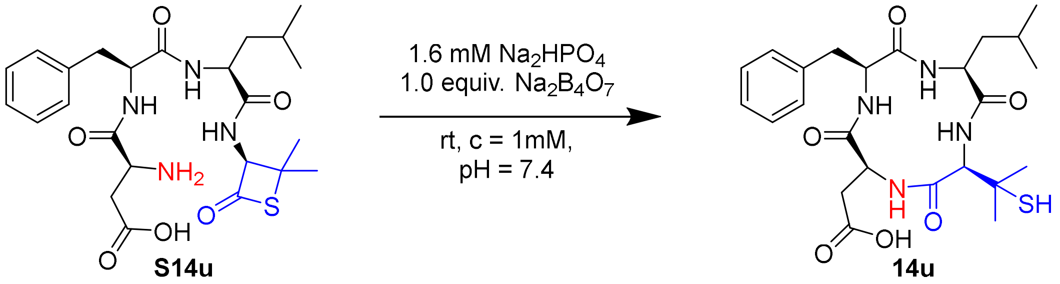

According to the general procedure E, **S14u** (5.00 mg, 0.099 mmol) was dissolved in 9.9 mL cyclization buffer with $Na_2B_4O_7$ (pH = 7.4). After 6h at room temperature, the cyclization reaction was complete (checked by LC-MS). Purification of the crude peptide using preparative HPLC (30 to 60% solvent B over 20 min, Higgins Analytical Pr oto 200 5 µm 250 × 10 nm C18 column) afforded peptide **14u** as a white solid after lyophilization (1.05 mg, 21%).

Analytical HPLC: t_R = 23.80 min (20 to 60% solvent B over 20 min, then 60 to 90% solvent B 20 min to 40 min Higgins Analytical Proto 200 5 µm 150 × 2.0 nm C18 column).

^{1}H NMR (500 MHz, DMSO-*d6*) δ 12.44 (s, 1H), 7.96 (d, *J* = 6.3 Hz, 1H), 7.65 (d, *J* = 8.8 Hz, 1H), 7.57 – 7.55 (m, 1H), 7.51 (d, *J* = 9.7 Hz, 1H), 7.28 – 7.24 (m, 2H), 7.24 – 7.10 (m, 3H), 4.39 (dd, *J* = 12.7, 7.7 Hz, 2H), 4.32 (d, *J* = 9.6 Hz, 1H), 4.19 (q, *J* = 8.8 Hz, 1H), 3.04 (dd, *J* = 13.7, 7.7 Hz, 1H), 2.92 (dd, *J* = 13.6, 8.5 Hz, 1H), 2.55 – 2.54 (m, 2H), 1.59 – 1.53 (m, 2H), 1.50 (dd, *J* = 12.8, 6.6 Hz, 1H), 1.37 (s, 3H), 1.32 (s, 3H), 0.89 (d, *J* = 6.4 Hz, 3H), 0.82 (d, *J* = 6.4 Hz, 3H).

^{13}C NMR (151 MHz, DMSO-*d6*) δ 173.08, 172.41, 171.40, 171.36, 171.21, 137.77, 129.47 (2C), 128.64(2C), 126.90, 62.00, 57.76, 52.33, 51.80, 44.97, 38.62, 36.49, 35.12, 30.55, 28.12, 24.95, 23.28, 22.54.

ESI-LRMS: calcd. for Exact Mass: $C_{24}H_{35}N_4O_6S$ $[M+H]^+$: 507.2272, (*m/z*); found $[M+H]^+$: 507.2682.

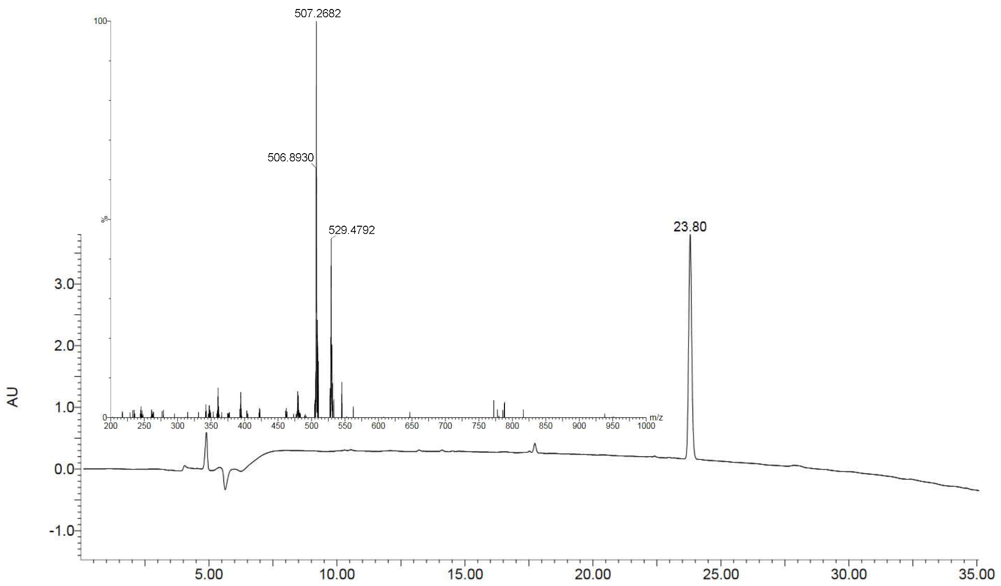

cyclo-pentapeptides S14

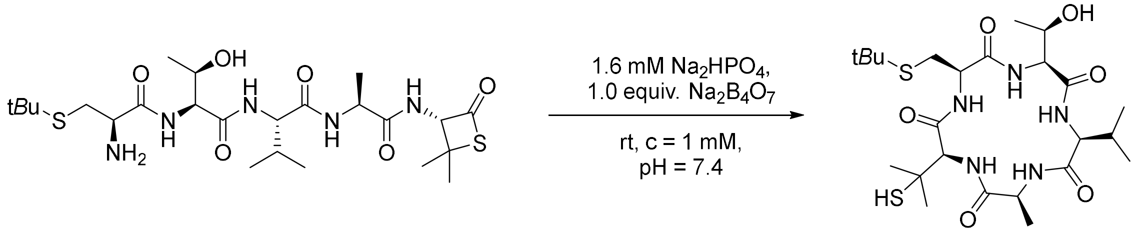

S13 **S14**

According to the general procedure E, **S13** (5.00 mg, 0.0089 mmol) was dissolved in 8.9 mL cyclization buffer with $Na_2B_4O_7$ (pH = 7.4). After 16h at room temperature, the cyclization reaction was complete (checked by LC-MS). Purification of the crude peptide using preparative HPLC (30 to 60% solvent B over 20 min, Higgins Analytical Proto 200 5 µm 250 × 10 nm C18 column) afforded peptide **S14** as a white solid after lyophilization (0.99 mg, 20%).

^{1}H NMR (500 MHz, DMSO-d_6) δ 8.37 (d, *J* = 6.7 Hz, 1H), 8.26 (d, *J* = 8.9 Hz, 1H), 7.95 (t, *J* = 8.4 Hz, 2H), 7.58 (d, *J* = 9.4 Hz, 1H), 6.53 (s, 1H), 5.30 (d, *J* = 4.1 Hz, 1H), 4.50 (d, *J* = 8.9 Hz, 1H), 4.41 (t, *J* = 6.8 Hz, 1H), 4.20 (td, *J* = 9.7, 8.6, 3.1 Hz, 2H), 4.02 – 3.91 (m, 1H), 3.81 (t, *J* = 8.0 Hz, 1H), 2.90 (dd, *J* = 13.2, 5.0 Hz, 1H), 2.86 – 2.78 (m, 2H), 2.16 (q, *J* = 6.8 Hz, 1H), 1.36 (d, *J* = 3.8 Hz, 3H), 1.29 (s, 9H), 1.16 (d, *J* = 6.6 Hz, 3H), 1.08 (d, *J* = 6.2 Hz, 3H), 0.86 (t, *J* = 7.0 Hz, 6H), 0.01 (s, 2H).
^{13}C NMR (126 MHz, DMSO-d_6) δ 172.08, 170.79, 170.43, 170.16, 170.14, 66.26, 59.42, 56.59, 48.51, 45.99, 42.89, 31.17 (3C), 30.35, 30.30, 30.23, 29.16, 20.79, 19.74, 18.70, 17.29.

cyclo-pentapeptides 32a

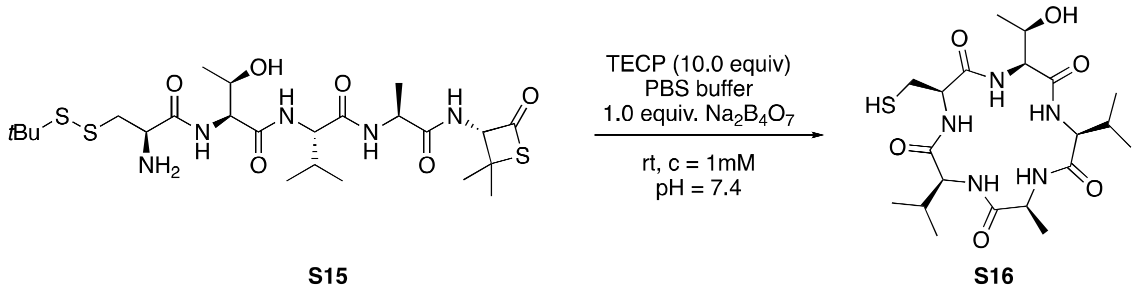

S15 **S16**

According to the general procedure E, **S15** (3.00 mg, 0.005mmol) was dissolved in 5 mL cyclization buffer with $Na_2B_4O_7$ (pH = 7.4). After 6h at room temperature, the cyclization reaction was complete (checked by LC-MS). Purification of the crude peptide using preparative HPLC (30 to 60% solvent B over 20 min, Higgins Analytical Proto 200 5 µm 250 × 10 nm C18 column) afforded peptide **S16** as a white solid after lyophilization (1.1 mg, 39%).

Analytical HPLC: t_R = 12.70 min (25 to 65% solvent B over 20 min, Higgins Analytical Proto 200 5 µm 150 × 2.0 nm C18 column).
^{1}H NMR (500 MHz, DMSO-d_6) δ 8.36 – 8.27 (m, 2H), 7.97 (dd, *J* = 12.3, 9.5 Hz, 2H), 7.36 (d, *J* = 9.2 Hz, 1H), 4.44 (d, *J* = 9.8 Hz, 1H), 4.10 (td, *J* = 7.7, 5.2 Hz, 4H), 4.01 (t, *J* = 9.1 Hz, 2H), 3.16 – 2.97 (m, 3H), 2.91 (s, 1H), 2.65 (t, *J* = 8.2 Hz, 2H), 2.04 (dt, *J* = 8.7, 6.5 Hz, 1H), 1.44 (s, 3H), 1.34 (s, 2H), 1.10 (d, *J* = 6.1 Hz, 3H), 0.89 (dd, *J* = 6.6, 1.7 Hz, 6H), 0.01 (s, 2H).
^{13}C NMR (126 MHz, DMSO-d_6) δ 172.16, 171.15, 170.57, 169.71, 169.57, 66.31, 62.04, 59.88, 59.49, 52.46, 46.81, 30.83, 30.53, 30.29, 25.24, 21.34, 19.78, 18.82, 17.95.
ESI-LRMS: calcd. for Exact Mass: $C_{20}H_{36}N_5O_6S_2$ [M+H]$^+$: 506.2102 (*m/z*); found [M+H]$^+$: 506.2177.

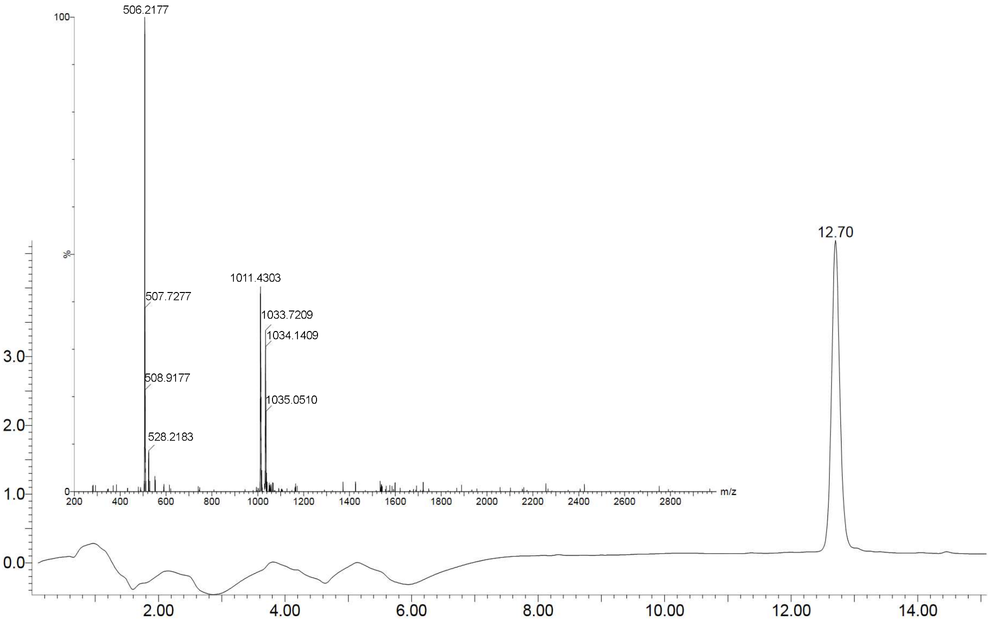

cyclo-pentapeptides S18

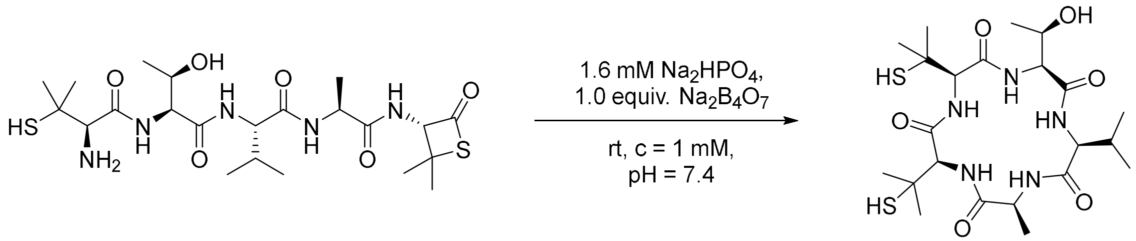

S17 **S18**

According to the general procedure E, **S17** (5.00 mg, 0.0098 mmol) was dissolved in 9.8 mL cyclization buffer with $Na_2B_4O_7$ (pH = 7.4). After 6h at room temperature, the cyclization reaction was complete (checked by LC-MS). Purification of the crude peptide using preparative HPLC (30 to 60% solvent B over

20 min, Higgins Analytical Proto 200 5 µm 250 × 10 nm C18 column) afforded peptide **S18** as a white solid after lyophilization (2.35 mg, 47%).

Analytical HPLC: t_R = 20.64 min (30 to 60% solvent B over 20 min, Higgins Analytical Proto 200 5 µm 150 × 2.0 nm C18 column).

^{1}H NMR (500 MHz, Methanol-d_4) δ 8.57 (dd, *J* = 14.8, 8.8 Hz, 2H), 8.08 (d, *J* = 4.4 Hz, 1H), 8.05 – 7.99 (m, 1H), 7.45 (d, *J* = 9.0 Hz, 1H), 5.37 (d, *J* = 9.7 Hz, 2H), 4.66 (dd, *J* = 7.9, 3.3 Hz, 1H), 4.63 – 4.58 (m, 1H), 4.58 – 4.53 (m, 1H), 3.99 (t, *J* = 4.6 Hz, 1H), 3.23 (t, *J* = 6.9 Hz, 1H), 2.24 (dq, *J* = 13.3, 6.9 Hz, 1H), 2.05 (s, 1H), 1.53 (s, 3H), 1.48 (s, 3H), 1.44 (s, 2H), 1.42 (s, 4H), 1.36 (s, 3H), 1.26 (d, *J* = 6.3 Hz, 3H), 1.05 (dd, *J* = 8.8, 6.9 Hz, 6H), 0.96 (t, *J* = 7.4 Hz, 2H).

^{13}C NMR (126 MHz, Methanol-d_4) δ 171.77, 170.53, 170.21, 169.12, 70.09, 61.28, 61.13, 60.64, 60.56, 57.03, 50.18, 45.89, 30.32, 29.67, 29.21, 28.13, 27.30, 18.93, 18.36, 18.08, 17.01.

ESI-LRMS: calcd. for Exact Mass: $C_{22}H_{40}N_5O_6S_2$ [M+H]$^+$: 534.2415 (*m/z*); found [M+H]$^+$: 534.2416.

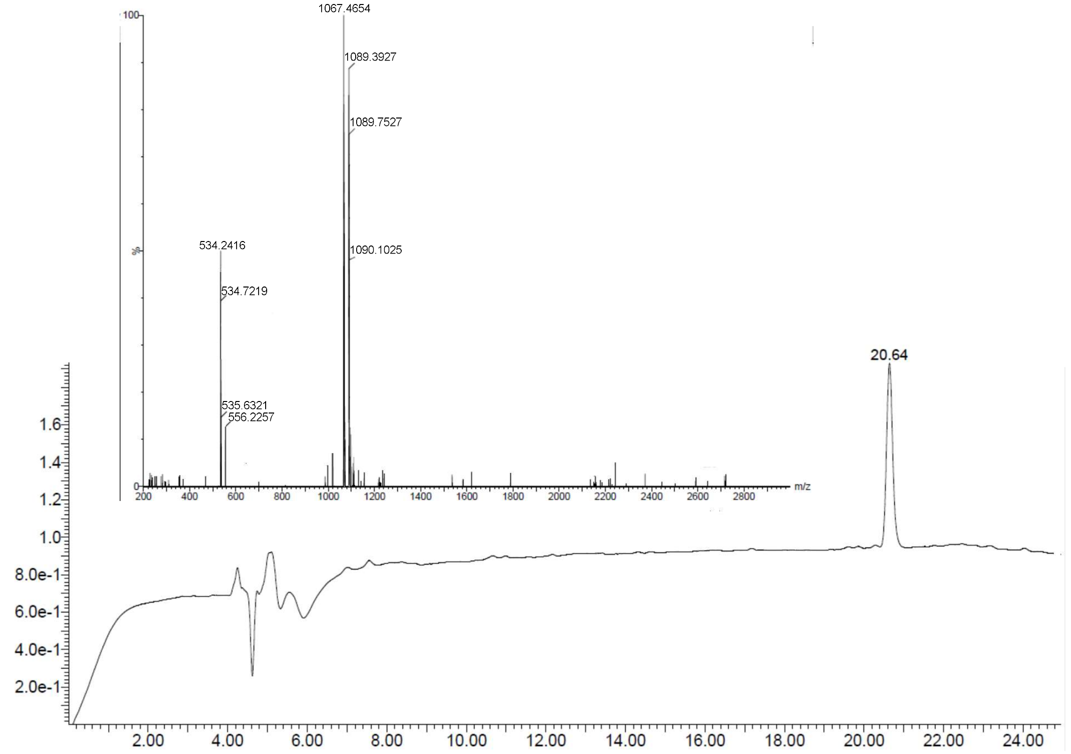

IV.3 Cyclization of sidechain protected tetrapeptides by HATU coupling.

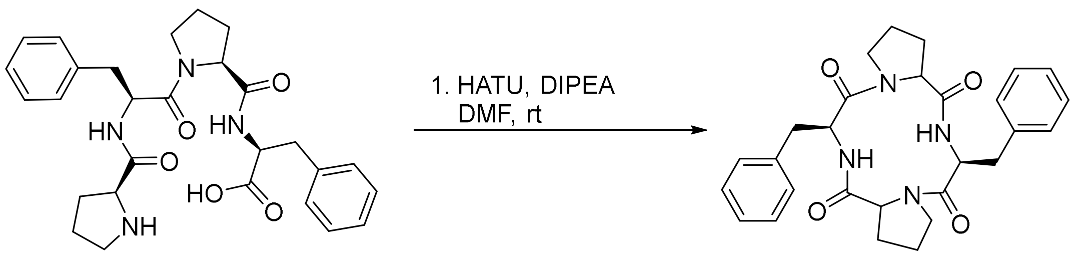

10a **3**

According to general procedure F, **10a** (5 mg, 0.01 mmol) was dissolved in 20 mL DMF, HATU (15.2 mg,

0.04 mmol) and DIPEA (7.0 µL, 0.04 mmol) was subsequently added into the reaction mixture. Then the resulting mixture was allowed stirred at room temperature for 8h before the solvent evaporated. The resulting residue was dissolved into acetonitrile/H_2O (1/1, V/V) and purified through preparation HPLC after filtration.

Purification of the crude peptide using preparative HPLC (30 to 90% solvent B over 20 min, Higgins Analytical Proto 200 5 µm 250 × 10 nm C18 column) afforded peptide **3** as a white solid after lyophilization (2.0 mg, 43%).

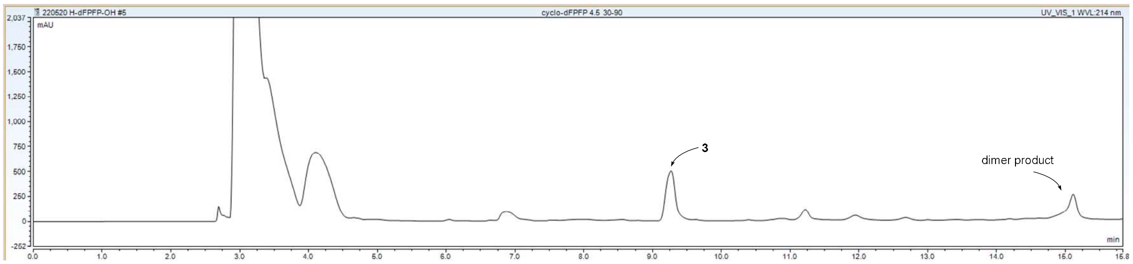

The crude prep-HPLC trace of cyclization from **10a** to **3** by HATU coupling.

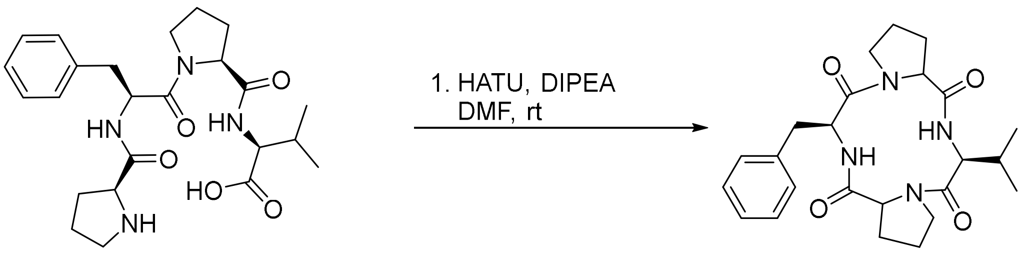

10b **11b**

According to general procedure F, **10b** (4.6 mg, 0.01 mmol) was dissolved in 20 mL DMF, HATU (15.2 mg, 0.04 mmol) and DIPEA (7.0 µL, 0.04 mmol) was subsequently added into the reaction mixture. Then the resulting mixture was allowed stirred at room temperature for 8h before the solvent evaporated. The resulting residue was dissolved into acetonitrile/H_2O (1/1, V/V) and purified through preparation HPLC after filtration.

Purification of the crude peptide using preparative HPLC (20 to 80% solvent B over 20 min, Higgins Analytical Proto 200 5 µm 250 × 10 nm C18 column) afforded peptide **11b** as a white solid after lyophilization (1.4 mg, 33%).

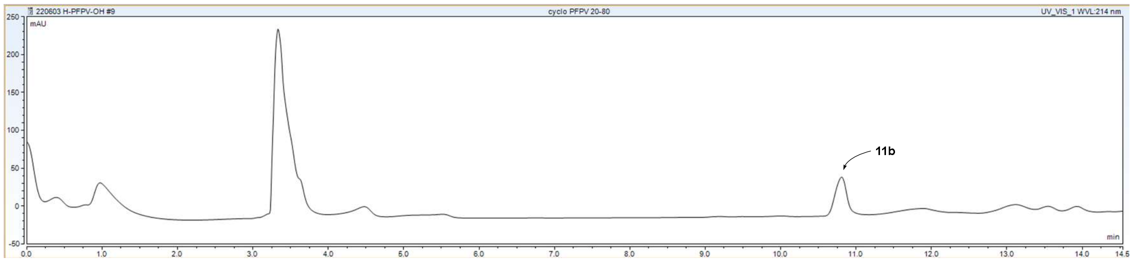

The crude prep-HPLC trace of cyclization from **10b** to **11b** by HATU coupling.

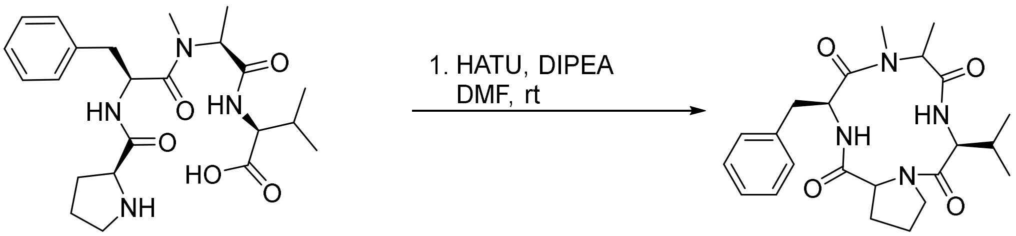

10c **11c**

According to general procedure F, **10c** (4.5 mg, 0.01 mmol) was dissolved in 20 mL DMF, HATU (15.2 mg, 0.04 mmol) and DIPEA (7.0 μL, 0.04 mmol) was subsequently added into the reaction mixture. Then the resulting mixture was allowed stirred at room temperature for 8h before the solvent evaporated. The resulting residue was dissolved into acetonitrile/H_2O (1/1, V/V) and purified through preparation HPLC after filtration.

Purification of the crude peptide using preparative HPLC (30 to 90% solvent B over 20 min, Higgins Analytical Proto 200 5 µm 250 × 10 nm C18 column) afforded peptide **11c** as a white solid after lyophilization (2.0 mg, 48%).

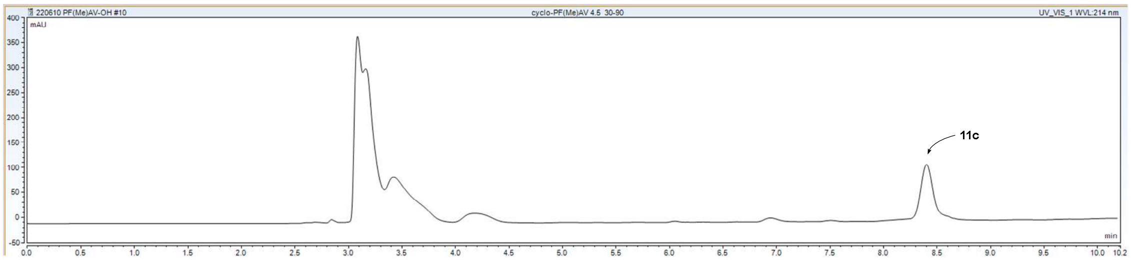

The crude prep-HPLC trace of cyclization from **10c** to **11c** by HATU coupling.

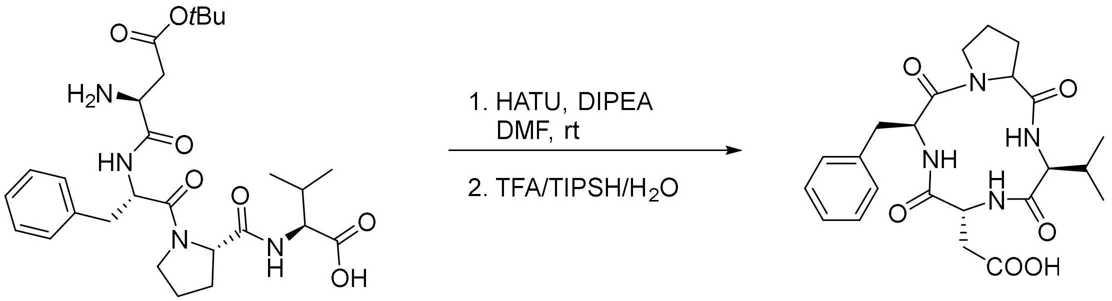

10d **11d**

According to general procedure F, **10d** (5.3 mg, 0.01 mmol) was dissolved in 20 mL DMF, HATU (15.2 mg, 0.04 mmol) and DIPEA (7.0 μL, 0.04 mmol) was subsequently added into the reaction mixture. Then the resulting mixture was allowed stirred at room temperature for 8h before the solvent evaporated. The resulting residue was added TFA/TIPSH/H_2O (95/2.5/2.5, V/V/V) 3 mL and stirred for 20 min. Then the solvent was blown out by argon stream and dissolved into acetonitrile/H_2O (1/1, V/V) and purified through preparation HPLC after filtration.

Purification of the crude peptide using preparative HPLC (20 to 60% solvent B over 20 min, Higgins Analytical Proto 200 5 µm 250 × 10 nm C18 column) afforded peptide **11d** as a white solid after lyophilization (0.4 mg, 10%).

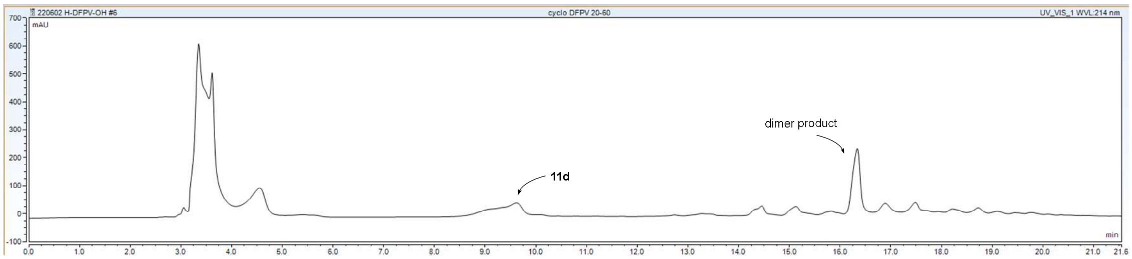

The crude prep-HPLC trace of cyclization from **10d** to **11d** by HATU coupling.

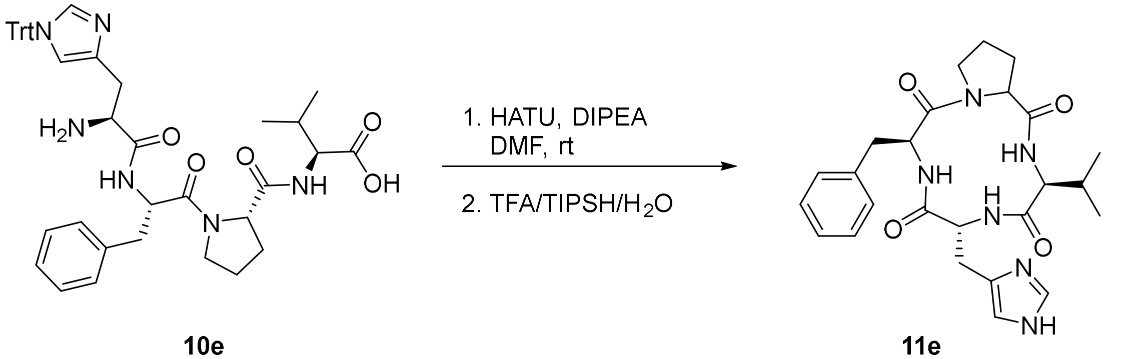

10e **11e**

According to general procedure F, **10e** (7.4 mg, 0.01 mmol) was dissolved in 20 mL DMF, HATU (15.2 mg, 0.04 mmol) and DIPEA (7.0 μL, 0.04 mmol) was subsequently added into the reaction mixture. Then the resulting mixture was allowed stirred at room temperature for 8h before the solvent evaporated. The resulting residue was added TFA/TIPSH/H_2O (95/2.5/2.5, V/V/V) 3 mL and stirred for 20 min. Then the solvent was blown out by argon stream and dissolved into acetonitrile/H_2O (1/1, V/V) and purified through preparation HPLC after filtration.

Purification of the crude peptide using preparative HPLC (30 to 90% solvent B over 20 min, Higgins Analytical Proto 200 5 µm 250 × 10 nm C18 column) afforded peptide **11e** as a white solid after lyophilization (0.6 mg, 12%).

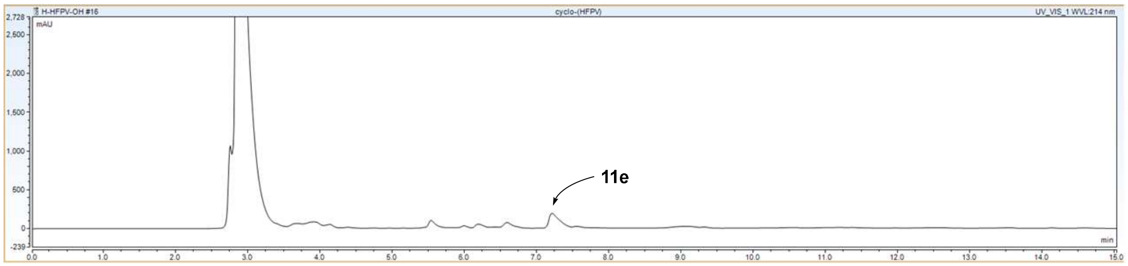

The crude prep-HPLC trace of cyclization from **10e** to **11e** by HATU coupling.

2. Supplementary Discussion

2.1 Thiolactone Mediated Cyclization Compared to Extant Protocols.

2.1.1 The crude cyclization reaction HPLC trace of tetracyclic Natural Products.

2.1.1.1 The crude reaction HPLC trace of cyclization from Pen-β-thiolactone S14a to cPYPV 1

Cyclization of thiolactone bearing linear peptide smoothly afforded cyclo-tetrapeptide without epimerization. the following HPLC traces depict a typical ring closure reaction. At room temperature, cyclization of **14a** (crude HPLC trace Supplementary Figure 5) was completed after 4h and the reaction product **14a** as shown in Supplementary Figure 6. The product **1** was generated after in situ desulfurization and produced **1** (yield = 57% over two steps) was obtained via HPLC purification (Supplementary Figure 7).

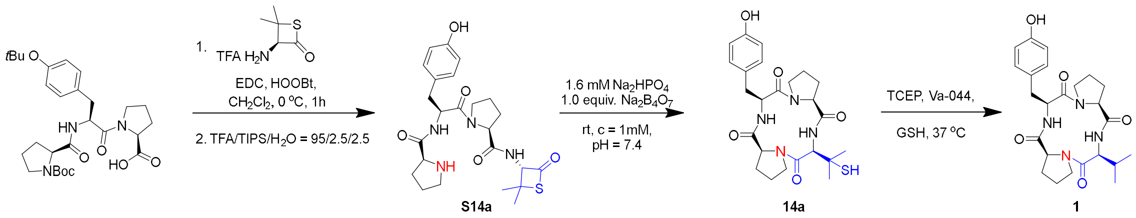

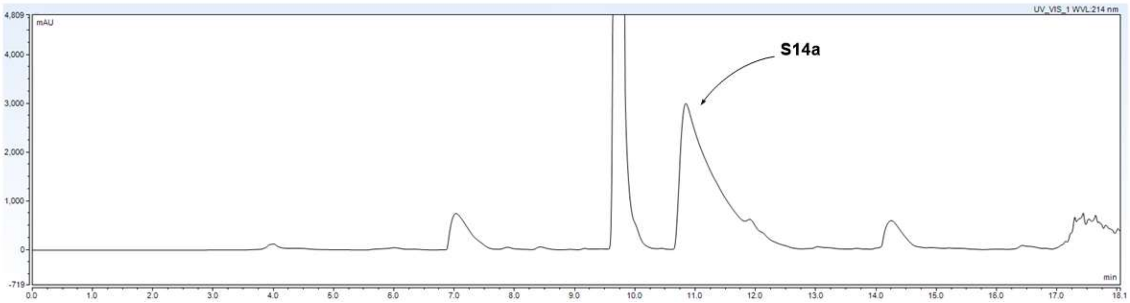

Supplementary Figure 5, the crude prep-HPLC trace of **S14a** (t_R = 10.84 min, 10 to 60% solvent B over 20 min)

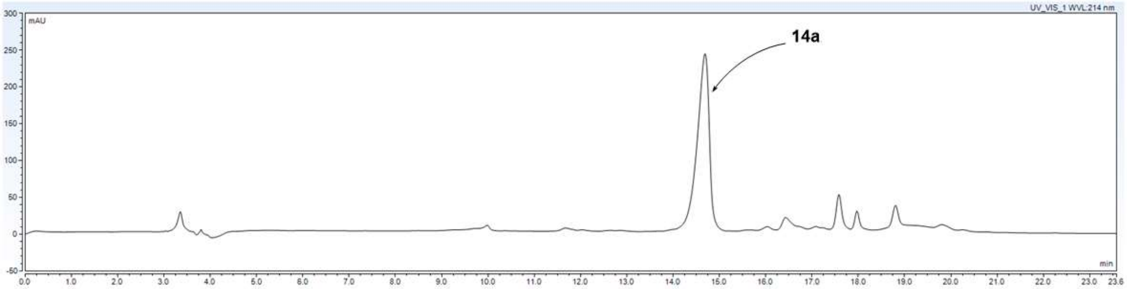

Supplementary Figure 6, the crude prep-HPLC trace of **14a** (t_R = 14.73 min, 10 to 60% solvent B over 20 min)

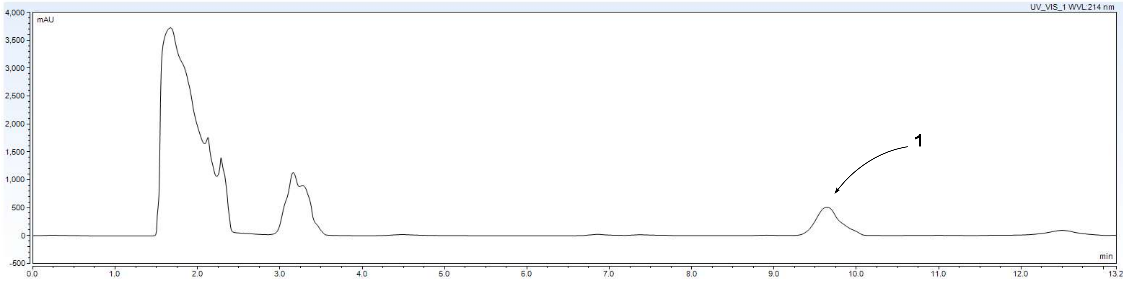

Supplementary Figure 7, the crude prep-HPLC trace of **1** (t_R = 9.64 min, 20 to 60% solvent B over 20 min)

2.1.1.2 The crude reaction HPLC trace of cyclization from Leu-β-thiolactone S9 to cPLPL 2

At room temperature, cyclization of **S2a** (crude HPLC trace Supplementary Figure 8) was completed after 4h and the reaction product **2a** as shown in Supplementary Figure 9. The product **2** was generated after in situ desulfurization and produced **2** (yield = 43% over two steps) was obtained via HPLC purification (Supplementary Figure 10).

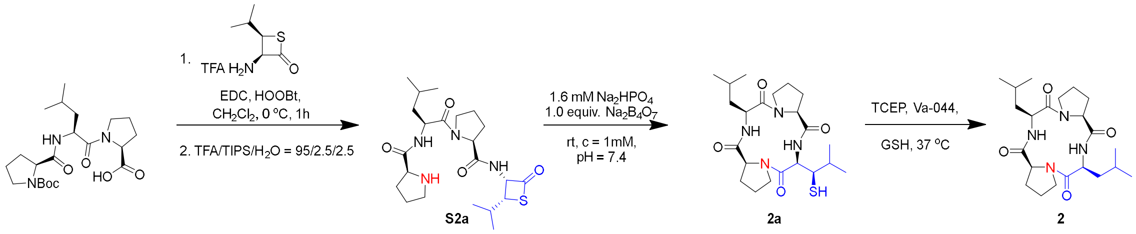

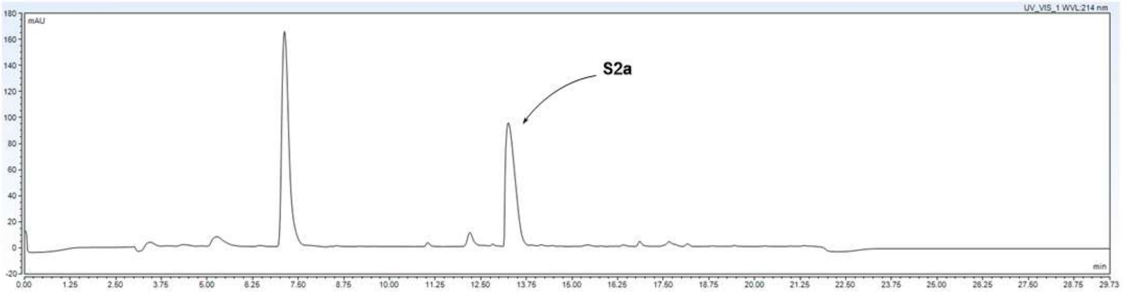

Supplementary Figure 8, the crude prep-HPLC trace of **S2a** (t_R = 12.82 min, 20 to 50% solvent B over 20 min)

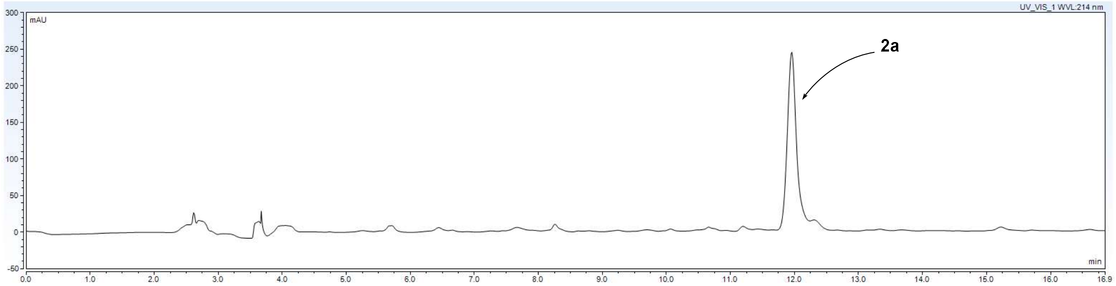

Supplementary Figure 9, the crude prep-HPLC trace of **2a** (t_R = 12.30 min, 30 to 70% solvent B over 20 min)

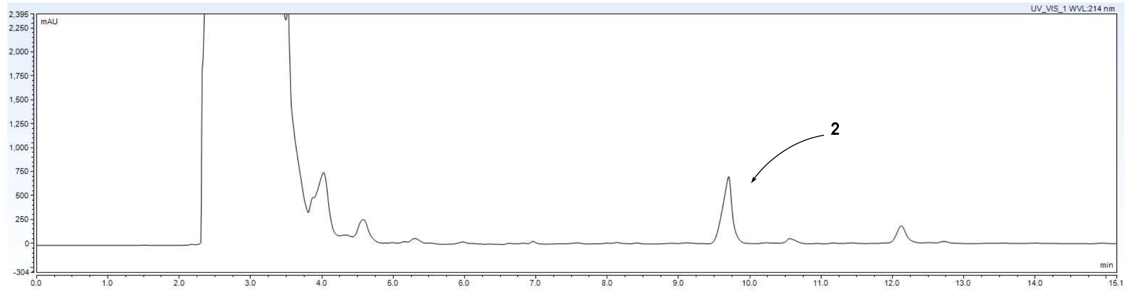

Supplementary Figure 10, the crude prep-HPLC trace of **2** (t_R = 9.70 min, 30 to 70% solvent B over 20 min)

2.1.1.3 The crude reaction HPLC trace of cyclization from Phe-β-thiolactone S9 to cPFPF 3

At room temperature, cyclization of **S9** (crude HPLC trace Supplementary Figure 11) was completed after 4h and the reaction product **9a** as shown in Supplementary Figure 12. The product **3** was generated after in situ desulfurization and produced **3** (yield = 49% over two steps) was obtained via HPLC purification (Supplementary Figure 13).

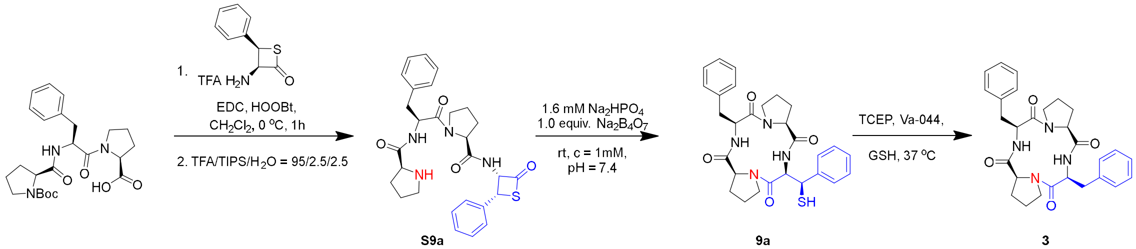

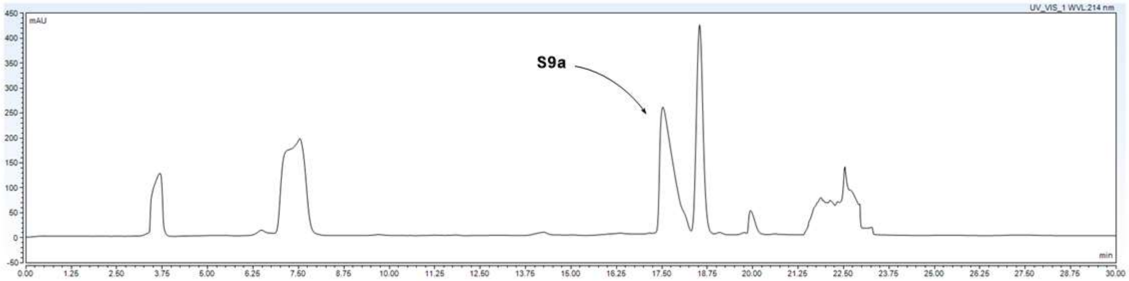

Supplementary Figure 11, the crude prep-HPLC trace of **S9a** (t_R = 17.52 min, 20 to 50% solvent B over 20 min)

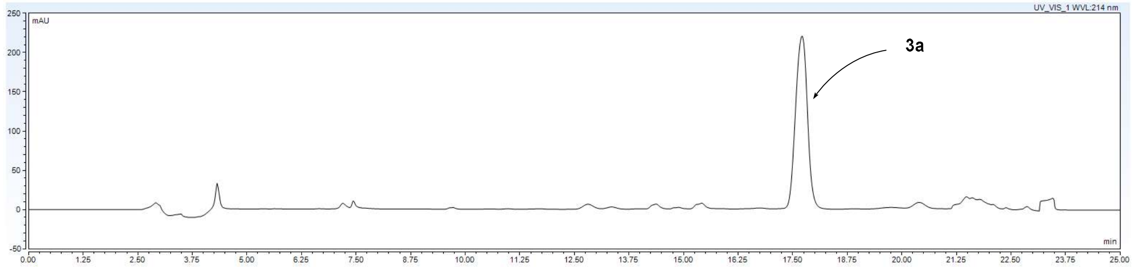

Supplementary Figure 12, the crude prep-HPLC trace of **2a** (t_R = 17.72 min, 30 to 80% solvent B over 20 min)

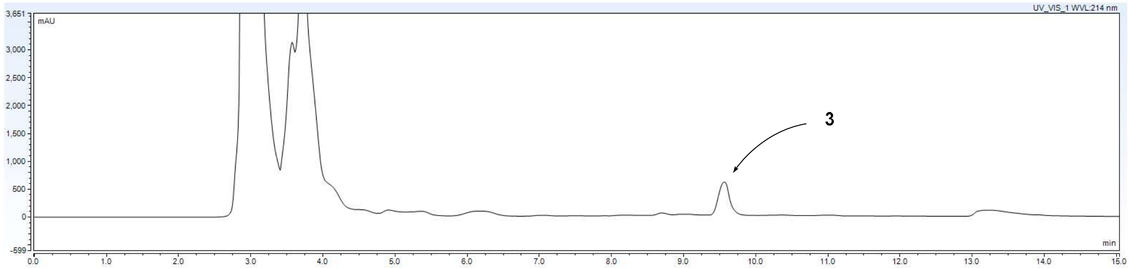

Supplementary Figure 13, the crude prep-HPLC trace of **3** (t_R = 9.59 min, 30 to 90% solvent B over 20 min)

2.1.1.4 The crude reaction HPLC trace of cyclization from Pen-β-thiolactone S4a to cPVPV 4

At room temperature, cyclization of **S4a** (crude HPLC trace Supplementary Figure 14) was completed after 4h and the reaction product **4a** as shown in Supplementary Figure 15. The product **2** was generated after in situ desulfurization and produced **4** (yield = 35% over two steps) was obtained via HPLC purification (Supplementary Figure 16).

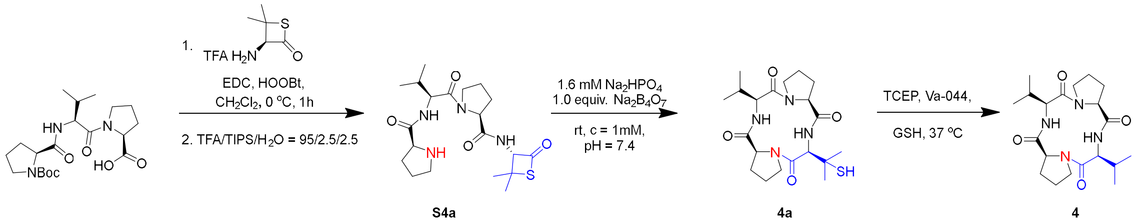

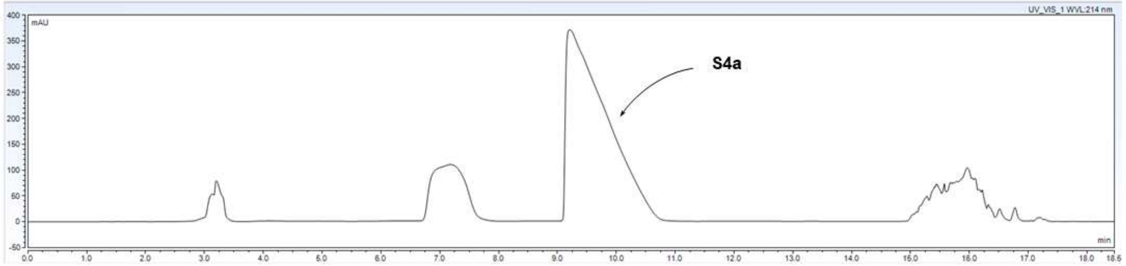

Supplementary Figure 14, the crude prep-HPLC trace of **S4a** (t_R = 9.20 min, 20 to 40% solvent B over 20 min)

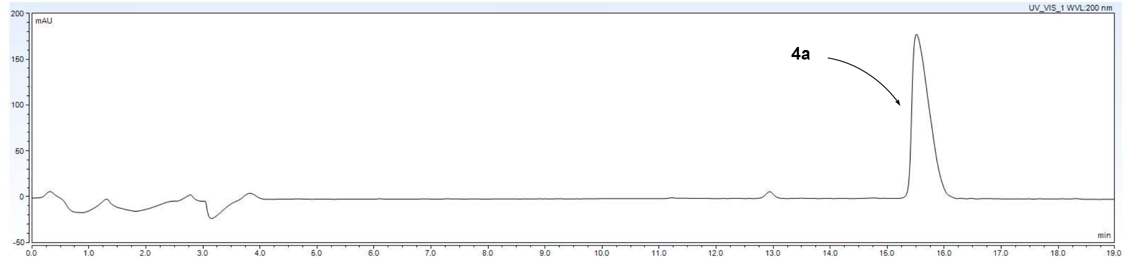

Supplementary Figure 15, the crude prep-HPLC trace of **4a** (t_R = 15.51 min, 20 to 60% solvent B over 20 min)

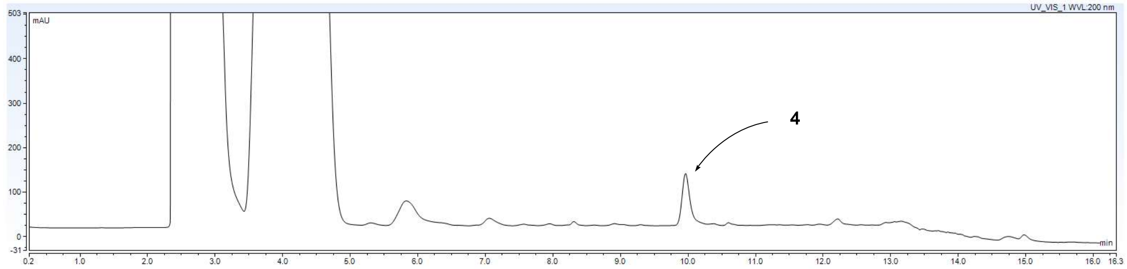

Supplementary Figure 16, the crude prep-HPLC trace of **4** (t_R = 9.96 min, 20 to 60% solvent B over 20 min)

2.1.1.5 The crude reaction HPLC trace of cyclization from Pen-β-thiolactone S9b to cPFPV 15e

At room temperature, cyclization of **S9b** (crude HPLC trace Supplementary Figure 17) was completed after 4h and the reaction product **9b** as shown in Supplementary Figure 18. The product **19b** was generated after in situ desulfurization and produced **19e** (yield = 55% over two steps) was obtained via HPLC purification (Supplementary Figure 19).

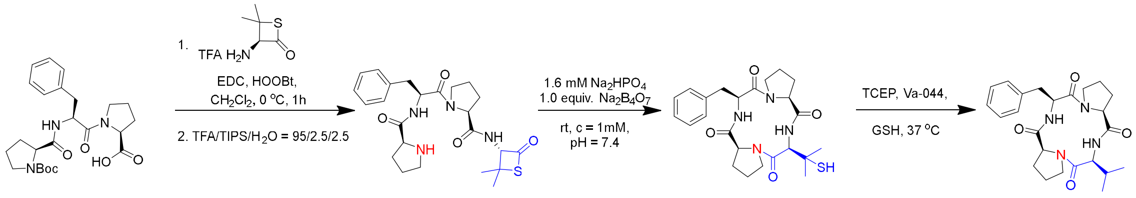

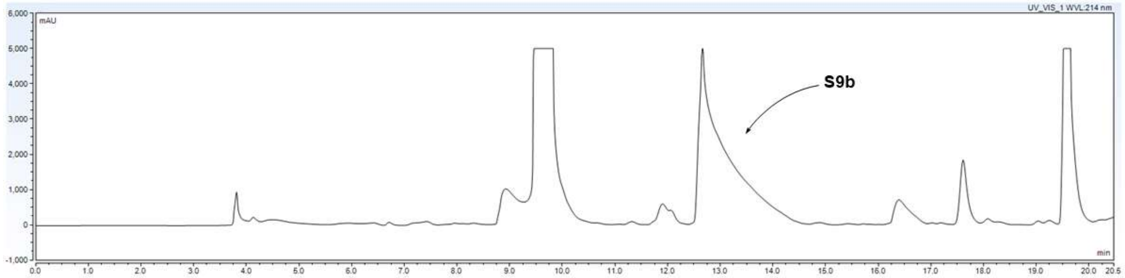

Supplementary Figure 17, the crude prep-HPLC trace of **S9b** (t_R = 12.66 min, 20 to 60% solvent B over 20 min)

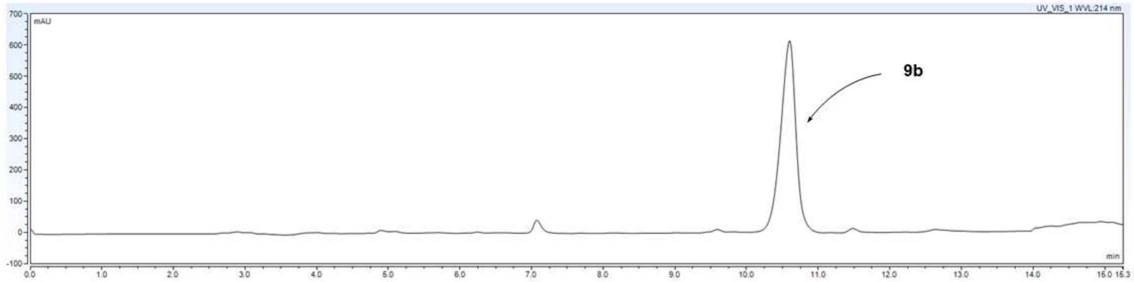

Supplementary Figure 18, the crude prep-HPLC trace of **9b** (t_R = 10.60 min, 20 to 60% solvent B over 20 min)

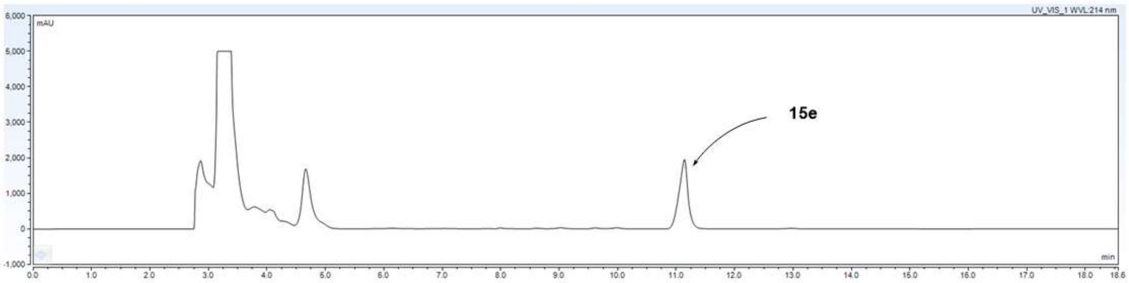

Supplementary Figure 19, the crude prep-HPLC trace of **15e** (t_R = 11.14 min, 20 to 80% solvent B over 20 min)

2.1.2 Cyclization of linear tetrapeptides attached with L-Val-phenyl thioester 16b and D-Val-phenyl thioester *d*-16b (crude reaction HPLC traces).

Preparation and cyclization of tetrapeptides 16b and *d*-16b

The side chain fully protected tripeptide Boc-Pro-Phe-Pro-OH was generated from SPPS on a 0.04 mmol scale. L-Val thiophenyl ester and D-Val thiophenyl ester salts were prepared according to literature procedure.[5] Boc-Pro-Phe-Pro-OH was coupled with L-Val thiophenyl ester or D-Val thiophenyl ester salt to afford the desired tetrapeptide **11b** and ***d*-11b** respectively using general procedure C (as shown in Supplementary Figure 20, **a** and **b**). The peptide cyclizations were performed under the standard procedure D (rt, 24h, 1mM concentration), and the reaction was monitored by LC-MS (Supplementary figure 20, **c** and **d**).

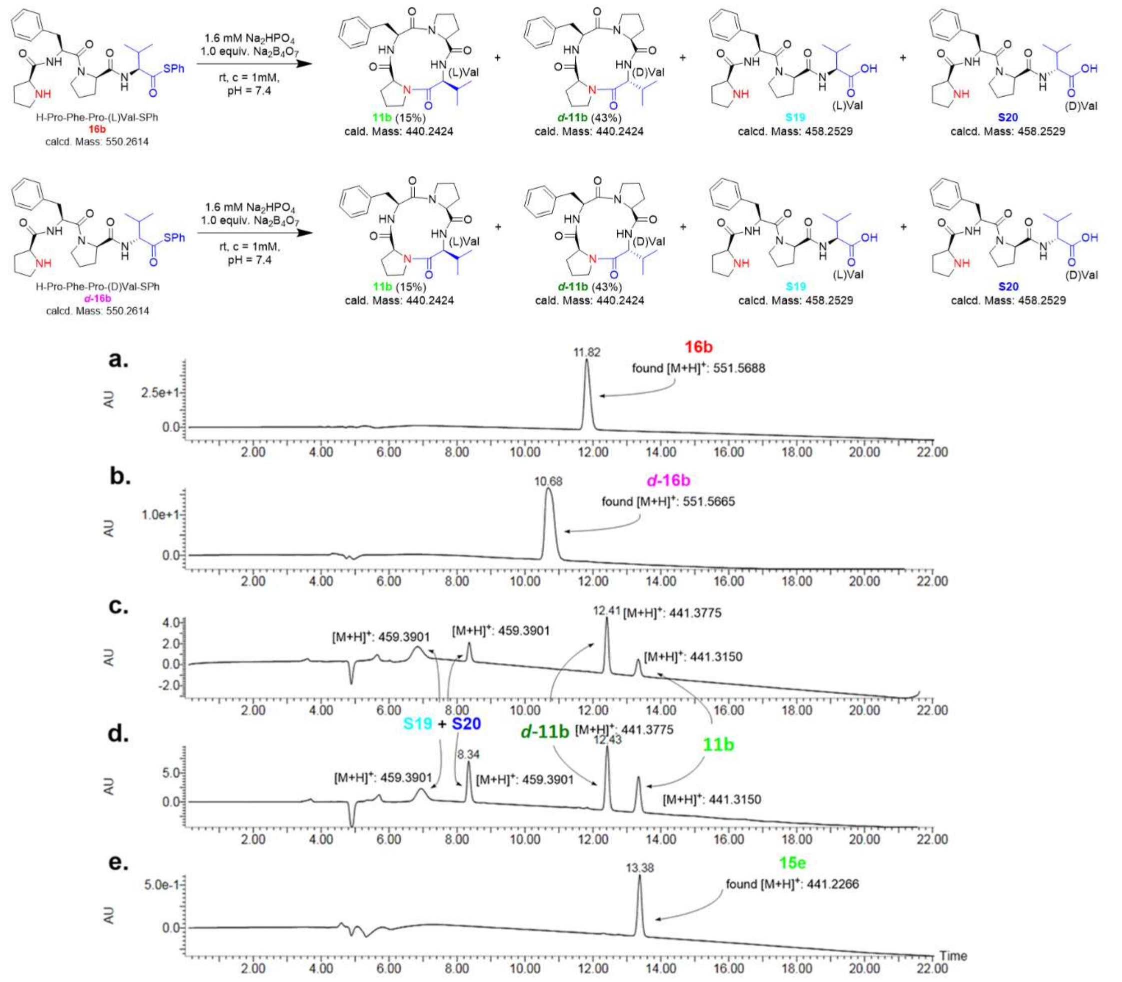

Supplementary Figure 20. HLPC traces of phenyl thioester peptide derived reactions. **a**. The HPLC trace of starting material L-Val-phenyl thioester **16b** (t_R = 11.82 min, 30 to 90%, solvent B over 20 min); **b**. The HPLC trace of starting material D-Val-phenyl thioester ***d*-16b** (t_R = 10.68 min, 30 to 90%, solvent B over 20 min), **c**. Cyclization of L-phenyl thioester **16b**, the crude products HPLC trace (20 to 80% solvent B over 20 min); **d**. Cyclization of D-phenyl thioester ***d*-16b**, the crude products HPLC trace (20 to 80% solvent B over 20 min); **e**. The HPLC trace of L-cyclo-tetrapeptide **15e** (t_R = 13.38 min, 20 to 80 solvent B over 20 min).

2.1.3 The cyclization of L-Val-phenyl thioester S21 (crude reaction HPLC traces).

Preparation and Cyclization of tetrapeptide **S21**

Tripeptide Boc-Gly-Phe-Leu-OH was generated via SPPS on a 0.04 mmol scale. Following the general procedure C, Boc-Gly-Phe-Leu-OH was coupled with L-Val phenyl thioester salt to afford the desired tetrapeptide **S21** (HPLC trace was shown in Supplementary Figure 21a).

Following the general procedure D, the cyclization of **S21** was attempted at room temperature for 48h (1mM concentration), and the reaction crude HPLC trace was shown in Supplementary Figure 21b, 8h).

Reference tetrapeptides **S22** and **S23** were prepared via SPPS using L-Val TGT resin or D-Val TGT resin respectively on a 0.04 mmol scale (HPLC traces was shown in Supplementary Figure 21c, 11d).

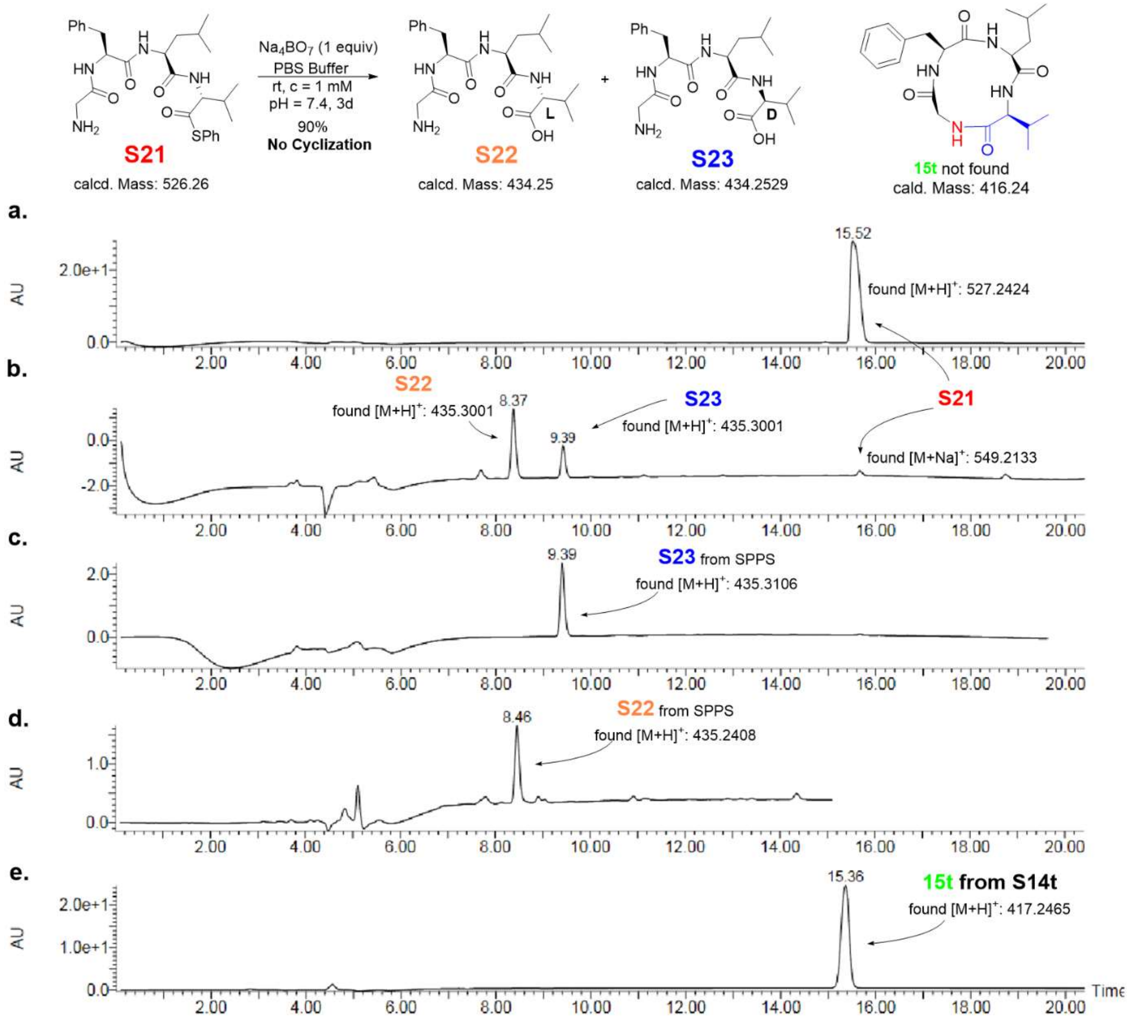

Supplementary Figure 21. HPLC traces for the cyclization of **S21**. **a**. The HPLC trace of starting material L-Val-phenyl thioester **S21**; **b**. The crude HPLC trace for cyclization of L-phenyl thioester **S21** at room temperature for 8h (30 to 90% solvent B over 20 min); **c.** The reference HPLC trace of Gly-Phe-Leu-D-Val **S23** prepared by SPPS using D-Val TGT resin (t_R = 9.39 min, 30 to 80%, solvent B over 20 min); **d.** The reference HPLC trace of Gly-Phe-Leu-L-Val **S22** prepared by SPPS using L-Val TGT resin (t_R = 8.46 min, 30 to 80%, solvent B over 20 min); **e.** The reference HPLC trace of cyclo-tetrapeptide **15t** (t_R = 15.36 min, 30 to 80 solvent B over 20 min).

2.2 The NMR Structure Determination of 1 and NMR Conformers of Synthesized Cyclopeptides.

2.2.1 NMR Spectroscopy.

NMR samples were prepared in DMSO-*d*6 to a concentration of approximately 0.5 mM. All NMR spectra were acquired on Bruker Avance III 600 MHz spectrometer equipped with TCI cryoprobe. The NMR spectra were acquired at 298 K, and 2,2-dimethyl-2-silapentane-5-sulfonate, DSS, was used as an internal reference. 2D ^{13}C-HSQC were collected using 1024 t_2 points and 512 t_1 of 32 transients, 2D ^{1}H,

^{1}H-TOCSY (spin lock time 80 ms) were collected using 4096 t_2 points and 256 t_1 of 4 transients, and 2D ^{1}H, ^{1}H-NOESY (mixing time 200 ms) spectra were collected using 4096 t_2 points and 512 t_1 of 8 transients. Spectra were processed using Topspin 1.3 (Bruker). Spectra were analyzed by CARA software and assignment were obtained using standard methods.

2.2.2 Structure determination of 1.

Structural calculations were carried out with Cyana 3.98.5 using 16 distance restraints derived from ^{1}H-^{1}H-NOESY, 12 pairs of backbone torsion angle restraints derived from TALOS. To account for the cyclic nature of **1** cyclo-(Pro-Tyr-Pro-Val), amide nitrogen of Pro1 and carbonyl carbon of Val4 were linked and upper and lower distance restraints between amide proton and nitrogen of Pro1 and carbonyl carbon and oxygen of Val4 were added. NOEs were converted to upper limit distances using the CALIBA module in CYANA. The reference volume determined by CALIBA was increased 2 times before conversion in order to loosen the distance restraints. All upper limit distances for intermolecular nOes were set to 5 Å. These experimental restraints are summarized in **Supplementary Table 1**.

Refinement: The CYANA-generated structures were subjected to minimization in explicit water by using YASARA and further analysis by PROCHECK_NMR. 100% of the **1** cyclo-(Pro-Tyr-Pro-Val) residues were in the additional allowed regions of Ramachandran plot. There were no residues in generously allowed or the disallowed regions of the Ramachandran plot. The structural statistics of the 10 best structures are reported in **Supplementary Table 1**. The solution structure of **1** cyclo-(Pro-Tyr-Pro-Val) has been deposited and assigned the PDB ID code 6DNY.

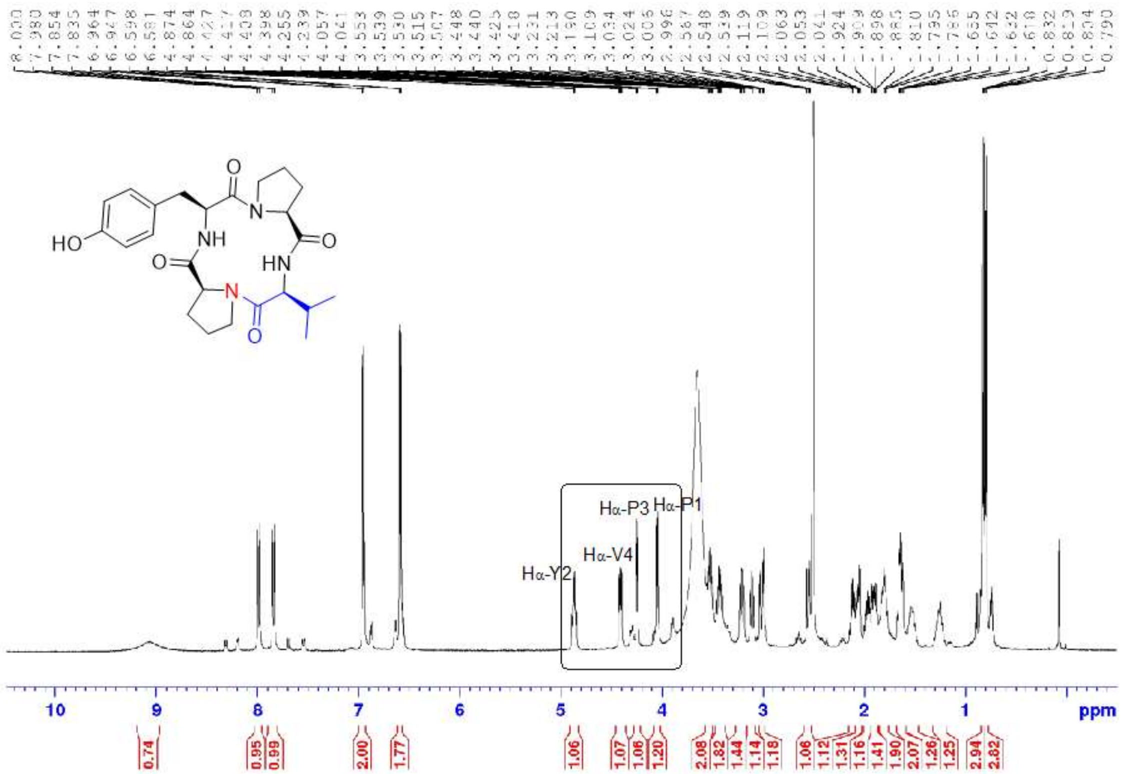

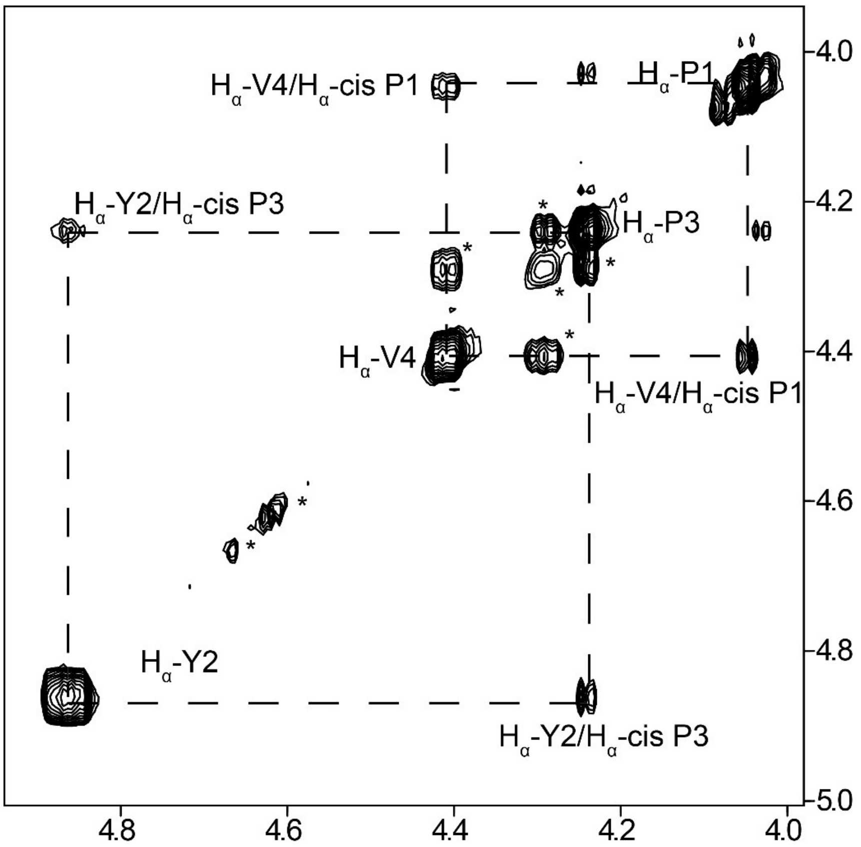

Supplementary Figure 22. P1 and P3 are cis proline isomers. Isomeric assignments of prolines are based on characteristic sequential connectivities observed **1** ^{1}H, ^{1}H-ROESY spectrum. The connectivity between Hα of Y2 and Hα of P3 is characteristic of cis configuration and the connectivity between Hα of V5 and Hα of P6 is characteristic of cis configuration.[6] Impurities are noted with an asterisks.

2.2.3 NMR Experiments to Identify Conformational Isomers.

2.2.3.1 Variable temperature experiment of linear tetrapeptide S14a.

Many of the linear tetrapeptides' NMR spectra show conformers due to the free rotation amide bonds. In order to confirm the nature of isomeric peaks are attributed to the conformational change of linear peptides in the NMR solvents instead of epimerization during the synthesis, the Variable Temperature (VT) NMR experiments of **S14a** were carried out and shown below (**Supplementary Figure 23**).

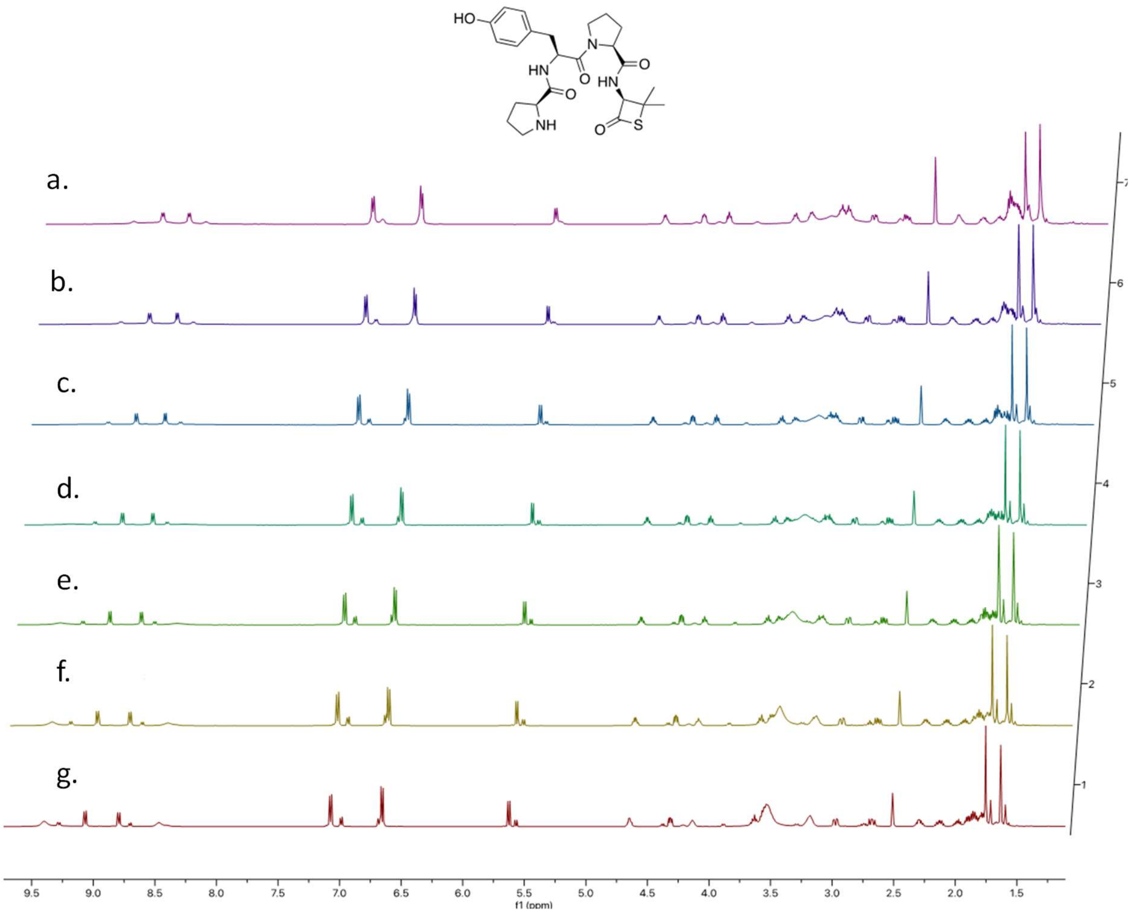

Supplementary Figure 23. The variable temperature ^{1}H-NMR spectrum of **S14a** (DMSO-*d6*, 500 MHz). a. ^{1}H-NMR spectrum of **S14a** in DMSO-d6 at 85 °C. b. ^{1}H-NMR spectrum of **S14a** in DMSO-d6 at 75 °C. c. ^{1}H-NMR spectrum of **S14a** in DMSO-d6 at 65 °C. d. ^{1}H-NMR spectrum of **S14a** in DMSO-d6 at 55 °C. e. ^{1}H-NMR spectrum of **S14a** in DMSO-d6 at 45 °C. f. ^{1}H-NMR spectrum of **S14a** in DMSO-d6 at 35 °C. g. ^{1}H-NMR spectrum of **S14a** in DMSO-d6 at 25 °C

The spectra of **S14a** in DMSO-*d*6 was compiled and show a clear trend of merging into one set of peaks. Unfortunately, the heating capacity of Bruker 500 MHz NMR is capped at 70 degree, 85 °C is the highest temperature that we could reach. Regardless, the above diagrams are sufficient to prove the isomeric peaks are derived from structure conformational changes.

2.2.3.2 NMR experiments of cyclic tetrapeptide 1 (cPYPV).

Although all the synthesized cyclopeptides' HPLC traces show clean single peak. The rigid structure of many cyclo-tetrapeptides did not provide exclusive, single conformer NMR spectra, rather, multiple conformers exists depending on the solvent of use. It is particularly true for the cyclic peptides containing two prolines. We found that DMSO-*d*6 generally affords spectra with minimal amount of conformers. In addition to VT experiments, alternating NMR solvents methods were used to determine the conformation of **1** (**Supplementary Figure 24** and **Supplementary Figure 25**). It has been reported that the change of polarity of the solvents will impact the ratio of conformers,[7] which in turns validate that cyclization did not induce epimerization.

Solvent Experiment of **1**

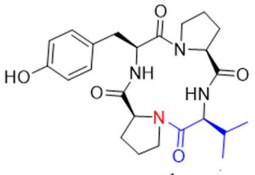

1

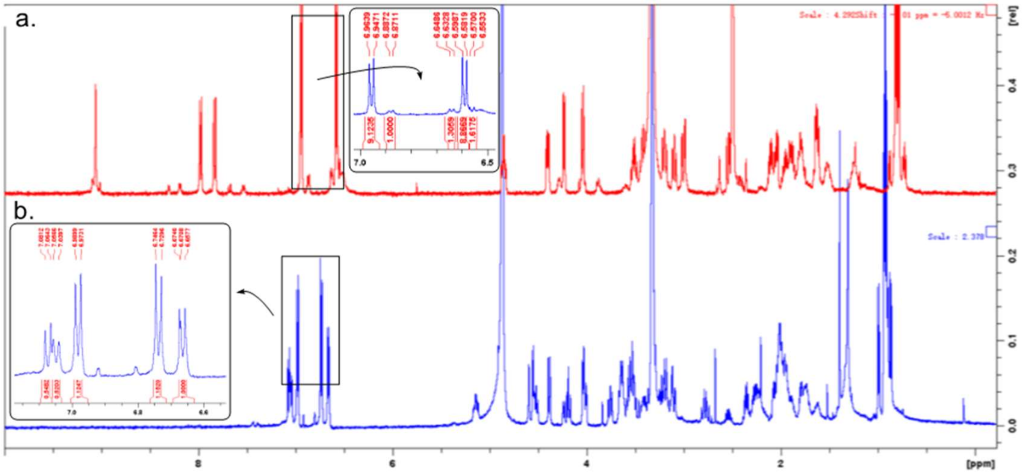

Supplementary Figure 24. ^{1}H NMR spectrum of compound **1** in different solvents. a. **1** in DMSO-*d*6, 500 MHz; the ratio of two conformers is 10: 1. b. **1** in Methanol-*d*4, 600 MHz; the ratio of two conformers is 1.1: 1

The VT Experiment of **1** (in DMSO-*d6*)

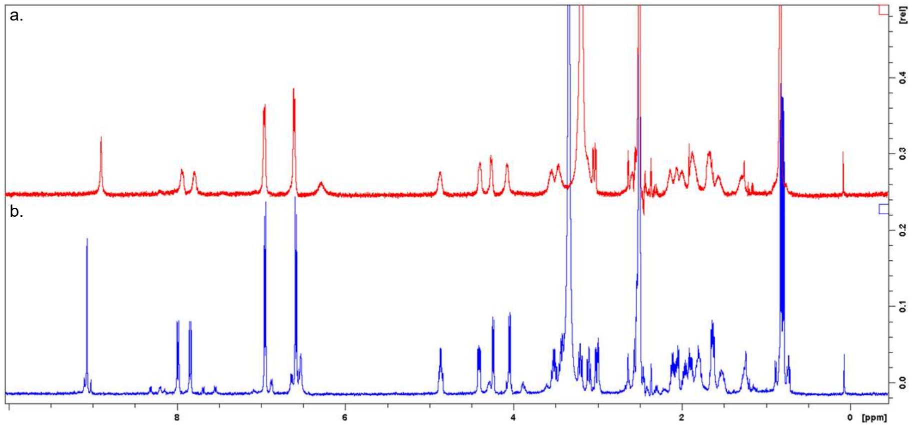

Supplementary Figure 25. ^{1}H NMR spectrum of compound **1** in different temperatures (DMSO-*d*6, 500 MHz) a.

1 in DMSO-*d6* at 60 °C, there was only one conformer was observed b. **1** in DMSO-*d6* at rt, there were two conformers with the ratio of 10: 1.

2.2.3.3 NMR experiments of symmetric cyclic tetrapeptide 2 (cPLPL) and 3 (cPYPY).

The NMR experiment of cyclic tetrapeptide **2** (cPLPL).

Cyclic tetrapeptide **2** appears as two sets of conformers in DMSO-*d*6, changing the solvent to $CDCl_3$ afforded cleaner NMR spectrum with only one set of symmetric peaks.

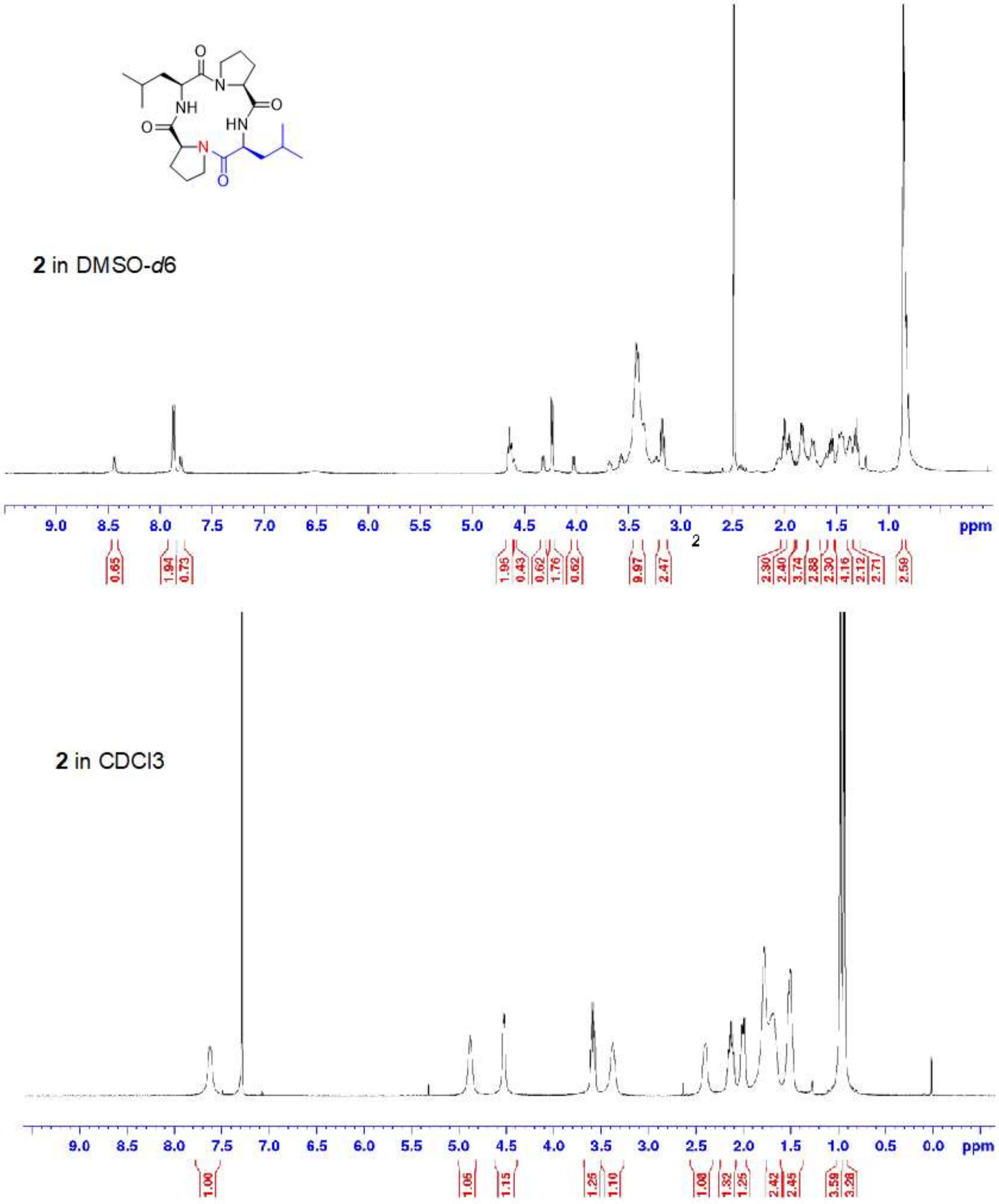

Supplementary Figure 26. The comparing of ^{1}H-NMR spectrum of **2** in DMSO-*d*6 and $CDCl_3$

The VT NMR experiments of cyclic tetrapeptide **3** (cPFPF).

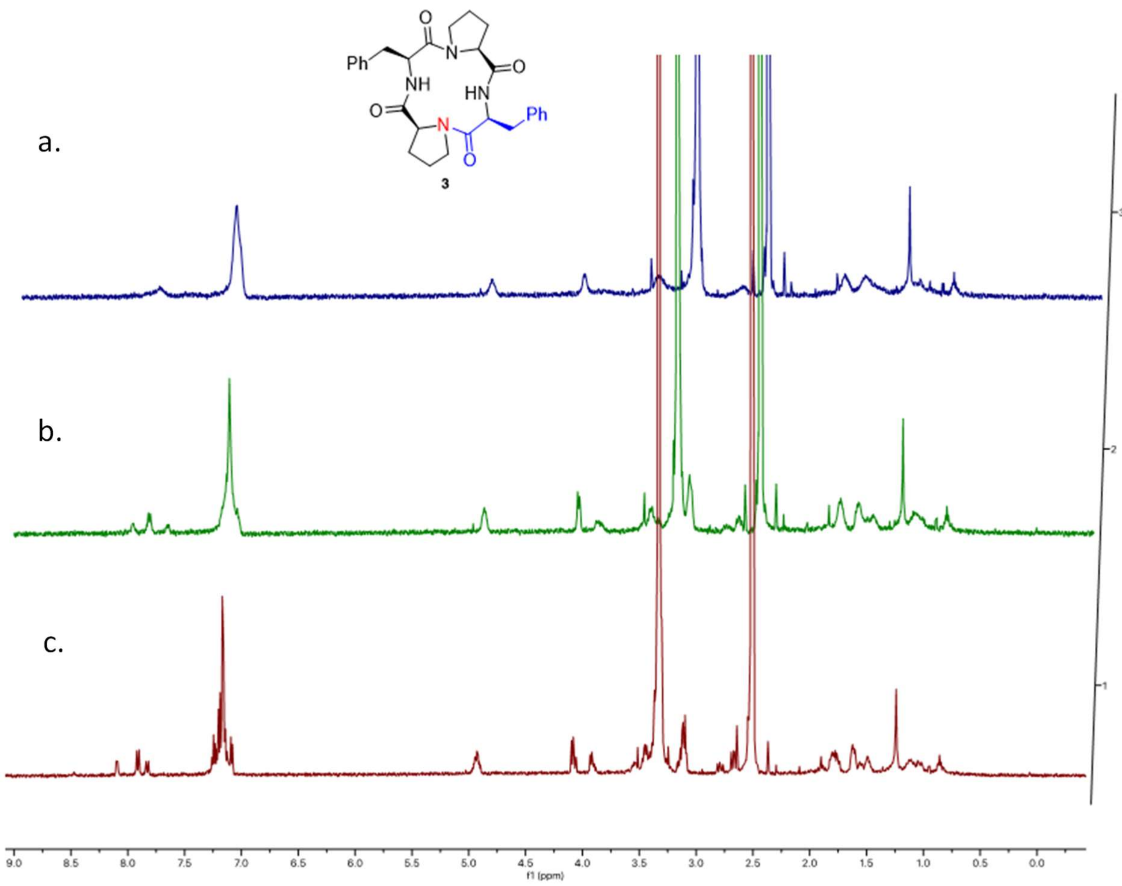

Supplementary Figure 27. The comparing of variety temperature ^{1}H-NMR spectrum of **3** (DMSO-*d6*, 500 MHz). a. ^{1}H-NMR spectrum of **3** in DMSO-d6 at 65 °C. b. ^{1}H-NMR spectrum of **3** in DMSO-d6 at 45 °C. c. ^{1}H-NMR spectrum of **3** in DMSO-d6 at 25 °C

The VT experiments indicate the symmetric structure of **3,** and please see section VII for symmetric cyclopeptides **4.**

2.2.3.4 NMR comparation for the corresponding cyclic tetrapeptide.

2.2.3.4.1 Cyclic (Phe-Pro-Phe-Pro) and Cyclic (*d*Phe-Pro-Phe-Pro)

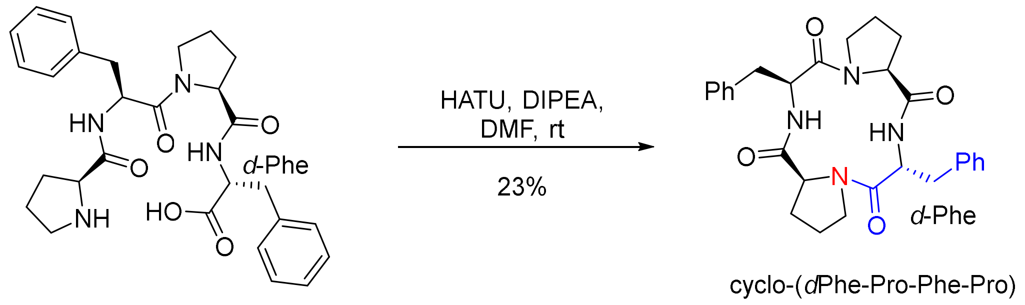

Cyclo-(*d*Phe-Pro-Phe-Pro) was prepared from HATU cyclization with 23% yield.

Cyclo-(*d*Phe-Pro-Phe-Pro), ^{1}H NMR (500 MHz, DMSO-d6) δ 8.49 (d, J = 5.0 Hz, 1H), 8.04 (d, J = 9.9 Hz, 1H), 7.28 – 7.24 (m, 2H), 7.21 (dd, J = 6.7, 2.1 Hz, 3H), 7.18 (d, J = 4.3 Hz, 4H), 7.15 – 7.11 (m, 1H), 5.10 (td, J = 10.4, 4.9 Hz, 1H), 4.28 (ddd, J = 8.4, 6.3, 4.7 Hz, 1H), 4.18 (dd, J = 13.4, 8.5 Hz, 2H), 3.22 – 3.11 (m, 3H), 3.02 (dd, J = 14.2, 8.5 Hz, 1H), 2.89 – 2.76 (m, 2H), 2.09 (ddd, J = 12.1, 8.1, 4.4 Hz, 1H), 1.77 (dt, J = 9.2, 4.8 Hz, 3H), 1.72 (ddd, J = 14.0, 7.4, 2.8 Hz, 2H), 1.43 – 1.35 (m, 1H), 1.33 – 1.19 (m, 2H), 1.02 (dd, J = 12.4, 6.5 Hz, 1H).

^{13}C NMR (126 MHz, DMSO-d6) δ 173.15, 168.83, 138.93, 138.11, 130.16, 129.49, 128.64, 127.97, 126.92, 126.19, 61.05, 59.05, 58.24, 51.52, 48.46, 47.98, 37.31, 35.31, 32.27, 31.63, 21.22, 21.07.

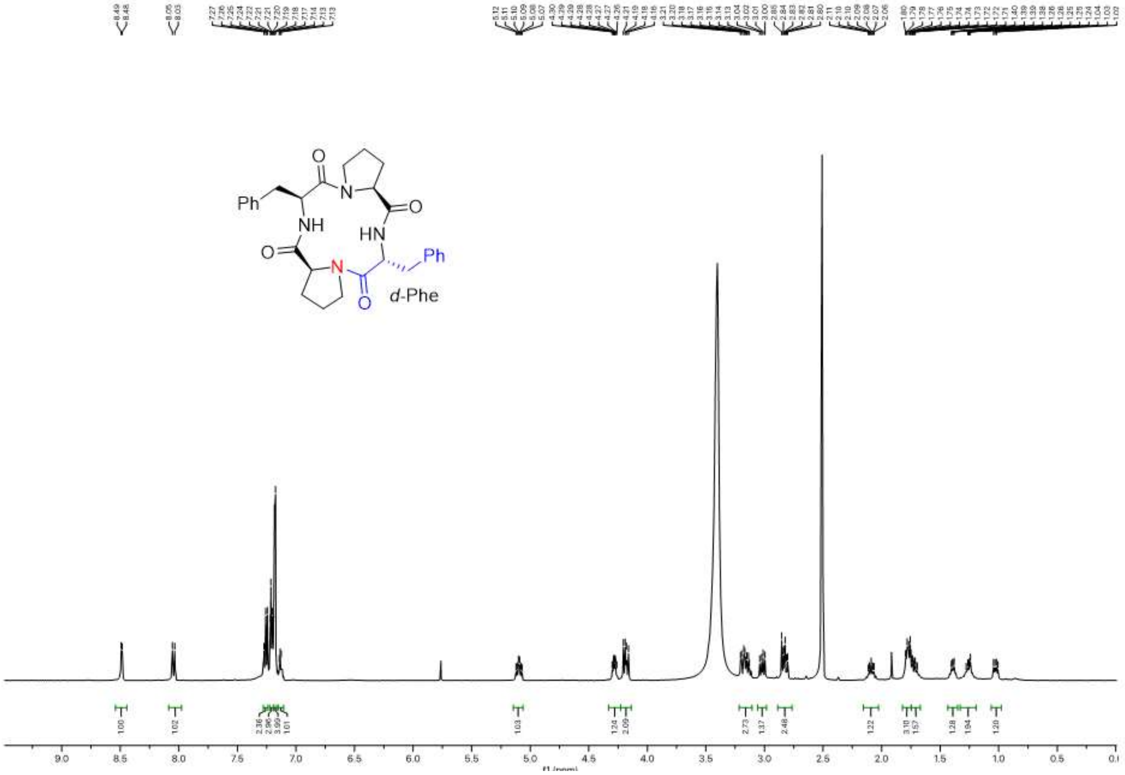

The ^{1}H-NMR spectrum of Cyclo-(*d*Phe-Pro-Phe-Pro) (DMSO-*d6*, 500 MHz)

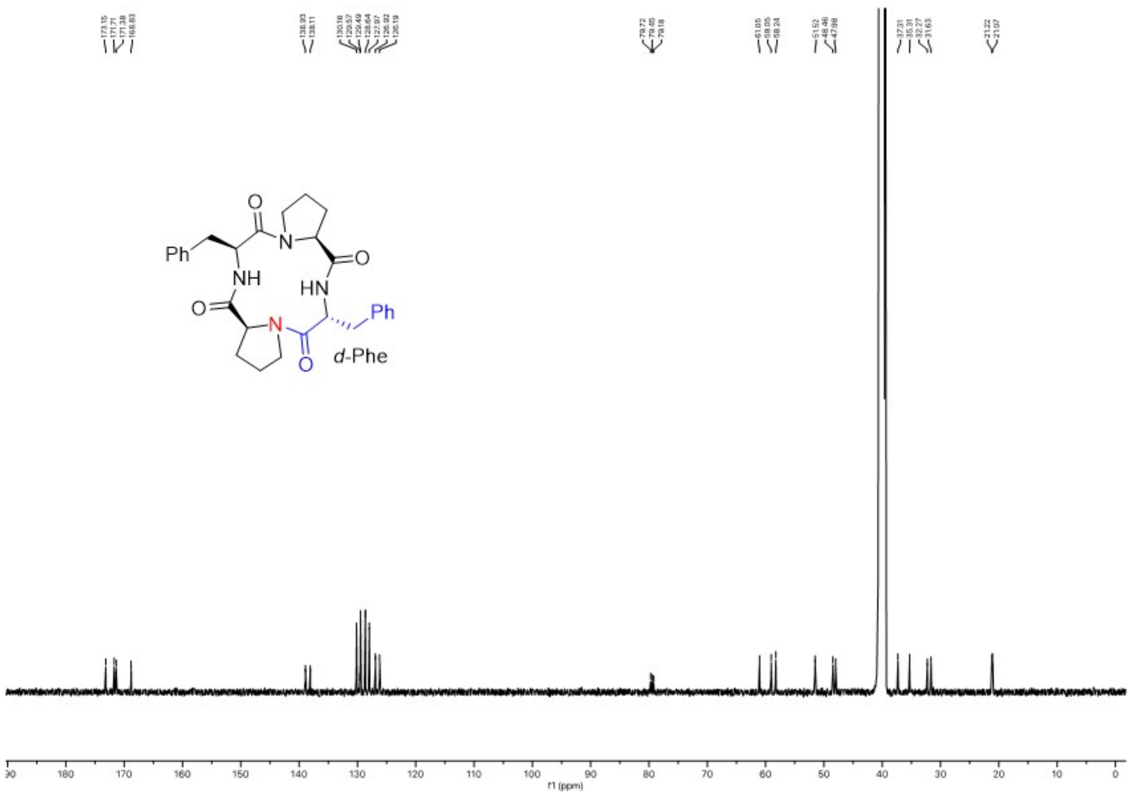

The ^{13}C-NMR spectrum of Cyclo-(*d*Phe-Pro-Phe-Pro) (DMSO-*d6*, 500 MHz)

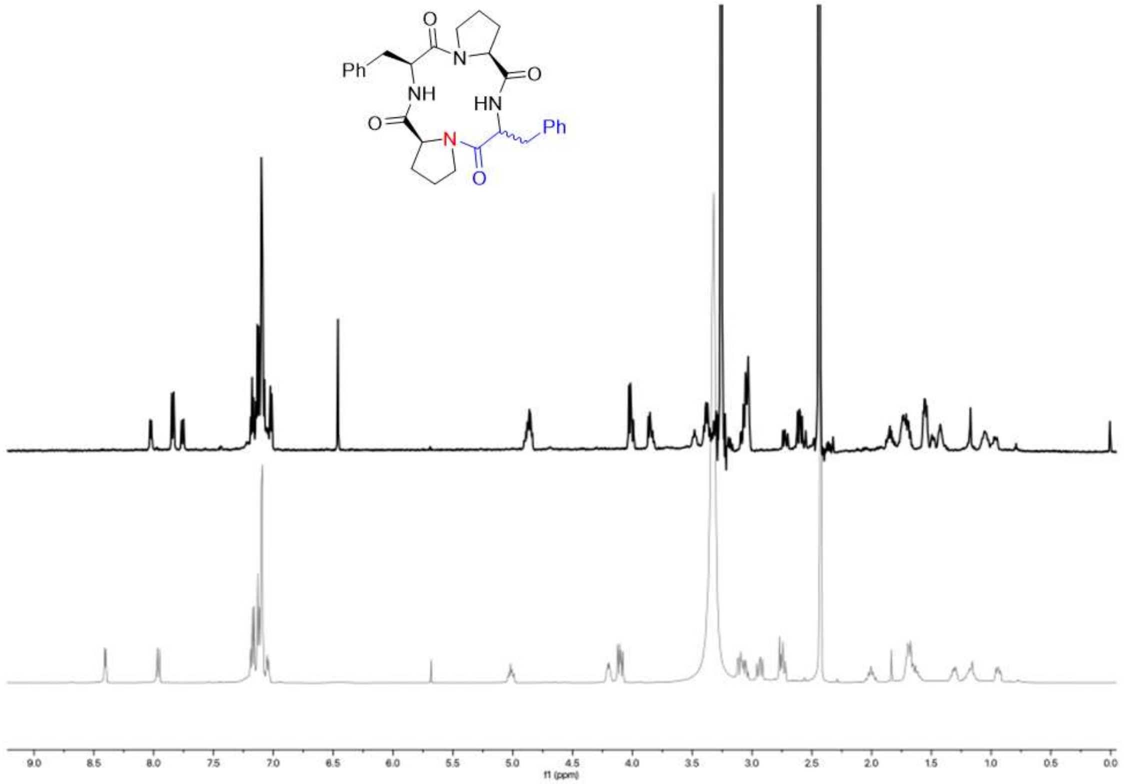

The comparation of ^{1}H-NMR spectrum for Cyclo-(*d*Phe-Pro-Phe-Pro) and Cyclo-(*d*Phe-Pro-Phe-Pro) (DMSO-*d6*, 500 MHz)

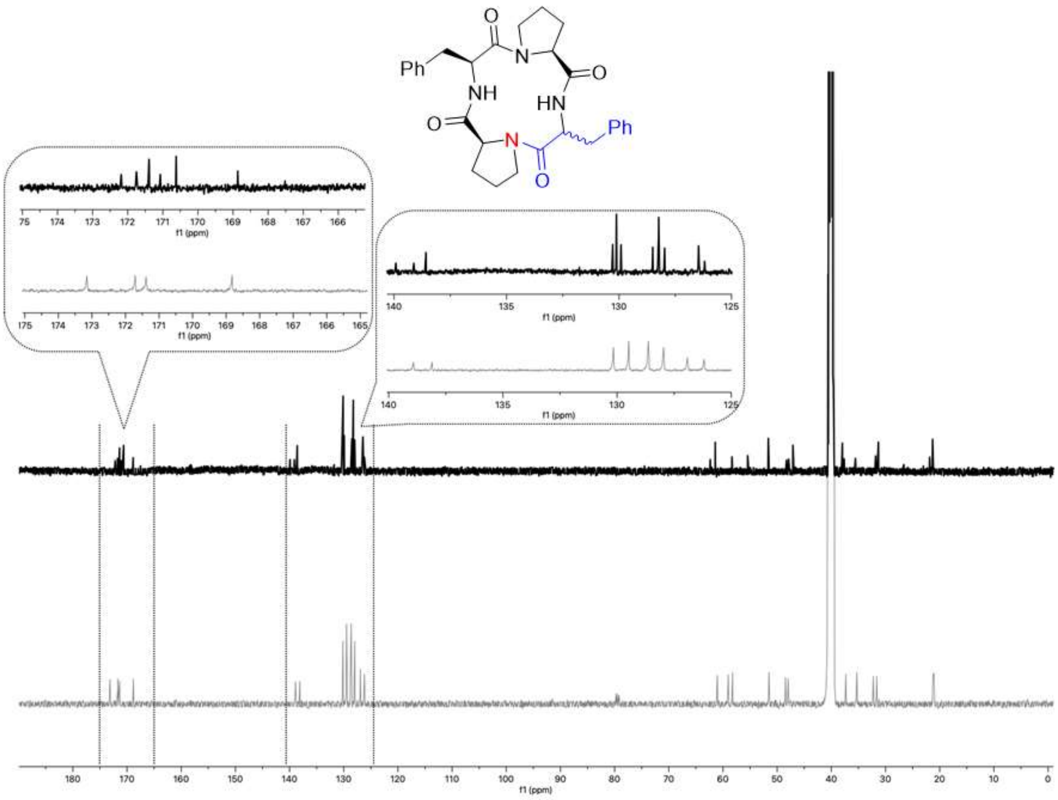

The comparation of ^{13}C-NMR spectrum for Cyclo-(*d*Phe-Pro-Phe-Pro) and Cyclo-(*d*Phe-Pro-Phe-Pro) (DMSO-*d6*, 500 MHz)

2.2.3.4.2 Cyclo-(Pro-Leu-Pro-Leu) and cyclo-(Pro-Leu-Pro-*d*Leu)

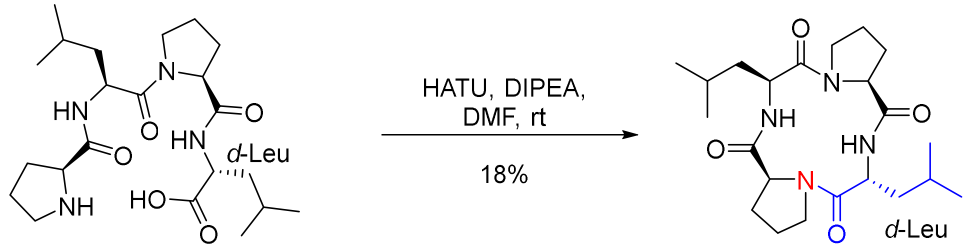

cyclo-(Pro-Leu-Pro-*d*Leu)

Cyclo-(Pro-Leu-Pro-*d*Leu) was prepared from HATU cyclization with 18% yield.

Cyclo-(Pro-Leu-Pro-*d*Leu), ^{1}H NMR (500 MHz, Chloroform-*d*) δ 6.73 (d, *J* = 10.3 Hz, 1H), 6.31 (d, *J* = 5.0 Hz, 1H), 4.85 (td, *J* = 9.7, 4.5 Hz, 2H), 4.47 (d, *J* = 8.8 Hz, 1H), 4.43 (dt, *J* = 9.4, 5.6 Hz, 1H), 4.27 (d, *J* = 8.3 Hz, 1H), 3.81 – 3.73 (m, 1H), 3.63 (dt, *J* = 7.6, 3.0 Hz, 2H), 3.58 (dt, *J* = 12.0, 6.4 Hz, 1H), 2.33 – 2.14 (m, 4H), 1.97 (t, *J* = 9.5 Hz, 3H), 1.73 (ddd, *J* = 17.7, 12.4, 6.3 Hz, 2H), 1.69 – 1.59 (m, 2H), 1.44 (ddd, *J* = 15.1, 9.1, 3.9 Hz, 2H), 1.02 (d, *J* = 6.4 Hz, 3H), 0.96 (dd, *J* = 6.4, 2.5 Hz, 9H).

^{13}C NMR (126 MHz, Chloroform-*d*) δ 173.79, 173.13, 172.48, 168.06, 61.48, 58.44, 56.41, 49.66, 48.42, 47.86, 41.35, 38.07, 32.97, 31.55, 25.55, 24.45, 23.48, 22.78, 21.89, 21.57, 21.60, 21.18.

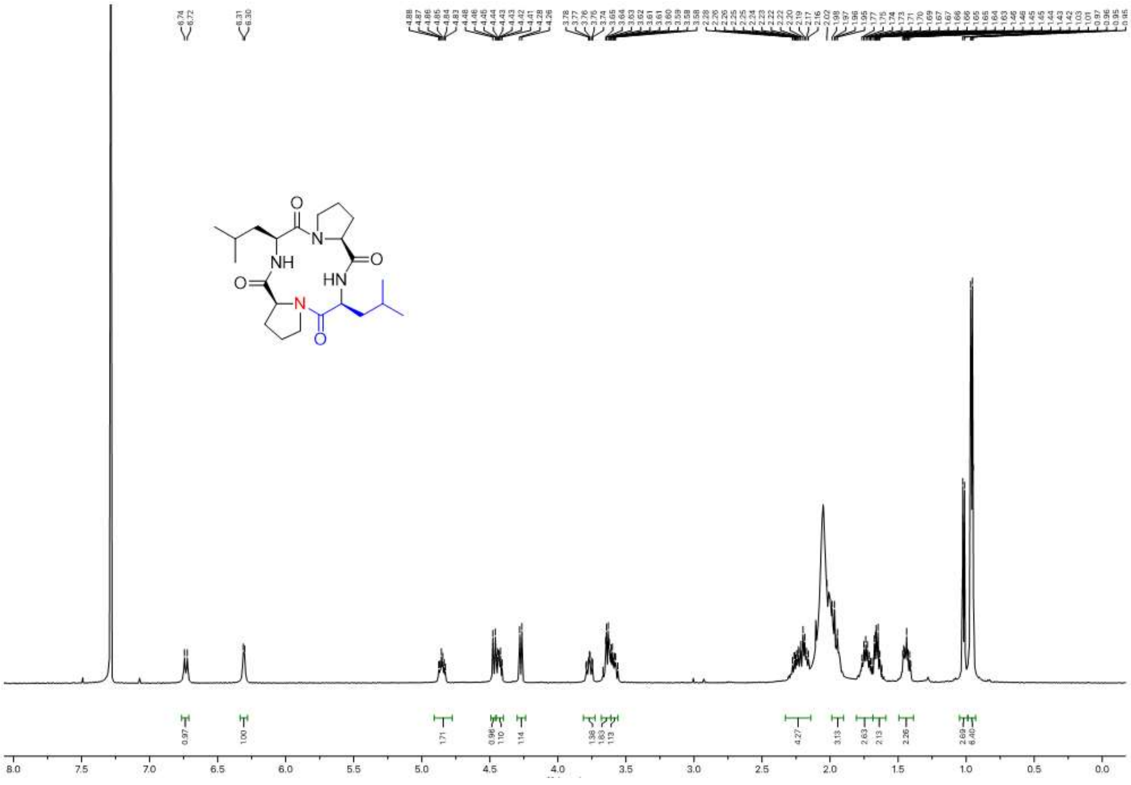

The ^{1}H-NMR spectrum of Cyclo-(Pro-Leu-Pro-*d*Leu) (Chloroform-*d6*, 500 MHz)

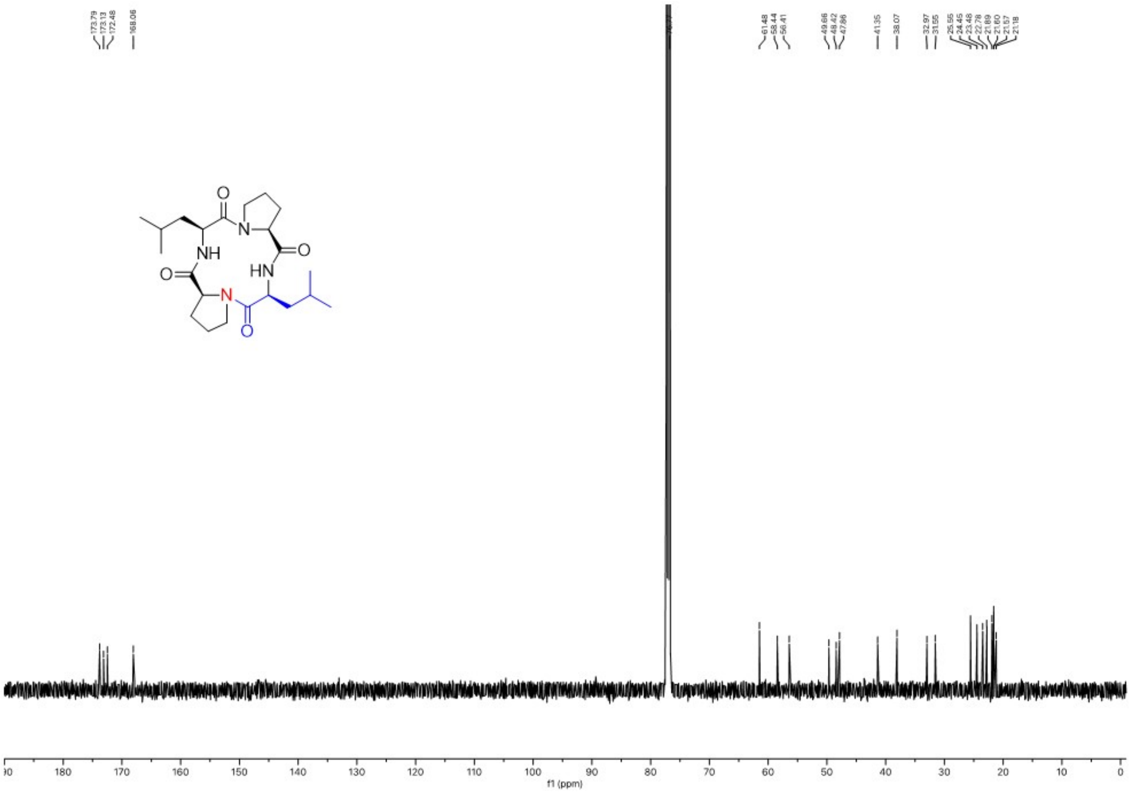

The ^{13}C-NMR spectrum of Cyclo-(Pro-Leu-Pro-*d*Leu) (Chloroform-*d6*, 500 MHz)

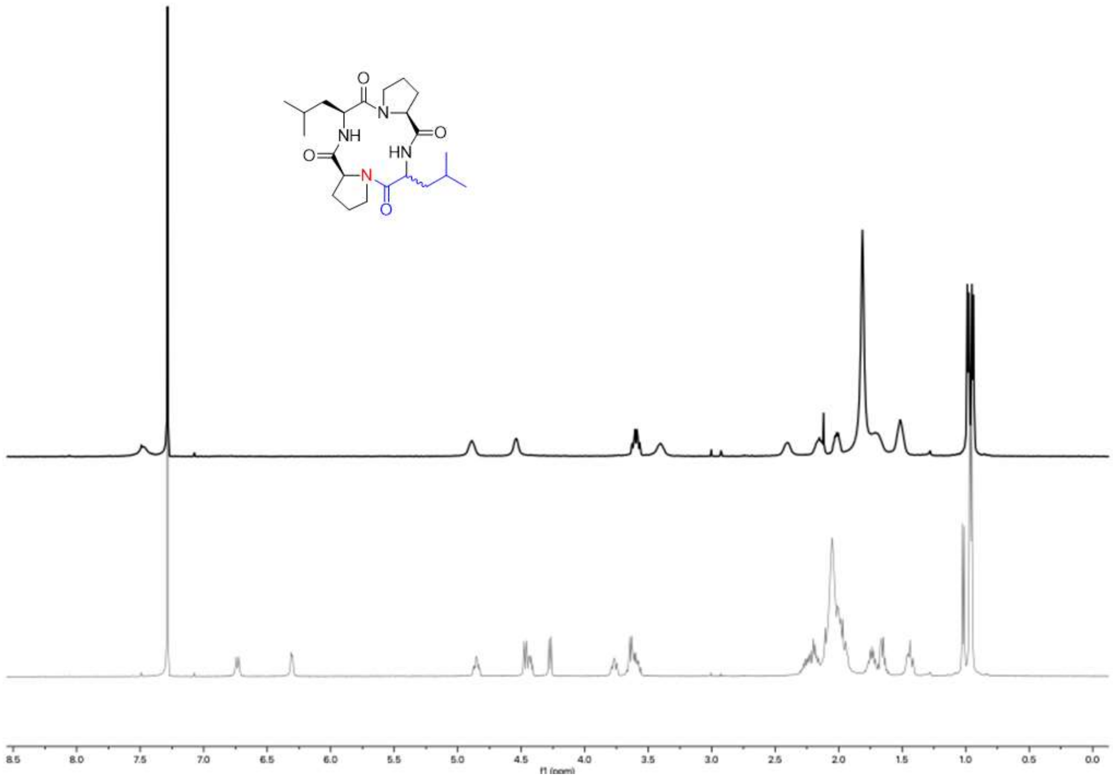

The comparation of ¹H-NMR spectrum for Cyclo-(Pro-Leu-Pro-*d*Leu) and Cyclo-(Pro-Leu-Pro-Leu) (Chloroform-*d6*, 500 MHz)

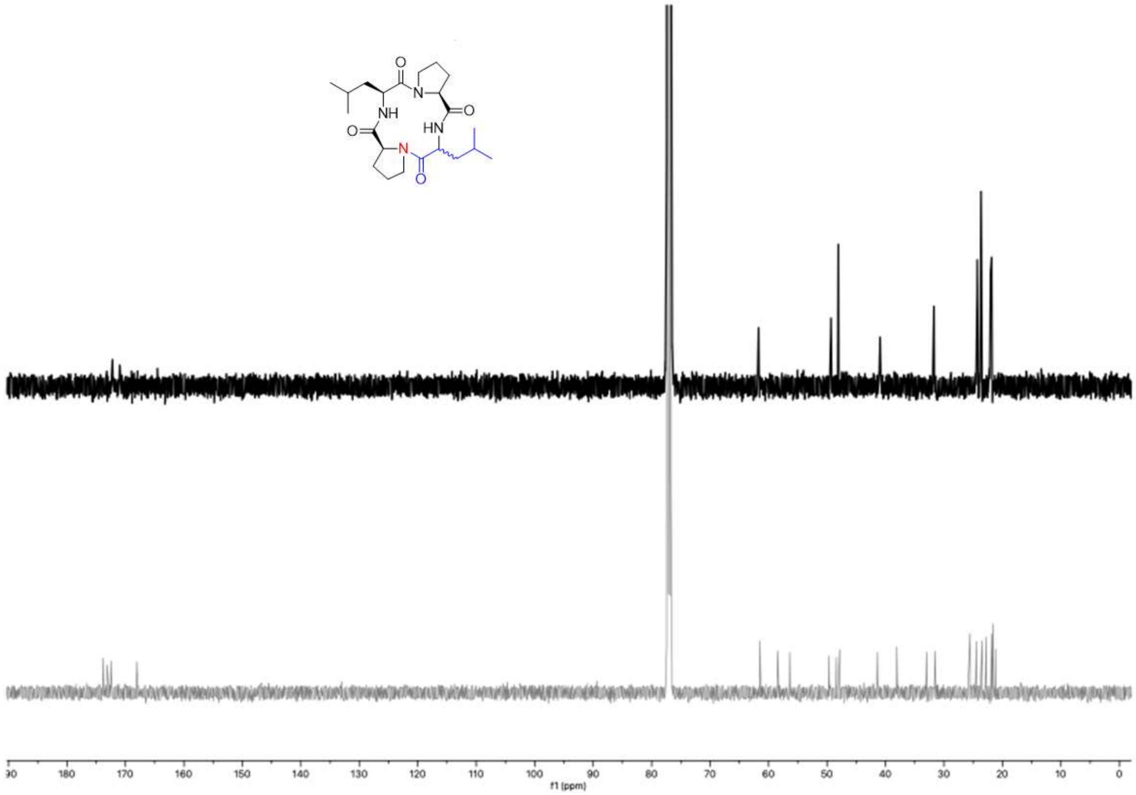

The comparation of ¹³C-NMR spectrum for Cyclo-(Pro-Leu-Pro-*d*Leu) and Cyclo-(Pro-Leu-Pro-Leu) (Chloroform-*d6*, 500 MHz)

2.2.3.4.3 Cyclo-(Pro-Tyr-Pro-Val) and cyclo-(Pro-Tyr-Pro-*d*Val)

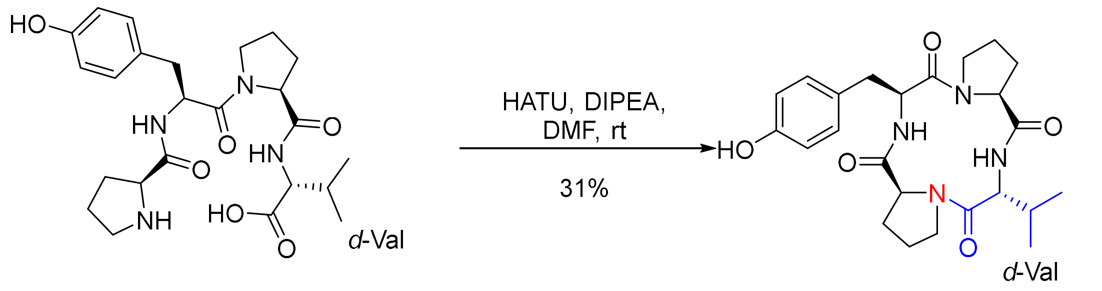

cyclo-(Pro-Tyr-Pro- *d*Val)

Cyclo-(Pro-Tyr-Pro-*d*Val) was prepared from HATU cyclization with 31% yield.

Cyclo-(Pro-Tyr-Pro-*d*Val) , ^{1}H NMR (500 MHz, DMSO-*d*$_6$) δ 9.03 (s, 1H), 8.24 (d, *J* = 4.9 Hz, 1H), 7.93 (d, *J* = 9.9 Hz, 1H), 6.98 – 6.92 (m, 2H), 6.59 – 6.52 (m, 2H), 4.96 (dt, *J* = 10.3, 5.1 Hz, 1H), 4.18 (d, *J* = 8.4 Hz, 2H), 3.68 (dd, *J* = 10.1, 4.7 Hz, 1H), 3.52 – 3.40 (m, 3H), 3.25 – 3.15 (m, 1H), 3.07 (dd, *J* = 14.2, 4.9 Hz, 1H), 2.09 (s, 1H), 1.91 (d, *J* = 9.4 Hz, 1H), 1.85 – 1.79 (m, 2H), 1.78 (s, 3H), 1.45 (d, *J* = 7.0 Hz, 1H), 1.24 (s, 1H), 1.25 – 1.16 (m, 1H), 0.98 (d, *J* = 6.3 Hz, 3H), 0.81 (d, *J* = 6.7 Hz, 3H).

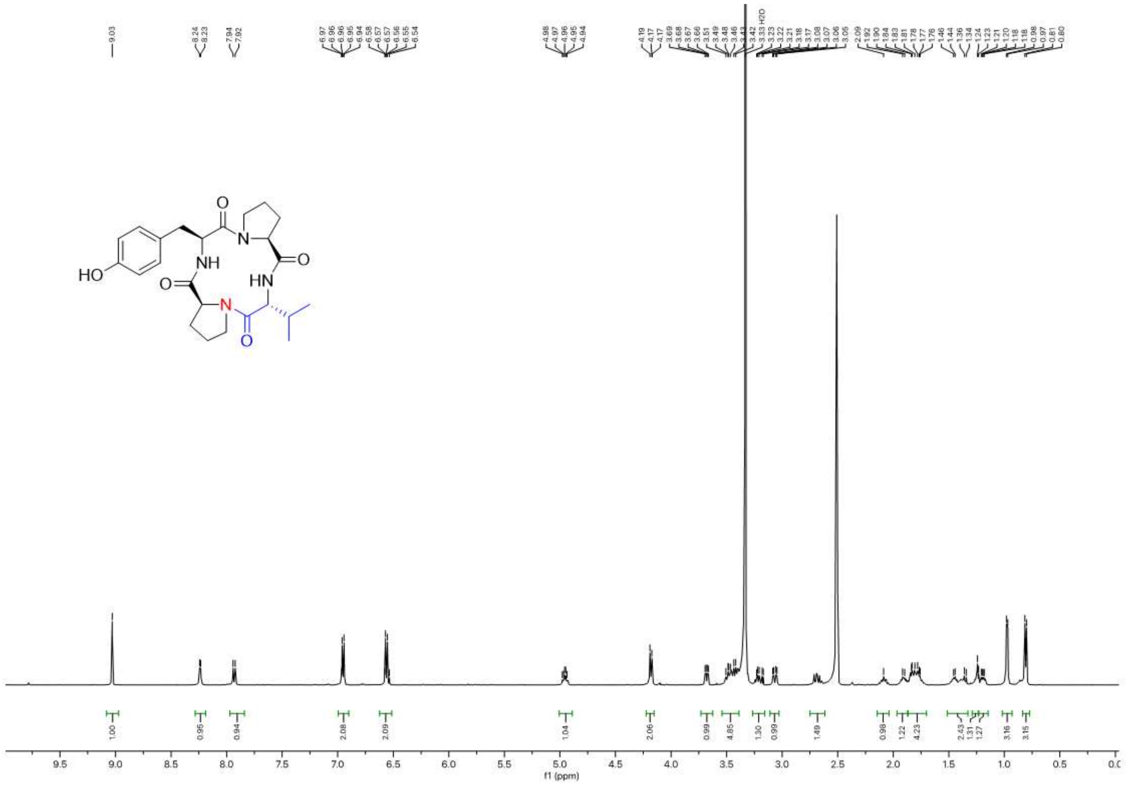

The ^{1}H-NMR spectrum of Cyclo-(Pro-Tyr-Pro-*d*Val) (DMSO-*d6*, 500 MHz)

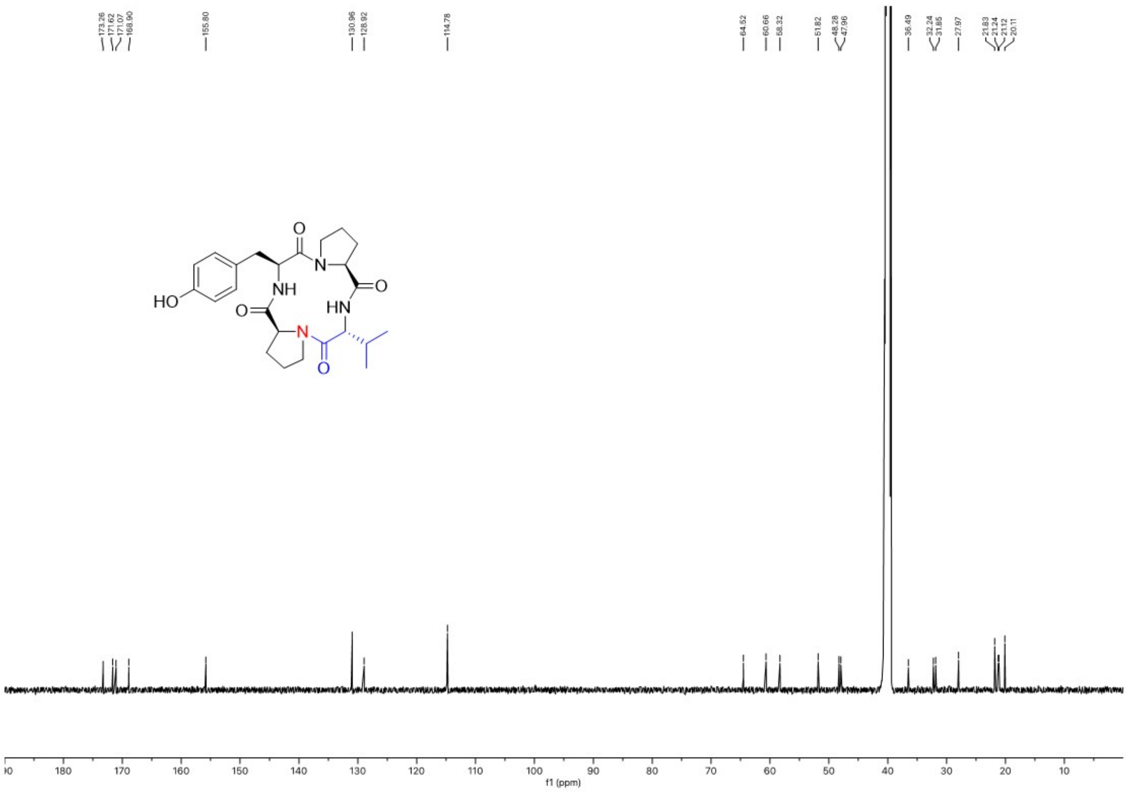

The ^{13}C-NMR spectrum of Cyclo-(Pro-Tyr-Pro-*d*Val) (DMSO-*d6*, 500 MHz)

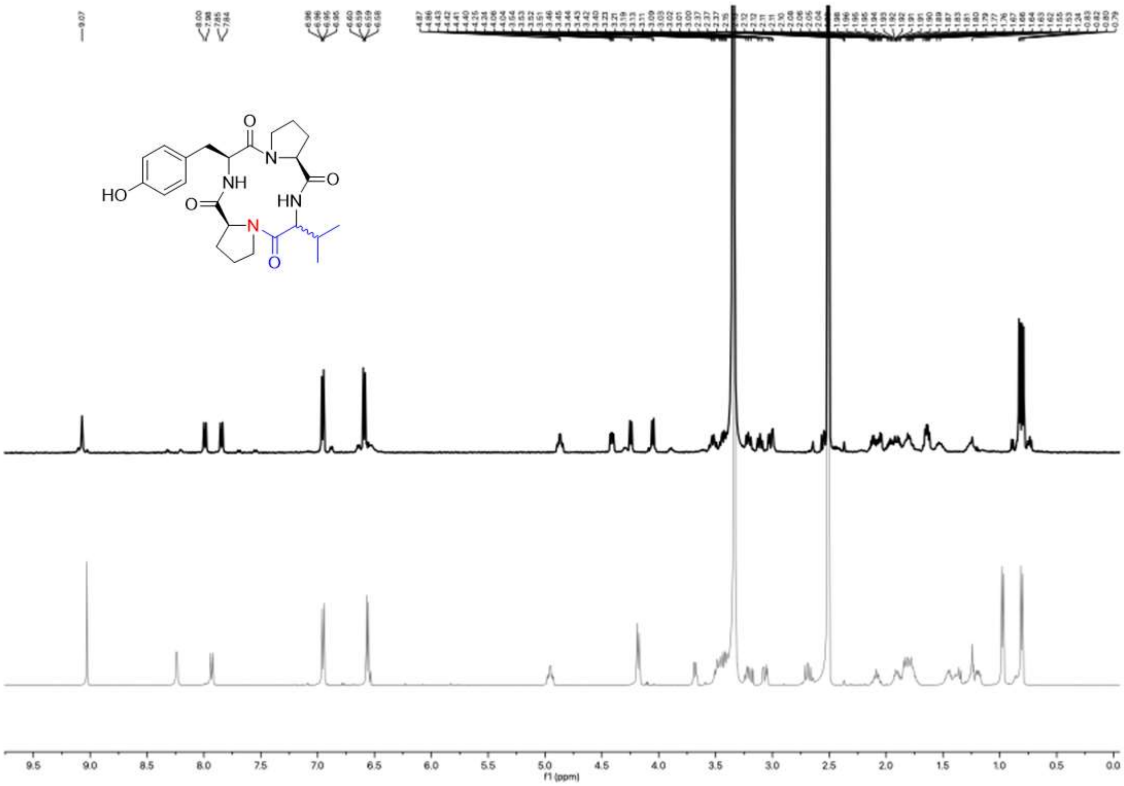

The comparation of ^{1}H-NMR spectrum for Cyclo-(Pro-Tyr-Pro-*d*Val) and Cyclo-(Pro-Tyr-Pro-Val) (DMSO-*d6*, 500 MHz)

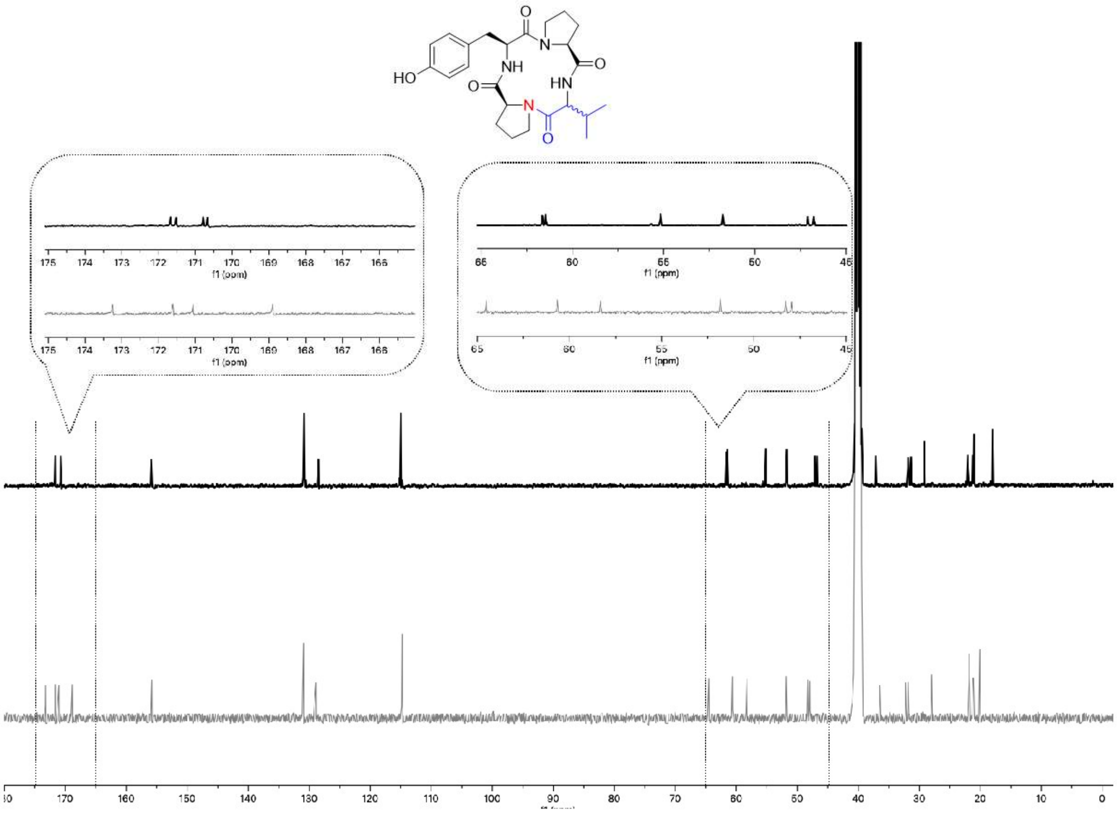

The comparation of ^{13}C-NMR spectrum for Cyclo-(Pro-Tyr-Pro-*d*Val) and Cyclo-(Pro-Tyr-Pro-Val) (DMSO-*d6*, 500 MHz)

The comparation of these corresponding tetracyclic peptides further verified that the cyclized reaction from our *b*-thiolactone reaction does not introduce any epimerization.

2.2.3.4.4 Synthesis and the cyclization attempt of H-Pro-Phe-Pro-Val-S-propyl

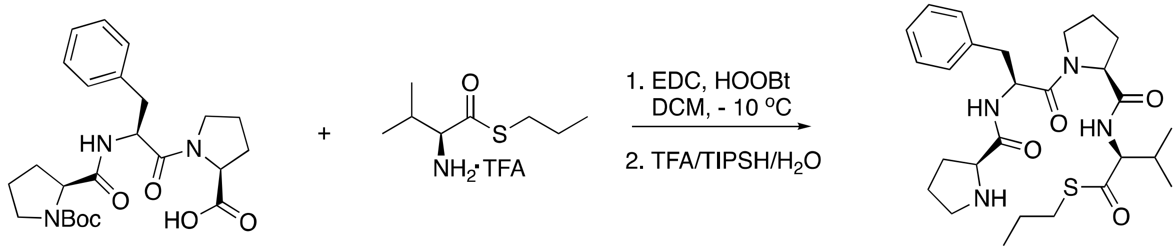

According to the general procedure A and B, the side chain fully protected tripeptide Boc-Pro-Phe-Pro-OH was generated from SPPS on a 0.04 mmol scale. The resulting tripeptide Boc-Pro-Phe-Pro-OH was coupled with H-Val-S-propyl to afford the desired tetrapeptide following the general procedure C. Purification of the crude product using preparative HPLC (20 to 70% solvent B over 20 min, Higgins Analytical Proto 200 5 µm 250 × 10 nm C18 column) afforded peptide H-Pro-Phe-Pro-Val-S-propyl as a white solid after lyophilization (5.6 mg, 27%).
Analytical HPLC: t_R = 6.78 min (40 to 80% solvent B over 20 min, Higgins Analytical Proto 200 5 µm 150 × 2.0 nm C18 column).
ESI-LRMS: calcd. for $C_{27}H_{41}N_4O_4S$ Exact Mass: $[M+H]^+$: 517.2849 (*m/z*); found $[M+H]^+$: 517.2647.

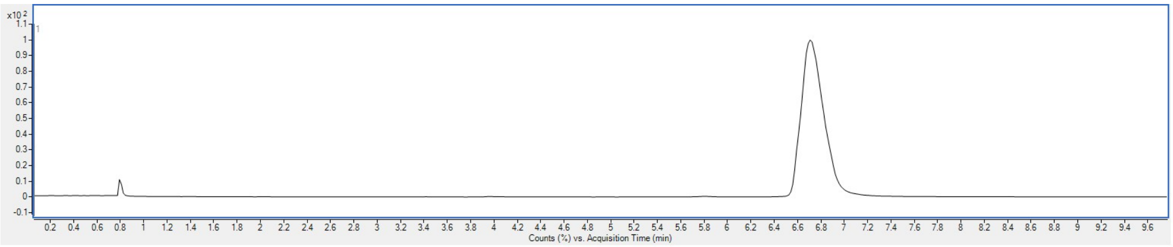

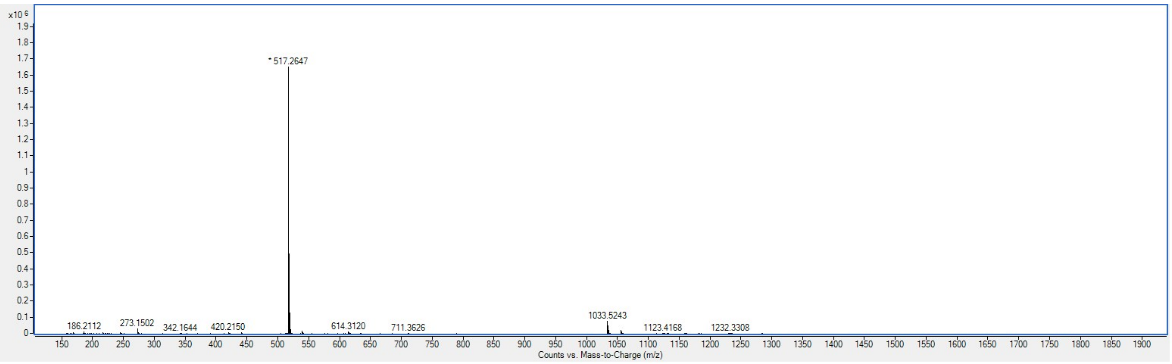

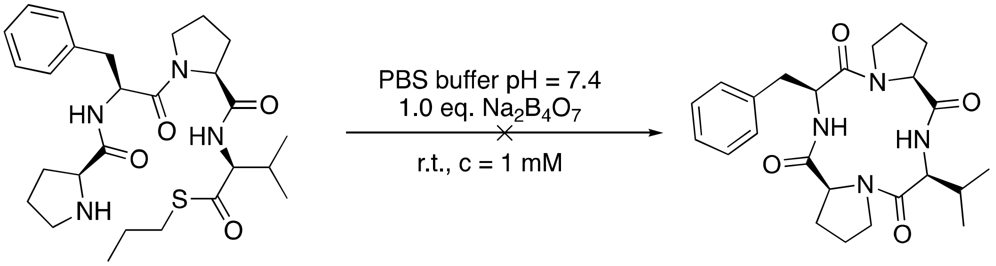

According to the general procedure D and E, H-Pro-Phe-Pro-Val-S-propyl (1.500 mg, 0.0095 mmol) was dissolved in 9.5 mL cyclization buffer (pH = 7.4). No cyclization reaction occurred after stirring at room temperature for 24h.

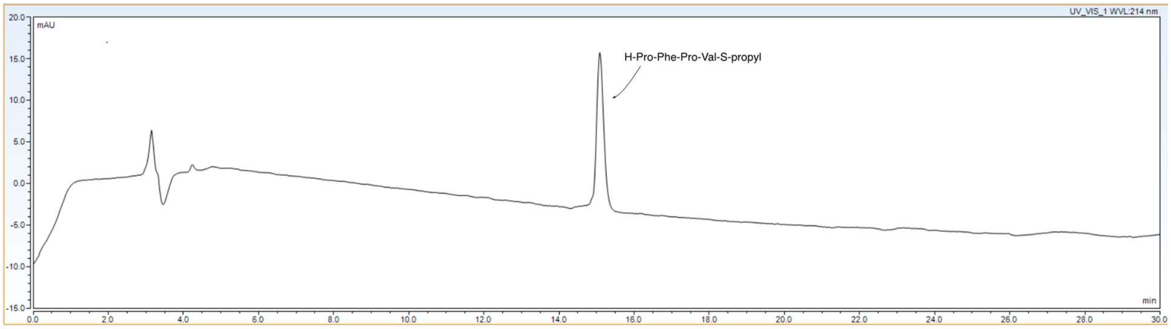

The crude HPLC trace of H-Pro-Phe-Pro-Val-S-propyl cyclization reaction after 24h at room temperature

2.2.3.4.5 Synthesis H-D-Pro-D-Phe-D-Pro-D-PenThiolactone (SS9)

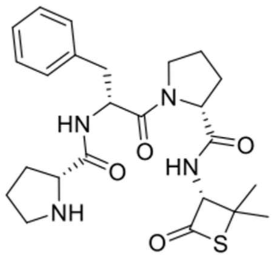

Chemical Formula: $C_{24}H_{32}N_4O_4S$
Exact Mass: 472.2144

SS9

According to the general procedure A and B, the side chain fully protected tripeptide Boc-D-Pro-D-Phe-D-Pro-OH was generated from SPPS on a 0.04 mmol scale. The resulting tripeptide Boc-D-Pro-D-Phe-D-Pro-OH was coupled with D-PenThiolactone TFA salt to afford the desired tetrapeptide following the general procedure C. Purification of the crude product using preparative HPLC (20 to 70% solvent B over 20 min, Higgins Analytical Proto 200 5 µm 250 × 10 nm C18 column) afforded peptide H-Pro-Phe-Pro-Val-S-propyl as a white solid after lyophilization (5.6 mg, 27%).
Analytical HPLC: t_R = 5.98 min (40 to 80% solvent B over 20 min, Higgins Analytical Proto 200 5 µm 150 × 2.0 nm C18 column).
ESI-LRMS: calcd. for $C_{25}H_{33}N_4O_4S$ Exact Mass: $[M+H]^+$: 473.2223 (*m/z*); found $[M+H]^+$: 473.2007.

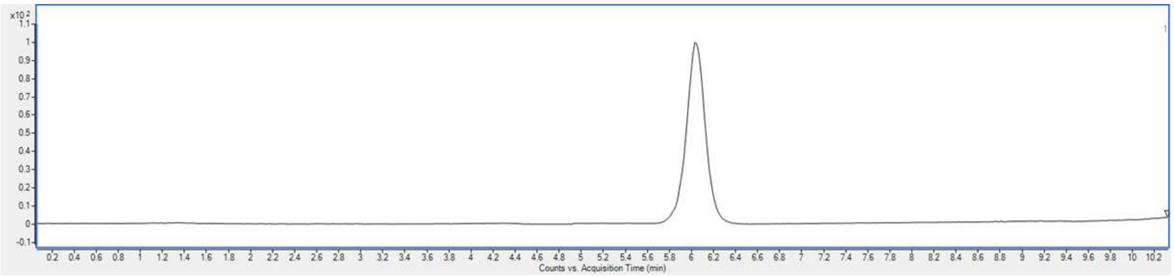

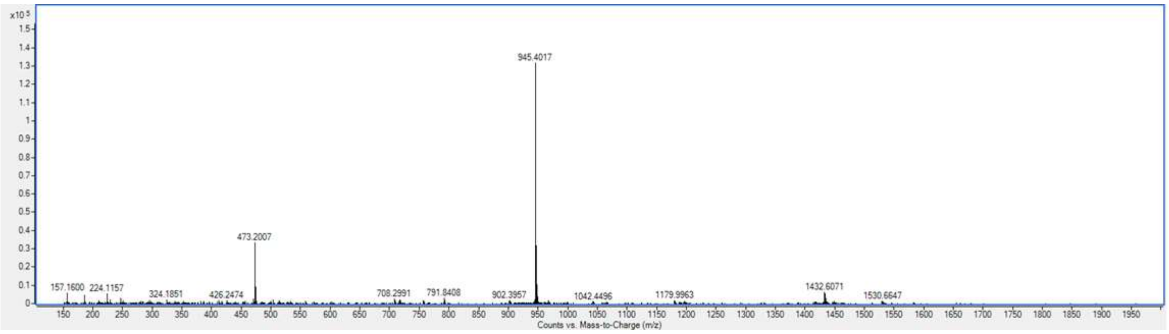

Cyclization of H-D-Pro-D-Phe-D-Pro-D-PenThiolactone (**SS9**)

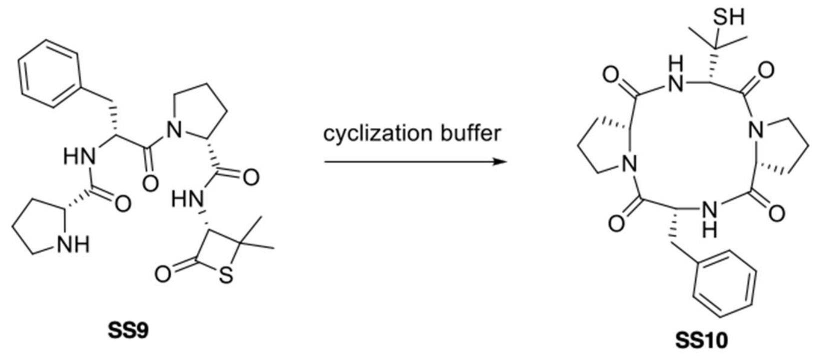

According to the general procedure D, **SS9** (5.00 mg, 0.011 mmol) was dissolved in 11.5 mL cyclization buffer with $Na_2B_4O_7$ (pH = 7.4). After 6h at room temperature, the cyclization reaction was complete (checked by LC-MS). Purification of the crude peptide using preparative HPLC (30 to 80% solvent B over 20 min, Higgins Analytical Proto 200 5 µm 250 × 10 nm C18 column) afforded peptide 14t as a white solid after lyophilization (2.60 mg, 52%).

Analytical HPLC: t_R = 11.2 min (30 to 80% solvent B over 20 min, Higgins Analytical Proto 200 5 µm 150 × 2.0 nm C18 column).

ESI-LRMS: calcd. for $C_{25}H_{33}N_4O_4S$ Exact Mass: $[M+H]^+$: 473.2223 (*m/z*); found $[M+H]^+$: 473.2007.

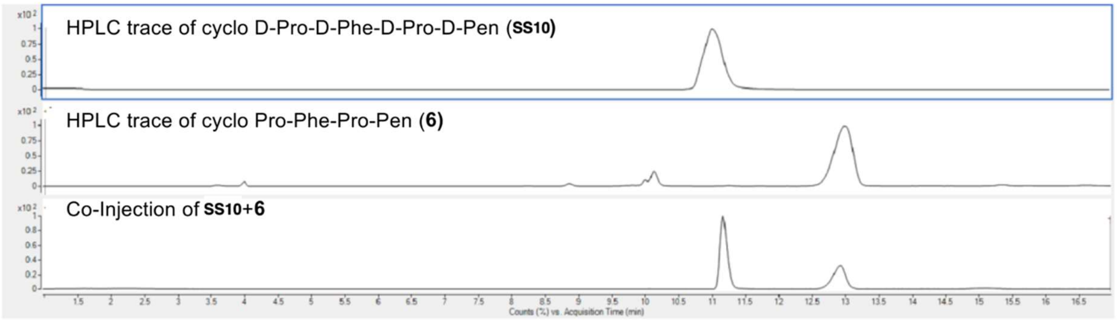

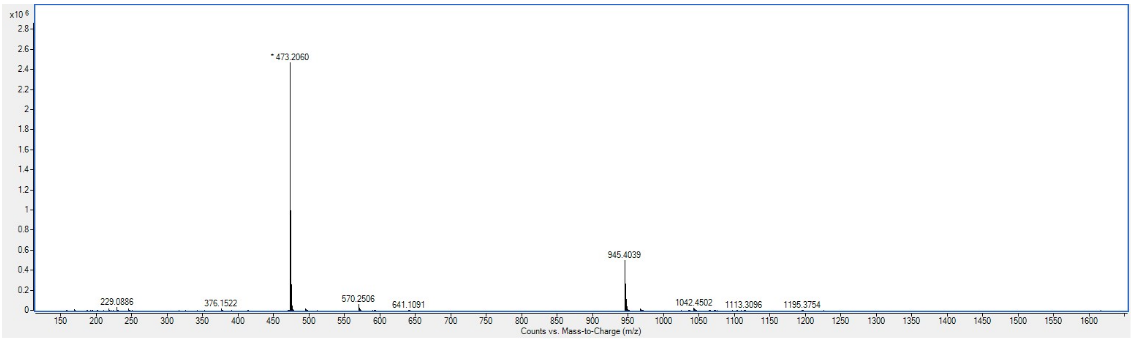

2.2.3.5 Borax Kinetic Studies.

Kinetic studies involving ^{11}B NMR spectra of Borax (1 equivalent) showed two peaks at 2 and 20 ppm,

representing the boron complex and $B(OH)_4^-$, respectively (Supplementary Figure 18). Addition of an

Supplementary Figure 18 ^{11}B NMR Spectra of Peptide and Borax

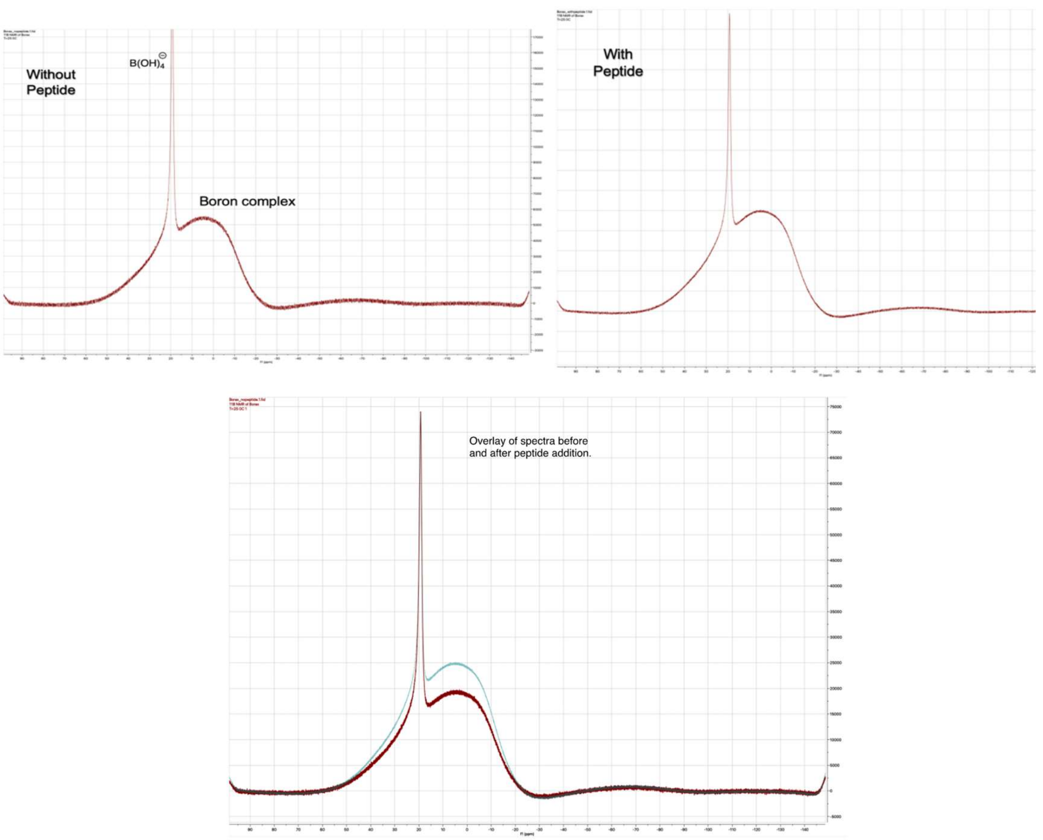

equal molar ratio of peptide **5** did not induce any spectral shifts or new peaks during the 5-minute to 8-hour observation period. Overlaying the spectra before and after peptide addition revealed no discernible changes, indicating no interaction between the peptide and Borax.

The investigation into reaction rates progressed by altering the pH of the reaction. At pH=7.45, Borax presence enhanced cyclization yield by 10% (Supplementary Figure 19a; 66% vs 56%) and reduced dimer byproduct formation to 2.1% from 4%. Shifting the pH higher or lower resulted in comparable yields but notably increased byproduct formation to 7.7% and 9.3%, respectively (Supplementary Figure 19c, d).

The kinetic plot of the reaction, utilizing the equation [−ln (1 −%yield)] against time at various pH levels, exhibits distinct rate constants (Supplementary Figure 20). Elevated pH levels correspond to higher rate constants, indicating an accelerated reaction rate.

Supplementary Figure 19 Cyclization Rate at Different Buffer pH.[a]

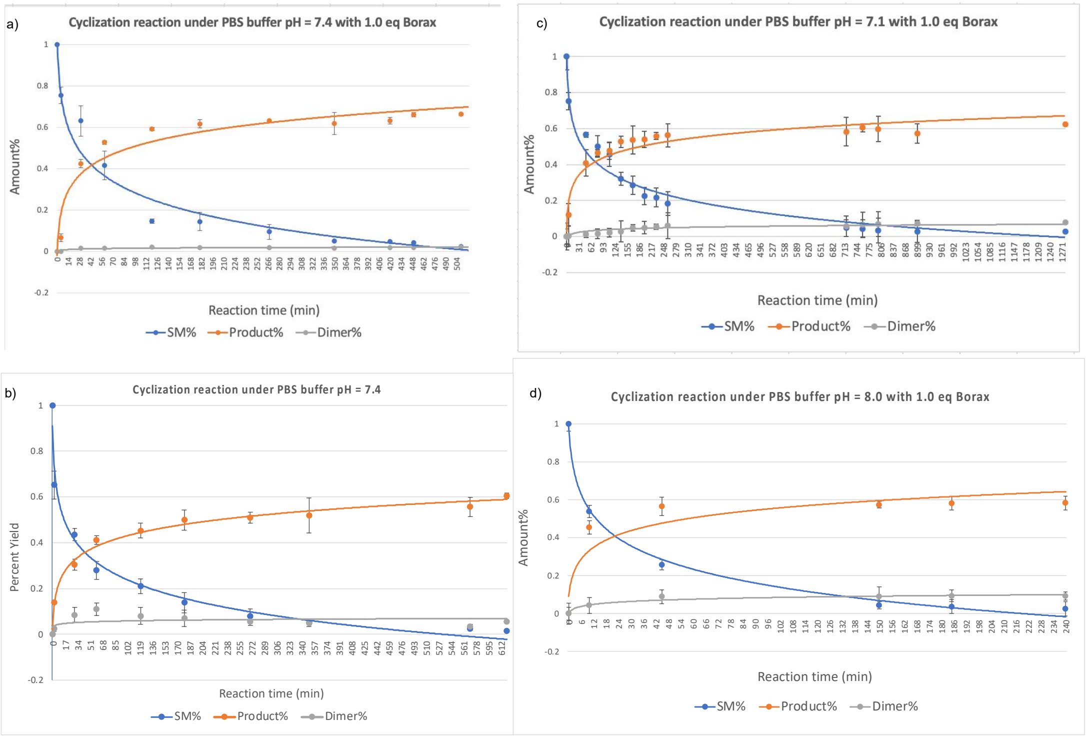

a. Column heights reported on the graph represent mean values, error bars represent the SD.

Supplementary Figure 20 Rate Disparity at Different pH.

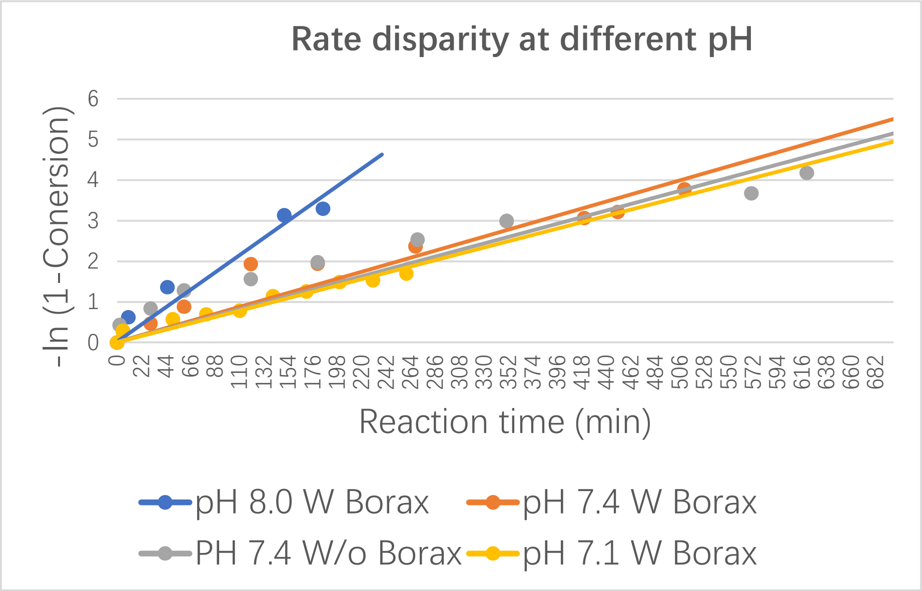

We investigated the influence of borax concentration on cyclization. Borax ($Na_2[B_4O_5(OH)_4]\cdot 8H_2O$)

Supplementary Figure 21 Rate Studies[a]

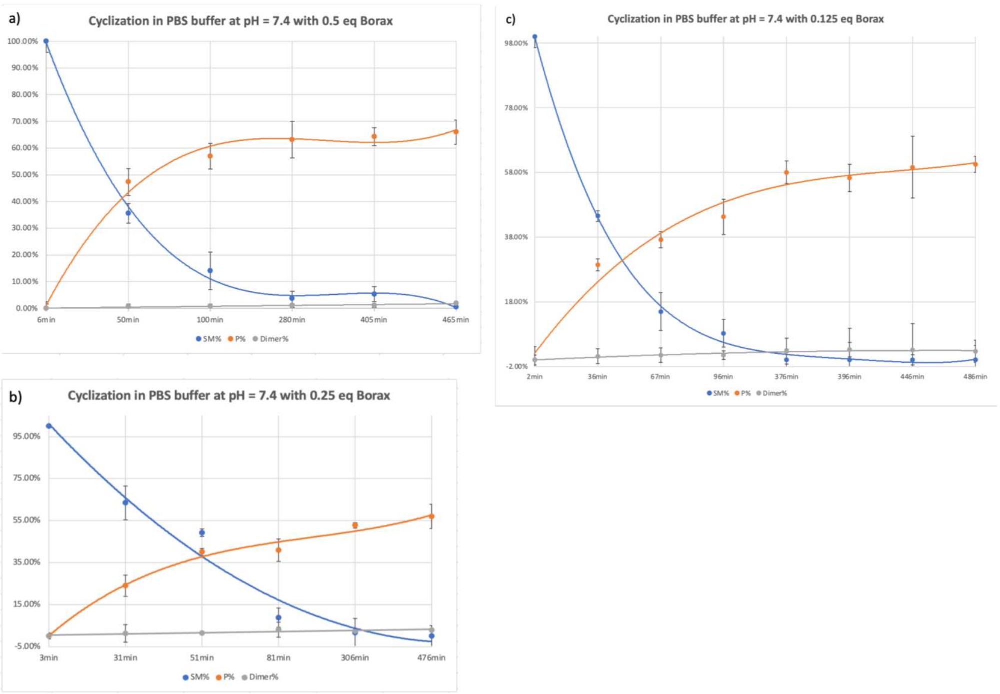

a. Column heights reported on the graph represent mean values, error bars represent the SD.

supplies 4 equivalents of boron per molar molecule. Across different concentrations (0-1 equivalent of borax), overall product and byproduct yields showed no significant differences (Supplementary Figure 21). However, the kinetic plot using varied concentrations revealed distinct rate trends (Supplementary Figure 22). Utilizing GraphPad Prism 6, linear regression and slope calculations were generated (Supplementary Figure 23). Notably, at 1 molar equivalent of borax, cyclization was markedly accelerated with a slope of 0.00759, whereas 0.5 equivalent displayed a smaller slope of 0.002703. Conversely, there was no substantial rate disparity observed among 0.25, 0.125, and 0 equivalents of

Supplementary Figure 22 Rates at Various Equivalences of Borax

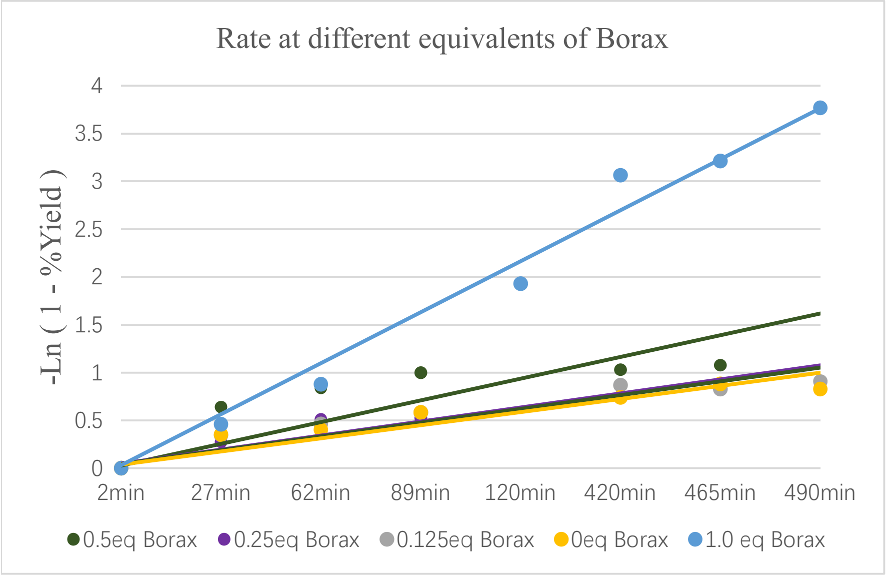

borax, with slopes around 0.0019. Overall, stoichiometric borax (1 equivalent) accelerated cyclization as a first-order reaction, albeit its catalytic effect was weak, and NMR spectra did not evidence borax-peptide binding. Borax improved overall yield by 10% and reduced byproduct formation by 50%.

Supplementary Figure 23. Rate Slope Calculation

	0.5eq	0.25eq	0.125eq	0eq	1.0 eq
Best-fit values					
Slope	0.002703 ± 0.0008404	0.001965 ± 0.0004134	0.001998 ± 0.0003198	0.001887 ± 0.0003088	0.007591 ± 0.0006080
Y-intercept when X=0.0	-0.005406	-0.003931	-0.003996	-0.003773	-0.01518
X-intercept when Y=0.0	2.000	2.000	2.000	2.000	2.000
1/slope	370.0	508.8	500.5	530.0	131.7
95% Confidence Intervals					
Slope	0.0005421 to 0.004864	0.0009024 to 0.003028	0.001215 to 0.002780	0.001131 to 0.002642	0.006103 to 0.009079
Goodness of Fit					
Sy.x	0.5321	0.2618	0.2556	0.2468	0.4885
Is slope significantly non-zero?					
t	3.216	4.754	6.248	6.110	12.48
DF	5.000	5.000	6.000	6.000	6.000
P value	0.0236	0.0051	0.0008	0.0009	< 0.0001
Deviation from zero?	Significant	Significant	Significant	Significant	Significant
Data					
Number of X values	6	6	7	7	7
Maximum number of Y replicates	1	1	1	1	1
Total number of values	6	6	7	7	7
Number of missing values	2	2	1	1	1
Equation	Y = 0.002703*X - 0.005406	Y = 0.001965*X - 0.003931	Y = 0.001998*X - 0.003996	Y = 0.001887*X - 0.003773	Y = 0.007591*X - 0.01518

2.3 Computational Studies.

The present study investigates the reaction mechanisms originating from β-thiolactone substituted and regular thioester substituted intermediates via density functional theory (DFT) calculations. In line with previous computational approaches[7] for studying aminolysis reaction mechanisms, the equilibrium geometries and vibrational frequencies of all intermediate and transition state structures were calculated using the BP86 functional with Grimme's dispersion correction (D3BJ) in conjunction with the 6-31G(d,p) basis set. No restrictions were imposed during the initial geometry optimizations. A vibrational analysis was conducted to determine the nature of each stationary point (minimum or saddle point). Conformational searches were employed for intermediates and transition state geometry to ensure they were at the lowest confirmation. Furthermore, solvent effects in aqueous conditions were considered using an implicit solvent model based on density (SMD) during optimization and frequency calculations. To obtain more accurate energy barrier values, we also used more complete basis sets, namely,

6-311++G(2d,p), to perform single-point calculations to obtain electronic energy in the gas phase. Additional solvation free energy was calculated at M05-2X/6-31G(d) level. The final free energy of each molecule was obtained following the routine shown in the reference[8], which is the summation of high-level electronic energy in the gas phase and Gibbs free energy provided by frequency calculations and the solvation free energy. Gaussian 16[9] software was used for all DFT calculations, and the conformational search was performed by CREST[10] combined with xTB[11] software.

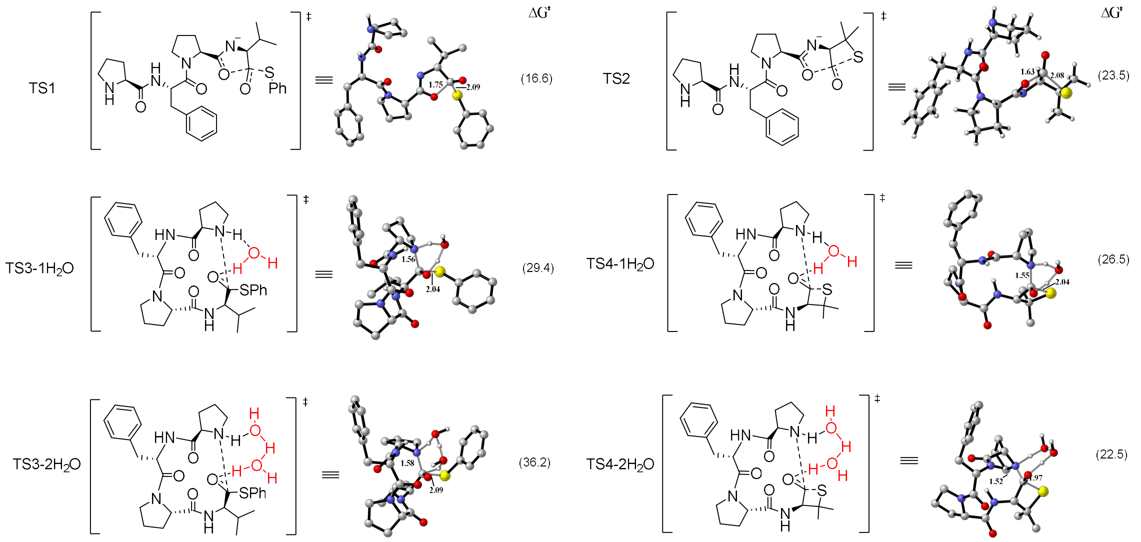

Supplementary Figure 28. The comparison of Gibbs free energy barrier and transition state structures of the route I and route II reaction pathways between β-thiolactone and conventional thioester substitute molecules. Scenarios with one and two additional water molecules in route II, which is direct ring closure reaction pathway, were considered. Hydrogen atoms associated with carbon atoms have been purposefully omitted to enhance the visual clarity of the molecular structures. The energy barrier is denoted in units of kcal/mol.

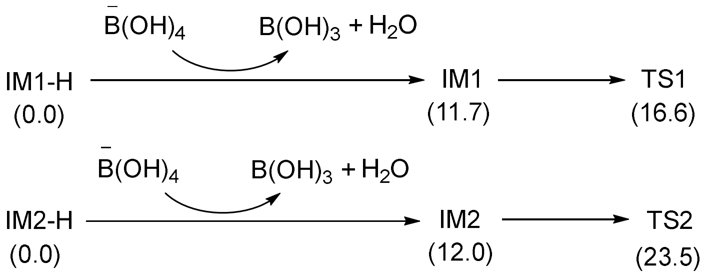

Supplementary Figure 29. The schematic of Route I including endothermic proton transfer process between β-thiolactone and conventional thioester substitute molecules. The values in parentheses are the relative energy respect to the initio reactants which is denoted in units of kcal/mol.

Cartesian coordinates of all the intermediates and transition state structures.

IM1

C	-0.25970000	-2.26173800	2.06418400
O	-0.41223600	-1.24350700	2.77828600
N	-1.17298700	-2.66979400	1.14895700
H	-0.83178300	-3.41391400	0.52656800
C	-2.26950200	-1.80267600	0.74436600
H	-2.84786200	-1.54357900	1.64314700
C	-3.17161500	-2.55371200	-0.26603600
H	-3.58687300	-3.44357700	0.23561000
H	-2.52822700	-2.89588700	-1.09581200
C	-4.28044300	-1.67179900	-0.79955300
C	-5.50493100	-1.55983200	-0.11160500
C	-4.07939500	-0.89039800	-1.95573300
C	-6.50568100	-0.68631100	-0.56656500
H	-5.67042700	-2.16201200	0.78869300
C	-5.07720700	-0.01666300	-2.41398400
H	-3.12578100	-0.96184500	-2.48920300
C	-6.29426500	0.08846900	-1.71966600
H	-7.45181400	-0.61180100	-0.02113000
H	-4.90358500	0.58256900	-3.31338100
H	-7.07429500	0.76851900	-2.07591200
C	-1.72258200	-0.53135500	0.05913500
O	-0.84405000	-0.64186300	-0.83673700
C	0.96688400	-3.17822500	2.18603600
C	2.28139400	-2.33910600	2.30554600
H	0.80103400	-3.80888600	3.07557700
C	1.99388300	-3.26794100	0.09462000
C	3.07410900	-2.68752500	1.02183100
H	2.83760600	-2.59434500	3.22070600
H	2.04155000	-1.26510800	2.35084900
H	2.38313700	-3.90645900	-0.71229200
H	1.40473100	-2.45318700	-0.36571100
H	3.83509300	-3.46054100	1.22955500
H	3.58332400	-1.81099600	0.59342200
N	1.08233300	-4.04399100	0.97859600
H	1.59548200	-4.88591100	1.27035300
C	-3.35619200	0.91700100	1.35584500
C	-4.06007800	2.13705800	0.74967900
H	-2.92369500	1.14728600	2.34727200
H	-4.02743200	0.05429500	1.45294800
C	-2.89485200	2.94158800	0.15119300
H	-4.74936100	1.80189800	-0.04430800

H	-4.63830100	2.69793000	1.50063800
H	-3.20357700	3.64761800	-0.63421400
H	-2.38449800	3.51220400	0.94692100
N	-2.26638500	0.65796600	0.38947100
C	-0.45180800	2.33672800	-0.31694800
O	-0.04973400	2.95945700	-1.36711400
N	0.14407600	2.10175300	0.84657000
C	1.52239300	2.56427000	1.03118200
H	1.72749900	2.51603100	2.12008200
C	-1.92445400	1.86549900	-0.39700400
C	2.46843700	1.49251100	0.47630900
O	3.12043900	0.73352700	1.19279100
C	3.65162300	0.00046600	-1.55970400
C	3.21122900	-1.12932200	-2.27541900
C	4.96994300	0.05401200	-1.06437500
C	4.08815300	-2.20380100	-2.49340600
H	2.18402600	-1.16811700	-2.64981800
C	5.83158900	-1.03441600	-1.26511800
H	5.31352600	0.93897500	-0.52210100
C	5.39427800	-2.16302600	-1.97994600
H	3.74310700	-3.07841600	-3.05321900
H	6.85166300	-0.99609600	-0.87031900
H	6.07287900	-3.00639400	-2.13985000
S	2.51514300	1.37119000	-1.35268500
H	-2.13544000	1.64637500	-1.45950200
C	1.94178700	3.99684600	0.59101600
C	3.37327300	4.28799200	1.06803500
C	0.95031800	5.02808900	1.14605000
H	1.90917100	4.04586300	-0.50907100
H	4.10200200	3.57702800	0.63939200
H	3.68859500	5.30416500	0.77453300
H	3.44565000	4.21758300	2.16926600
H	-0.06326600	4.86496400	0.74580100
H	0.89697800	4.97422500	2.24917700
H	1.26272800	6.05194400	0.87553900

IM2

C	0.29441000	2.75445300	1.14763300
O	0.30095300	2.15893000	2.24851900
N	1.27190200	2.62576600	0.20722300
H	0.98627800	3.01656800	-0.70294400
C	2.16970100	1.47517300	0.21789900
H	2.39208900	1.25185400	1.27104800

C	3.47944200	1.77219500	-0.54626700
H	3.96146900	2.65095900	-0.08751100
H	3.22052700	2.02392400	-1.58978300
C	4.39380000	0.56691100	-0.49767700
C	5.31567000	0.41143800	0.55700800
C	4.25801700	-0.47458700	-1.43809700
C	6.07787000	-0.76135400	0.67631100
H	5.42549500	1.21499600	1.29378000
C	5.01545500	-1.65019000	-1.31960000
H	3.53915900	-0.36572000	-2.25718400
C	5.92484300	-1.79879000	-0.25888600
H	6.78910100	-0.86688800	1.50176400
H	4.89464100	-2.45108400	-2.05596000
H	6.51495100	-2.71571500	-0.16475700
C	1.42570700	0.30423200	-0.45613600
O	0.88444600	0.48550100	-1.57934600
C	-0.81581000	3.74528000	0.76556500
C	-2.22575700	3.17780900	1.14745300
H	-0.59924500	4.68433800	1.30201100
C	-1.84766700	3.07949700	-1.22829400
C	-2.98588800	3.12725200	-0.19732500
H	-2.72916300	3.81217500	1.89289900
H	-2.11903700	2.17234700	1.58655700
H	-2.14429100	3.37650100	-2.24588300
H	-1.41461100	2.06450700	-1.27987500
H	-3.57611900	4.04948000	-0.34468700
H	-3.67299400	2.26880400	-0.27073000
N	-0.80590800	4.00277700	-0.70346600
H	-1.16054700	4.95835200	-0.83559900
C	2.06764200	-1.26161700	1.42937100
C	2.14026100	-2.78883000	1.29700100
H	1.48009600	-0.95547800	2.31493200
H	3.06075300	-0.79365100	1.47887900
C	0.80299000	-3.14124800	0.62228000
H	2.98785000	-3.05722400	0.64258100
H	2.28109600	-3.28397900	2.27034100
H	0.80662700	-4.11999500	0.11968800
H	-0.00788600	-3.14011500	1.37094000
N	1.35582800	-0.87257500	0.19379400
C	-0.93309100	-1.65190800	-0.55162100
O	-1.51030200	-2.19030200	-1.56486000
N	-1.46443300	-0.88747100	0.40049000
C	0.56566200	-1.98362800	-0.37900300
H	0.96731100	-2.22841100	-1.37791600

C	-2.87207300	-0.65056400	0.22680000
C	-3.91770100	-1.71902900	0.75458800
H	-3.12703100	0.30790500	0.72751200
S	-4.97694200	-1.53251400	-0.80586100
C	-4.65584900	-1.27212300	2.01429500
H	-5.46718100	-1.97074900	2.27930400
H	-3.94378100	-1.24561100	2.85917100
H	-5.08501700	-0.26358700	1.89551700
C	-3.36917300	-3.13686700	0.89366900
H	-2.82216600	-3.44162600	-0.00928300
H	-2.67822600	-3.18112200	1.75372100
H	-4.18896600	-3.85014600	1.08284800
C	-3.48392900	-0.51993500	-1.17439800
O	-3.22372200	0.15808700	-2.15797500

IM3

C	-1.29473500	0.02625600	1.05145000
O	-1.06667900	1.24767900	1.23947500
N	-1.71675900	-0.48515800	-0.12414400
H	-1.79175000	-1.52422800	-0.24098400
C	-2.07678600	0.32498300	-1.28914400
C	-0.87214800	1.18813700	-1.71772000
O	0.10348400	0.59249000	-2.24850000
C	-1.19415200	-1.01067400	2.17039400
C	-2.39699500	-0.90469300	3.15411100
H	-1.19448300	-2.02536600	1.71239300
C	-0.31397200	-1.62305100	4.22920900
C	-1.79613200	-1.30455000	4.53549900
H	-3.22426100	-1.55497900	2.83185100
H	-2.76640000	0.13351400	3.17426000
H	-0.19928100	-2.71160100	4.02287500
H	0.36897600	-1.36341100	5.05566800
H	-2.31177800	-2.16772400	4.98459300
H	-1.87262800	-0.46386400	5.24344100
N	-0.04035000	-0.78901900	3.04677500
H	0.82303300	-1.05558600	2.56081300
C	-1.74226300	3.43885000	-0.82424400
C	-0.88585500	4.69279200	-0.57388800
H	-2.05787600	2.95885500	0.11147000
H	-2.62088000	3.66810500	-1.45048800
C	0.08647400	4.71193800	-1.76531000
H	-1.50149700	5.60150400	-0.49913300
H	-0.32556000	4.57933500	0.36989300

H	-0.41381800	5.09262800	-2.67157500
H	0.99141600	5.31309100	-1.59211100
N	-0.81851500	2.53326000	-1.55429100
C	1.64793600	2.78734800	-1.12303300
O	2.79426500	3.01402300	-1.54540100
N	1.38454900	2.22289700	0.09352200
C	2.45648000	1.61445200	0.87285200
C	0.42491200	3.21929000	-1.95876300
C	2.57464500	0.15660000	0.39032000
O	1.71787000	-0.70301200	0.59860200
C	3.93990400	-1.86889500	-0.95465900
C	3.83379800	-2.83528600	0.06426600
C	3.96590200	-2.25298400	-2.30775500
C	3.74108600	-4.19092700	-0.28189700
H	3.82121500	-2.52842900	1.11300000
C	3.89797500	-3.61380400	-2.64083500
H	4.03629400	-1.49149300	-3.08955200
C	3.77862300	-4.58181000	-1.63101400
H	3.65062900	-4.94395200	0.50667000
H	3.92436400	-3.91477000	-3.69231000
H	3.71558100	-5.64186700	-1.89423200
S	4.07671500	-0.12863600	-0.55362100
H	0.67570100	2.96855600	-3.00039600
H	0.42263700	1.94246600	0.35426300
O	-1.53949700	-3.15744400	-0.75349600
H	-2.15222600	-3.29600600	-1.49984900
H	-0.68273800	-2.82651000	-1.18664300
O	0.66006700	-2.02832700	-1.72234900
H	1.19001300	-1.86272600	-0.90982000
H	0.37715300	-1.10216000	-1.97111500
C	2.18681300	1.70023900	2.39103700
C	2.08275500	3.17128700	2.81991600
C	3.28545500	0.96310300	3.17327300
H	1.21983800	1.19782500	2.58448400
H	1.29353800	3.69841000	2.25980000
H	1.84374800	3.23938100	3.89431600
H	3.03587900	3.70387100	2.64946900
H	3.32863000	-0.10873200	2.91535700
H	4.28030400	1.40001000	2.97307800
H	3.09619300	1.03618600	4.25671700
H	3.38755500	2.15041100	0.61612200
C	-3.48518800	0.94943500	-1.12545100
H	-3.71053300	1.56042100	-2.01683000
H	-3.51219600	1.60531000	-0.24438000

C	-4.50194200	-0.16045300	-0.95851300
C	-4.95409700	-0.52433800	0.32450600
C	-4.94360800	-0.90648100	-2.06939600
C	-5.82804300	-1.60900900	0.49694000
H	-4.61459900	0.04836200	1.19394400
C	-5.81706100	-1.99188300	-1.90154800
H	-4.59899900	-0.63378300	-3.07360700
C	-6.26017800	-2.34773500	-0.61642600
H	-6.17020500	-1.87732300	1.50158500
H	-6.15396700	-2.55925300	-2.77498000
H	-6.94080700	-3.19460500	-0.48463900
H	-2.14984800	-0.39763600	-2.12022100

IM4

C	-1.02478700	-0.34395500	0.82962900
O	-1.29802400	0.40734700	1.78707900
N	-1.28456500	-0.07961400	-0.47779200
H	-0.77118700	-0.68764000	-1.14685800
C	-1.91014600	1.15589400	-0.99019200
C	-0.76628000	2.19357700	-1.11849400
O	-0.07585700	2.15468300	-2.15843700
C	-0.39299300	-1.73117400	1.07914700
C	-0.49643800	-2.13937000	2.55321200
H	-0.81507000	-2.46929000	0.38440700
C	1.73629000	-1.17373600	2.12141200
C	0.69764000	-1.43143200	3.24287700
H	-0.40253400	-3.23449500	2.62447300
H	-1.46488000	-1.84527400	2.98006300
H	2.69665300	-1.68428900	2.27234200
H	1.92496400	-0.10373300	1.96466900
H	1.12148900	-2.04502700	4.05138700
H	0.37052900	-0.47240500	3.66800700
N	1.11380700	-1.75172900	0.87119300
C	-1.12905600	3.41629900	1.13839900
C	-0.21175300	4.50161600	1.73098900
H	-1.28819700	2.58066200	1.83667200
H	-2.10329900	3.81938800	0.82429400
C	1.20097300	4.02553400	1.35224700
H	-0.41917600	5.47697700	1.25865500
H	-0.35443300	4.60887200	2.81703700
H	1.96385300	4.81618500	1.39755200
H	1.51913300	3.19695100	2.00910700
N	-0.36189400	2.93702800	-0.04371000

C	2.02905200	2.44393800	-0.52710900
O	3.00258300	2.73988800	-1.23131300
N	1.80708000	1.16983300	-0.04103600
C	1.00532600	3.50161800	-0.08572500
H	1.07928100	4.31795700	-0.82521400
C	2.28177100	0.03726300	-0.83879100
C	3.79805100	-0.33104400	-0.75800100
S	3.42167700	-2.17323400	-0.66447500
C	4.53857600	0.20203500	0.46757500
H	5.55505300	-0.22288900	0.50844000
H	4.62264400	1.29937100	0.38443600
H	4.02967600	-0.03468900	1.41099100
C	4.55829700	0.02249000	-2.03969500
H	4.04820600	-0.38509500	-2.92750900
H	4.60679500	1.11983300	-2.13342500
H	5.58312300	-0.38402000	-2.01179000
C	1.62425700	-1.37194200	-0.61142100
O	0.72259800	-1.74512300	-1.46712700
H	1.32634300	-2.81342500	0.89889100
C	-3.22415500	1.51687400	-0.26314900
H	-3.03502200	1.72382200	0.79665800
H	-3.61404900	2.43642900	-0.73363600
C	-4.22746500	0.38852900	-0.38836600
C	-4.38416700	-0.54419700	0.65565100
C	-4.97875800	0.21920800	-1.56810400
C	-5.27152500	-1.62362200	0.52420700
H	-3.80064400	-0.41807000	1.57368200
C	-5.86771100	-0.85816400	-1.70254000
H	-4.86719500	0.94068800	-2.38584300
C	-6.01525600	-1.78438100	-0.65623000
H	-5.38247900	-2.33949400	1.34507100
H	-6.44830400	-0.97331000	-2.62347200
H	-6.70889900	-2.62469700	-0.75981800
H	-2.16504000	0.92893900	-2.03717700
H	2.04043900	0.24942300	-1.89660600
H	0.89230000	1.05565100	0.40279900
O	0.32045200	-4.32711700	-1.54527700
H	0.44695300	-3.31584000	-1.59510900
H	-0.64415200	-4.44925900	-1.46769500
O	1.50227500	-4.42138600	0.78671600
H	0.96856300	-4.90197800	1.44652100
H	1.01008000	-4.54058900	-0.09927000

TS1

C	-3.38998000	-2.23653900	0.56585900
O	-2.78610300	-2.07868100	1.65171300
N	-3.93607400	-1.21302500	-0.14214500
H	-4.23014400	-1.48542800	-1.08962500
C	-3.55201900	0.16766400	0.12066100
H	-3.67667700	0.35460100	1.19700800
C	-4.44948200	1.12961000	-0.69318100
H	-5.49514300	0.97605500	-0.37940500
H	-4.36421500	0.84980600	-1.75815600
C	-4.03369300	2.57158700	-0.49536300
C	-4.55480600	3.32792900	0.57328300
C	-3.05311200	3.15428100	-1.32387700
C	-4.10415900	4.63590500	0.81214300
H	-5.31542200	2.88191800	1.22373200
C	-2.60182800	4.46221700	-1.08939900
H	-2.63489900	2.56935600	-2.14993100
C	-3.12479400	5.20641700	-0.01842300
H	-4.51873800	5.21036200	1.64662000
H	-1.83991900	4.89958900	-1.74232400
H	-2.77354400	6.22637700	0.16623500
C	-2.08291100	0.37233800	-0.30698600
O	-1.69714500	-0.03989500	-1.43092300
C	-3.58891600	-3.62096300	-0.07543000
C	-2.27538800	-4.42387700	-0.02160600
H	-4.41946300	-4.12066800	0.45156900
C	-2.58541200	-3.40048800	-2.21812700
C	-1.48653200	-3.81216400	-1.19585200
H	-2.50022500	-5.48890700	-0.20256700
H	-1.76108500	-4.33376300	0.94659800
H	-2.60628600	-4.05469300	-3.10343800
H	-2.41716400	-2.36789100	-2.56578100
H	-0.74874000	-4.50769100	-1.62586300
H	-0.94163100	-2.91582100	-0.85190900
N	-3.91507800	-3.47889000	-1.51849300
H	-4.35491200	-4.36244200	-1.79813800
C	-1.56745800	1.67273400	1.82272300
C	-0.56672500	2.83473900	1.86602200
H	-1.39858500	0.95572000	2.64698600
H	-2.60947900	2.01623400	1.85384000
C	0.68954600	2.24493000	1.20142500
H	-0.96119300	3.67591900	1.27111100
H	-0.38504700	3.18720800	2.89296500
H	1.36308600	3.00684400	0.78287500

H	1.26251200	1.63975300	1.92393700
N	-1.25151100	1.02741200	0.53052600
C	0.96412400	0.06337900	-0.07436500
O	2.00689700	0.19242500	-0.89274400
N	0.75688700	-1.02826900	0.60516900
C	1.80106700	-1.98047000	0.20111700
H	1.41956300	-2.59549000	-0.64509200
C	0.12701200	1.31718400	0.09050300
C	2.97453000	-1.18111400	-0.42047500
O	3.70720700	-1.56679300	-1.34387600
C	4.86141600	0.89262200	0.17936700
C	4.49746000	2.25828100	0.16487300
C	5.93373400	0.46347200	-0.63514300
C	5.19656200	3.17348800	-0.63575600
H	3.66255000	2.59416600	0.78697400
C	6.62098900	1.38068400	-1.44670000
H	6.22565000	-0.58993600	-0.62237900
C	6.25797200	2.73765300	-1.44855100
H	4.90582400	4.22928900	-0.63174700
H	7.44884000	1.03365300	-2.07412100
H	6.80021400	3.45205100	-2.07601600
S	3.95488500	-0.26844100	1.17766200
H	0.08612600	1.83698000	-0.88279800
C	2.19616600	-2.94559800	1.33496500
C	3.35378500	-3.86528500	0.91629200
C	0.97747700	-3.77534600	1.76986500
H	2.52361900	-2.32961400	2.19530000
H	4.26446800	-3.29333200	0.67789200
H	3.59551700	-4.57501700	1.72604800
H	3.07857900	-4.45559200	0.02318000
H	0.11970400	-3.13262200	2.02323700
H	0.66413800	-4.45214400	0.95453000
H	1.21980500	-4.39843700	2.64809600

TS2

C	0.33892300	2.74307900	1.15121900
O	0.28161000	2.11290600	2.22998300
N	1.37117900	2.63905400	0.26404600
H	1.11747600	3.04040800	-0.65223100
C	2.23121800	1.45783100	0.27007600
H	2.45371100	1.21658800	1.31961300
C	3.54166800	1.71719600	-0.50491400
H	4.05534400	2.57641900	-0.04365500

H	3.28021200	1.98696400	-1.54336600
C	4.41824500	0.48390200	-0.47718100
C	5.34775500	0.29129100	0.56458500
C	4.23952700	-0.54379800	-1.42543600
C	6.07509900	-0.90512700	0.66410700
H	5.49072700	1.08439100	1.30692700
C	4.96220300	-1.74294300	-1.32652300
H	3.51630000	-0.40476300	-2.23617600
C	5.87914200	-1.92872500	-0.27829000
H	6.79283900	-1.03969900	1.47959600
H	4.80907700	-2.53269800	-2.06882200
H	6.44213600	-2.86392600	-0.19952900
C	1.43299600	0.32265000	-0.40276000
O	0.85818100	0.53881900	-1.50024900
C	-0.74244600	3.75229800	0.73057500
C	-2.17459100	3.22453300	1.08099000
H	-0.51789300	4.69485800	1.25778200
C	-1.73137600	3.07387300	-1.27981000
C	-2.89725200	3.16459800	-0.28410800
H	-2.68461100	3.88399400	1.79958600
H	-2.10181400	2.22582300	1.54221100
H	-1.99160200	3.35674100	-2.31130300
H	-1.32022300	2.04801700	-1.29869400
H	-3.46590600	4.09438700	-0.46461700
H	-3.59415200	2.31507800	-0.36968200
N	-0.68562300	3.98618800	-0.74217600
H	-1.01976400	4.94557900	-0.89877900
C	2.07597200	-1.33223200	1.41782600
C	2.06293500	-2.85463700	1.22524900
H	1.54765500	-1.03257900	2.34152800
H	3.09145400	-0.91275800	1.43142200
C	0.67271600	-3.11553000	0.61853900
H	2.85784200	-3.13903600	0.51448500
H	2.22865000	-3.39443700	2.17008500
H	0.59657200	-4.07256200	0.08162500
H	-0.09588300	-3.09598300	1.40837900
N	1.32781200	-0.86242200	0.23292100
C	-0.99419800	-1.42810300	-0.36244600
O	-1.58292900	-1.38384800	-1.56745100
N	-1.61980800	-1.04891200	0.71338800
C	0.44615400	-1.91526600	-0.33070700
H	0.76425500	-2.13816500	-1.36263300
C	-2.94890000	-0.59918700	0.30079700
C	-4.13170400	-1.52762500	0.71041100

H	-3.10727200	0.44134500	0.64394100
S	-4.24316600	-2.40970000	-0.95571100
C	-5.39260800	-0.69469400	0.96572000
H	-6.28115500	-1.34057200	1.06523000
H	-5.28252200	-0.11194900	1.90023000
H	-5.57169800	0.01288600	0.13889900
C	-3.85187300	-2.48209500	1.86755900
H	-2.93586300	-3.06587500	1.69060900
H	-3.72652400	-1.92019800	2.81164200
H	-4.69514300	-3.18149400	2.00067400
C	-3.04062400	-0.73319600	-1.24925900
O	-3.36065700	0.14618600	-2.07705400

TS3-2H2O

C	-1.12821600	-0.26430900	0.87578000
O	-1.50192300	0.85265200	1.29590200
N	-1.66914900	-0.86234500	-0.21313800
H	-1.30747200	-1.77676900	-0.48227200
C	-2.62909000	-0.19586900	-1.11529900
C	-1.81955200	0.91587900	-1.83638700
O	-1.06703800	0.53160400	-2.76731300
C	-0.06612900	-1.07629000	1.61060600
C	-0.09225900	-0.84710400	3.13369500
H	-0.26208600	-2.14612300	1.41338800
C	2.15124300	-1.38418000	2.30586100
C	1.37169900	-1.05083800	3.59371600
H	-0.78972800	-1.55904000	3.60267500
H	-0.46367200	0.16663400	3.34790400
H	2.22453200	-2.48045500	2.18267100
H	3.16678600	-0.97157800	2.26622700
H	1.46752200	-1.86935700	4.32374900
H	1.76536300	-0.14020300	4.07042700
N	1.37012800	-0.88259100	1.12863800
H	1.32470700	-1.90518500	0.15701700
C	-2.78965900	2.93559100	-0.57038700
C	-2.21021400	4.36040600	-0.47399300
H	-2.87197200	2.45935700	0.41487400
H	-3.77340500	2.92979800	-1.06826500
C	-1.37137600	4.50993100	-1.75515200
H	-3.00478800	5.11579500	-0.38159700
H	-1.55804800	4.44018700	0.41204400
H	-2.01679100	4.72104300	-2.62465500
H	-0.60513000	5.29690700	-1.69167800

N	-1.80225000	2.20746800	-1.41521200
C	0.58468900	2.98291300	-1.11221600
O	1.63466800	3.41647800	-1.61555300
N	0.47848000	2.46860400	0.14775800
C	1.63780900	1.89418300	0.82551700
C	-0.74158600	3.11298600	-1.91197500
C	1.80337200	0.38396800	0.28537300
O	1.30769900	0.29627200	-0.89935400
C	3.98867600	-1.30488500	-0.68800900
C	4.29191900	-2.52597600	-0.04264000
C	3.72302500	-1.31001800	-2.07433300
C	4.29110800	-3.72966500	-0.76471100
H	4.51633500	-2.52849800	1.02722100
C	3.72097800	-2.51532100	-2.79320000
H	3.50115000	-0.36455700	-2.57525300
C	3.99478600	-3.72867700	-2.14148300
H	4.52012700	-4.66954800	-0.25276900
H	3.49387400	-2.50663100	-3.86333100
H	3.98719000	-4.66863800	-2.70130500
S	3.88588800	0.21601000	0.22912000
H	-0.49394100	2.86085800	-2.95196800
H	-0.41820700	2.03980200	0.40196200
O	1.01440700	-2.77345200	-0.53549900
H	1.82148600	-3.31354400	-0.67441100
H	0.75487000	-2.19954800	-1.63683300
O	0.70043400	-1.42549900	-2.52067600
H	1.12414900	-0.66595000	-1.89346900
H	-0.19178800	-0.99809400	-2.67850900
C	1.60933800	2.16129300	2.34645900
C	1.05874700	3.56719500	2.64999900
C	3.00642900	2.01556900	2.97347900
H	0.92677900	1.43386200	2.81382000
H	0.01056300	3.67355300	2.33161400
H	1.11399100	3.76813000	3.73362800
H	1.64998300	4.34177700	2.12966700
H	3.46403800	1.03493900	2.77631900
H	3.68801700	2.78410200	2.56600300
H	2.95510200	2.15820600	4.06616300
H	2.50350100	2.42584200	0.39204300
C	-3.98138400	0.09005300	-0.42106300
H	-4.63907000	0.59082600	-1.15199100
H	-3.83791600	0.76429400	0.43174600
C	-4.59973600	-1.20539400	0.06002900
C	-4.36770100	-1.65125300	1.37632500

C	-5.35807200	-2.01493900	-0.80803700
C	-4.88267600	-2.87965700	1.81744000
H	-3.78135000	-1.02455000	2.05702700
C	-5.87613500	-3.24338500	-0.36970600
H	-5.54543600	-1.67699900	-1.83376200
C	-5.63799300	-3.67976400	0.94433400
H	-4.69548600	-3.21131500	2.84374100
H	-6.46867000	-3.85934900	-1.05365900
H	-6.04252300	-4.63725400	1.28707600
H	-2.82547900	-0.92592600	-1.91573300

TS4-2H2O

C	-1.01573500	-0.36399100	0.81522500
O	-1.27432700	0.40735600	1.76233800
N	-1.30341400	-0.11830000	-0.49160000
H	-0.79397000	-0.72485800	-1.16082800
C	-1.94526000	1.10875000	-1.00294800
C	-0.81822200	2.16449100	-1.12443200
O	-0.11522300	2.12942500	-2.15657500
C	-0.35793100	-1.73643000	1.06791900
C	-0.46226200	-2.13746500	2.54518200
H	-0.79459200	-2.47941400	0.38539900
C	1.77817000	-1.20187100	2.10140900
C	0.73561900	-1.43097200	3.22533600
H	-0.37797700	-3.23317300	2.62715800
H	-1.42804800	-1.83717400	2.97444100
H	2.72336200	-1.74264500	2.25796500
H	2.01029700	-0.13357200	1.97604200
H	1.15065600	-2.03074300	4.04922800
H	0.41210600	-0.46230900	3.63179500
N	1.13788600	-1.75336000	0.86094200
C	-1.22705100	3.40257500	1.11652000
C	-0.33093500	4.50306000	1.71363500
H	-1.38120500	2.56865800	1.81781600
H	-2.20325800	3.79071700	0.78958600
C	1.09174400	4.04107100	1.35529900
H	-0.54372800	5.47311200	1.23268300
H	-0.48875200	4.61533000	2.79714300
H	1.84535600	4.84029900	1.40651800
H	1.41049700	3.21929100	2.02044400
N	-0.44026700	2.92638600	-0.05316700
C	1.96071600	2.46315500	-0.51274300
O	2.92381600	2.77187700	-1.22740600

N	1.76782200	1.19178400	-0.01218900
C	0.91958600	3.50837900	-0.08214500
H	0.99128200	4.32347800	-0.82343900
C	2.25564800	0.05882700	-0.80259000
C	3.78480600	-0.25551000	-0.75186800
S	3.47116400	-2.10521200	-0.61917000
C	4.53177400	0.31408900	0.45345100
H	5.56149200	-0.07951000	0.47899200
H	4.57979100	1.41250800	0.35757600
H	4.04668500	0.06925800	1.40780700
C	4.50817600	0.10230500	-2.05302600
H	3.99095700	-0.33068400	-2.92460500
H	4.52455600	1.19912900	-2.16106600
H	5.54421800	-0.27609600	-2.04180400
C	1.64828900	-1.36803700	-0.52044900
O	0.77901100	-1.76323200	-1.45198200
H	1.37799700	-3.08813800	0.88587600
C	-3.26477300	1.44642100	-0.27402400
H	-3.07563100	1.65851400	0.78480800
H	-3.67379500	2.35776000	-0.74429500
C	-4.24730800	0.29971500	-0.39218800
C	-4.37435100	-0.63788200	0.65162000
C	-5.00847900	0.11804000	-1.56361700
C	-5.24252000	-1.73363800	0.52844600
H	-3.78307800	-0.50158400	1.56330000
C	-5.87845800	-0.97580500	-1.68995900
H	-4.91980700	0.84293900	-2.38113700
C	-5.99662400	-1.90636900	-0.64380400
H	-5.33051800	-2.45291200	1.34914200
H	-6.46728000	-1.10040900	-2.60443700
H	-6.67556800	-2.75937100	-0.74103400
H	-2.19634400	0.88144300	-2.05081100
H	1.98131100	0.24482000	-1.85666400
H	0.87211900	1.05953200	0.46451900
O	0.70405900	-4.21895300	-1.52421300
H	0.69290500	-3.09323500	-1.55116800
H	-0.22562200	-4.51283700	-1.57825100
O	1.55745900	-4.24538600	0.74287500
H	1.00143300	-4.73509400	1.37897300
H	1.10325600	-4.37134800	-0.36063900

2.4. The method of Bioactivity Experiment

2.4.1 Tissue Culture and Treatment

35 mm tissue culture dishes (Corning) were prepared by seeding them with 03.x10^6 SK-N-SH cells (ATCC). Dishes were incubated at 37°C and 5% CO_2 for 24-48 h in Dulbecco's Modified Eagle Medium (DMEM, Gibco) containing 4.5 g/L D-glucose, 110 mg/L sodium pyruvate, and supplemented with 10% fetal bovine serum, FBS (Gibco), to ~85% confluence (~1x10^6 cells/dish). Fresh media containing 0.5 mM IBMX was added, and the cells incubated for another 30 min. Dishes were washed with 1X PBS (Gibco) and replaced with fresh media containing 0.5 mM Bestatin and 10 µM Forskolin. Dishes were then treated in triplicate with concentrations of TCP ranging from 80 to 2.44 x 10^{-5} µM, which were incubated for 5 min, washed with 1X PBS, and immediately frozen at -80°C for 2 h.

2.4.2 ELISA

Cells were harvested by adding 1 mL of 1X ELISA buffer (Cayman), 100 mM phosphate, 0.1% BSA, 400 mM NaCl, 1 mM EDTA, and 0.01% sodium azide, to the frozen dishes and collecting the lysate. The solution was centrifuged in an Eppendorf 5415D at 16110x***g*** for 2 h and the supernatant collected. ELISA samples for each treatment were immediately prepared according to the colorimetric kit specifications (Cayman, 581001). Samples were loaded onto the 96-well plate provided with the kit, incubated at 4°C for 18 h, wells washed with 1X wash buffer (Cayman), developed at room temperature, and read with a Synergy HT plate reader (BioTek) at time intervals from 15 to 60 min. Absorbance statistics were processed in GraphPad Prism.

3.Supplementary Notes

3.1 Selected NMR Spectrums

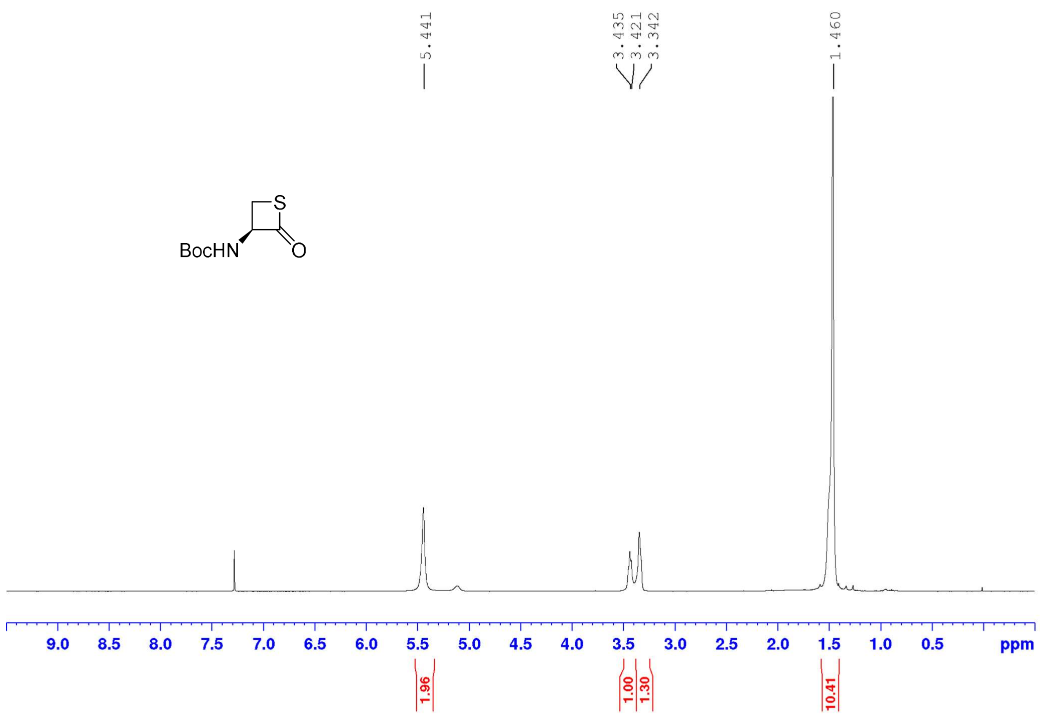

^{1}H NMR spectrum of compound **S7a** ($CDCl_3$, 500 MHz)

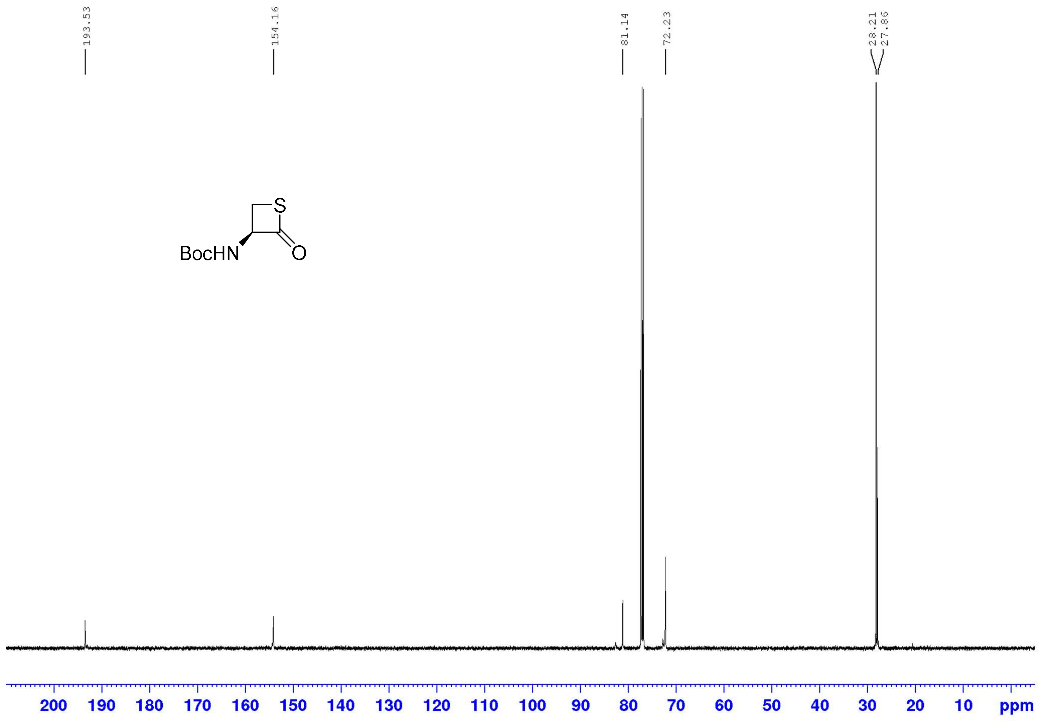

^{13}C NMR spectrum of compound **S7a** ($CDCl_3$, 126 MHz)

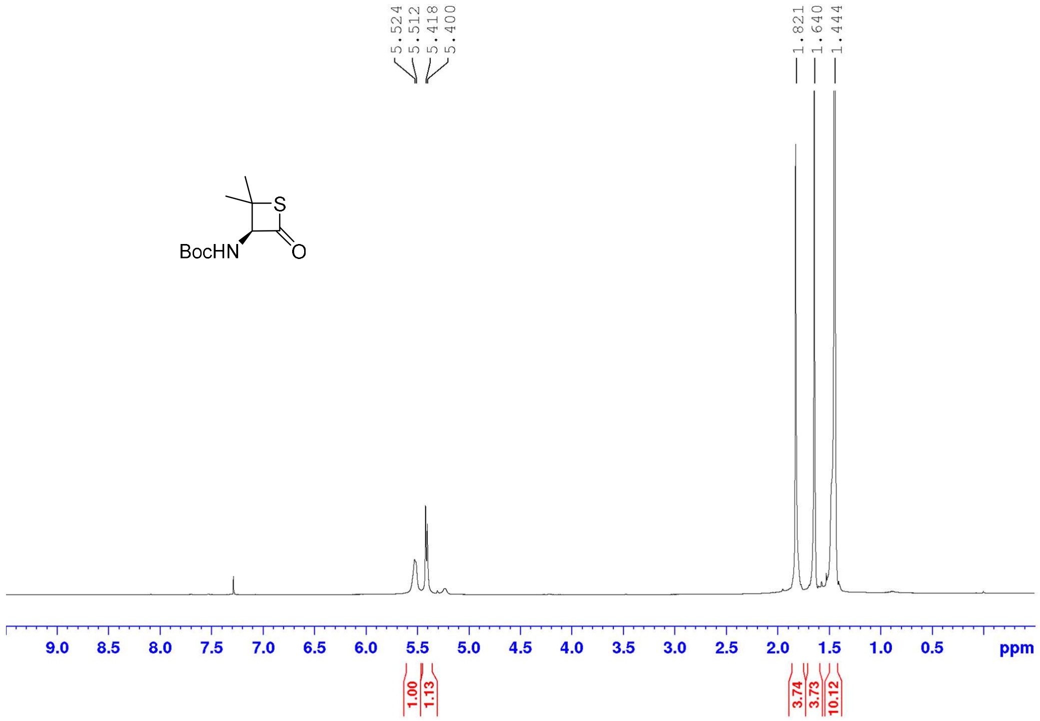

^1H NMR spectrum of compound **S7b** ($CDCl_3$, 500 MHz)

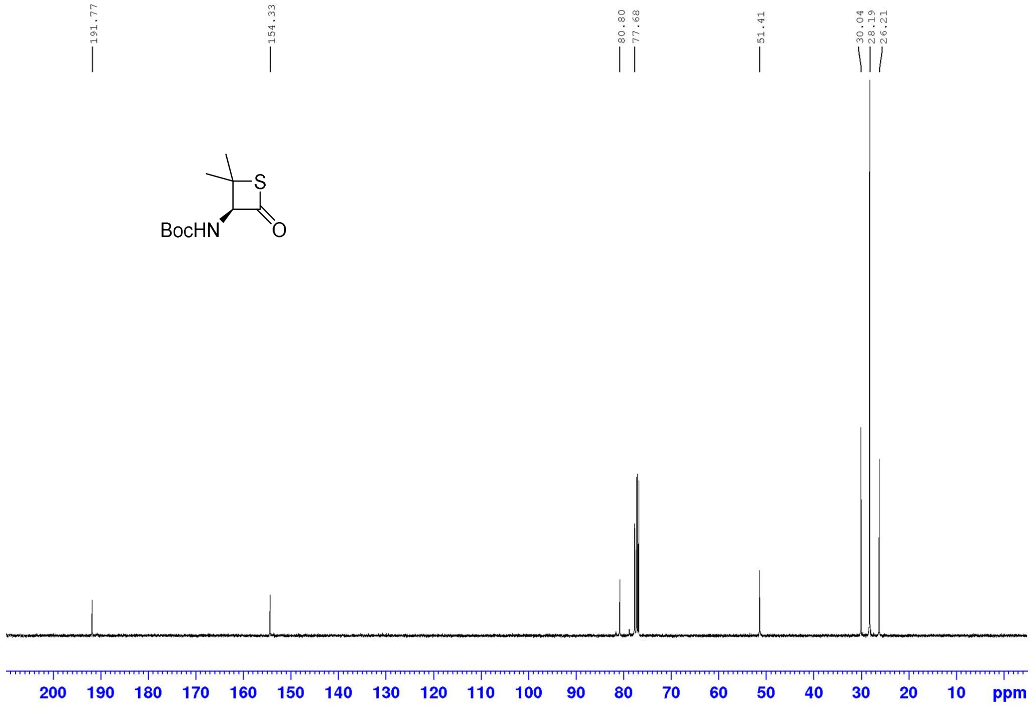

^{13}C NMR spectrum of compound **S7b** ($CDCl_3$, 126 MHz)

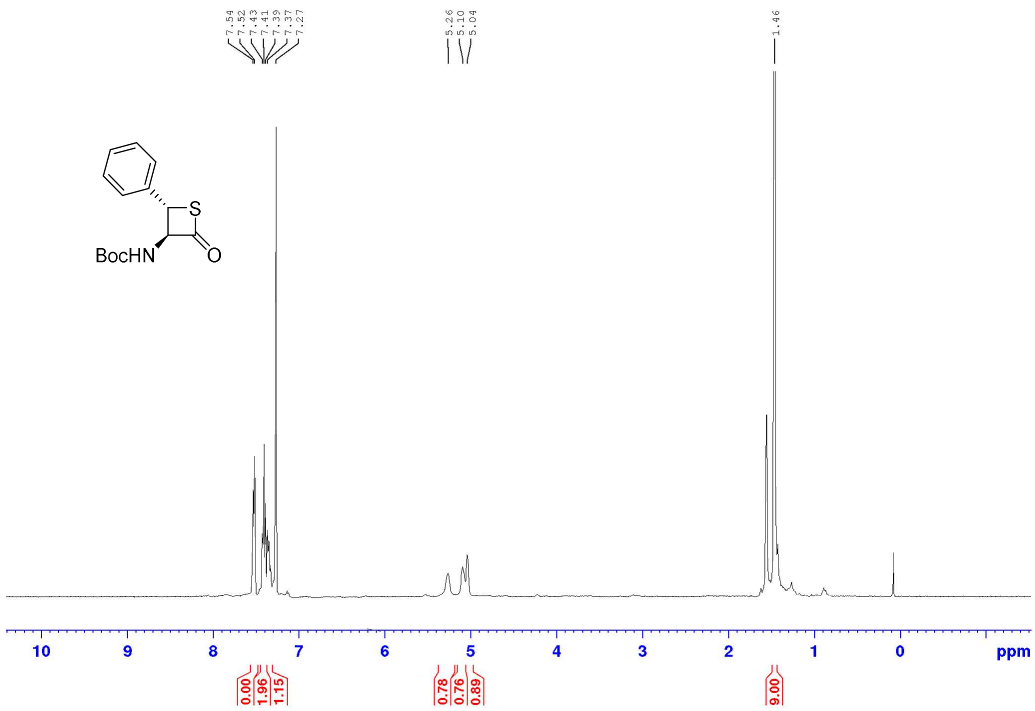

^{1}H NMR spectrum of compound **S7c** ($CDCl_3$, 400 MHz)

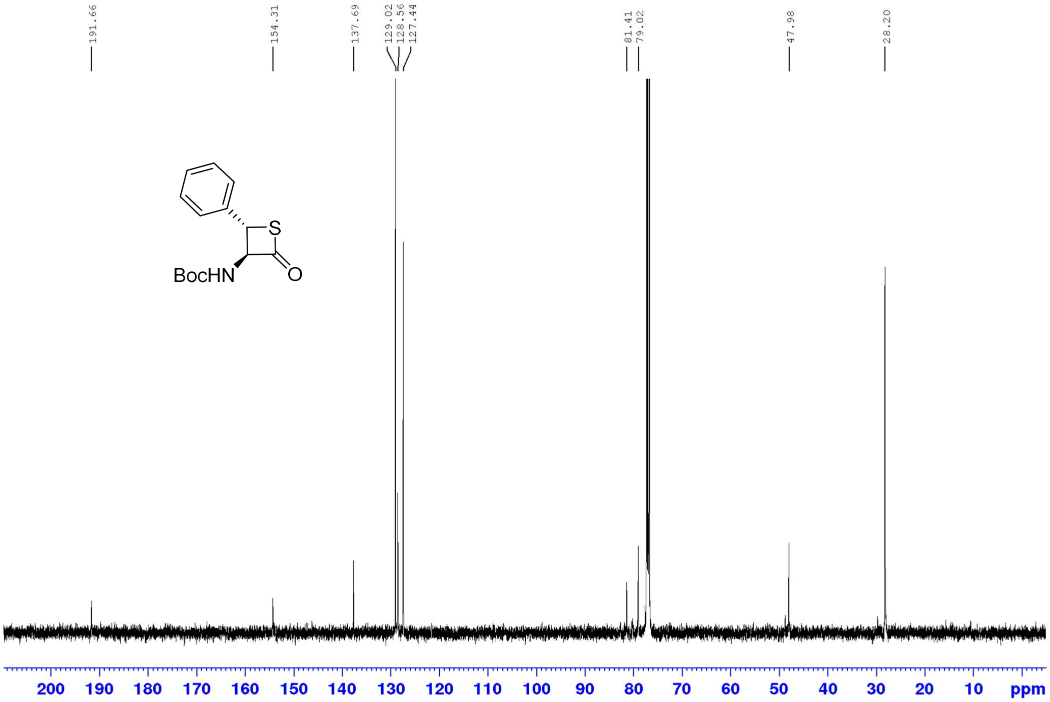

^{13}C NMR spectrum of compound **S7c** ($CDCl_3$, 126 MHz)

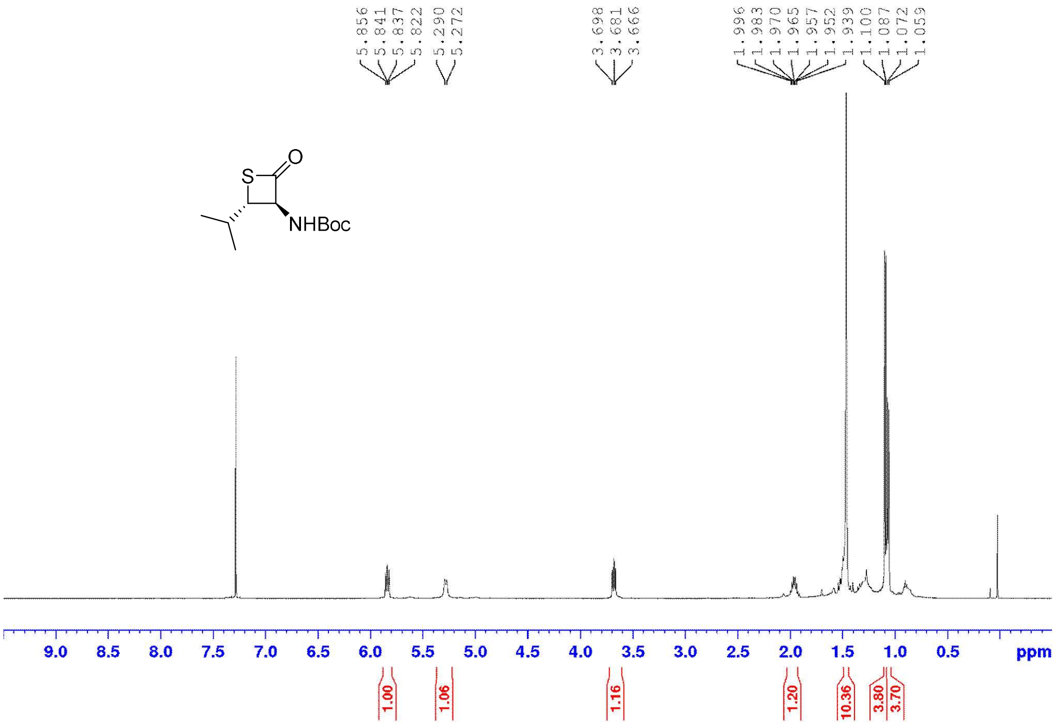

^{1}H NMR spectrum of compound **S7d** ($CDCl_3$, 500 MHz)

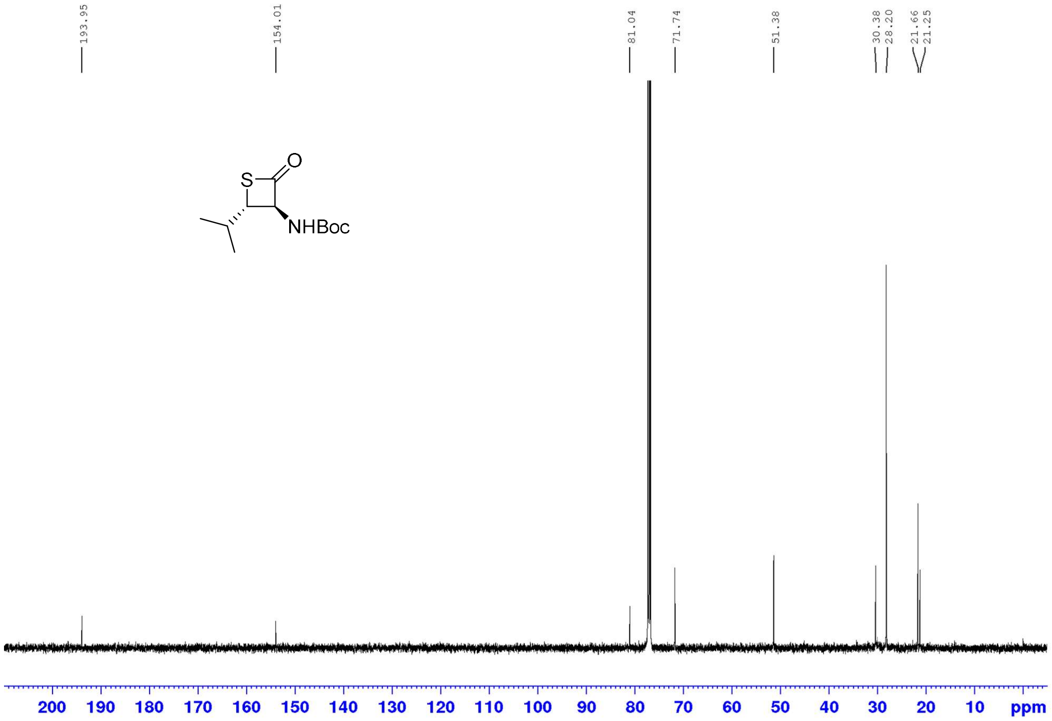

^{13}C NMR spectrum of compound **S7d** ($CDCl_3$, 126 MHz)

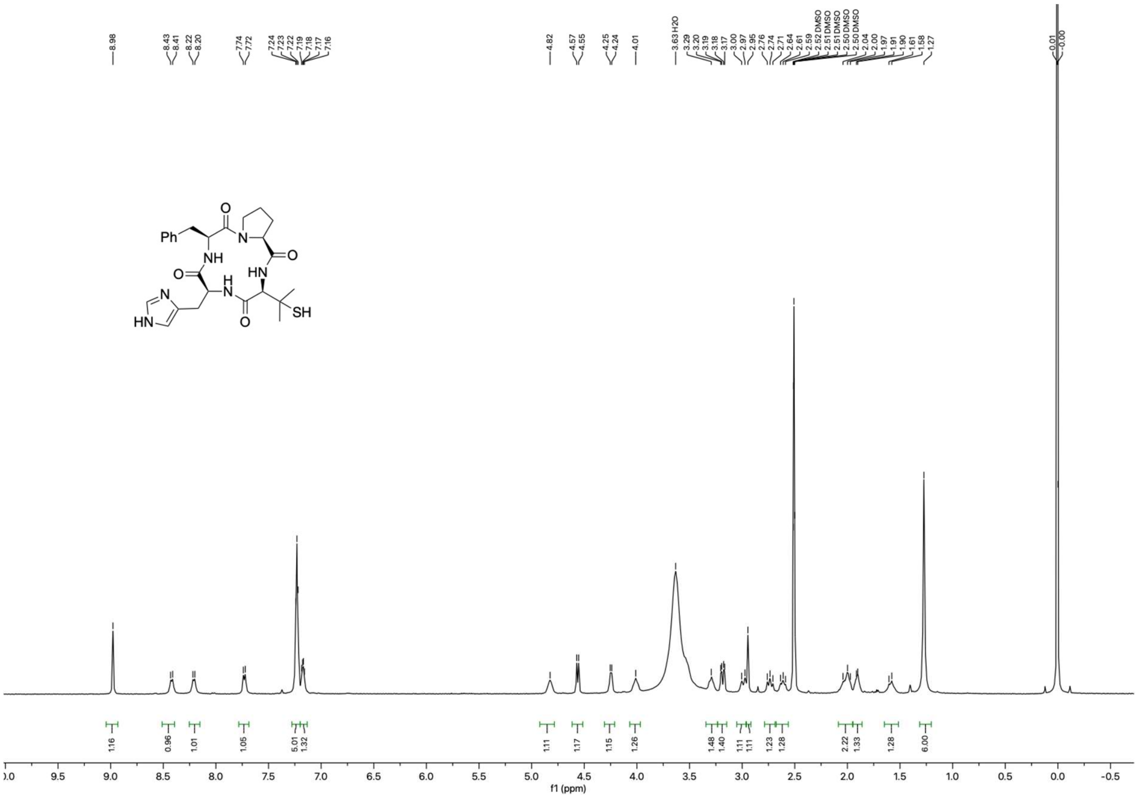

^{1}H NMR spectrum of compound **9e** (DMSO-d6, 500 MHz)

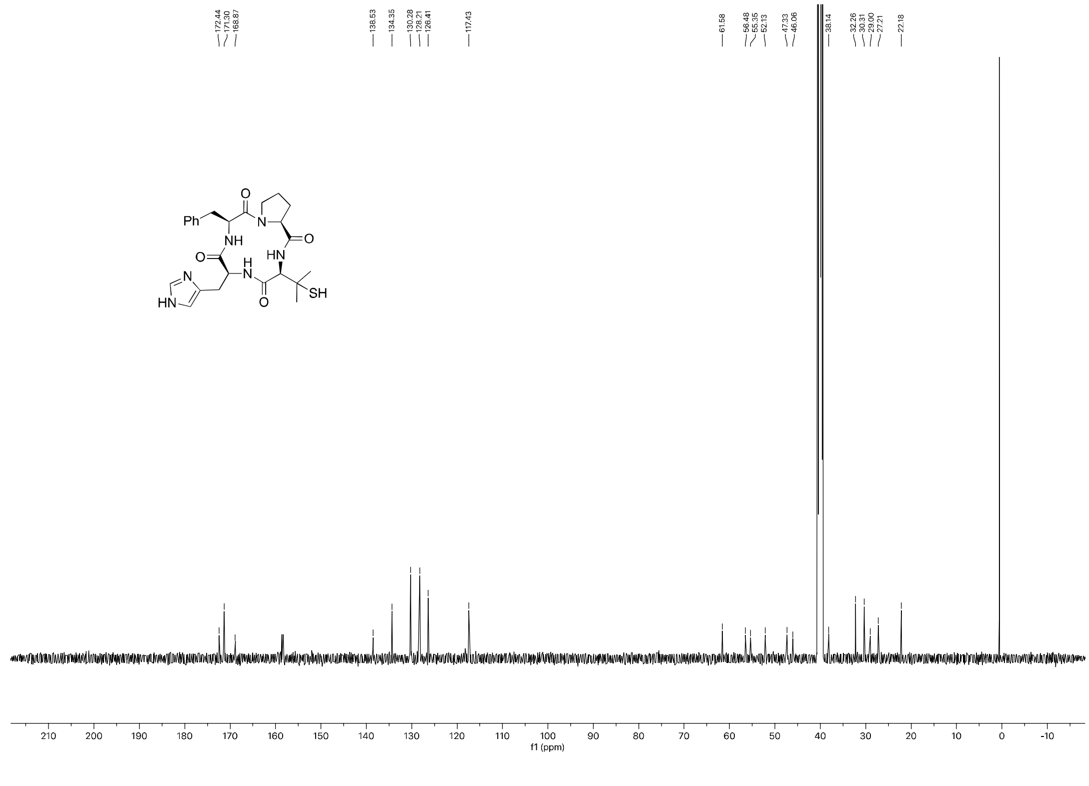

^{13}C NMR spectrum of compound **9e** (DMSO-*d6*, 126 MHz)

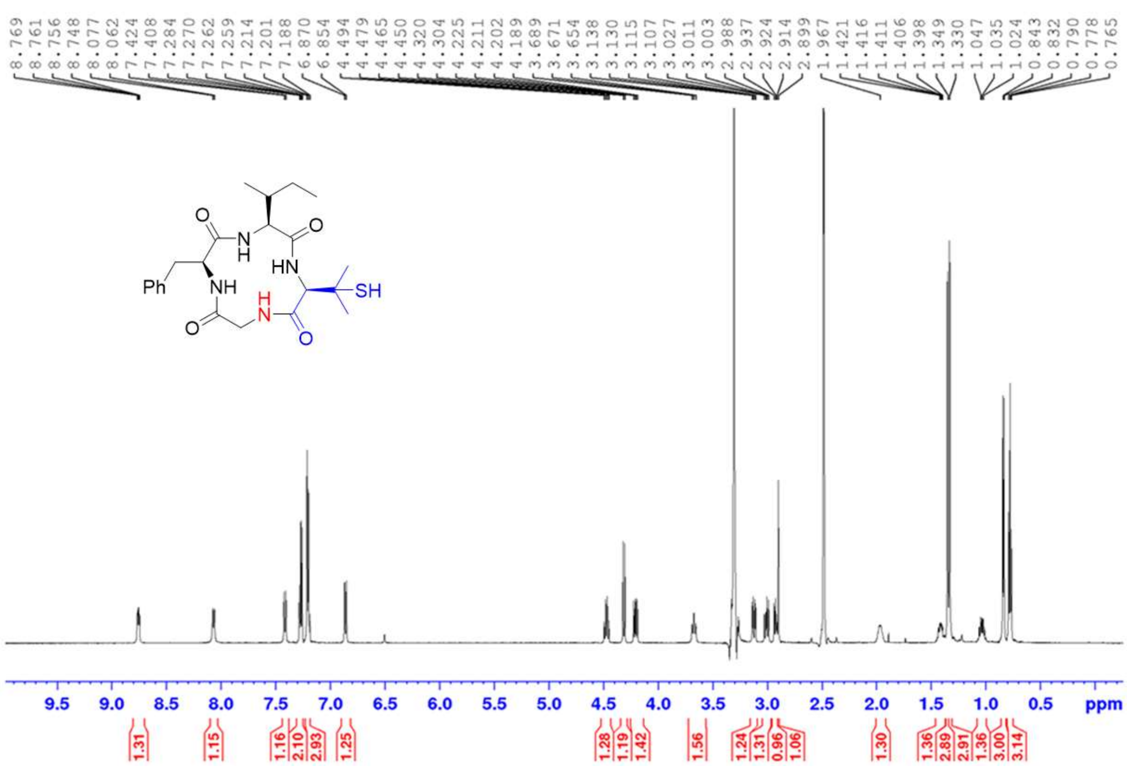

^{1}H NMR spectrum of compound **9f** (DMSO-d6, 600 MHz)

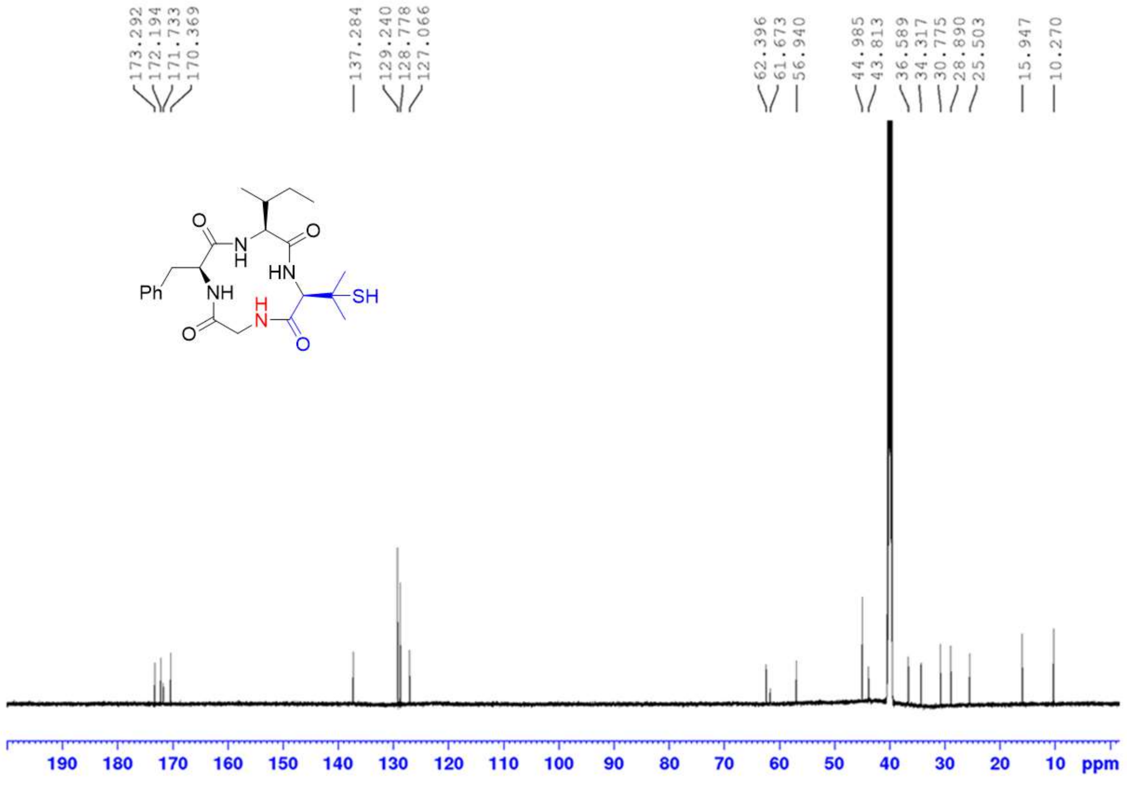

^{13}C NMR spectrum of compound **9f** (DMSO-*d6*, 151 MHz)

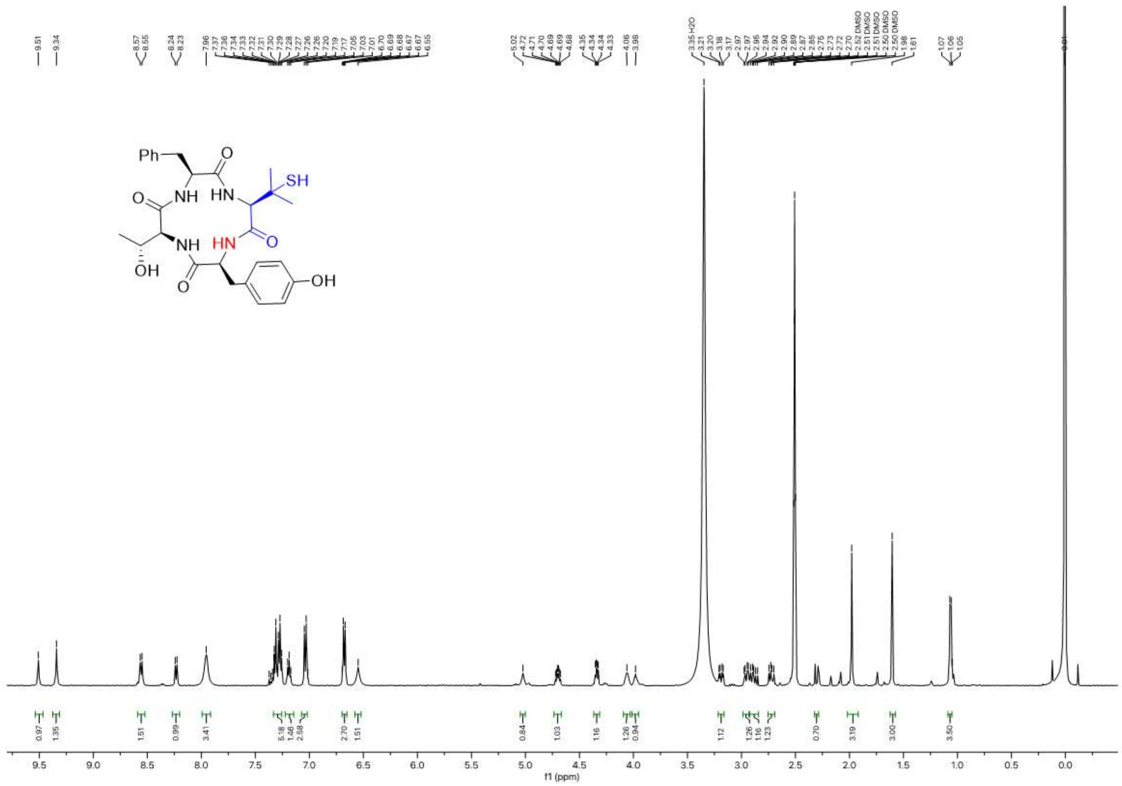

^{1}H NMR spectrum of compound **9h** (DMSO-d6, 500 MHz)

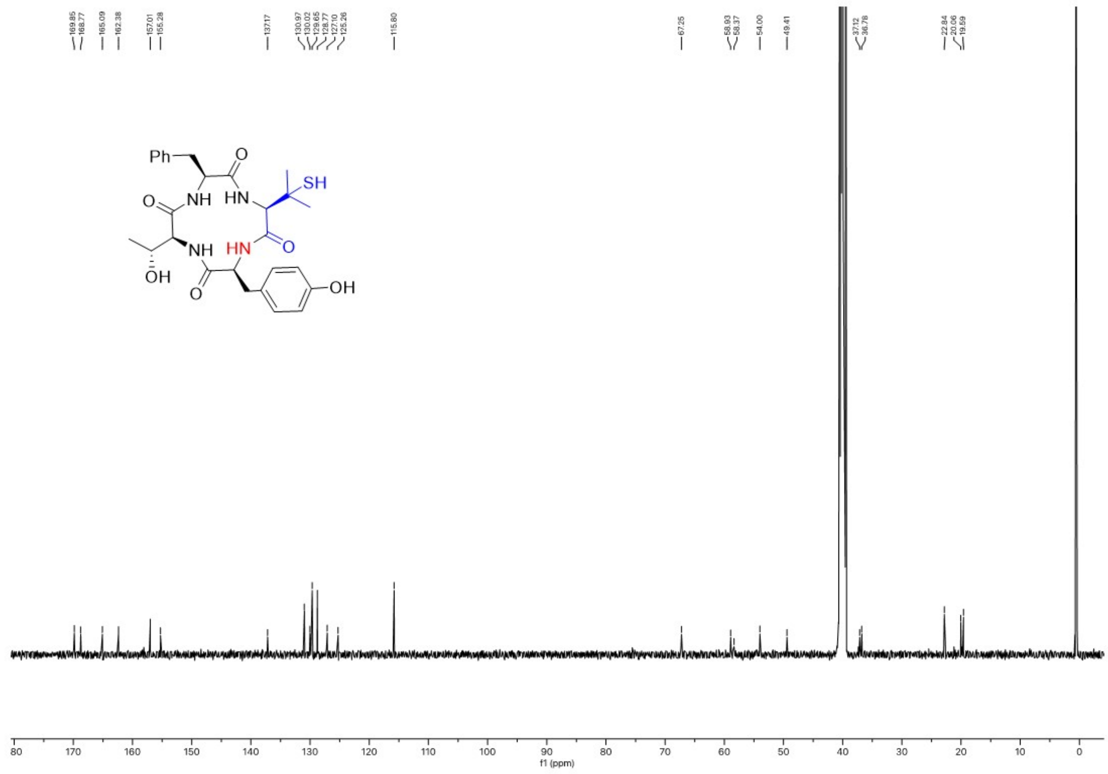

^{13}C NMR spectrum of compound **9h** (DMSO-*d6*, 126 MHz)

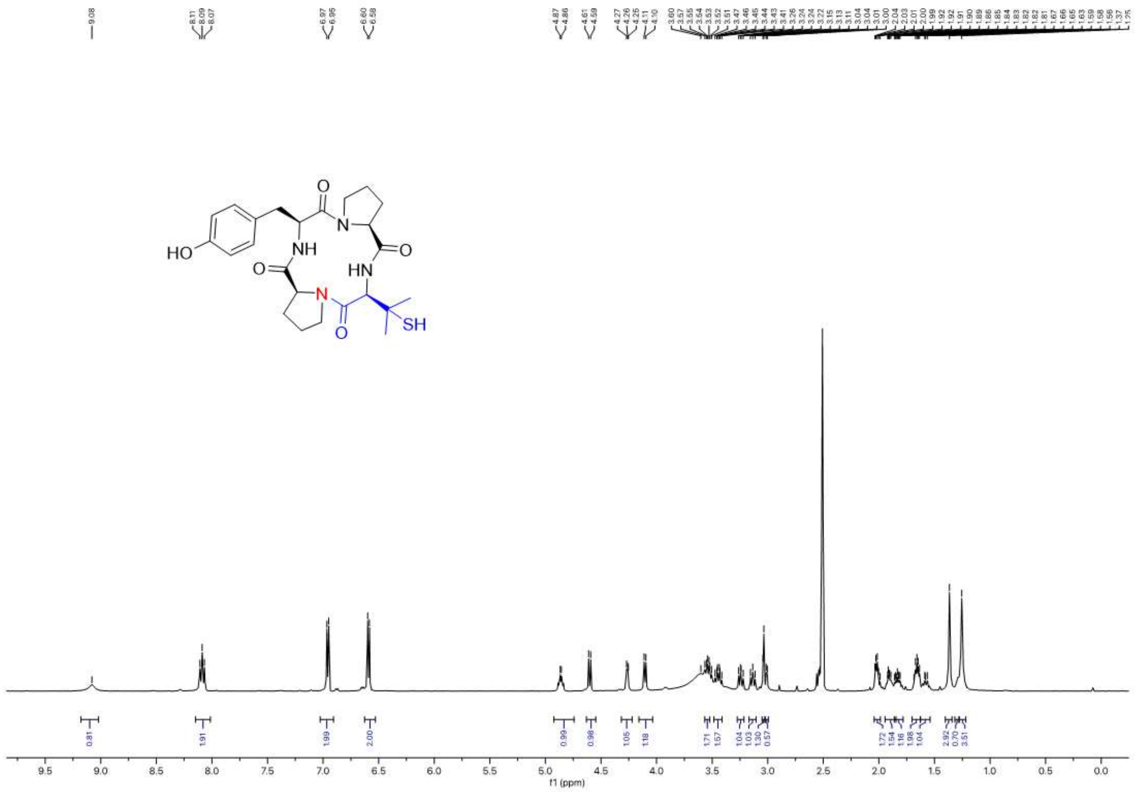

^{1}H NMR spectrum of compound **14a** (DMSO-*d6*, 500 MHz)

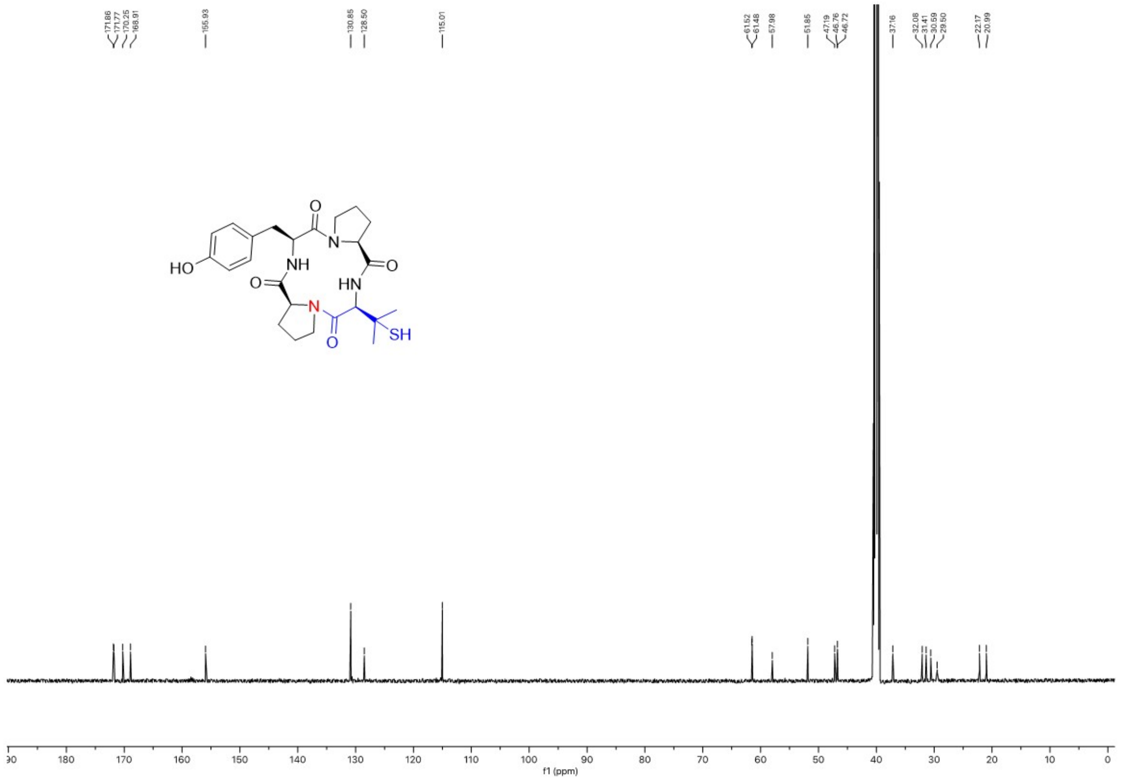

^{13}C NMR spectrum of compound **14a** (DMSO-*d6*, 126 MHz)

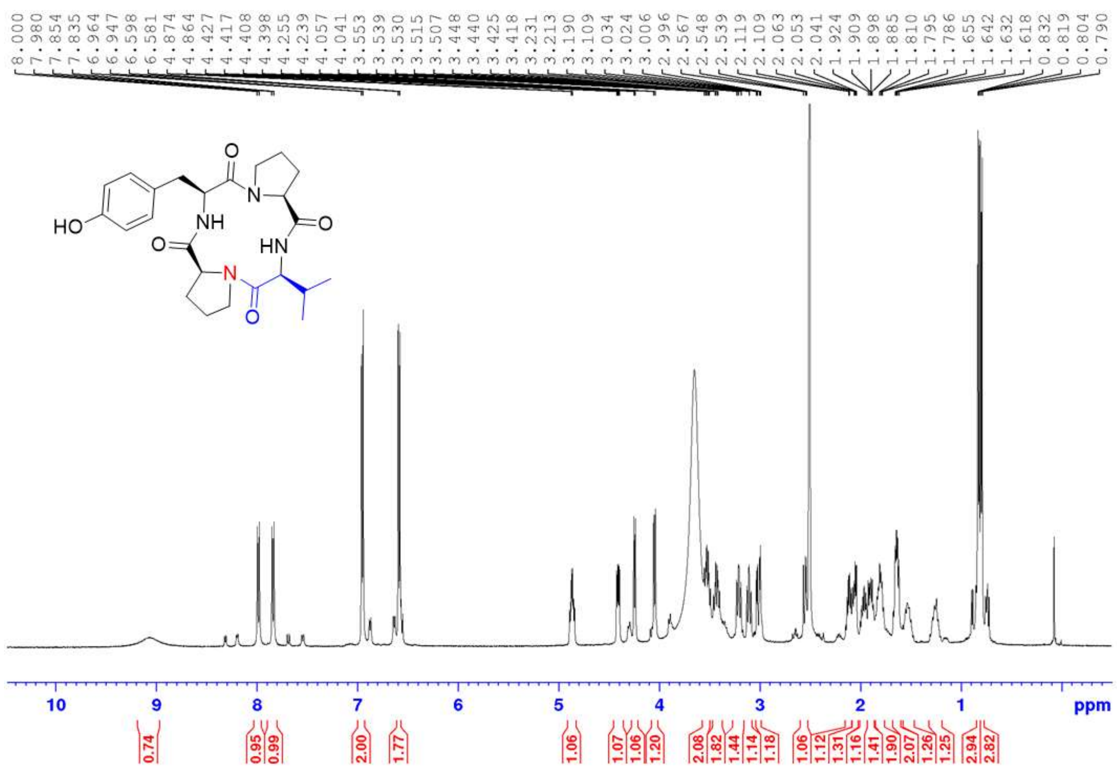

^{1}H NMR spectrum of compound 1 (DMSO-d6, 500 MHz)

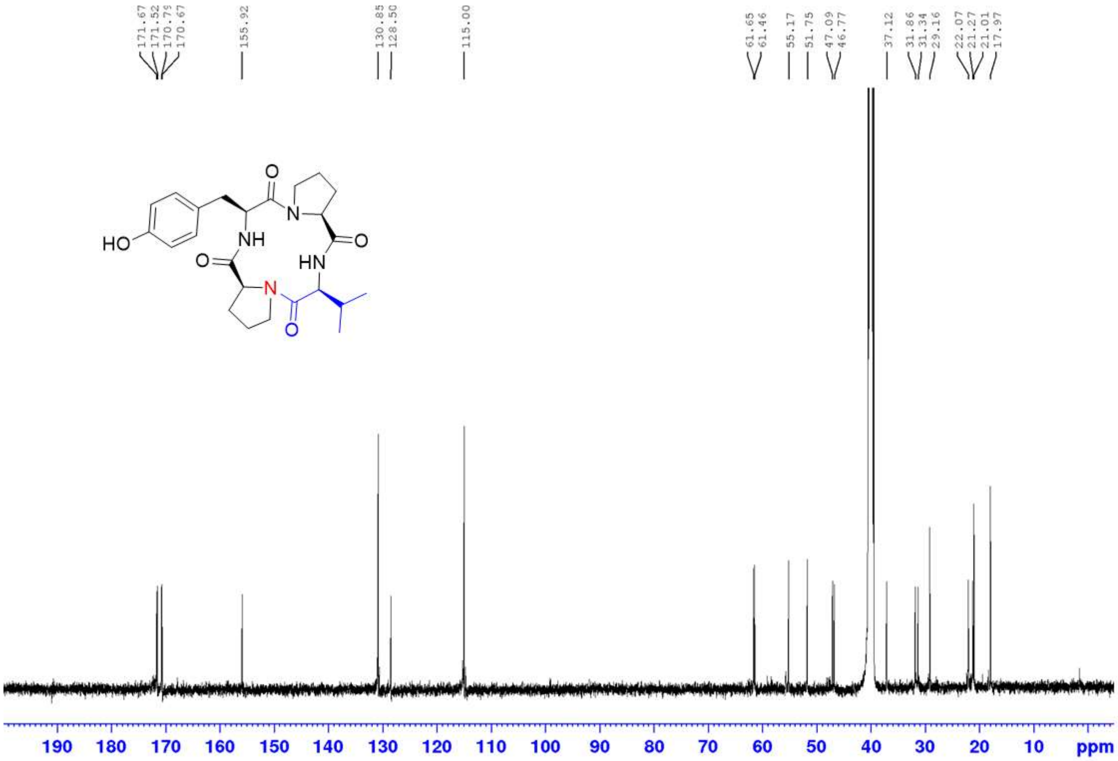

^{13}C NMR spectrum of compound 1 (DMSO-*d6*, 126 MHz)

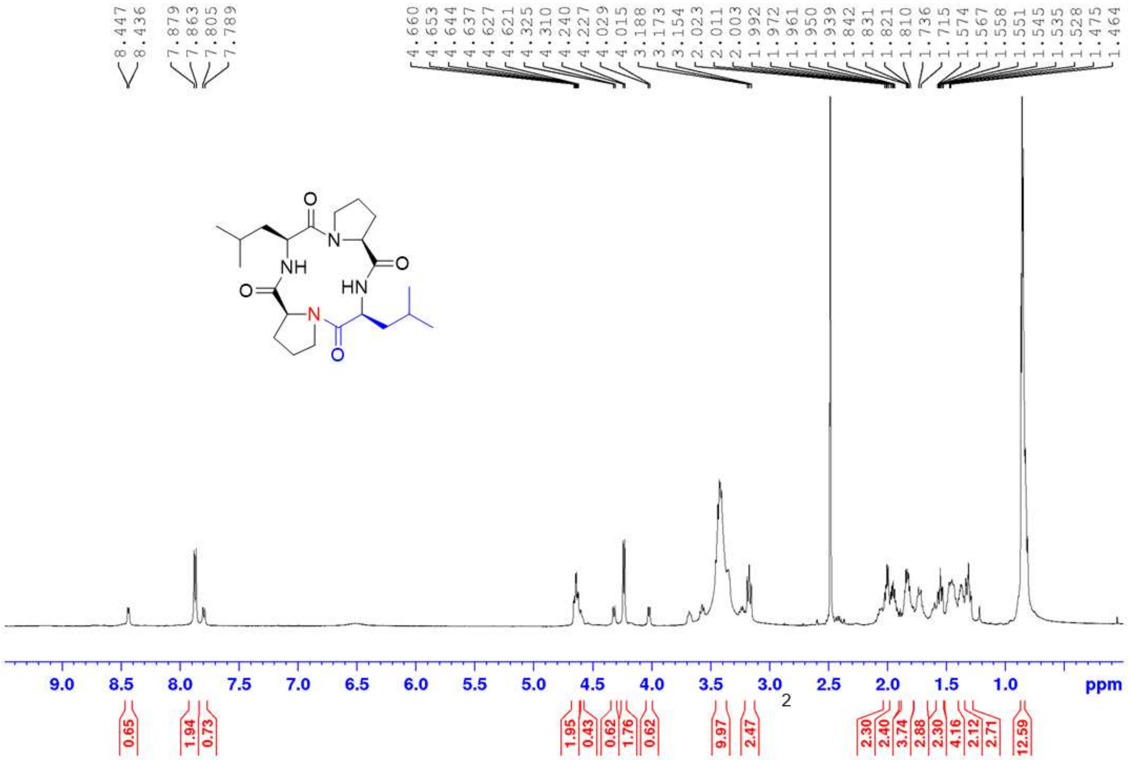

^{1}H NMR spectrum of compound **2** (DMSO-d6, 600 MHz, ratio = 5: 3)

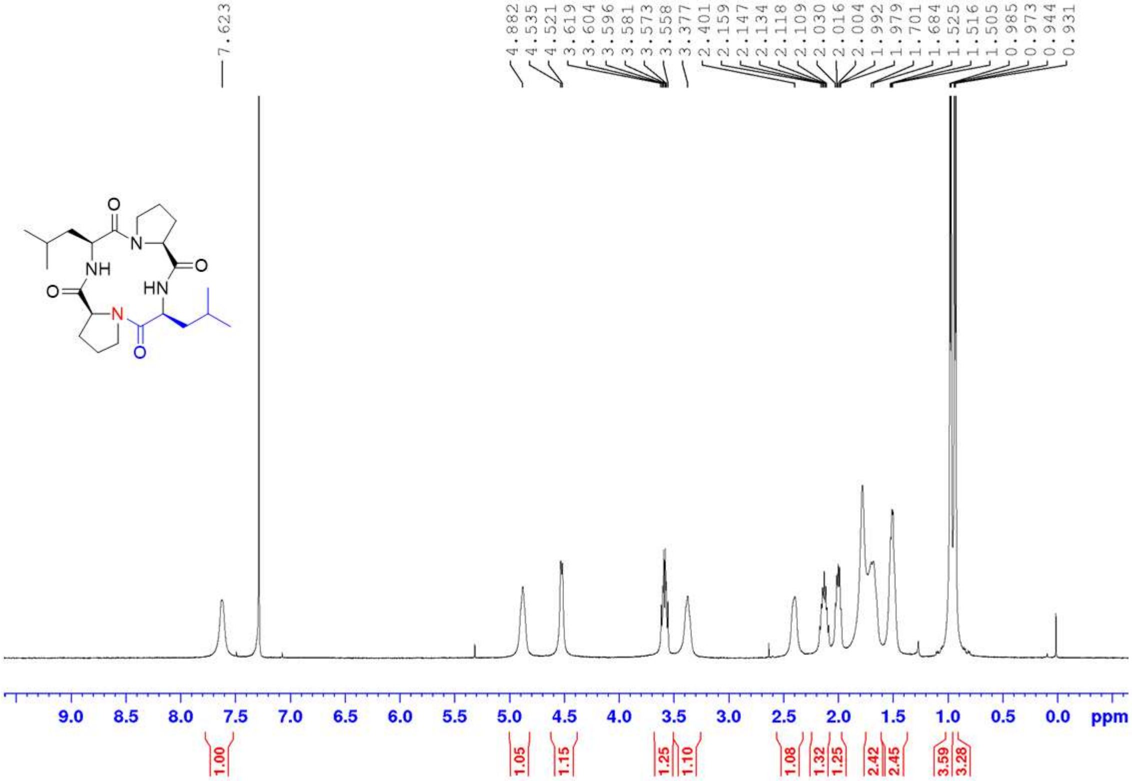

^{1}H NMR spectrum of compound **2** ($CDCl_3$, 500 MHz, signal conformer)

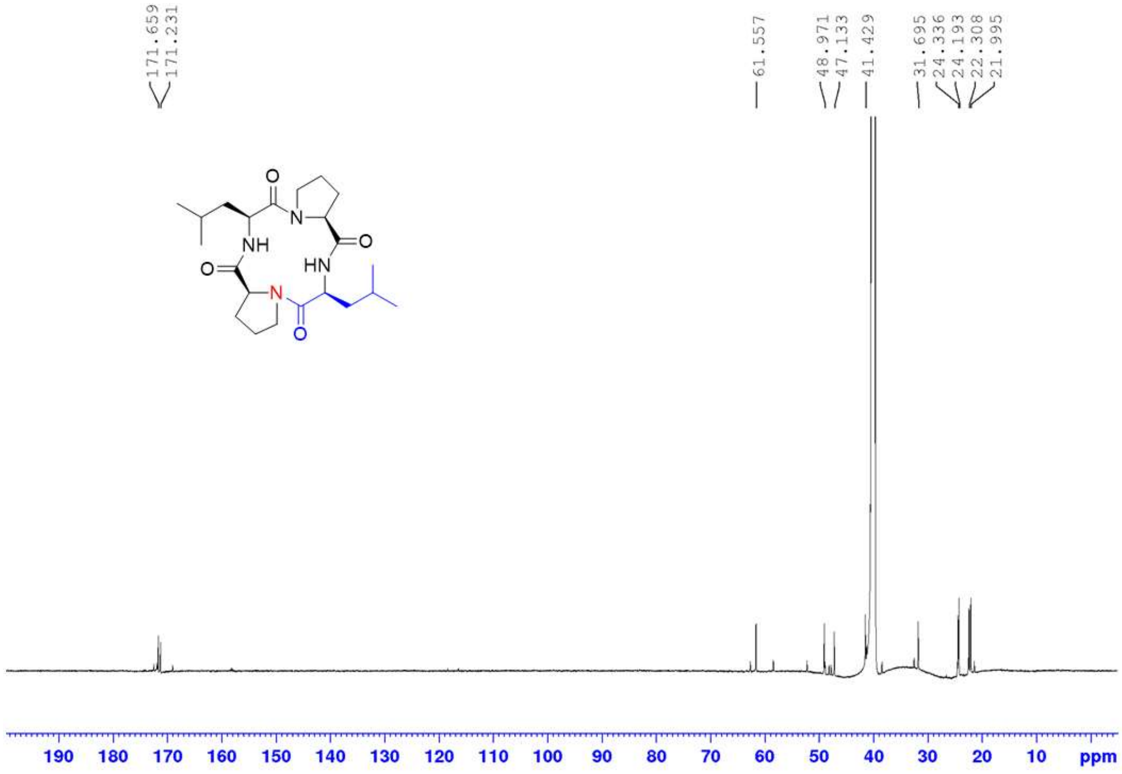

^{13}C NMR spectrum of compound **2** (DMSO-*d6*, 151 MHz)

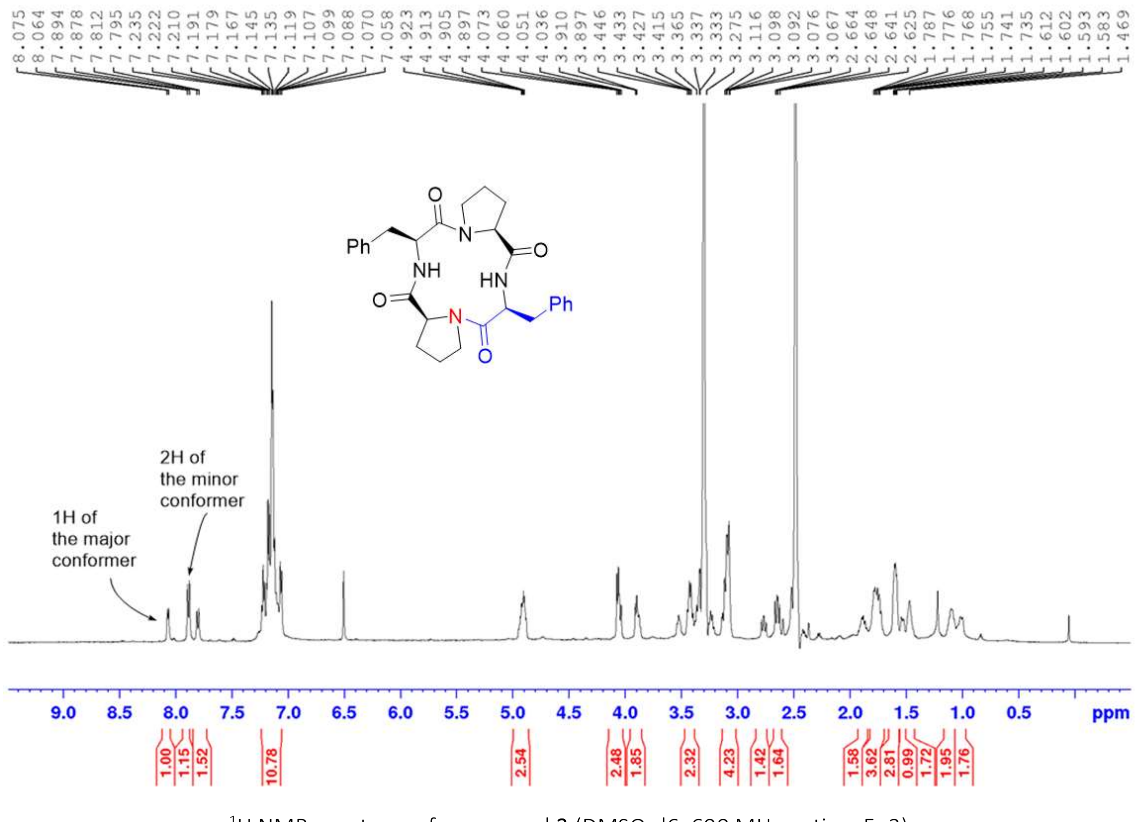

^{1}H NMR spectrum of compound **3** (DMSO-d6, 600 MHz, ratio = 5: 3)

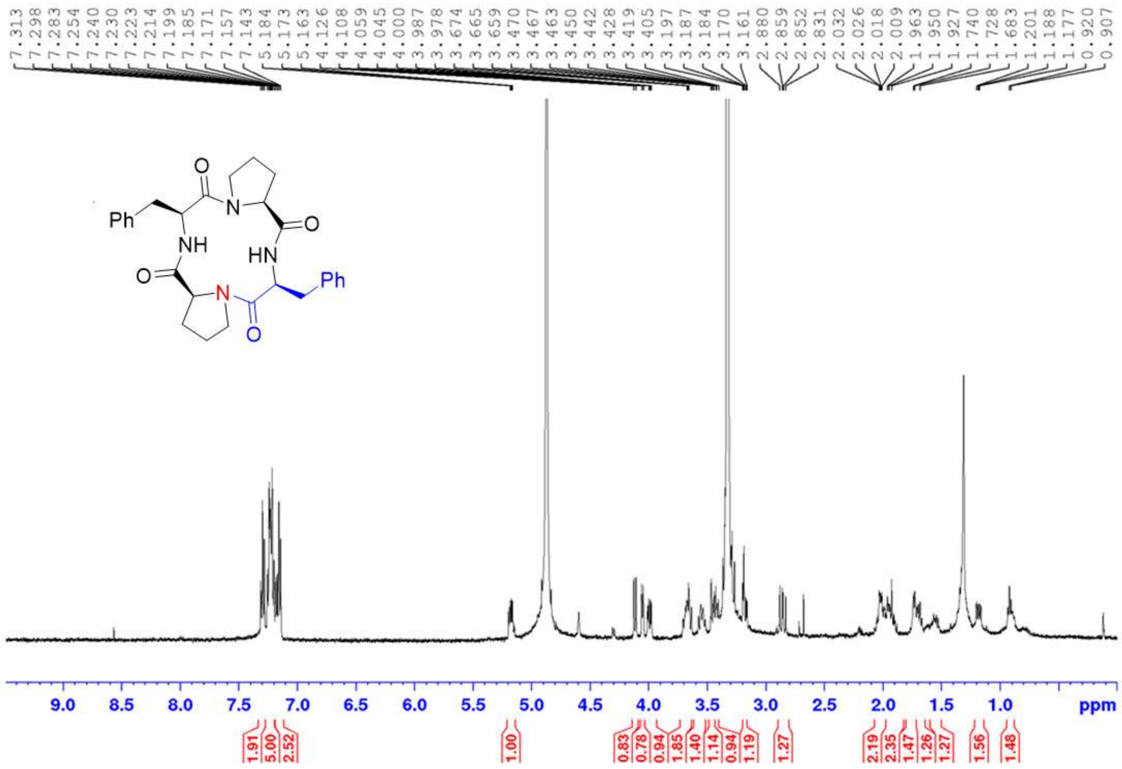

^{1}H NMR spectrum of compound **3** (MeOH-*d4*, 500 MHz, signal conformer)

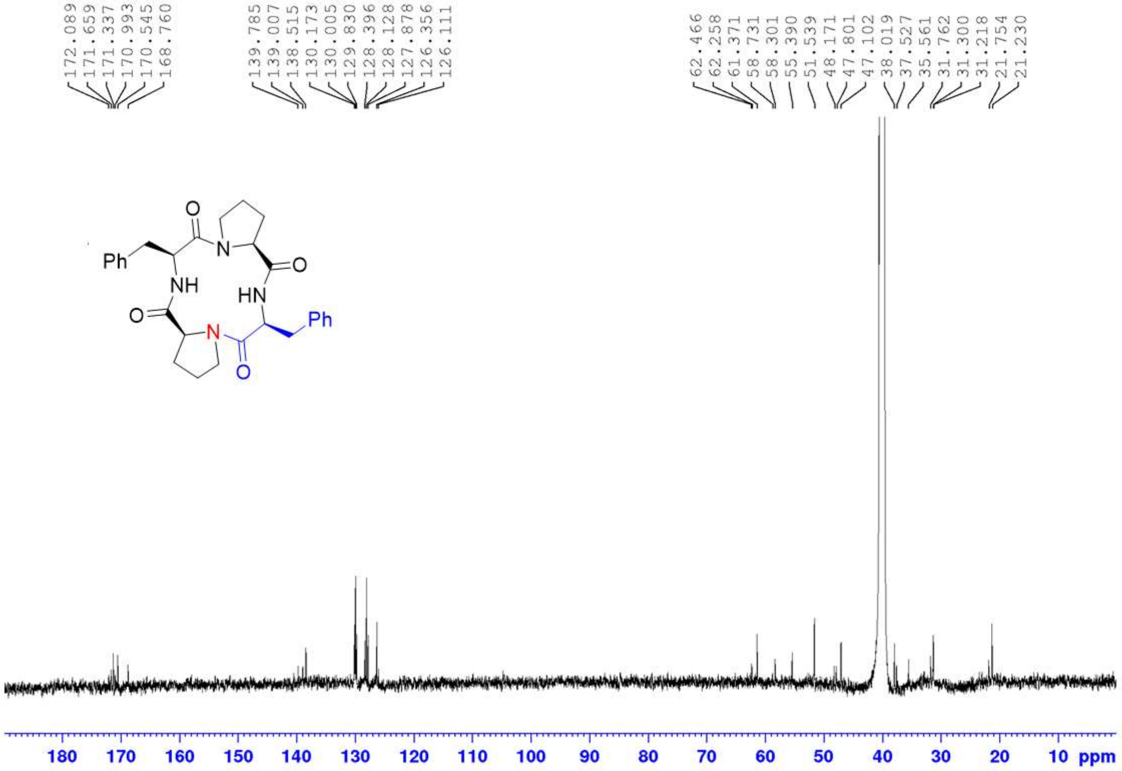

^{13}C NMR spectrum of compound **3** (DMSO-*d6*, 151 MHz)

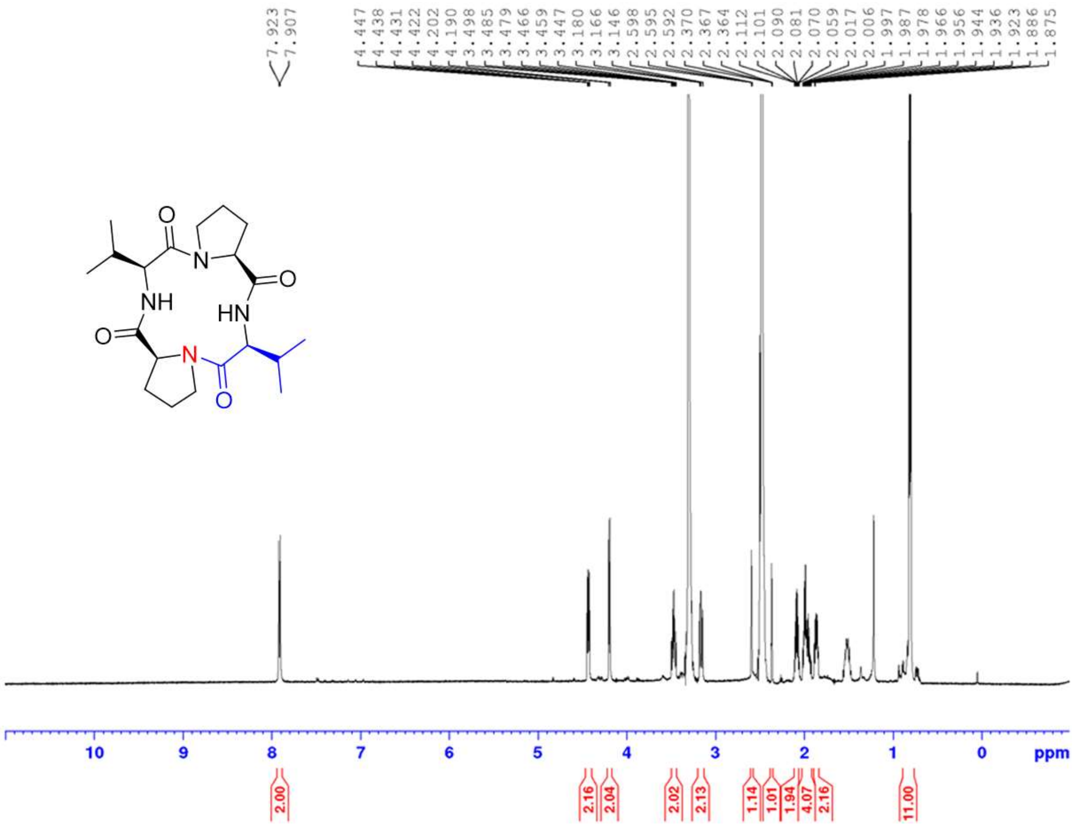

^{1}H NMR spectrum of compound **4** (DMSO-d6, 600 MHz)

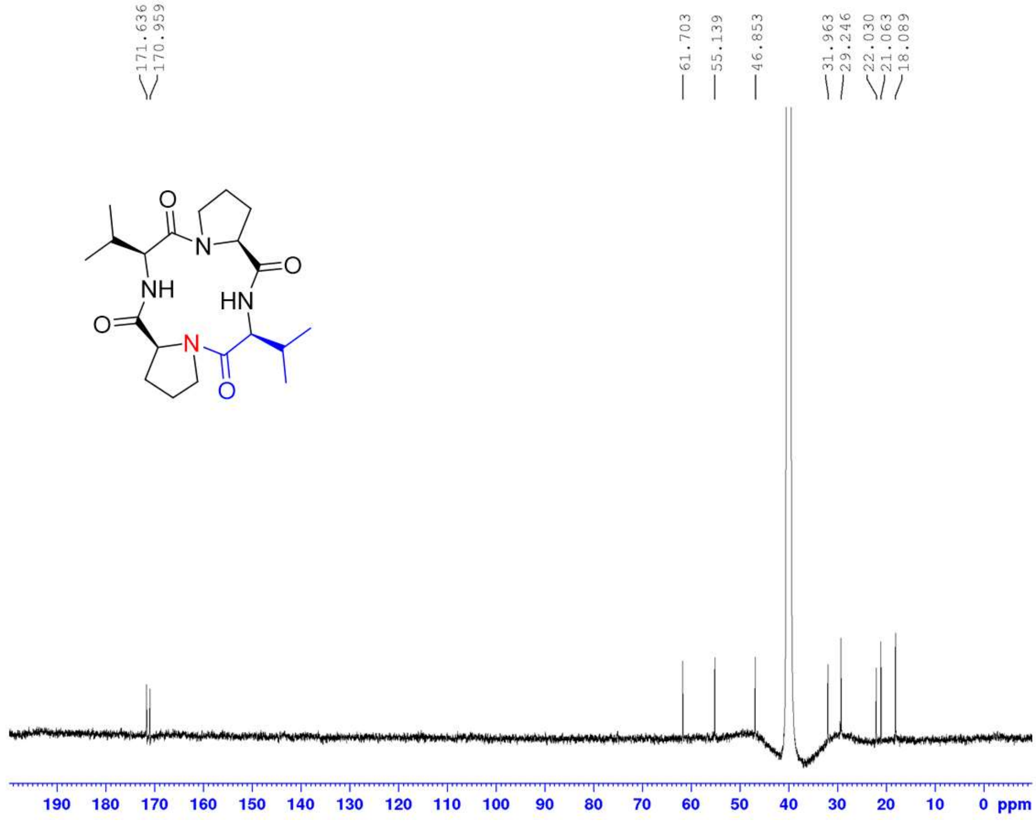

^{13}C NMR spectrum of compound **4** (DMSO-*d6*, 151 MHz)

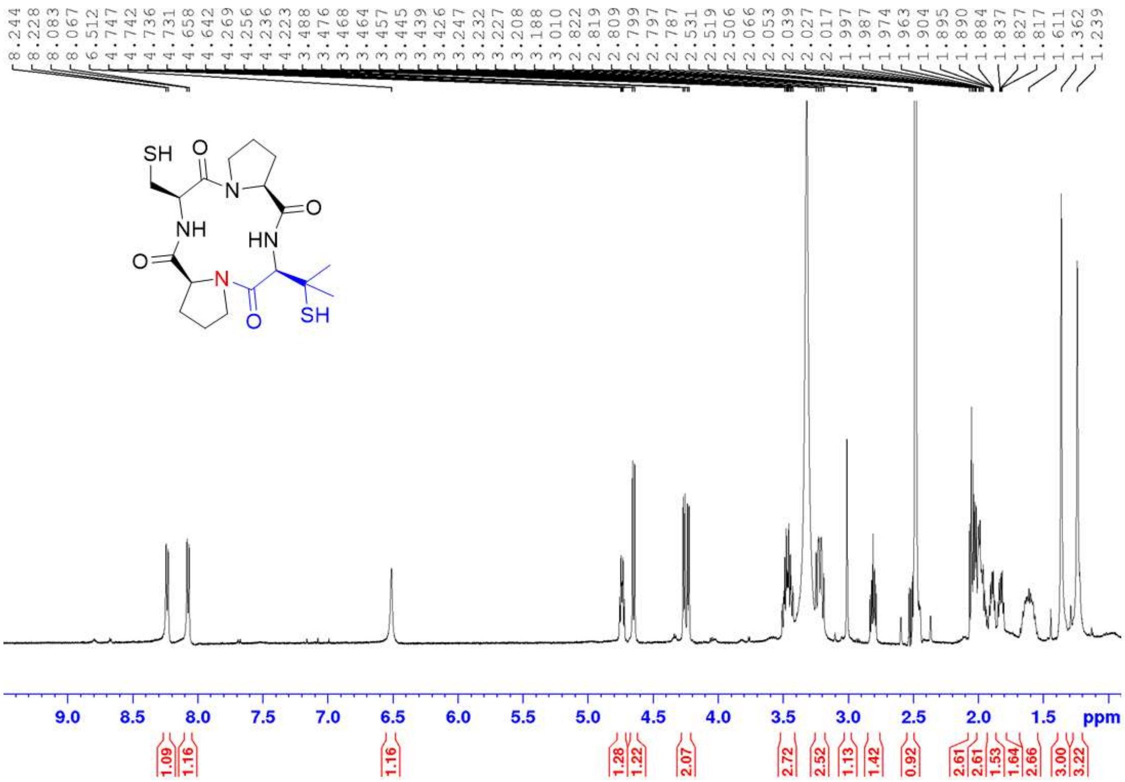

^{1}H NMR spectrum of compound **14c** (DMSO-*d6*, 600 MHz)

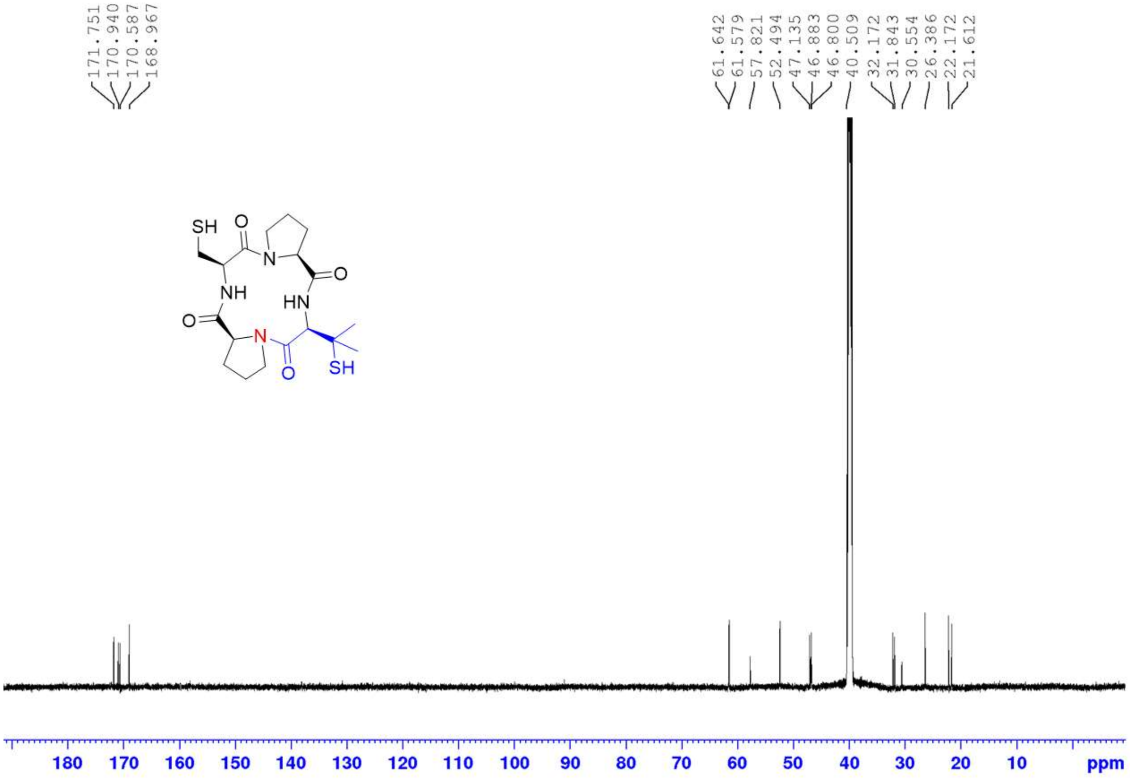

^{13}C NMR spectrum of compound **14c** (DMSO-*d6*, 151 MHz)

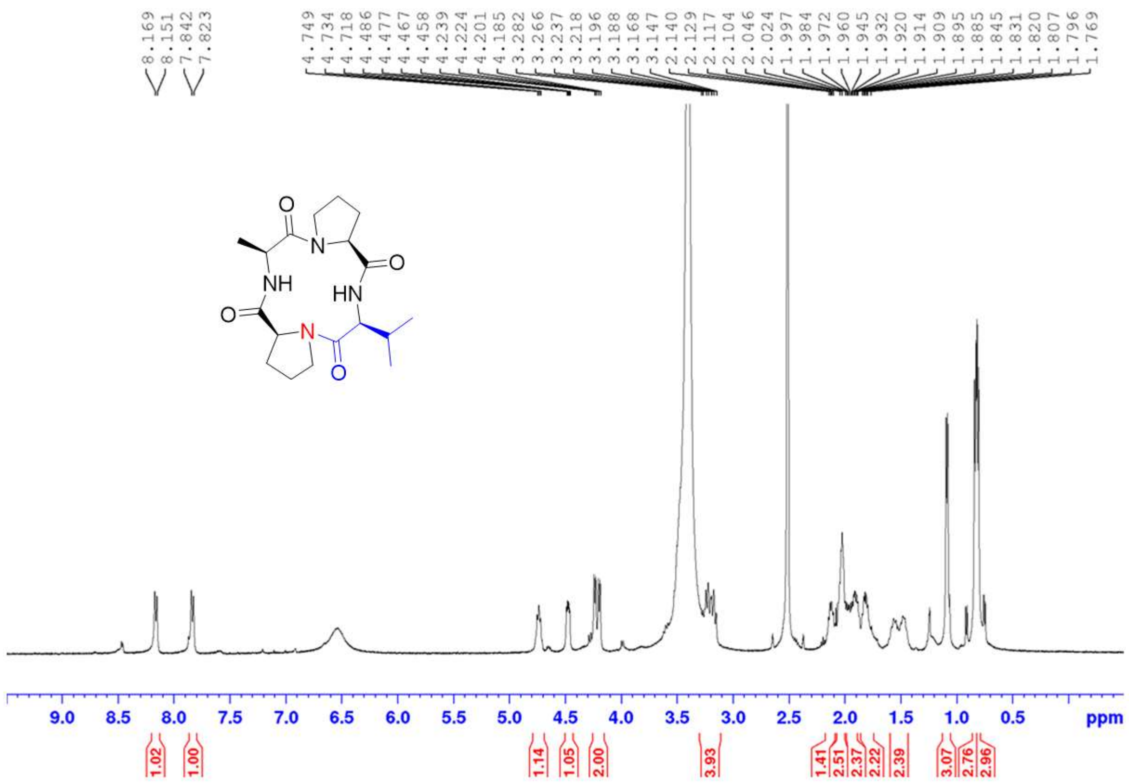

^{1}H NMR spectrum of compound **15c** (DMSO-*d6*, 500 MHz)

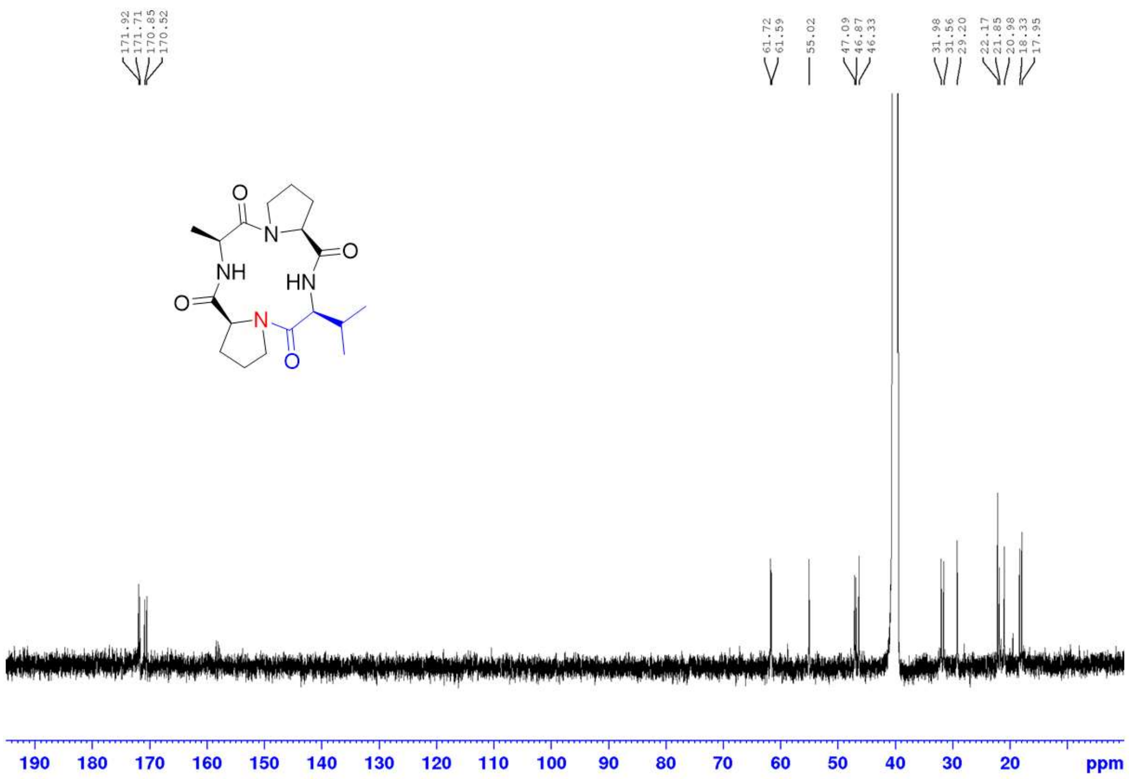

^{13}C NMR spectrum of compound **15c** (DMSO-*d6*, 126 MHz)

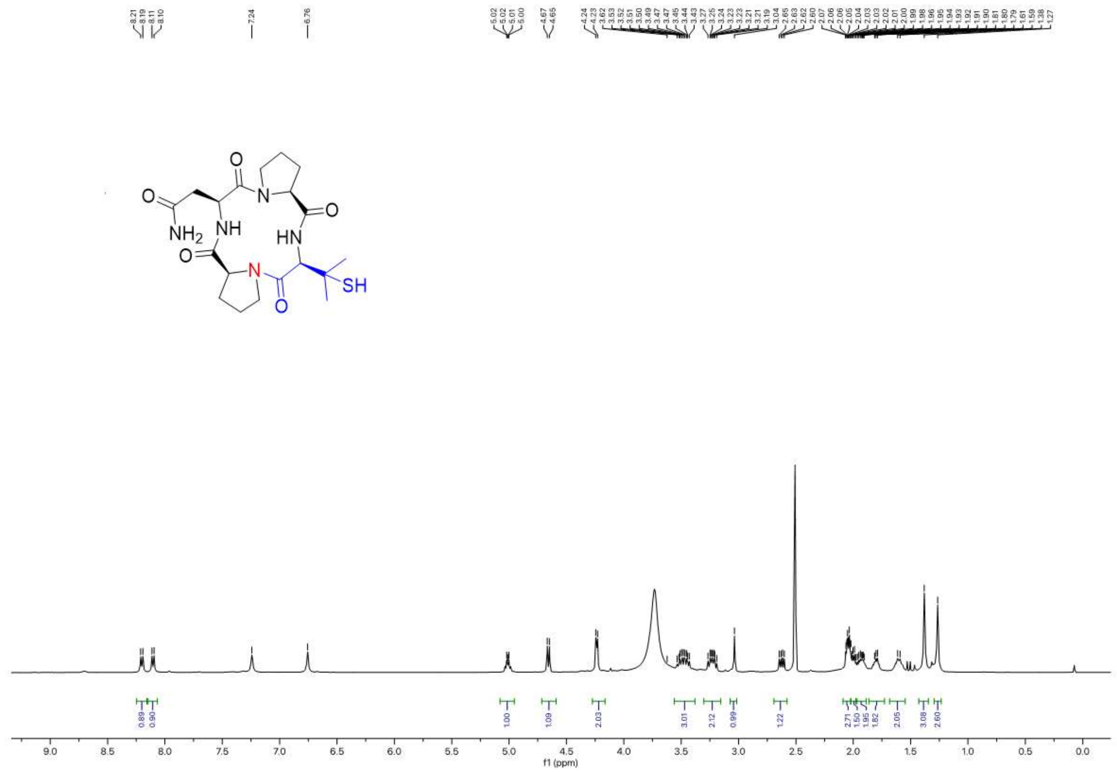

^{1}H NMR spectrum of compound **14d** (DMSO-*d6*, 600 MHz)

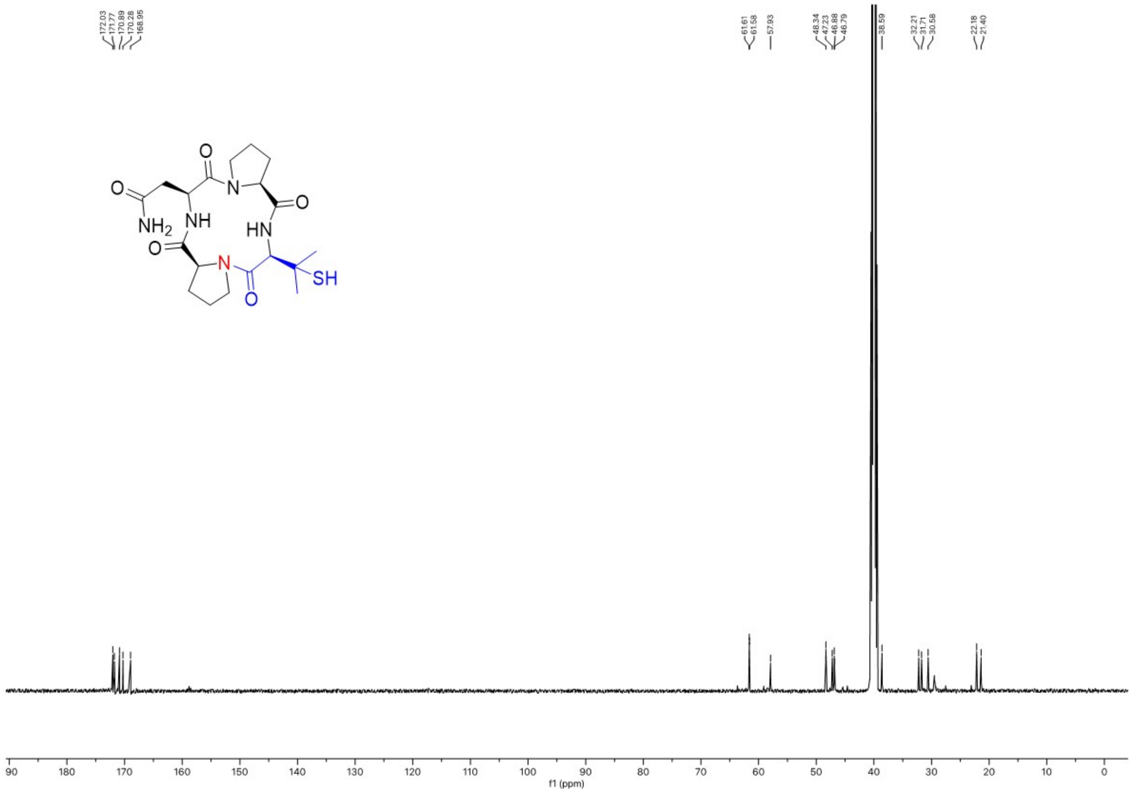

^{13}C NMR spectrum of compound **14d** (DMSO-*d6*, 151 MHz)

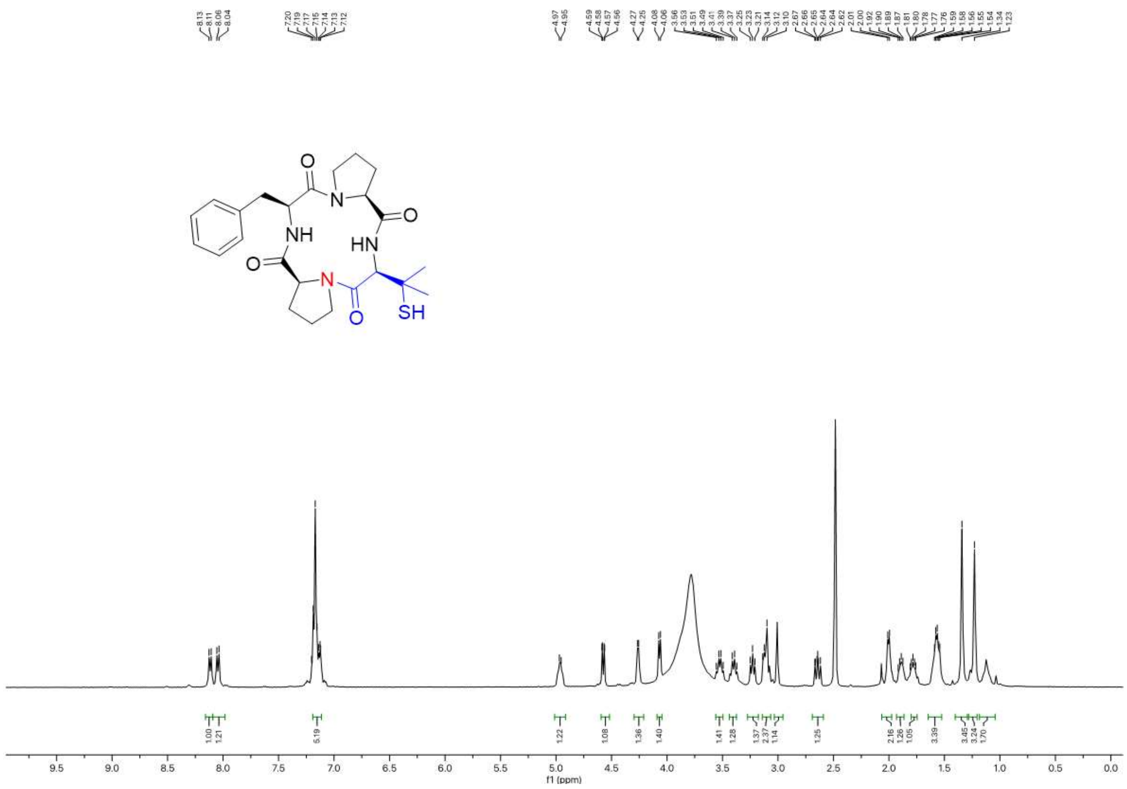

^{1}H NMR spectrum of compound **9b** (DMSO-*d6*, 600 MHz)

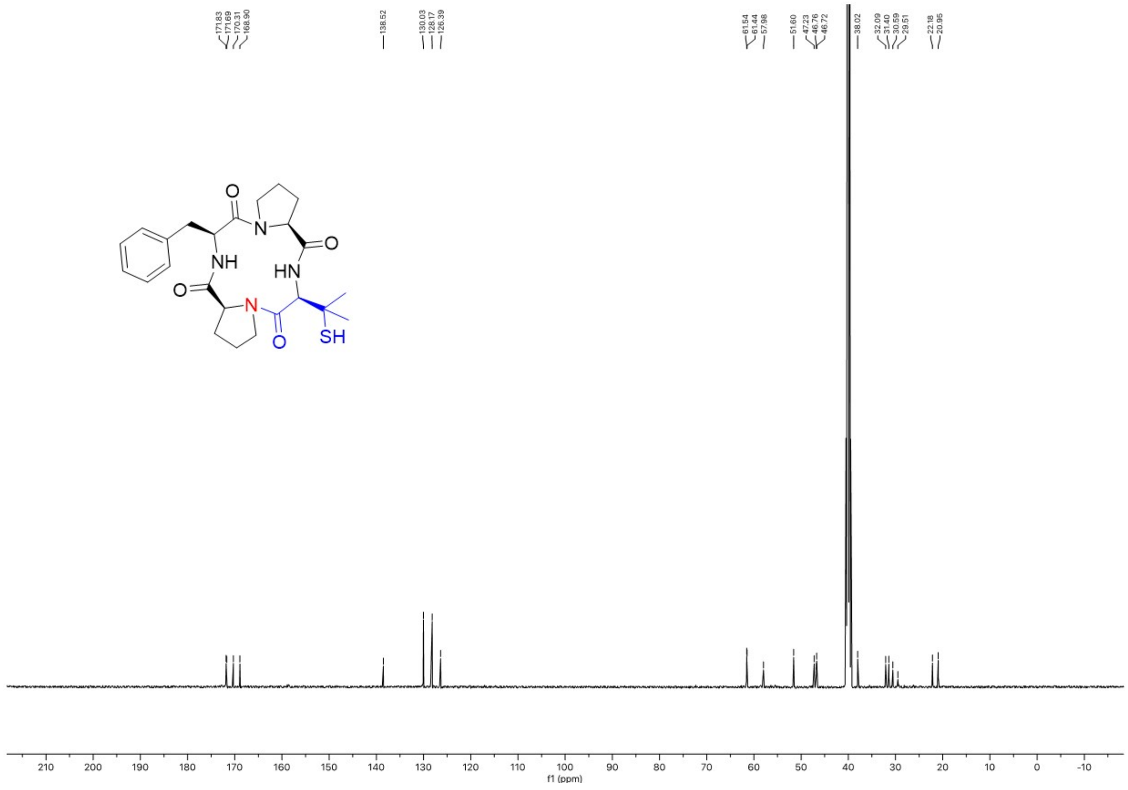

^{13}C NMR spectrum of compound **9b** (DMSO-*d6*, 151 MHz)

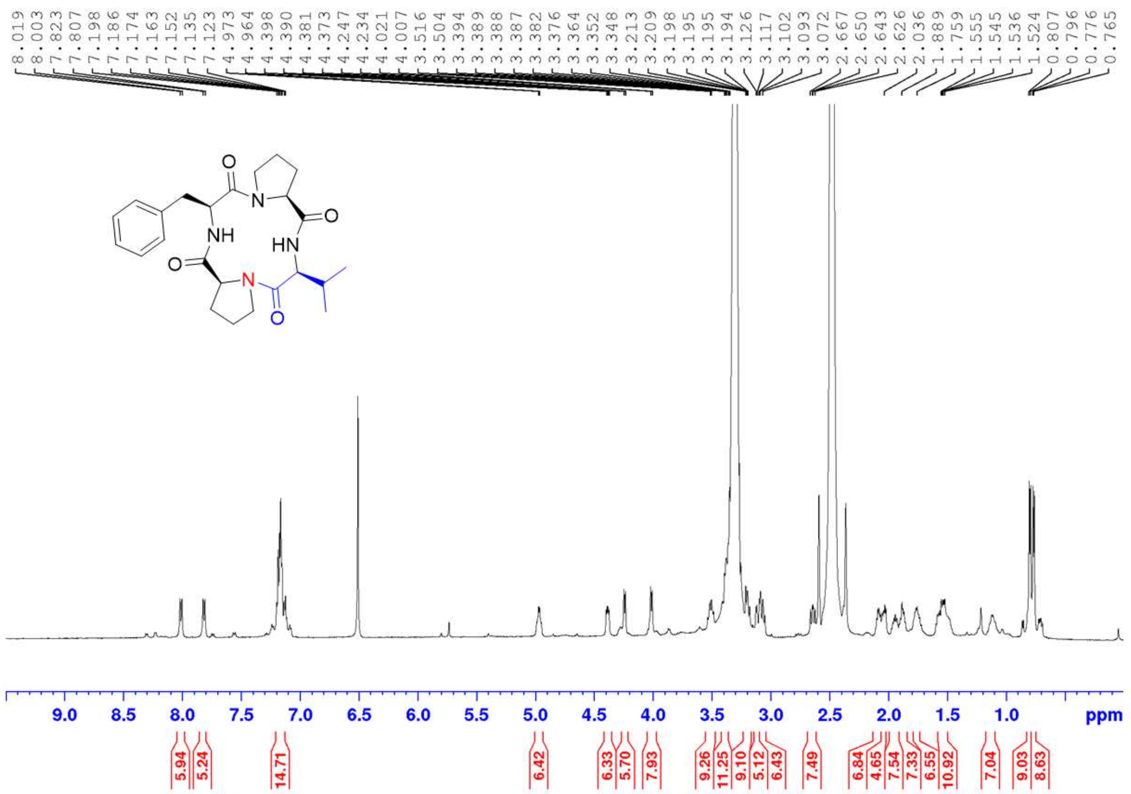

^{1}H NMR spectrum of compound **15e** (DMSO-*d*6, 600 MHz)

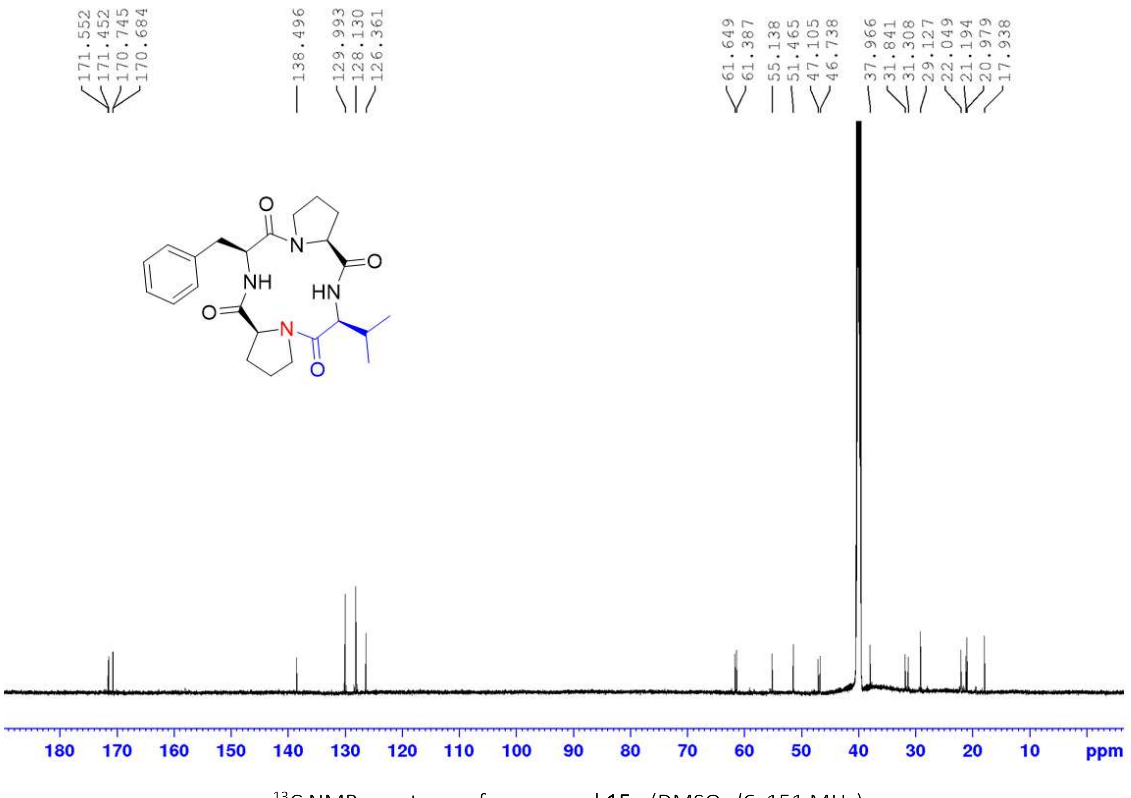

^{13}C NMR spectrum of compound **15e** (DMSO-*d6*, 151 MHz)

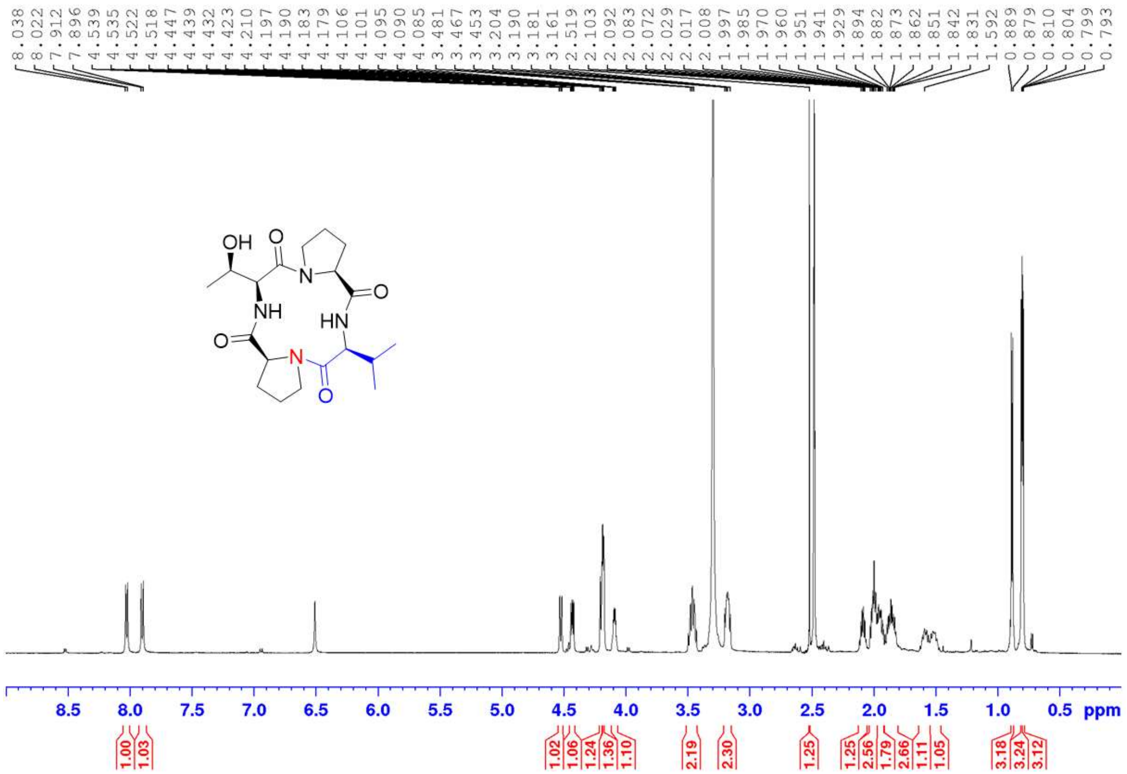

^{1}H NMR spectrum of compound **15f** (DMSO-*d6*, 600 MHz)

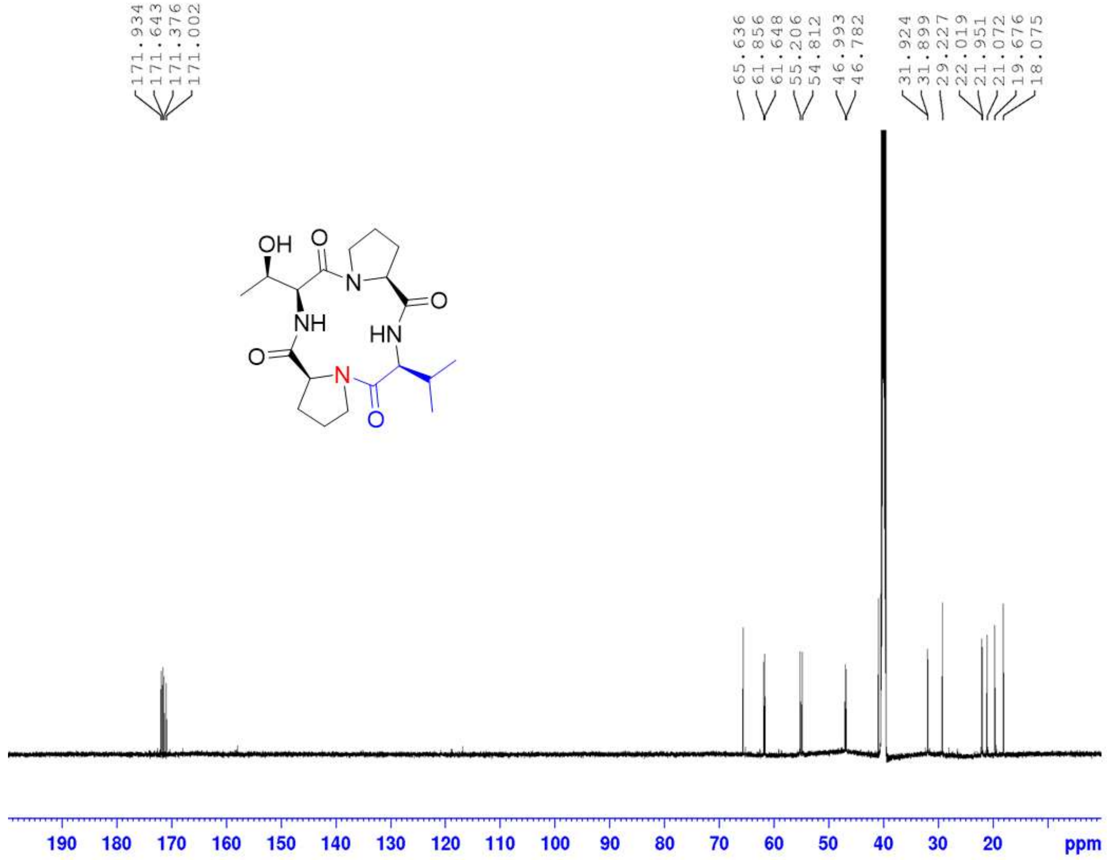

^{13}C NMR spectrum of compound **15f** (DMSO-*d6*, 151 MHz)

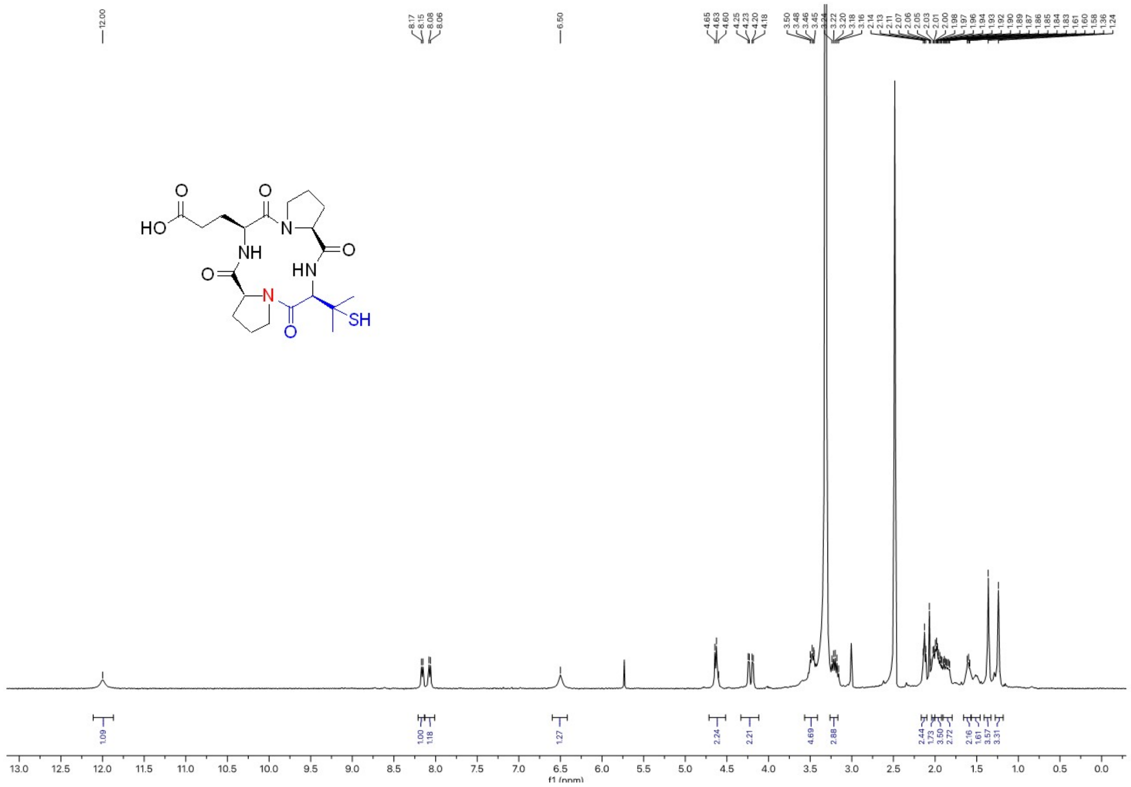

^{1}H NMR spectrum of compound **14g** (DMSO-*d6*, 500 MHz)

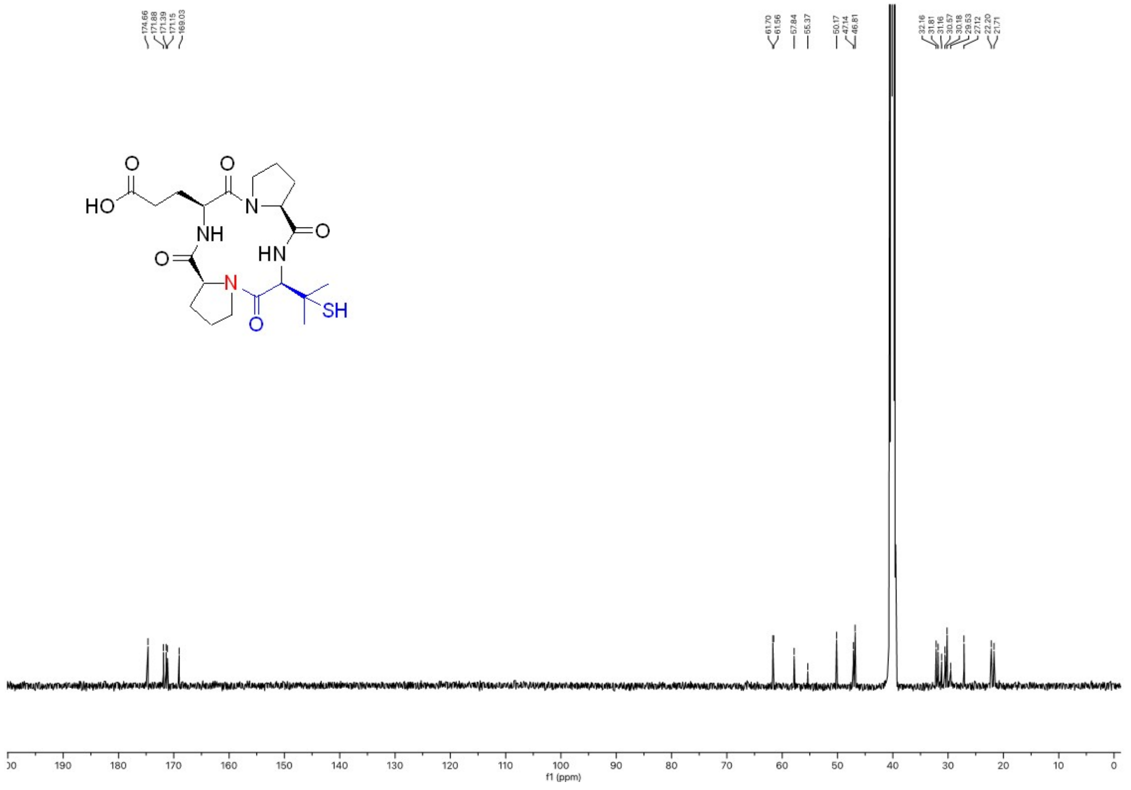

^{13}C NMR spectrum of compound **14g** (DMSO-*d6*, 151 MHz)

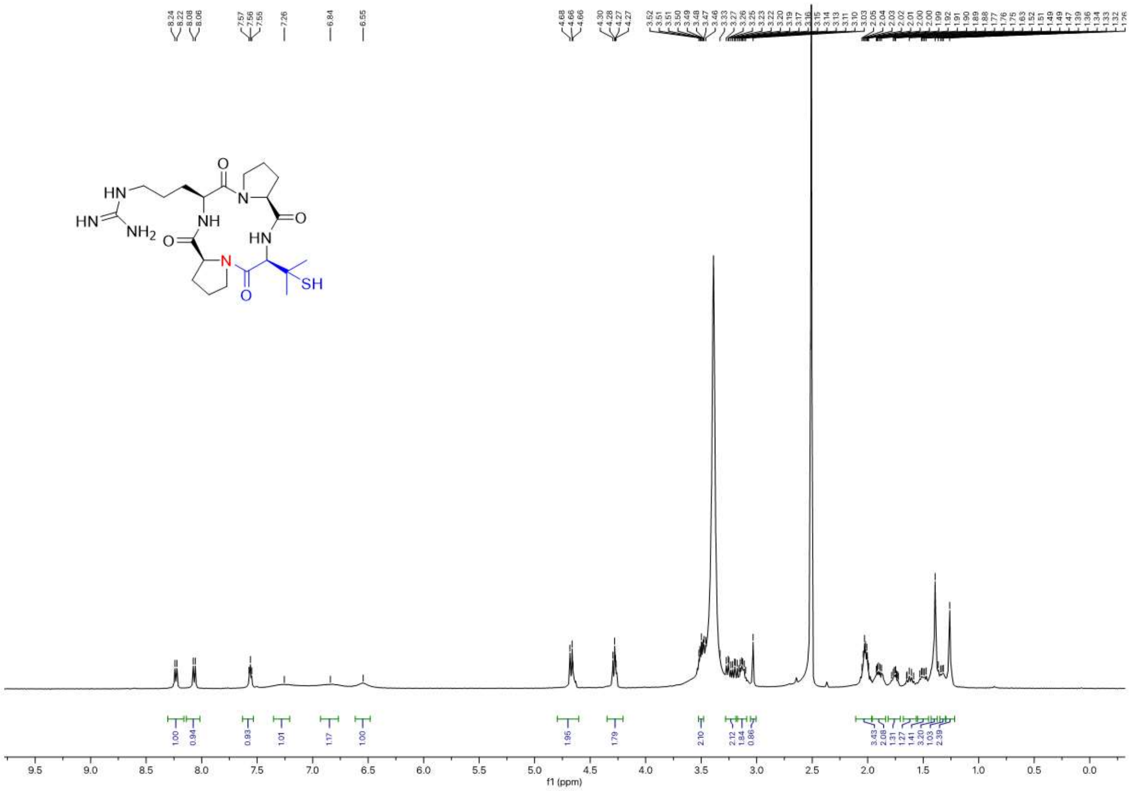

^{1}H NMR spectrum of compound **14h** (DMSO-*d6*, 600 MHz)

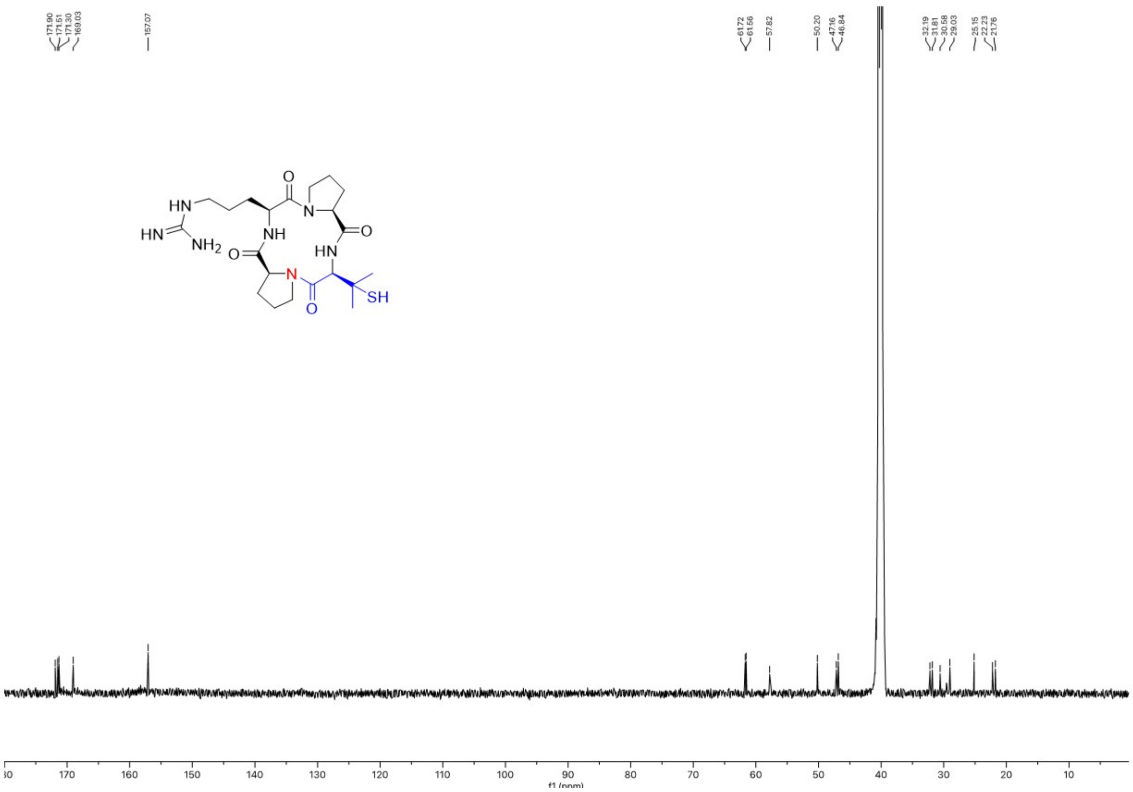

^{13}C NMR spectrum of compound **14h** (DMSO-*d6*, 151 MHz)

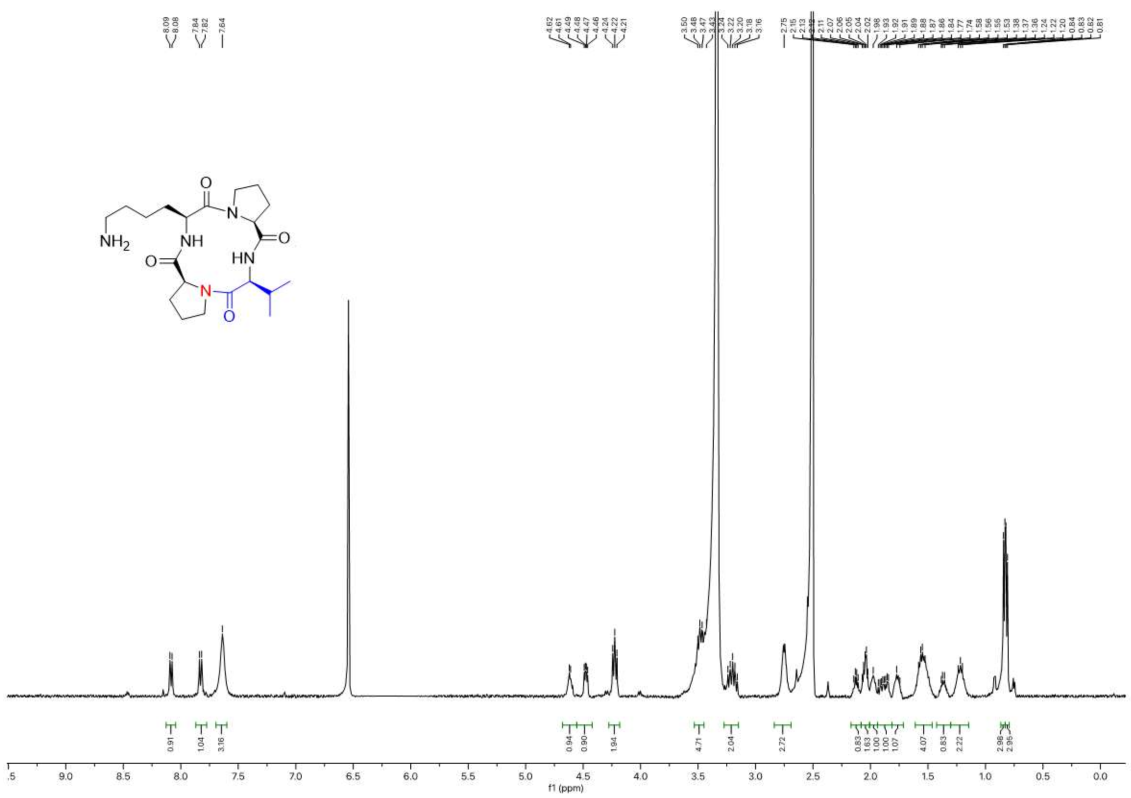

^{1}H NMR spectrum of compound **15i** (DMSO-*d6*, 500 MHz)

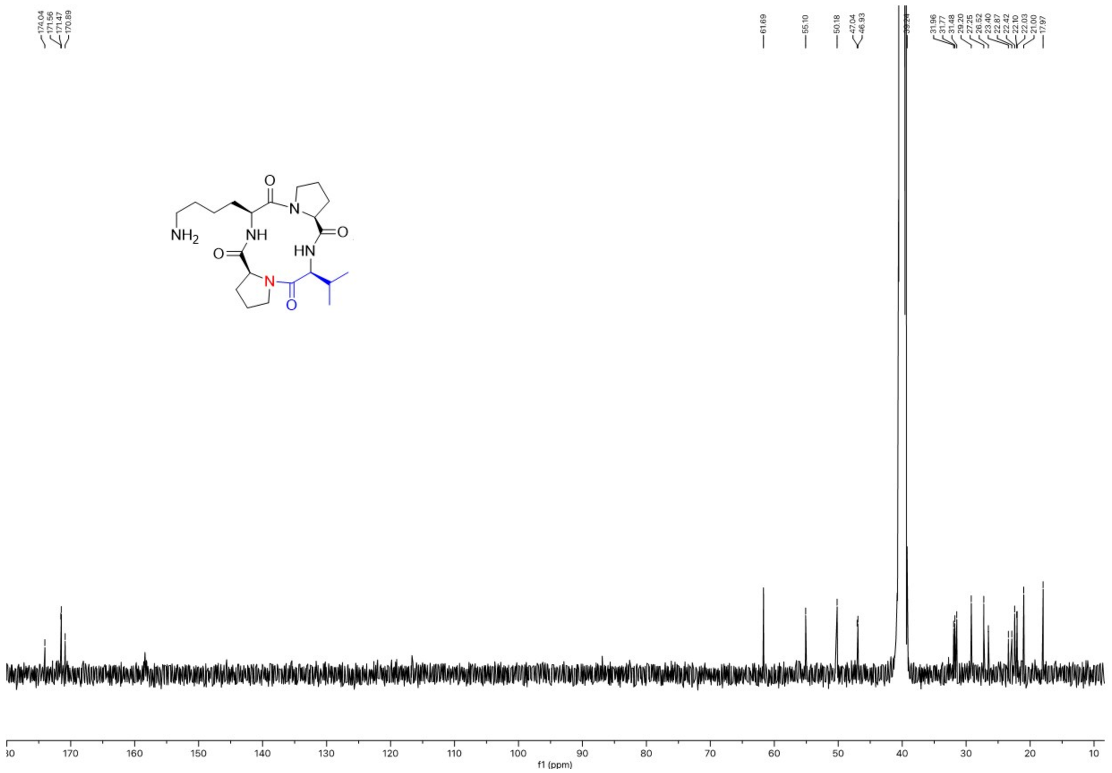

^{13}C NMR spectrum of compound **15i** (DMSO-*d6*, 126 MHz)

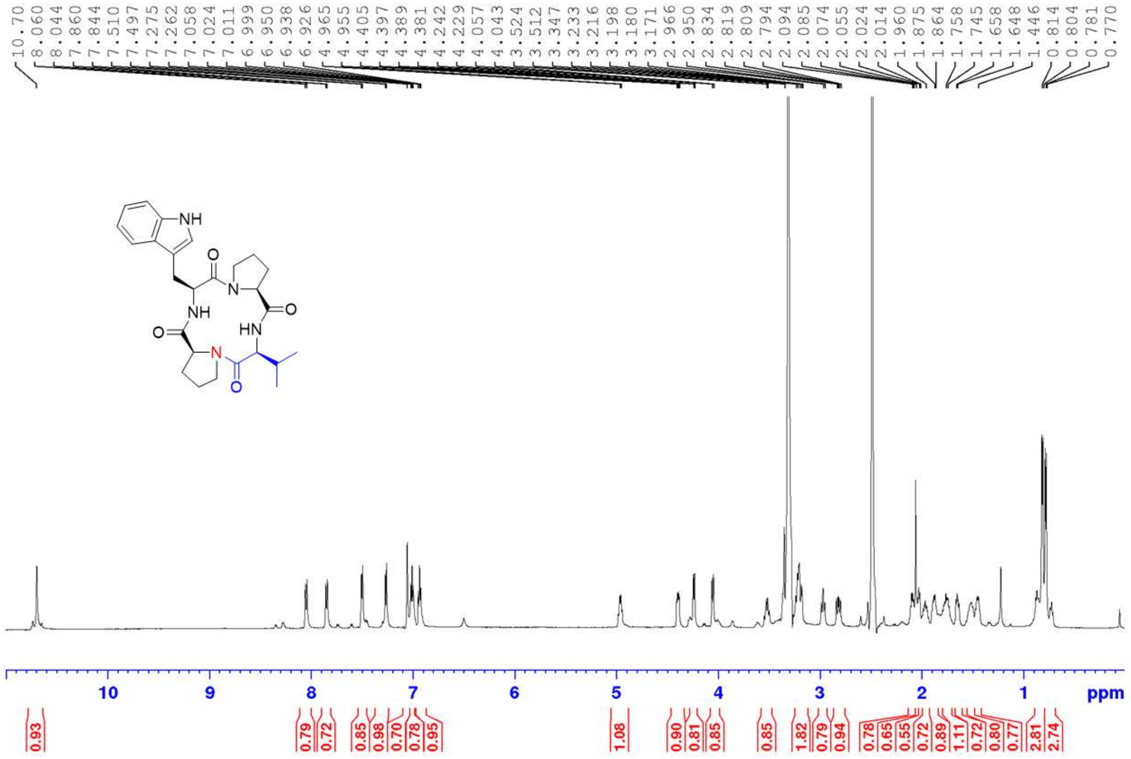

^{1}H NMR spectrum of compound **15j** (DMSO-d6, 600 MHz)

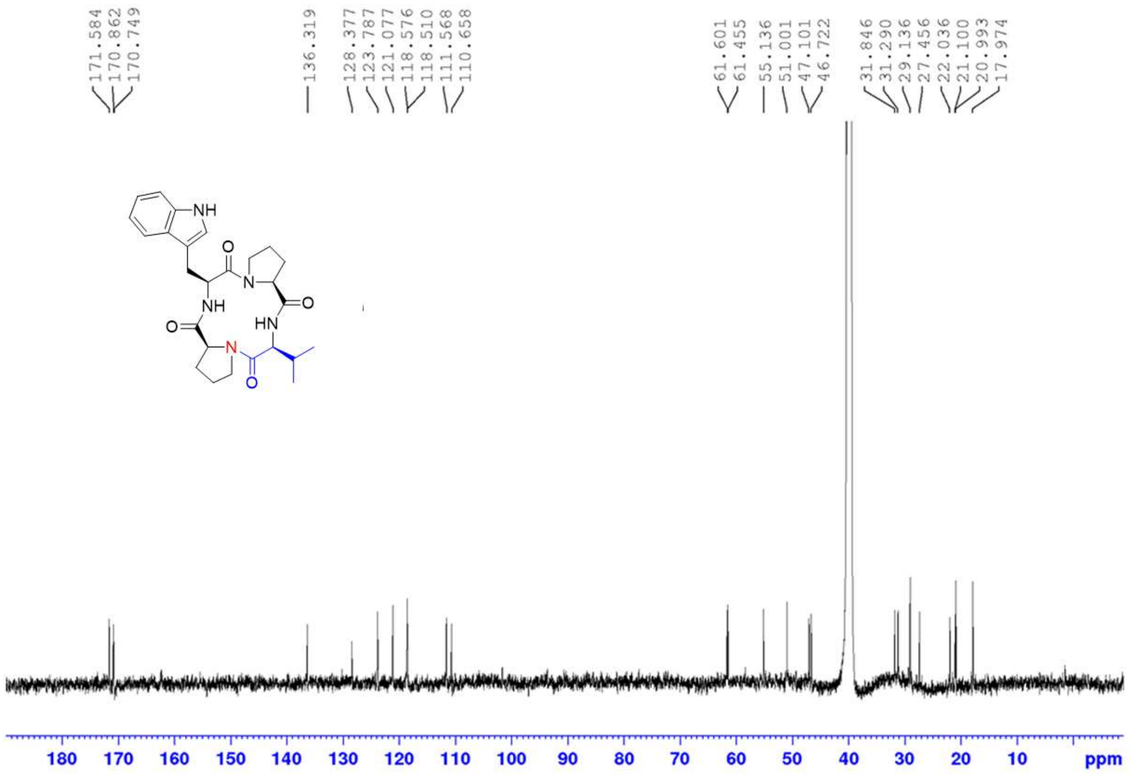

^{13}C NMR spectrum of compound **15j** (DMSO-*d6*, 151 MHz)

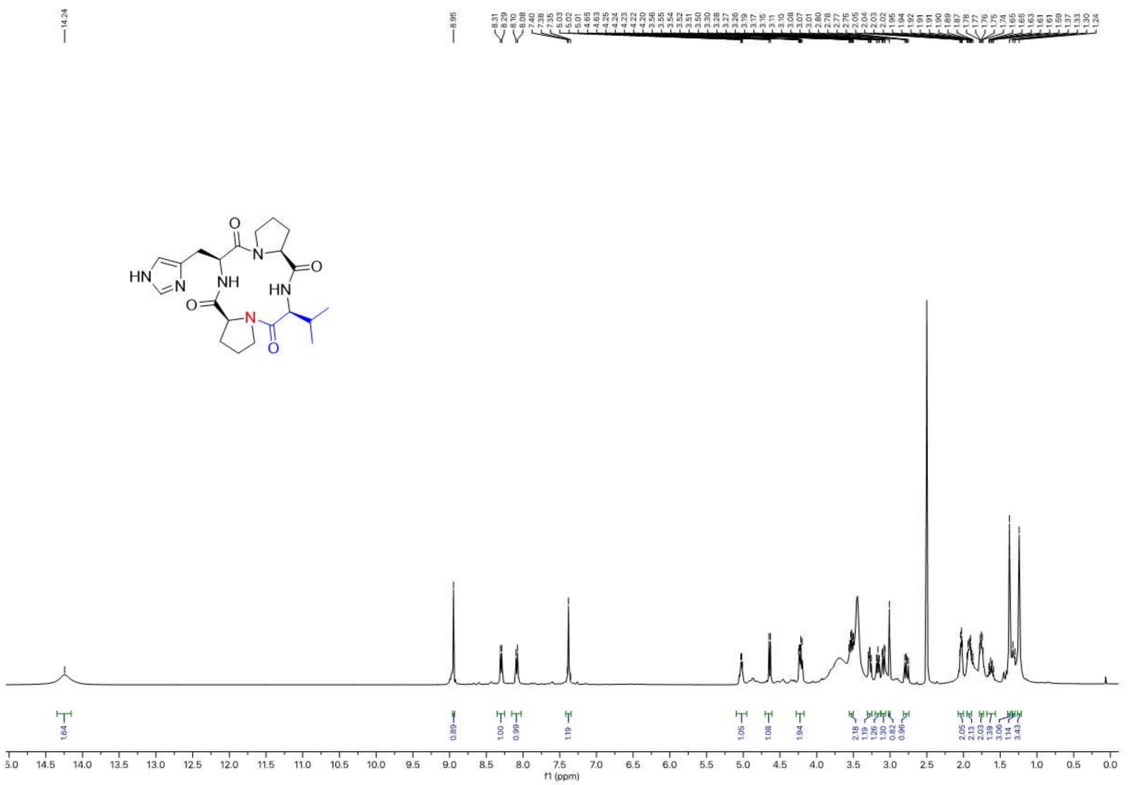

^{1}H NMR spectrum of compound **14k** (DMSO-*d6*, 500 MHz)

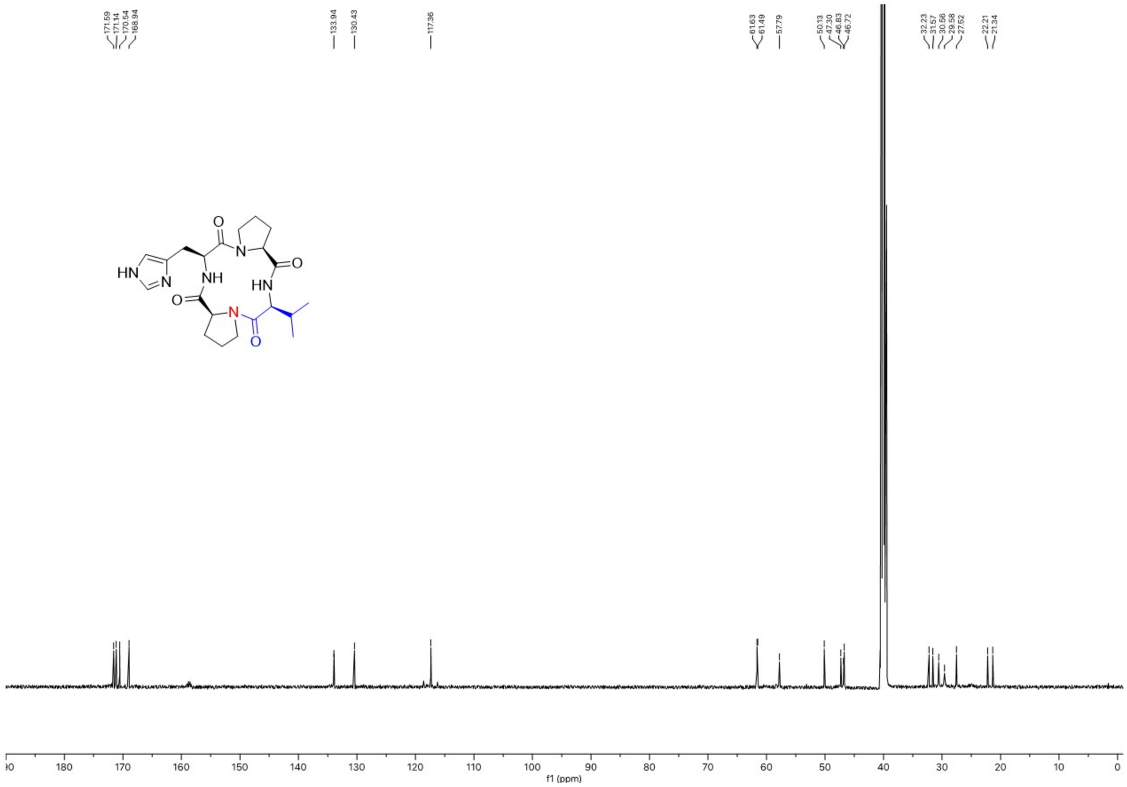

^{13}C NMR spectrum of compound **14k** (DMSO-*d6*, 151 MHz)

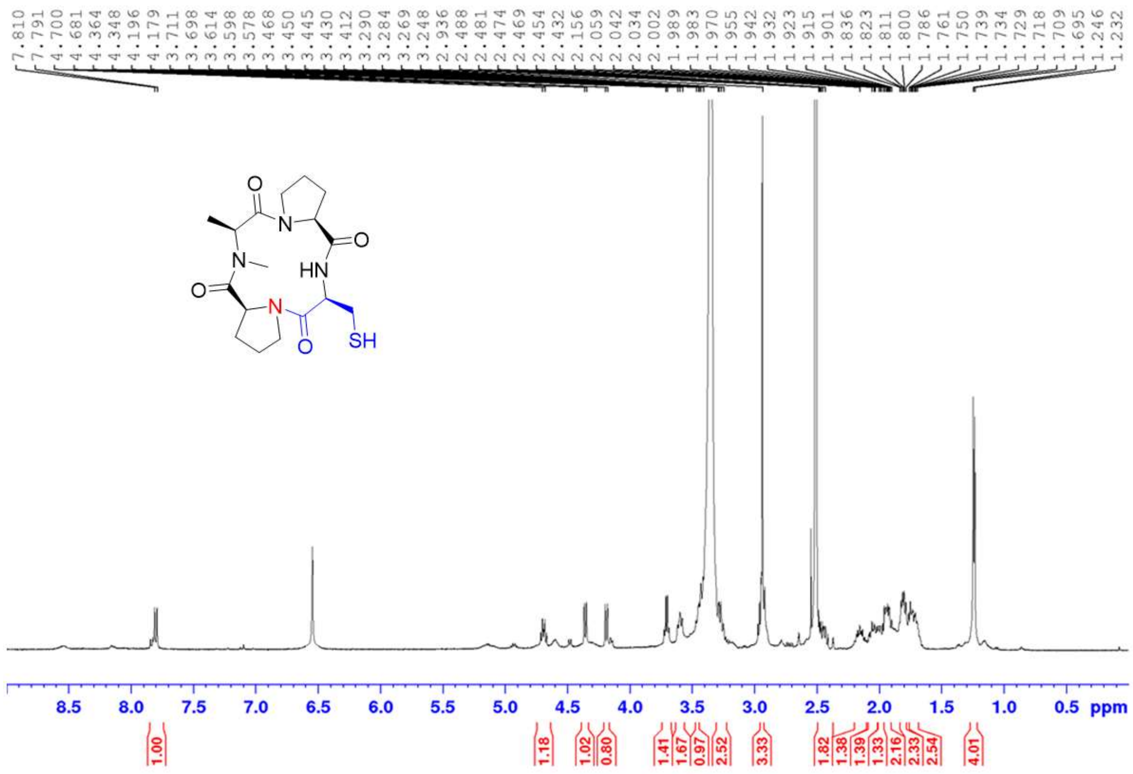

^{1}H NMR spectrum of compound **14l** (DMSO-*d6*, 500 MHz)

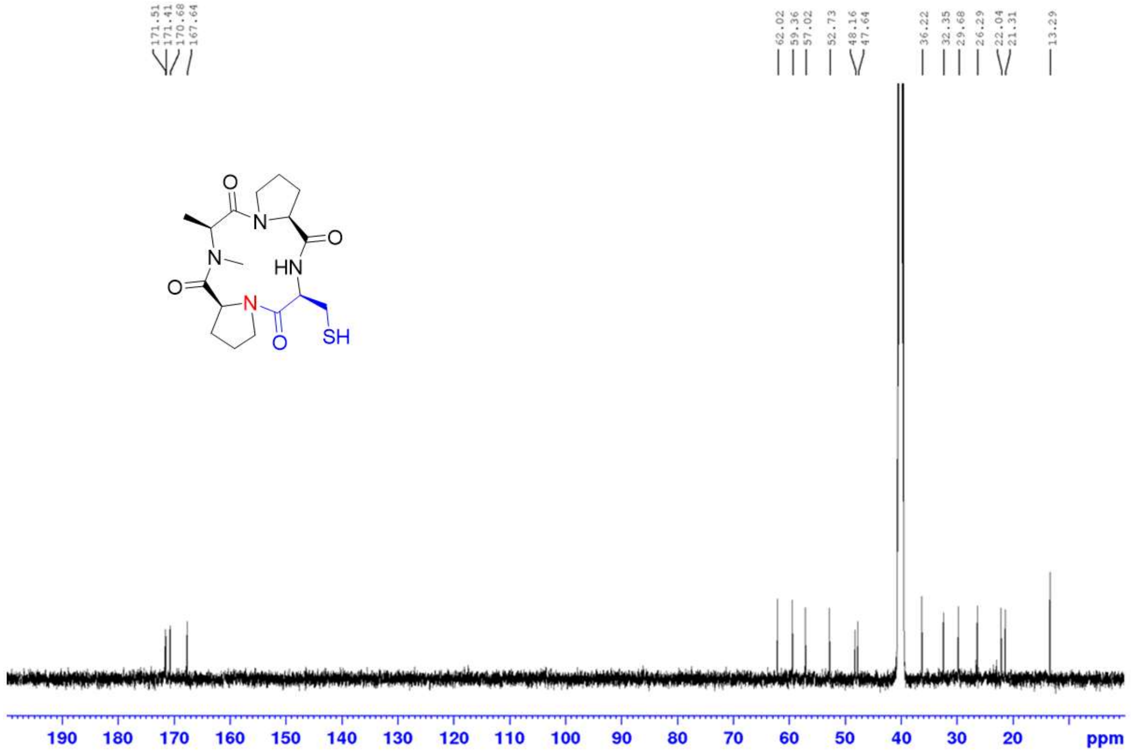

^{13}C NMR spectrum of compound **14l** (DMSO-*d6*, 126 MHz)

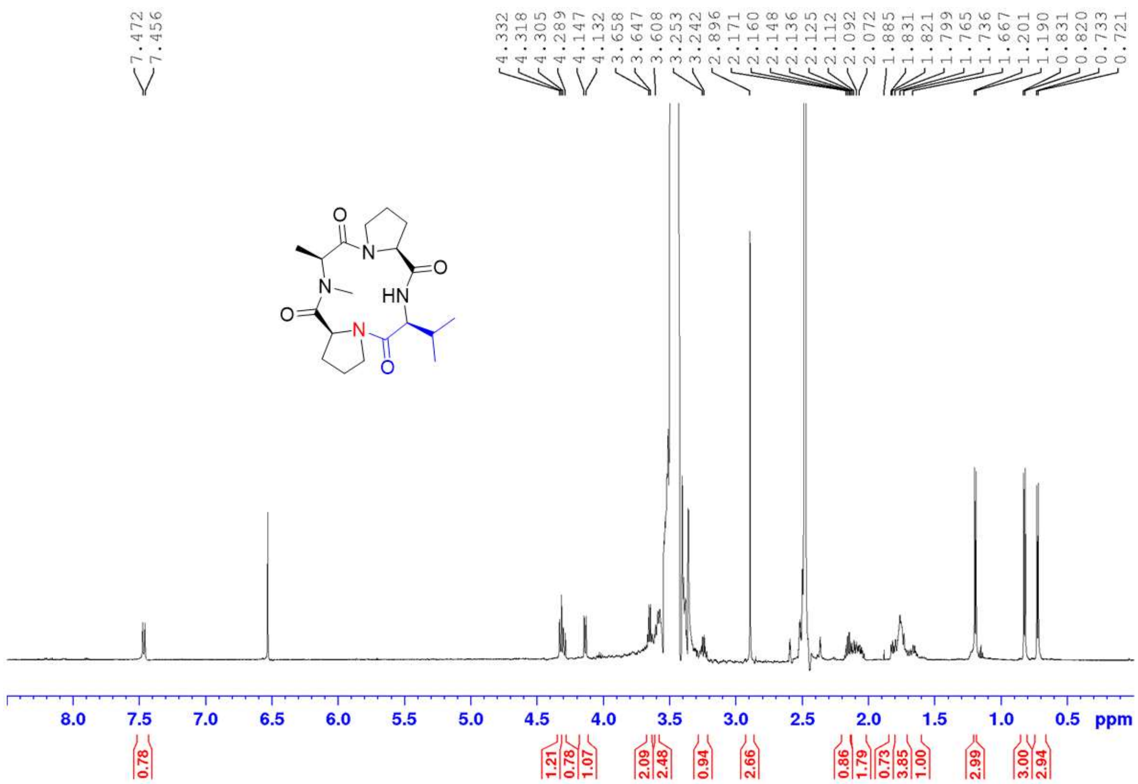

^{1}H NMR spectrum of compound **15m** (DMSO-d6, 600 MHz)

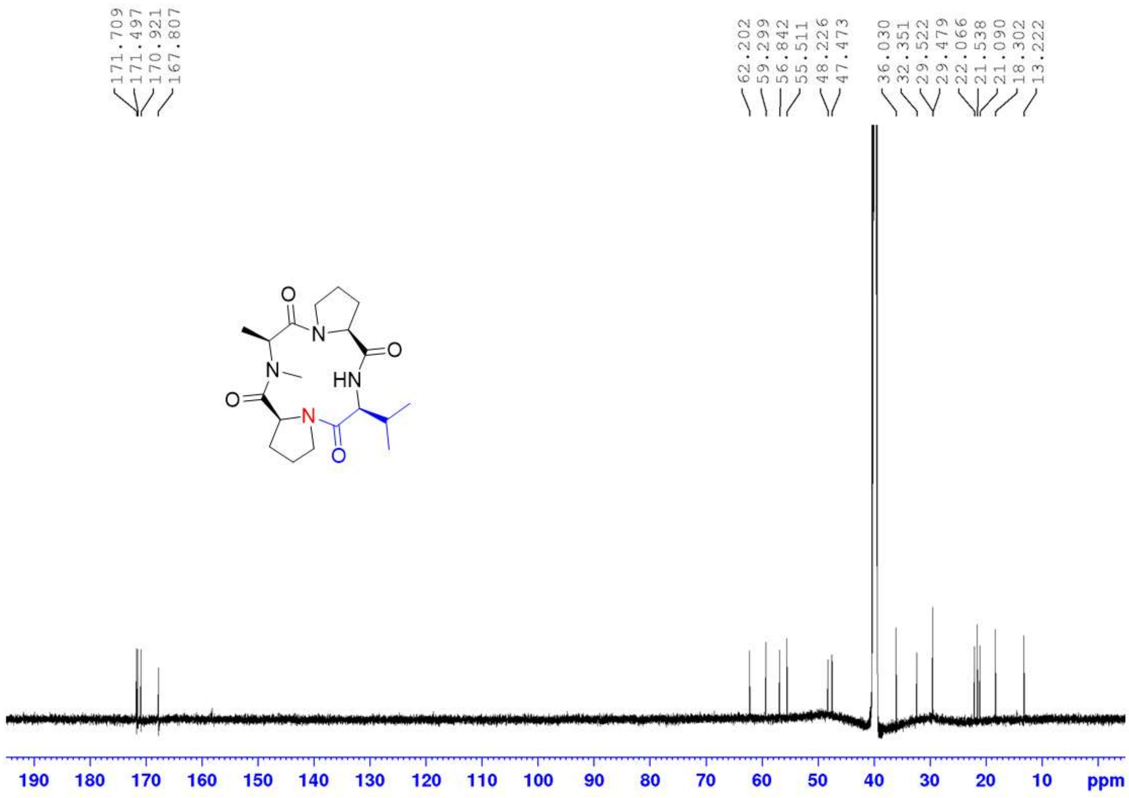

^{13}C NMR spectrum of compound **15m** (DMSO-*d6*, 151 MHz)

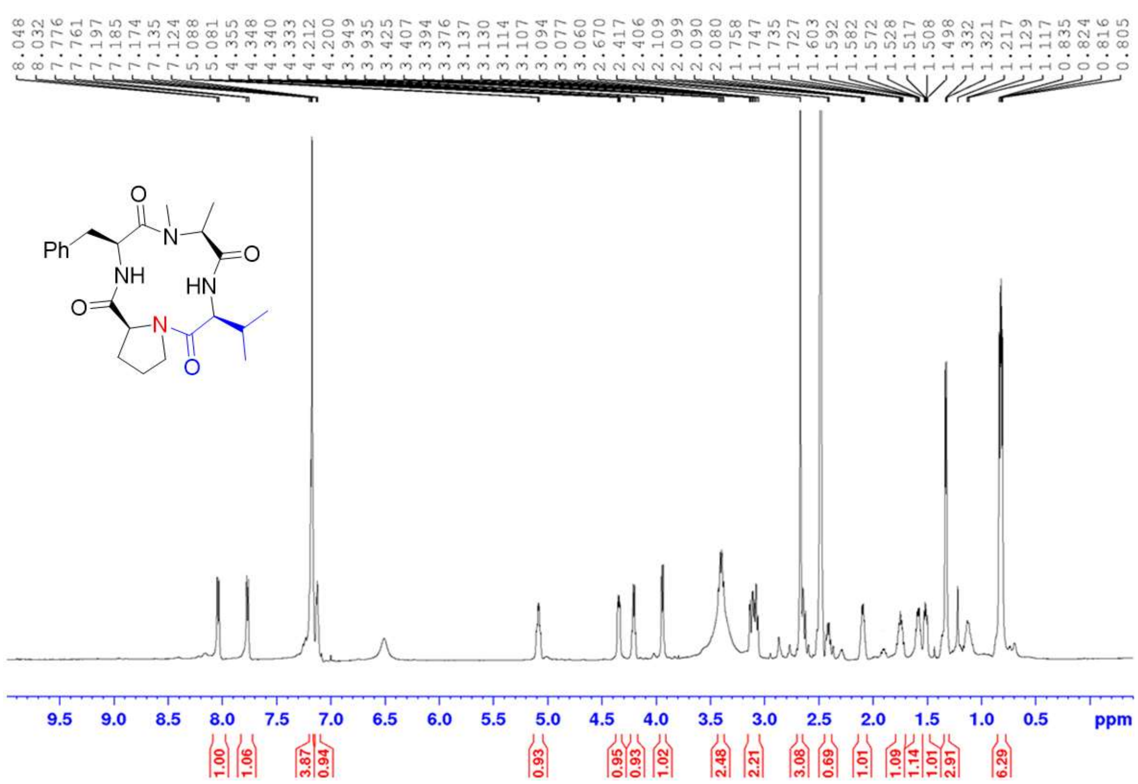

^{1}H NMR spectrum of compound **15n** (DMSO-d6, 600 MHz)

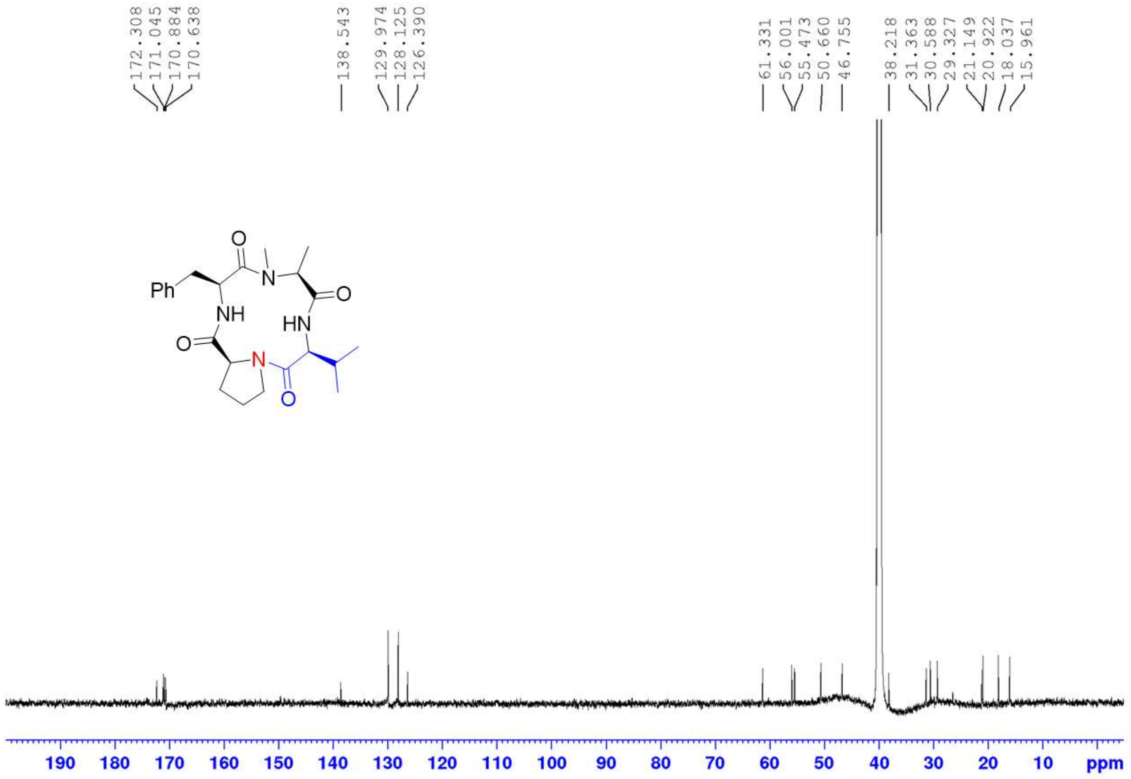

^{13}C NMR spectrum of compound **15n** (DMSO-*d6*, 151 MHz)

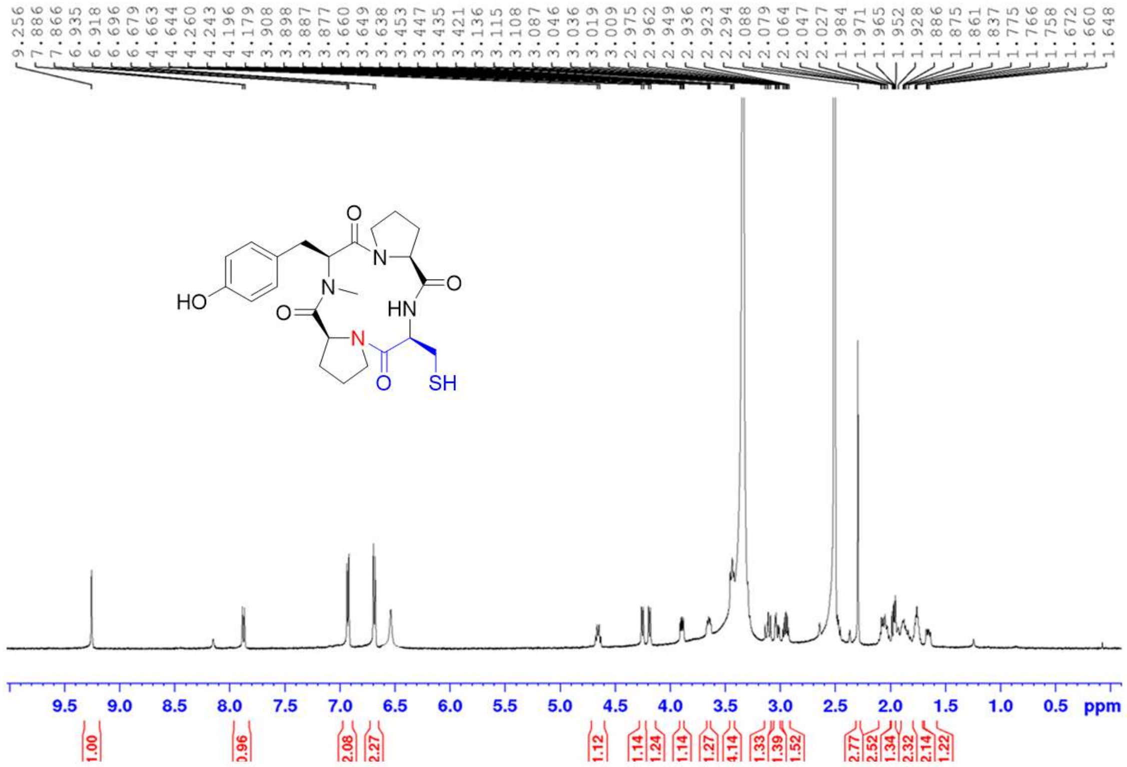

^{1}H NMR spectrum of compound **14o** (DMSO-*d6*, 500 MHz)

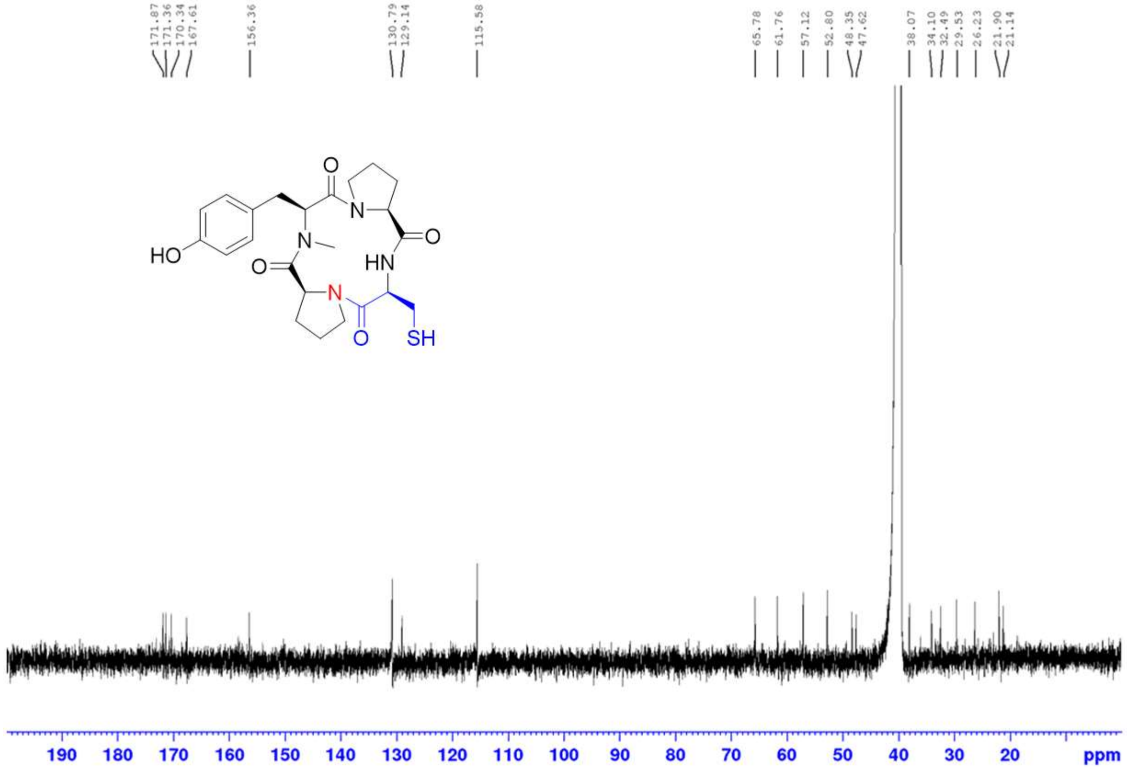

^{13}C NMR spectrum of compound **14o** (DMSO-*d6*, 126 MHz)

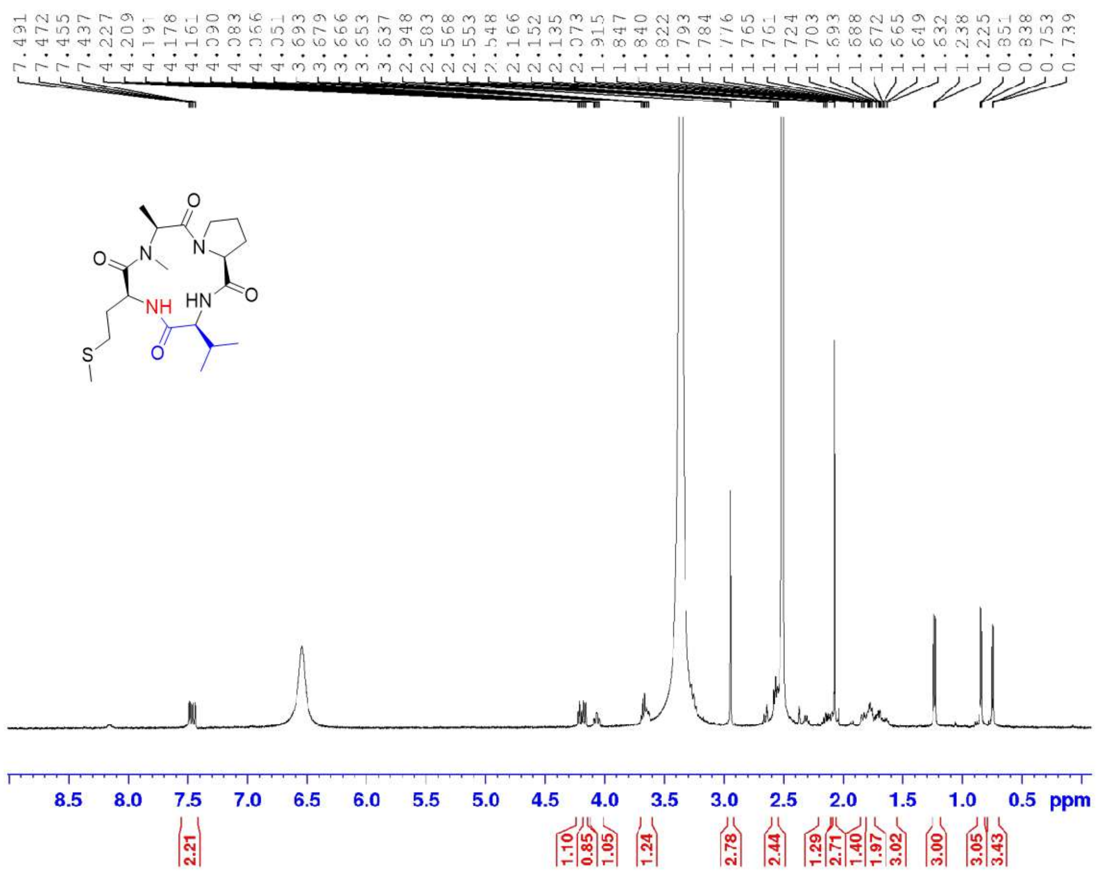

^{1}H NMR spectrum of compound **15p** (DMSO-*d6*, 500 MHz)

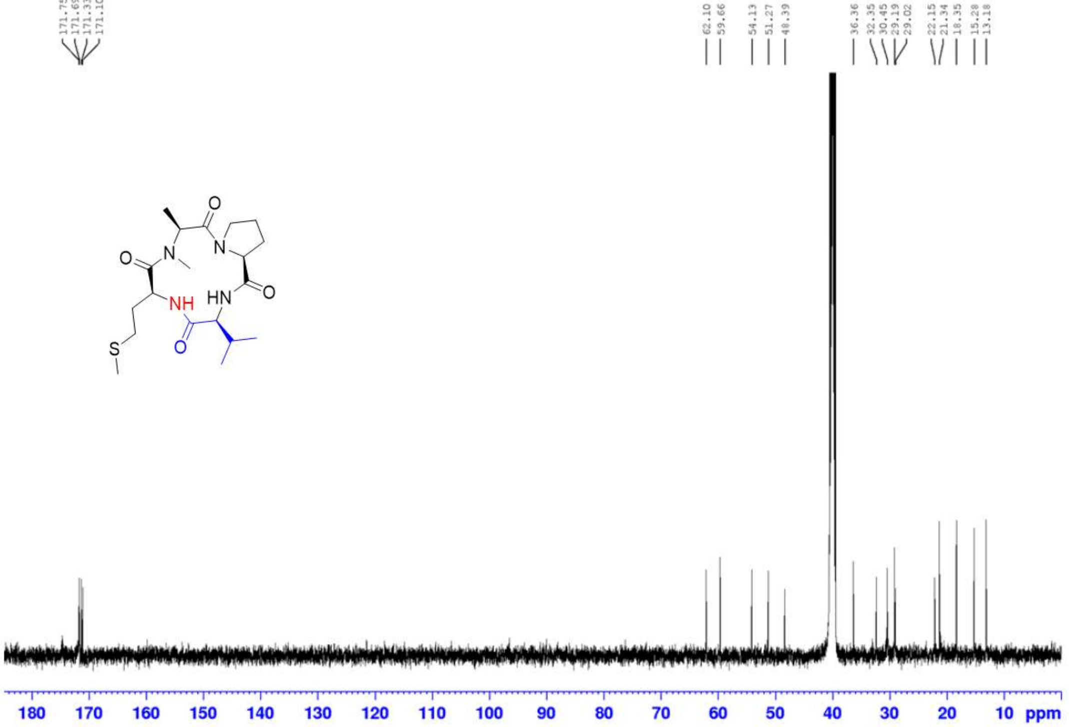

^{13}C NMR spectrum of compound **15p** (DMSO-*d6*, 151 MHz)

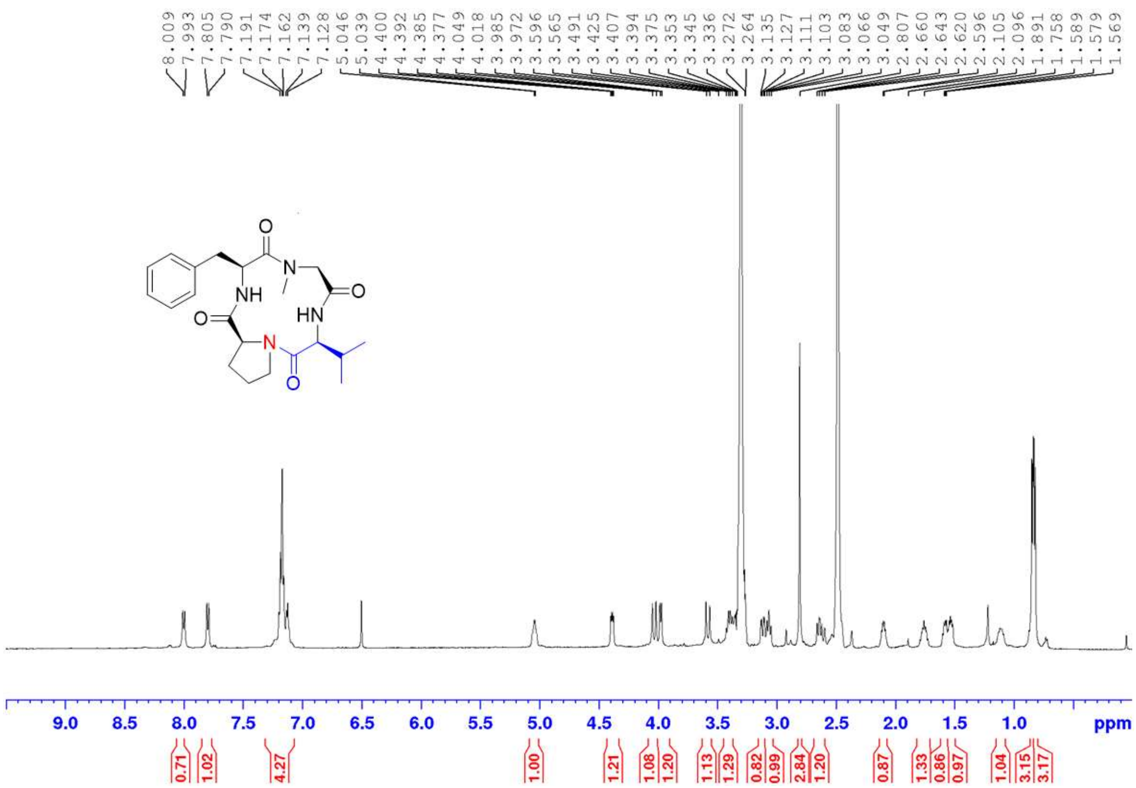

^{1}H NMR spectrum of compound **15q** (DMSO-d6, 600 MHz)

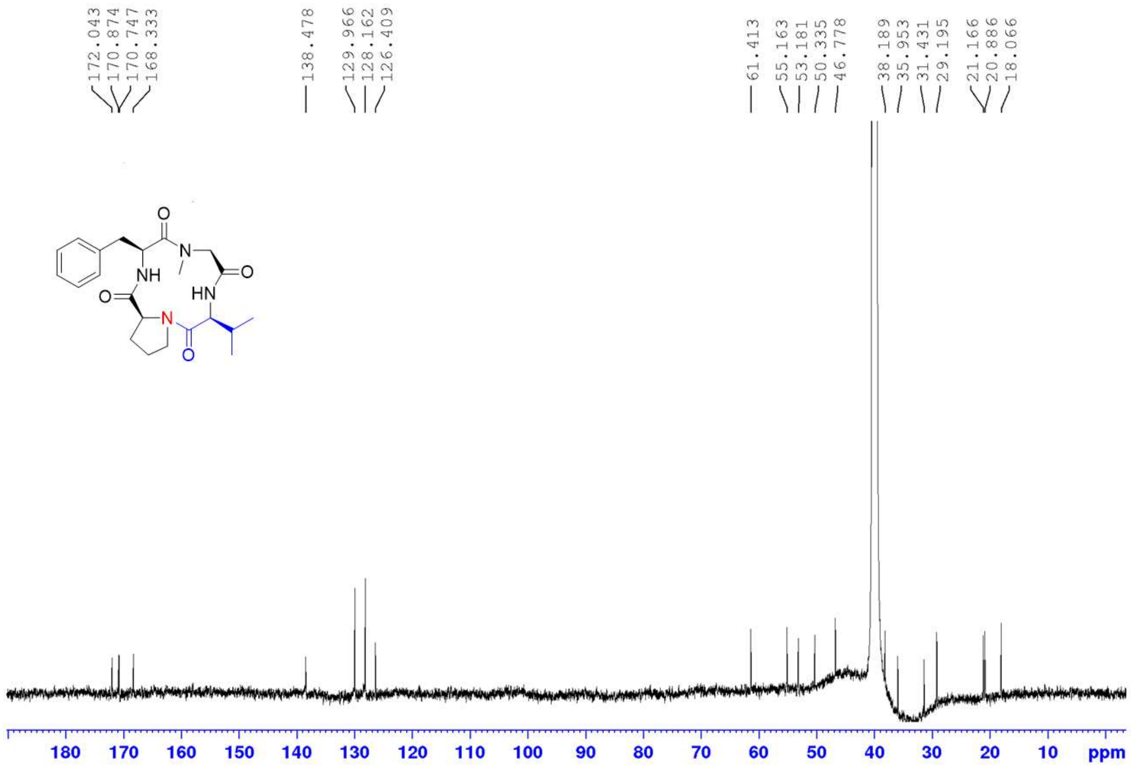

^{13}C NMR spectrum of compound **15q** (DMSO-*d6*, 151 MHz)

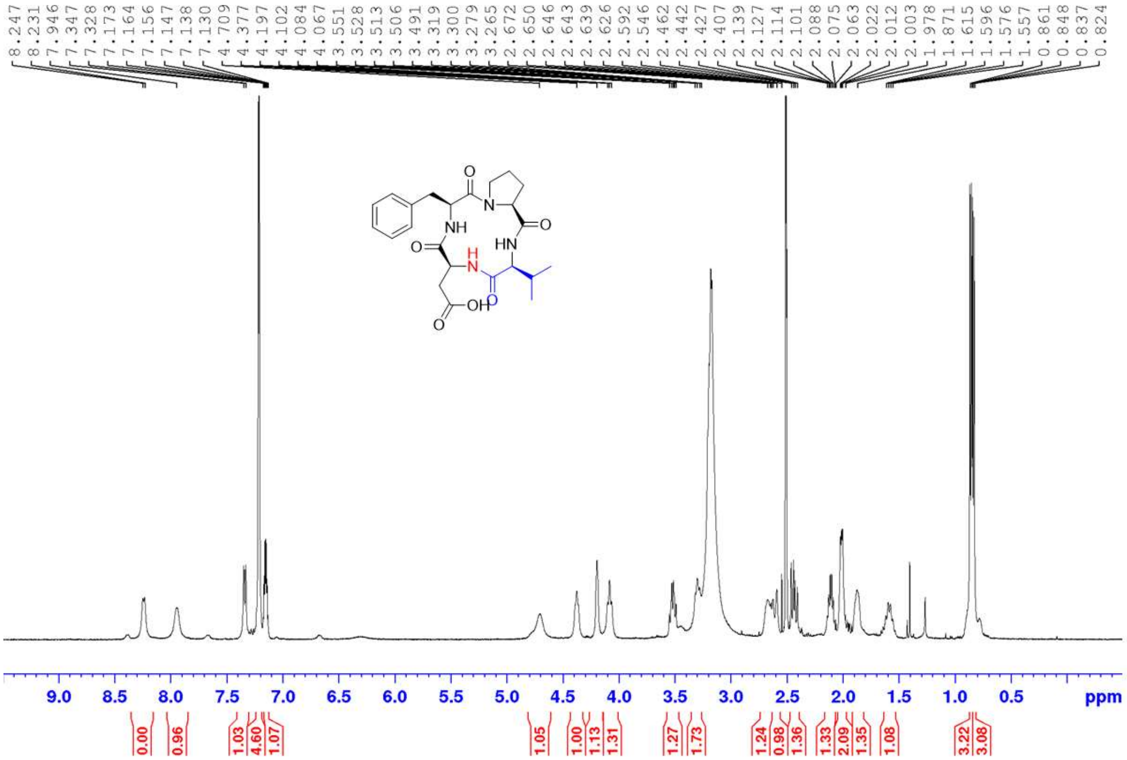

^{1}H NMR spectrum of compound **15r** (DMSO-d6, 500 MHz)

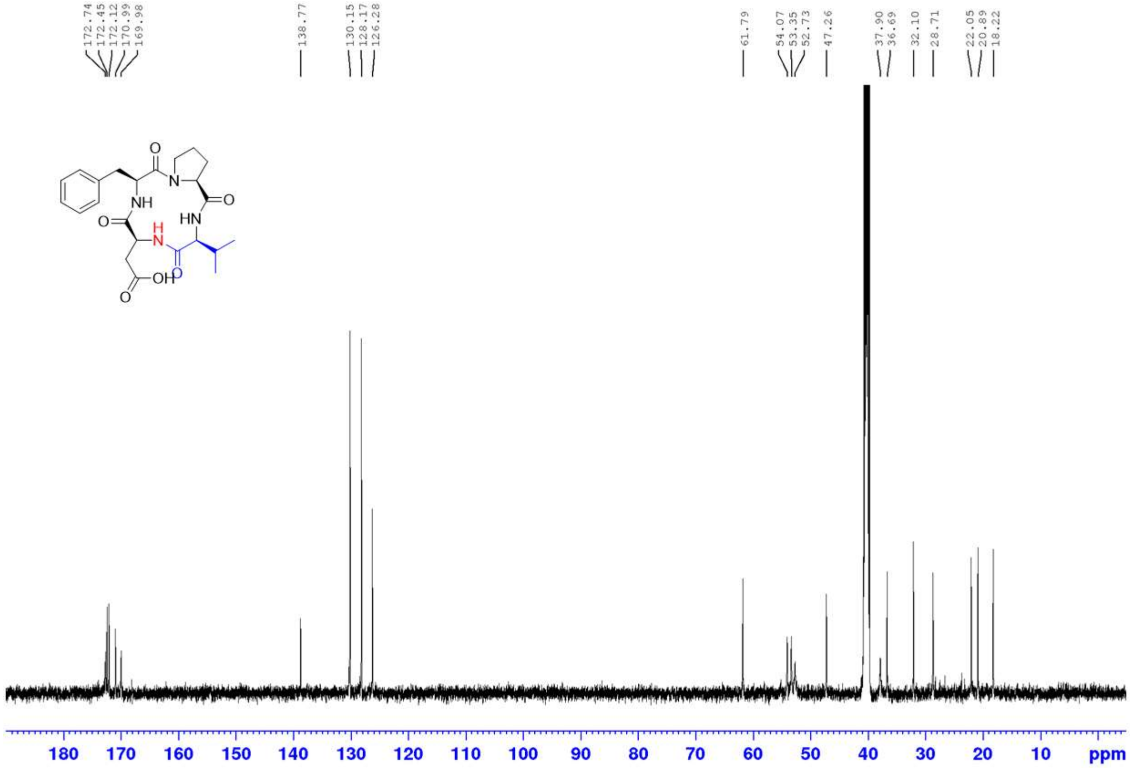

^{13}C NMR spectrum of compound **15r** (DMSO-*d6*, 126 MHz)

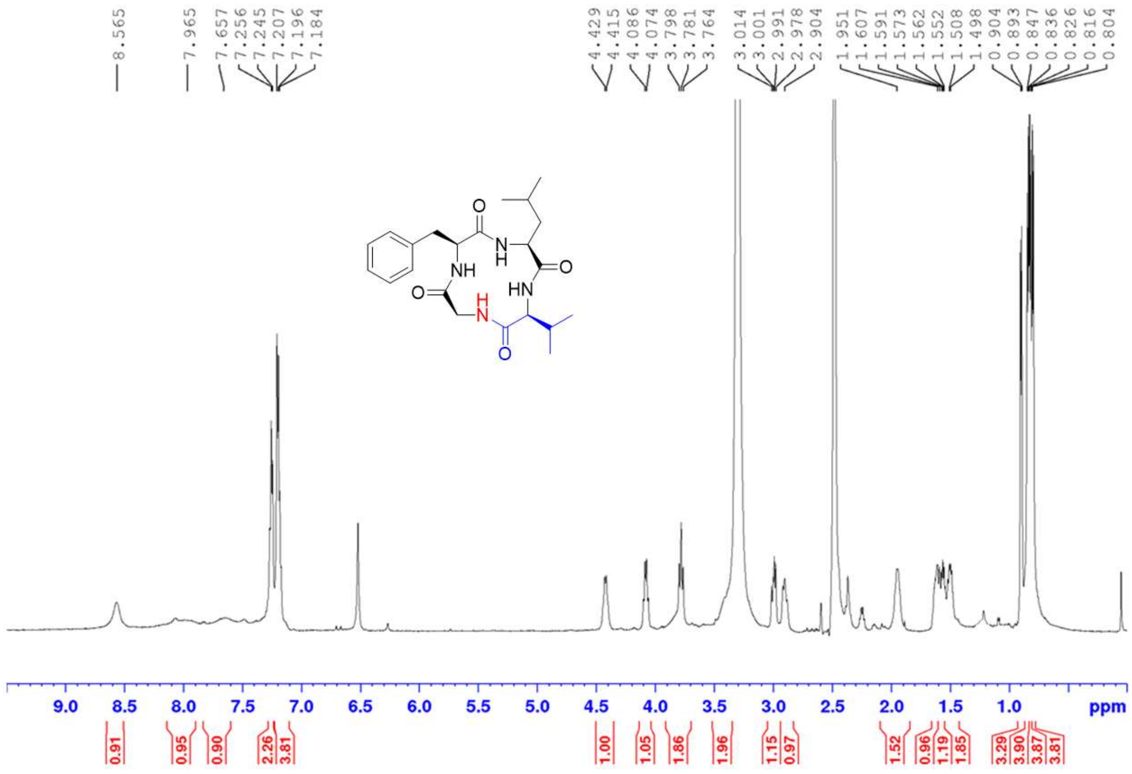

^{1}H NMR spectrum of compound **15s** (DMSO-d6, 600 MHz)

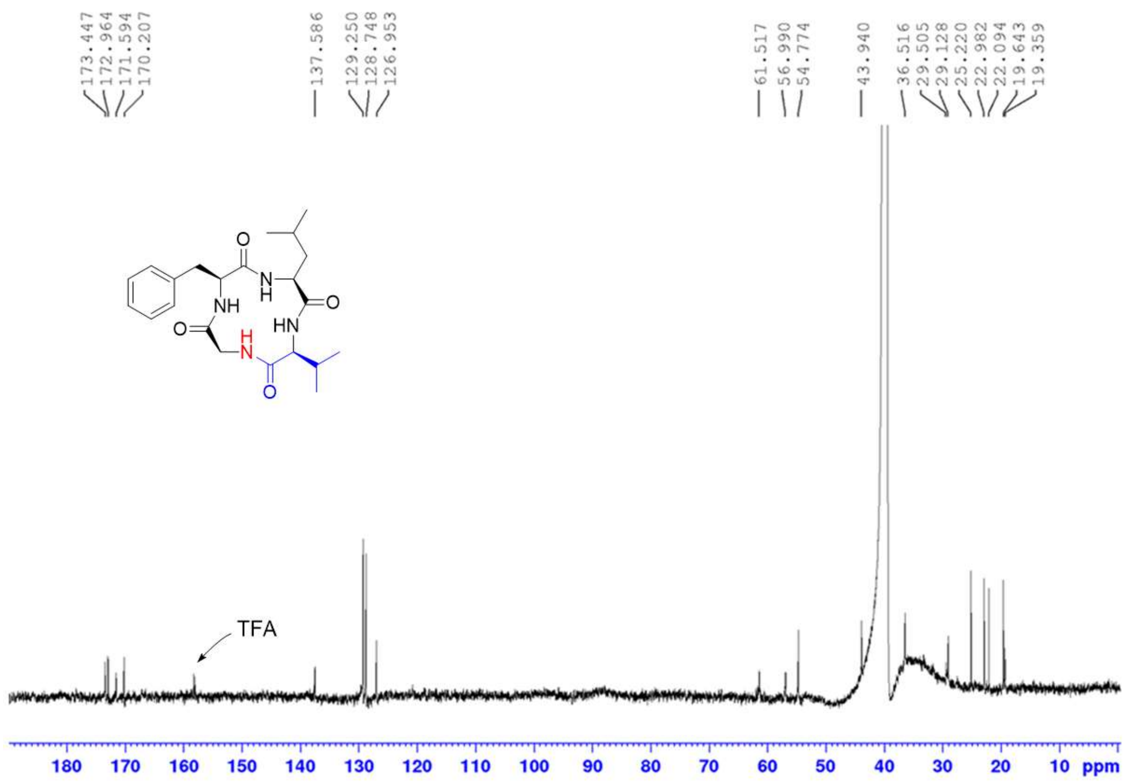

^{13}C NMR spectrum of compound **15s** (DMSO-*d6*, 151 MHz)

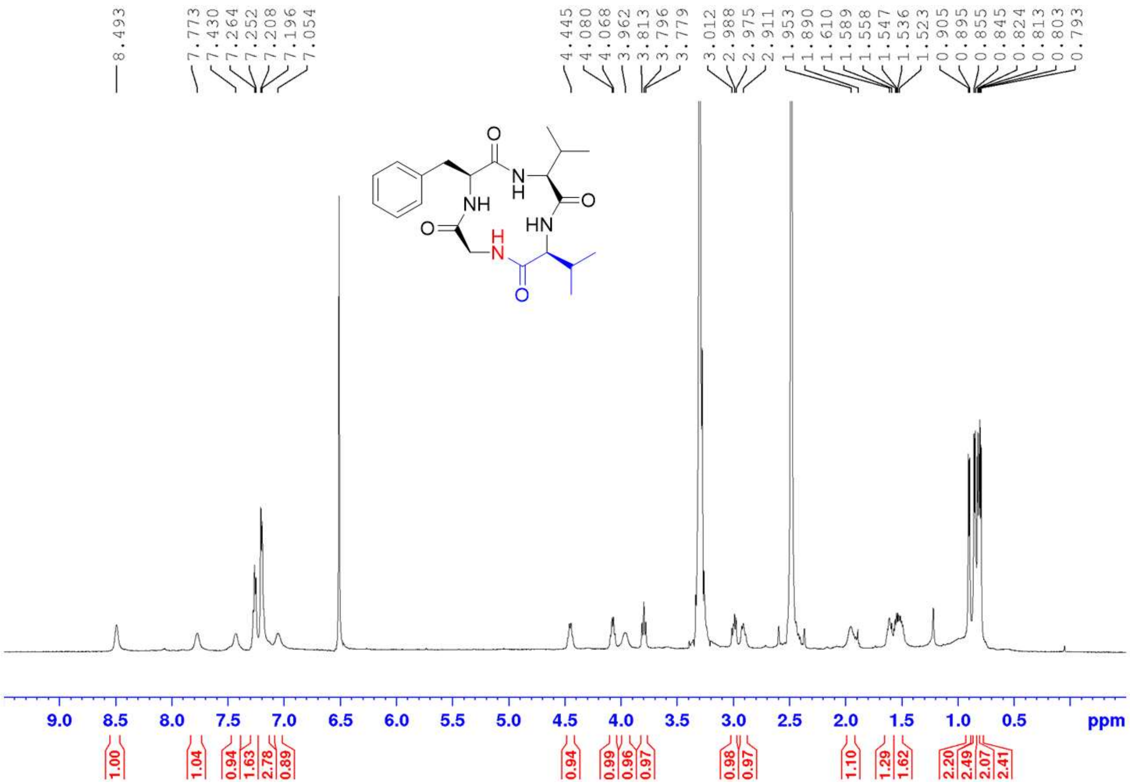

^{1}H NMR spectrum of compound **15t** (DMSO-d6, 600 MHz)

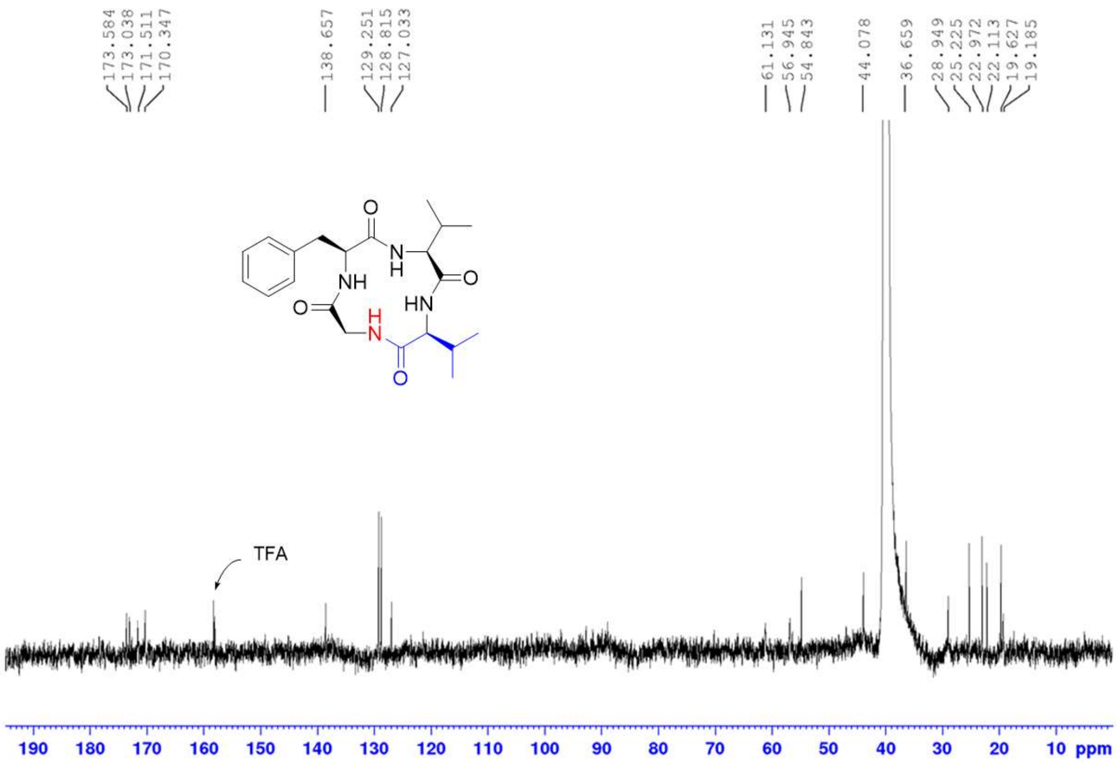

^{13}C NMR spectrum of compound **15t** (DMSO-*d6*, 151 MHz)

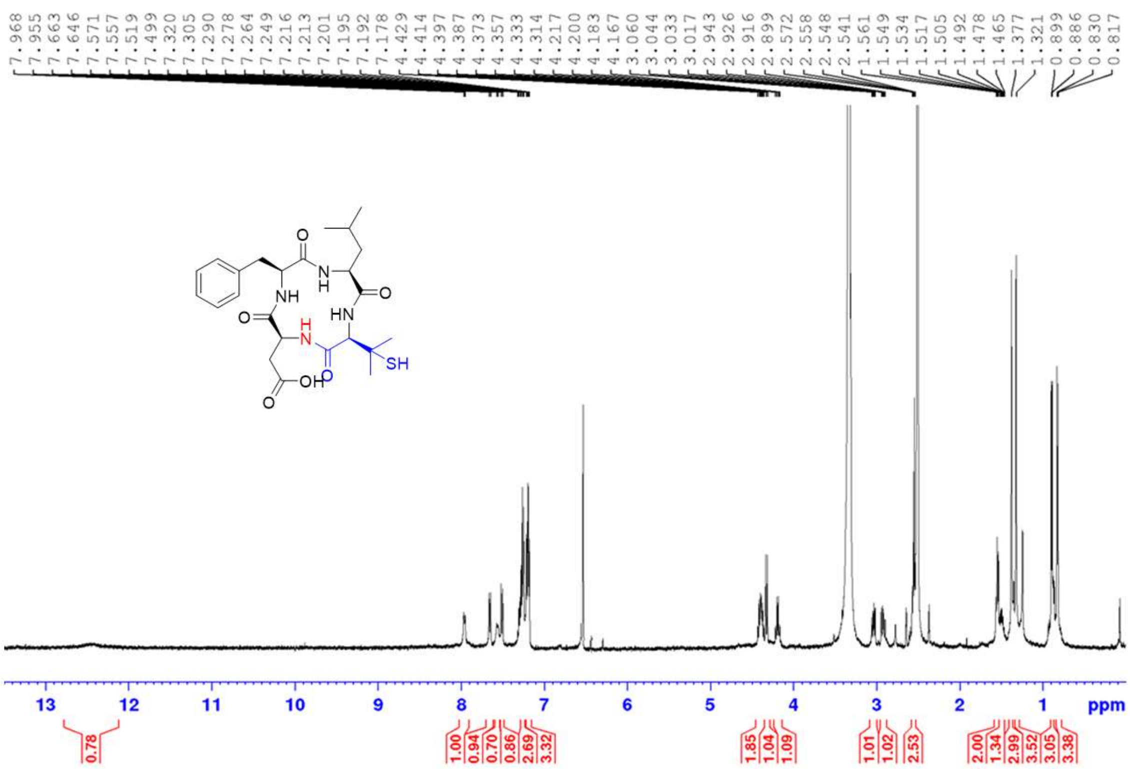

^{1}H NMR spectrum of compound **14u** (DMSO-d6, 500 MHz)

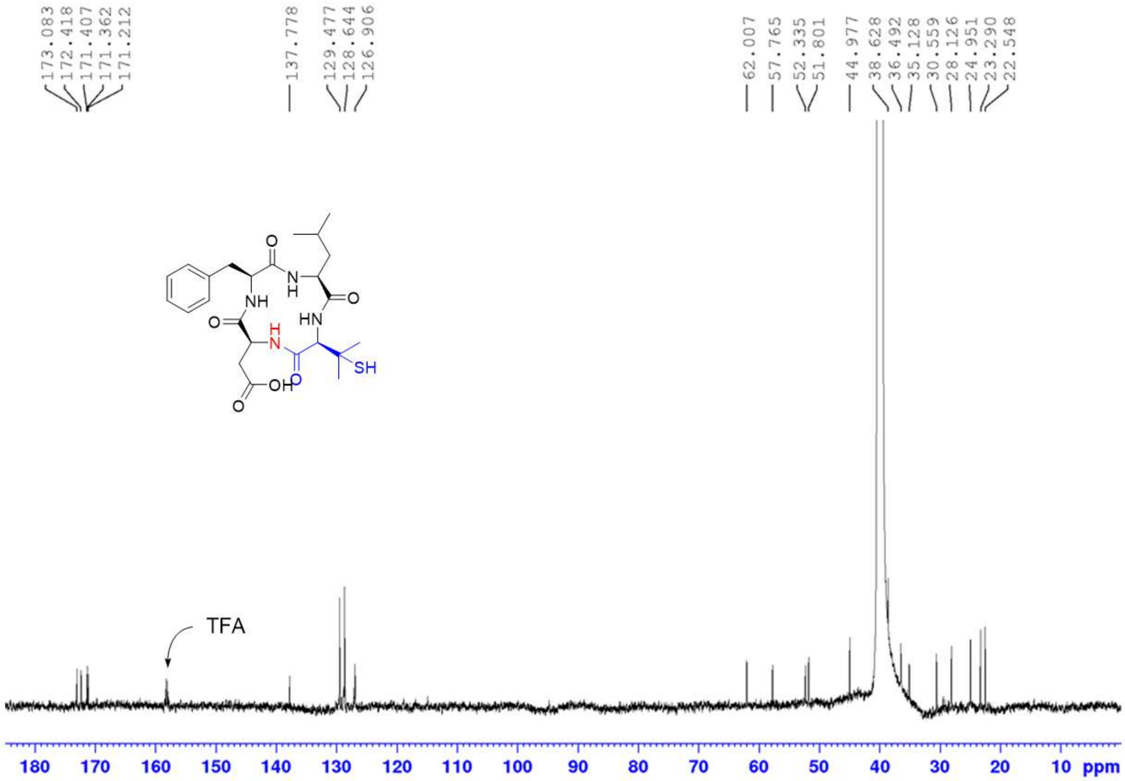

^{13}C NMR spectrum of compound **14u** (DMSO-*d6*, 151 MHz)

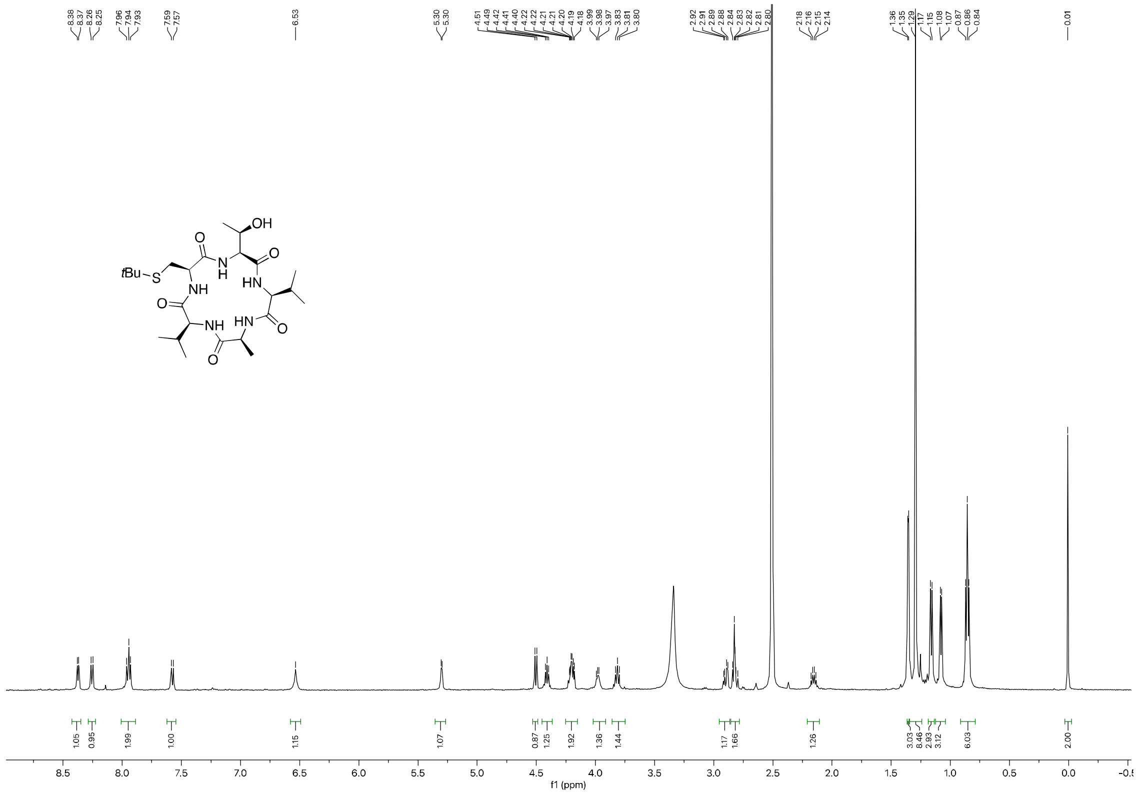

^{1}H NMR spectrum of compound **S14** (DMSO-d6, 600 MHz)

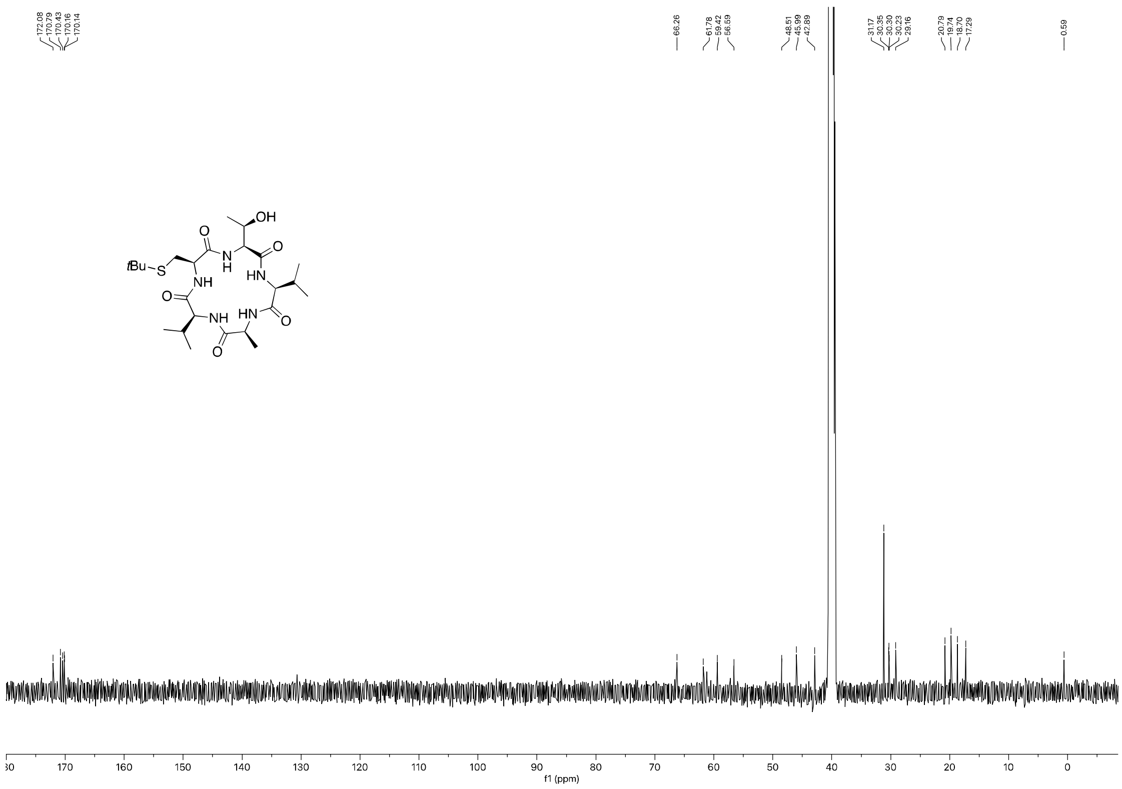

^{13}C NMR spectrum of compound **S14** (DMSO-*d6*, 151 MHz)

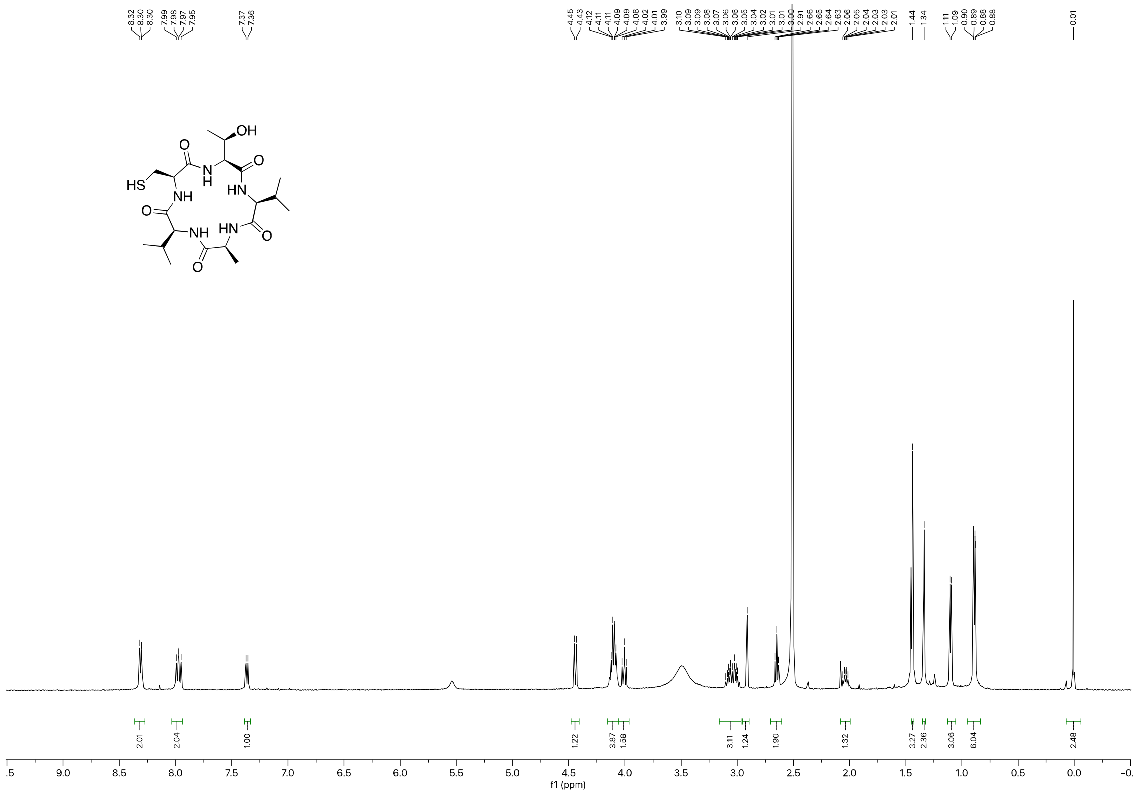

^{1}H NMR spectrum of compound **S16** (DMSO-d6, 600 MHz)

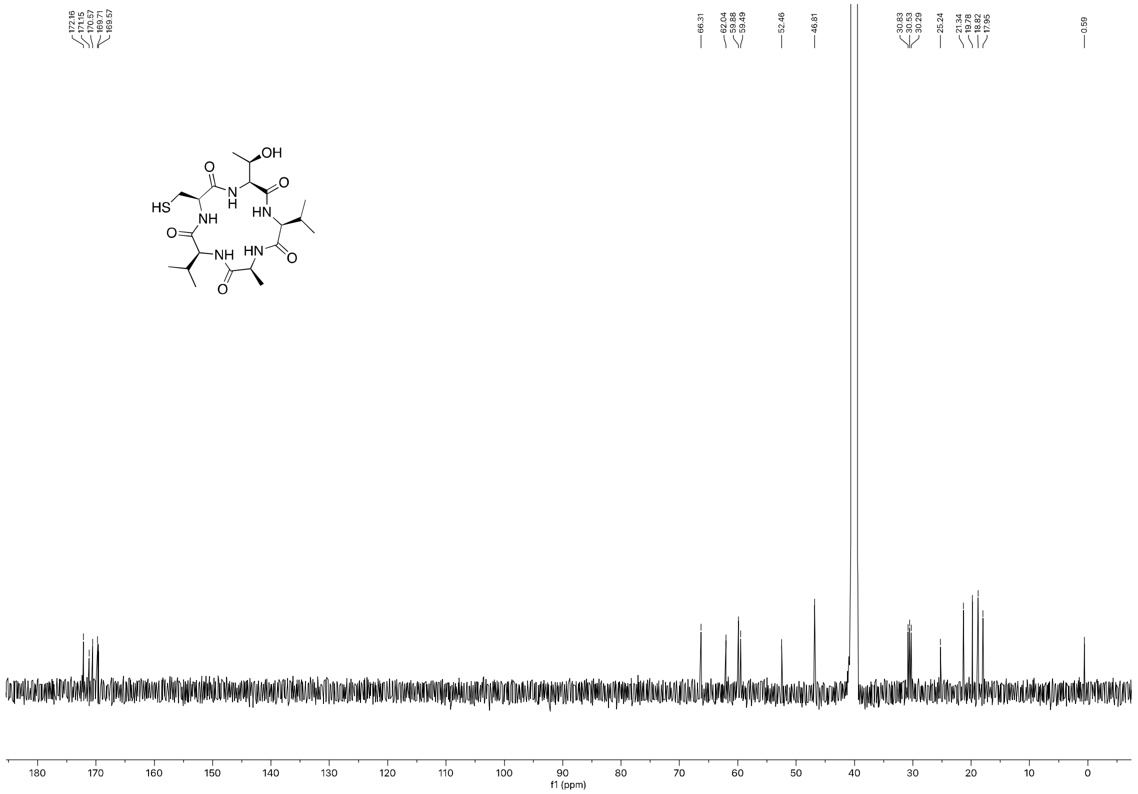

^{13}C NMR spectrum of compound **S16** (DMSO-*d6*, 151 MHz)

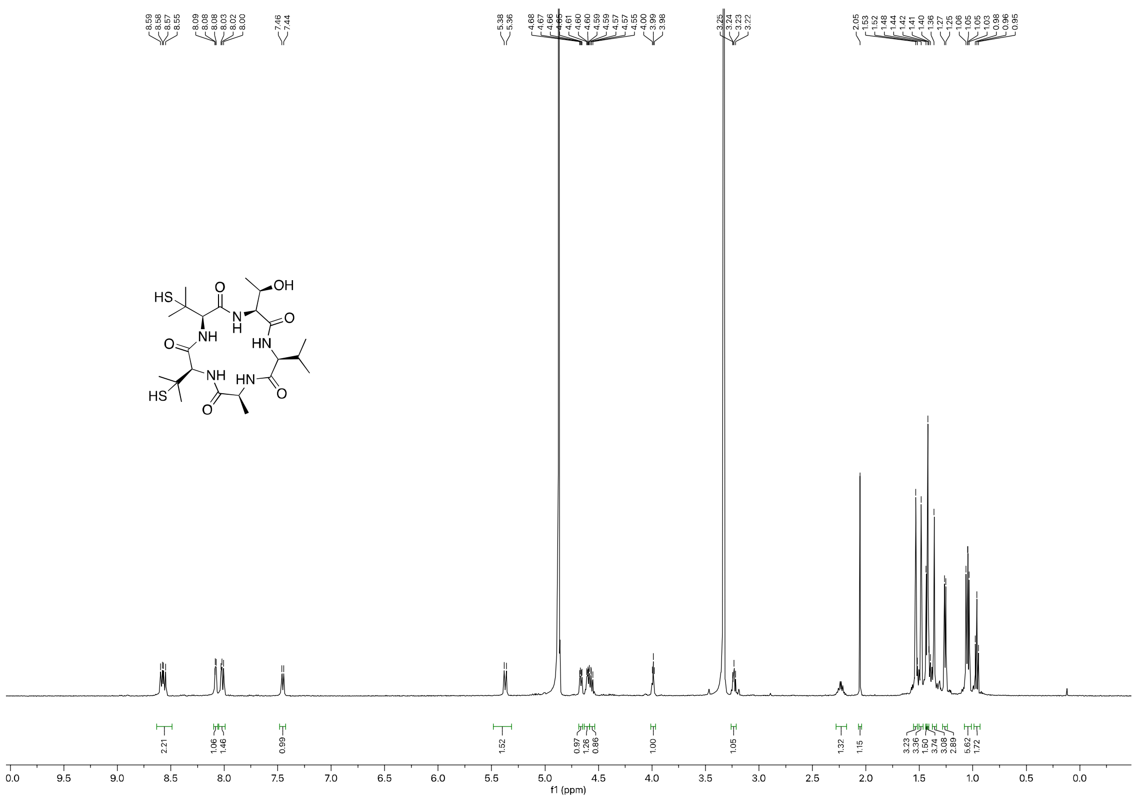

^{1}H NMR spectrum of compound **S18** (DMSO-d6, 600 MHz)

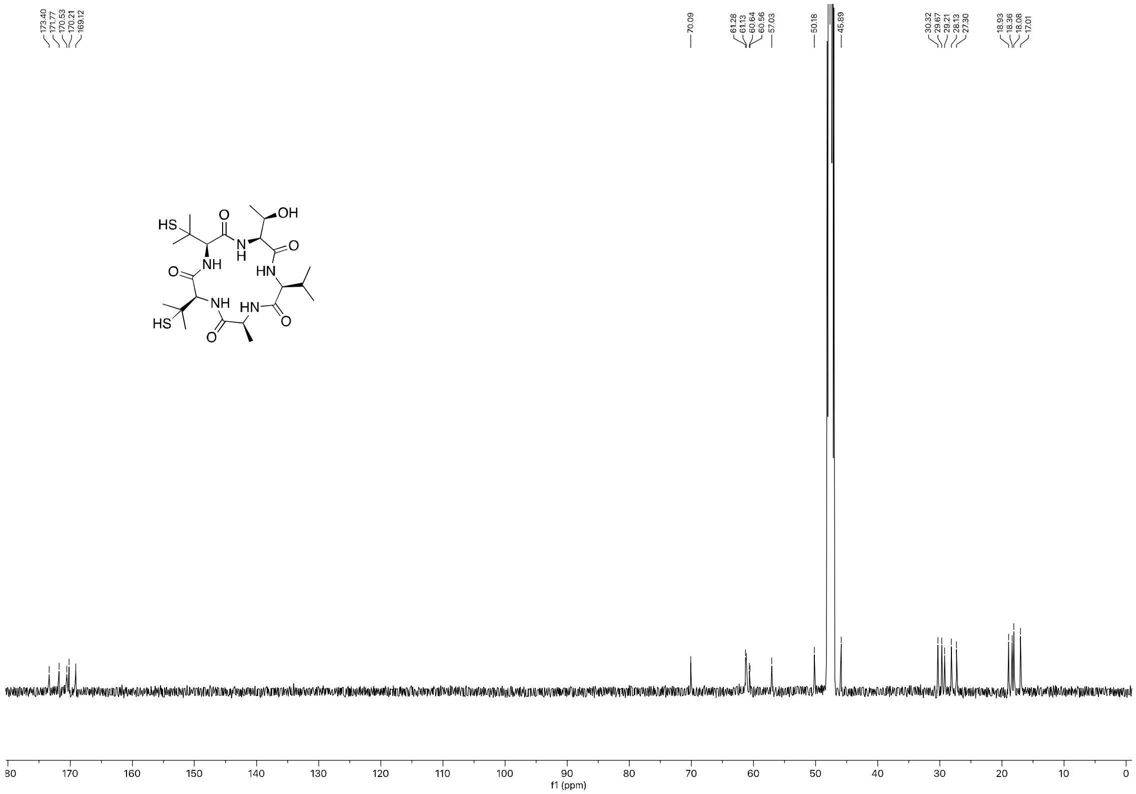

^{13}C NMR spectrum of compound **S18** (DMSO-*d6*, 151 MHz)

4. Supplementary References

1. Crich D., *et al.*, S_N2-Type Nucleophilic Opening of β-Thiolactones (Thietan-2-ones) as a Source of Thioacids for Coupling Reactions. *J. Org. Chem.* **2009**, *74*, 3389–3393.
2. Z. Tan *et al.* Insights into the Finer Issues of Native Chemical Ligation: An Approach to Cascade Ligations. *Angew. Chem. Int. Ed.*, **2010**, *49*, 9500 –9503.
3. D. Crich *et al.* Native Chemical Ligation at Phenylalanine. *J. Am. Chem. Soc.*, **2007**, *129*, 10064-10065.
4. *M.* L. Haddadi, *et al.,* All-L-Leu-Pro-Leu-Pro: A Challenging Cyclization *J. Peptide Sci*. **2000**, *6*, 560–570.
5. H. Chen, *et. al.*, Coupling of sterically demanding peptides by β-thiolactone-mediated native chemical ligation *Chem. Sci*., **2018**, *9*, 1982–1988.
6. K. Wüthrich, *NMR of Proteins and Nucleic Acids*, Wiley, **1986**.
7. Xu, S. *et al.* Ynamide-Mediated Peptide Bond Formation: Mechanistic Study and Synthetic Applications. *Angew. Chem. Inter. Ed.* **2022**, *61*, e202212247.
8. Ho, J., *et al.* Comment on the Correct Use of Continuum Solvent Models. *J. Phys. Chem. A*, **2010**, *114,* 13442-13444.
9. Gaussian 16 Rev. A.03 (Wallingford, CT, **2016**).
10. P. Pracht *et al.* Automated exploration of the low-energy chemical space with fast quantum chemical methods. *Phys. Chem. Chem. Phys.* **2020**, *22*, 7169-7192.
11. C. Bannwarth, *et al.* GFN2-xTB—An Accurate and Broadly Parametrized Self-Consistent Tight-Binding Quantum Chemical Method with Multipole Electrostatics and Density-Dependent Dispersion Contributions. *J. Chem. Theory Comput.* **2019**, *15*, 1652-1671.
